# Discovery of SHANK1-PDZ Peptide-Fragment Inhibitors Using a Dynamic Ligation Screening Strategy

**DOI:** 10.1021/acs.biochem.5c00804

**Published:** 2026-04-07

**Authors:** Yue Li, Diana Gimenez, Stuart L. Warriner, Andrew J. Wilson

**Affiliations:** † School of Chemistry, 1724 University of Birmingham, Edgbaston B15 2TT, U.K.; ‡ School of Chemistry, University of Leeds, Woodhouse Lane, Leeds LS2 9JT, U.K.; § Astbury Centre for Structural Molecular Biology, University of Leeds, Woodhouse Lane, Leeds LS2 9JT, U.K.

## Abstract

The development of ligands that modulate protein–protein interactions (PPIs) remains an ongoing challenge in chemical biology and drug discovery. While several approaches have been elaborated to target α-helix-mediated PPIs, methods for β-strand-mediated PPIs are less well developed. In addition to the shallow and extended interfaces characteristic of PPIs, β-strand-mediated PPIs exhibit topographical complexity, with side chains oriented above and below the plane of the strand, alongside hydrogen-bond donor and acceptor groups oriented perpendicular to the side chains. One class of β-strand-mediated PPIs involves the structurally conserved PDZ domains, which recognize protein partners through a β-strand containing a short consensus motif; canonical PDZ binding motifs (PBMs) recognize their substrates through a C-terminal carboxylate, offering a particularly challenging motif to mimic. Peptides and peptidomimetics represent a promising template for the design of ligands that target β-strand-mediated PPIs. In this work, we replaced segments of a peptide-based template using target/structure-agnostic fragments to achieve β-strand mimicry. Using reversible hydrazone exchange reactions allowed us to identify fragments at both the C- and N-terminus of an internal PDZ recognition motif with affinity for the SHANK1-PDZ domain. When combined into ligands bearing two different fragments, negative co-operativity was observed. In addition to broadening the acylhydrazone-fragment approach to screen for PDZ-binding ligands, this workflow for successive screening and combination of fragments should have broader applicability to other targets in future.

## Graphical abstract

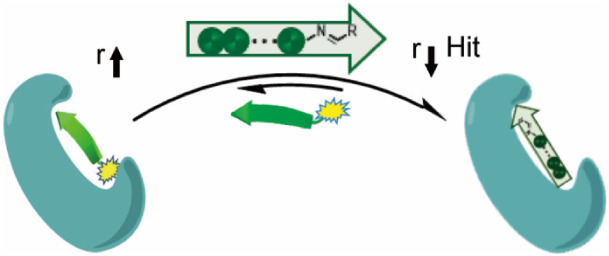

## Introduction

Proteins seldom perform their functions as isolated entities and, instead, are regulated through interactions with other proteins; such protein–protein interactions (PPIs) can misfunction, and therefore play a role in disease development and progression.^1^ Modulating PPIs has, therefore, come into focus for drug discovery; however, PPIs represent challenging targets given the larger surface area, lack of defined pockets, and disparate presentation of recognition handles at protein–protein interfaces.^2–4^ Small-molecule, peptide, and protein-based approaches for competitive (orthosteric) PPI inhibition have been developed, making the design of inhibitors, particularly for α-helix-mediated PPIs, more feasible;^4–7^ however, methods to target β-strand-mediated PPIs are less well established.^8–12^ Peptides and peptidomimetics are a particularly promising class of PPI inhibitors that occupy a unique chemical space between small molecules and proteins; peptides and peptidomimetics can mimic a native binding partner to achieve high affinity and selective target binding.^5,7^ For instance, mini- ^13,14^ and designed proteins,^15,16^ cyclic peptides,^17–19^ foldamers,^20–23^ and grafted proteins^24^ have all been shown to be suitable design templates for the inhibition of PPIs. These methods have been applied to a range of targets; however, while α-helix-mediated PPIs are well explored, β-strand-mediated PPIs are less so. β-strand-mediated PPIs exhibit topographic complexity with shallow and extended interfaces, making their modulation challenging.

An alternative approach to identifying PPI modulators is to replace segments of peptides using small-molecule fragments. The target/structure-agnostic nature of fragment screening should, in theory, render this approach well-suited to β-strand mimicry. In this strategy, a known peptide motif that recognizes a target protein is truncated and linked to different fragments to generate a hybrid with more desirable properties. Different truncations can be explored and then combined to replace significant elements of the peptide. An early example from 2006, termed “REPLACE” (Replacement with Partial Ligand Alternatives through Computational Enrichment), reported peptide-fragment hybrid inhibitors for CDK2/cyclin A substrate recruitment.^25–29^ More recently, peptide-fragment hybrid inhibitors of α-helix-mediated PPIs were developed using both computational and experimental screening.^30,31^ A further evolution of this approach has been to link a peptide to a fragment through a reversible bond-forming reaction and use the shift in equilibrium under template-assisted screening to identify hits.^32–35^ Our group recently reported the use of reversible hydrazone exchange^36^ as a tool to identify peptide-fragment hybrids as ligands for the SHANK1-PDZ domain.^33^ PDZ domains have proven to be challenging targets for ligand discovery.^37–40^ The highly conserved structure of PDZ domains recognizes protein partners through a β-strand containing a short consensus motif.^41^ Canonical PDZ binding motifs (PBMs) recognize their substrates through a C-terminal carboxylate.^42^ Perhaps unsurprisingly, in our original study^33^ we only identified potent peptide-fragment hybrid hits using N-terminal acyl hydrazones. In the current manuscript, we circumvent this limitation by using an internal PDZ binding motif as a starting template; this allows us to identify both C-terminal and N-terminal fragments, which can subsequently be combined into ligands bearing two different fragments (Figure 1). This workflow for successive screening and combination of fragments should have broader applicability to other targets in the future.

**Figure 1:**
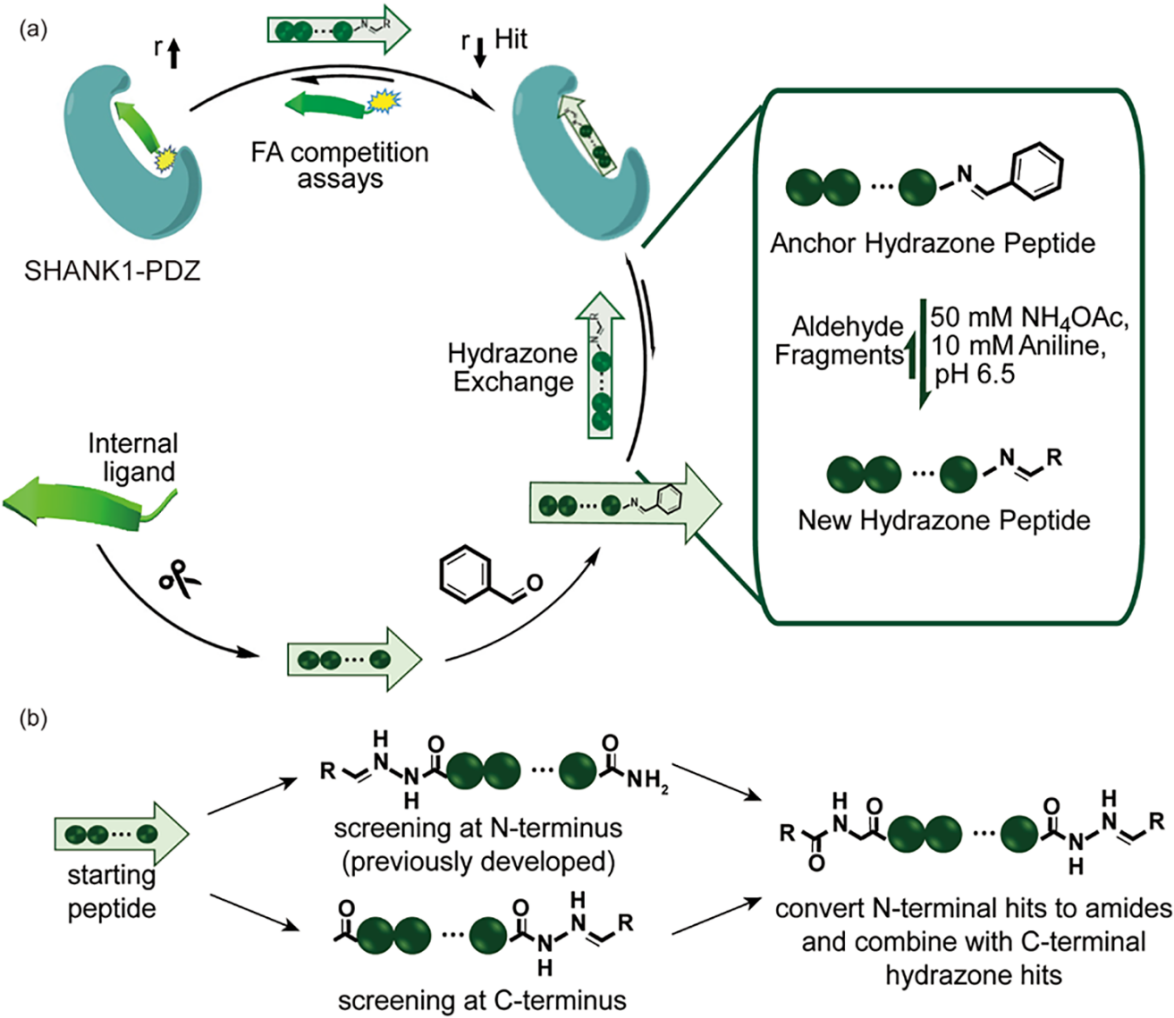
Study design: (a) General approach for acylhydrazone peptide-fragment hit identification incorporating sequence truncation; phenylhydrazone functionalization and hydrazone exchange reactions; and dynamic ligation screening. (b) Modifications explored and combined in this study.

## Materials and Methods

### Peptide Synthesis

Peptides were prepared using a CEM Liberty Blue or a Purepep Chorus 6 automated microwave synthesizer based on the Fmoc-based SPPS (solid-phase peptide synthesis) method. Rink-Amide MBHA resin (100–200 mesh) was used with a loading of 0.35 mmol/g. All Fmoc-protected amino acids and coupling reagents were purchased from Merck or Fluorochem. The procedures included four major steps: swelling, washing, deprotection, and amino acid coupling. Dichloromethane-treated resin was deprotected using 20% piperidine in DMF, so that the *N*-terminal amine group was exposed. The reagents DIC (5 equiv. in DMF) and Oxyma Pure (5 equiv. in DMF) were used to couple the corresponding amino acids at 90 °C for 5 min. The coupling step was repeated twice, and deprotection followed alternately. Following every step, the resin was washed with DMF three times and dried.

### Peptide *N*-Terminal Modification

*N*-terminally acetylated peptides were obtained by treatment of the resin-bound peptide with acetic anhydride–DIPEA (1:1, v/v, 10 equiv. in DMF) at room temperature for 30 min, followed by two washes with DMF and DCM solvents. *N*-terminally fluorescently labeled peptides were obtained by elongating the *N*-terminus with Fmoc-Ahx–OH (5 equiv. in DMF) and 5(6)-carboxyfluorescein or fluorescein isothiocyanate (FITC) (5 equiv. in DMF), respectively; both with *N*,*N*’-diisopropylcarbodiimide (DIC, 9 equiv. in DMF) and hydroxybenzotriazole (HOBt, 5 equiv. in DMF) as coupling reagents at room temperature for 4 h.

### Preparation of Fmoc-2-Chlorotrityl Hydrazine Resin

Prior to the synthesis of *C*-terminal hydrazone-functionalized peptides, Fmoc-2-chlorotrityl hydrazine resin was prepared.^53^ Ten gram of 2-chlorotrityl resin with a loading of 0.5 mmol/g was first swollen using a minimum volume of DCM in a round-bottom flask, followed by the addition of 5 equiv. of thionyl chloride. The mixture was stirred under nitrogen at room temperature for 2.5 h and transferred to a clean SPE fritted syringe. DMF and DCM were used to wash the resin three times. The resin was then treated with 5 equiv. of Fmoc hydrazide suspended in a DMF–DCM mixture (v/v, 1:1) and stirred for 1 h at room temperature. After that, the resin was flow-washed with about 20 mL of DMF and DCM alternately and then with diethyl ether.

A loading test was subsequently performed in triplicate. In each case, 20 mg of the resin was transferred into a 1.5 mL Eppendorf tube and suspended in 1 mL of 20% piperidine in DMF. After 5 min sonication, the tubes were rotated at room temperature for 30 min, followed by centrifugation at 5000 rpm for 5 min. After centrifugation, 10 μL of the supernatant was extracted and diluted to 1 mL using DMF. A blank sample was prepared as 0.2% piperidine in DMF. A Nanodrop spectrometer was first blanked and then used to check the samples’ absorbance at a wavelength of 301 nm. The resin loading was calculated according to the formula: as L (mmol/g) = (101 × A301)/(7.8 × weight [mg]). Averaged results from the triplicate samples were used to determine the final loading of the resin.

### *C*-Terminal Hydrazone Functionalization

For the synthesis of the *C*-terminal peptide hydrazones, crude peptide hydrazides were first synthesized using Fmoc-2-Chlorotrityl hydrazine resin, as described above. After the *N*-terminal acetylation and cleavage, the crude hydrazide products were freeze-dried. Peptide hydrazones were obtained by mixing 10 equiv. of aldehyde fragments with peptide hydrazides in DMSO. LC-MS was used to monitor the conversion rate of the reactions. Once completed, the crude products were purified by preparative HPLC.

### Preparation of the Linker 1-[2-[(1,1-Dimethylethoxy)­carbonyl]­hydrazide

Prior to the synthesis of the *N*-terminal peptide hydrazones, a linker was first prepared. succinic anhydride (10g, 100 mmol) and *tert*-butylhydrazinecarboxylate (13.2g, 100 mmol) were dissolved in 100 mL of water. After stirring at room temperature for 4 h, the reaction mixture was lyophilized using a freeze-dryer. ^1^H NMR (400 MHz, d6-DMSO, 298 K): (ppm) = 12.39–11.69 (br s, 1H, CH_2_COO ***H***), 9.53 (s, 1H, N ***H*** C­(O)­CH_2_), 8.69 (s, 1H, N ***H***-*t*Boc), 2.43 (t, 2H, C ***H***_2_C­(O)­NH, J = 6.8 Hz), 2.35–2.39 (m, 2H, C ***H***_2_COOH), 1.36 (s, 9H, C­(C ***H***_3_)_3_). ESI-MS calcd. for C_9_H_16_N_2_O_5_ ([M-1H]-): 231.0986, found 231.0983. These data agree with those reported in the literature.^33^

### *N*-Terminal Hydrazone Functionalization

The synthesis of the *N*-terminal peptide hydrazones was performed using Rink-Amide MBHA resin (100–200 mesh, 0.3–0.8 mmol/g) as described above. When completed, 5 equiv. of the linker 1-[2-[(1,1-dimethylethoxy)­carbonyl]­hydrazide, 9 equiv. of DIPEA and 5 eq of HCTU in DMF were mixed and added to the resin. After coupling for 2 h at room temperature, the resin was washed with DMF and DCM three times, followed by the general cleavage and lyophilization steps. Crude *N*-terminal hydrazides were mixed with a 10-fold of aldehyde fragments in DMSO, and LC-MS was used to monitor the formation of hydrazone. Once completed, crude products were purified by preparative HPLC.

### Peptide Cleavage

After synthesis, all peptides were obtained by treatment with a cleavage cocktail (TFA/triisopropylsilane/2,2-(ethylenedioxy)­diethanethiol/water, 92.5:2.5:2.5:2.5, v/v/v/v %) at room temperature for 3.5 h. Peptide products were precipitated using cold diethyl ether and then centrifuged. Precipitates were collected and dissolved in 10% acetonitrile in water, then freeze-dried for purification. An Agilent 1260 Infinity HPLC and Bruker maXis II ESI–QTOF mass spectrometer were used to purify the crude peptides and acquire analytical HPLC and high-resolution mass spectrometry data, respectively.

### Synthesis of Ternary Peptide-Fragment Hybrids

#### Attachment of Fmoc-5ava–OH on the N-Terminus

For the synthesis of the ternary peptide-fragment hybrids, peptide motifs were first synthesized on the automated synthesizer using the preprepared 2-chlorotrityl hydrazide resin. Fmoc-5ava–OH (5 equiv. in DMF), together with the coupling reagents DIC (9 equiv. in DMF) and HOBt (5 equiv. in DMF), were mixed with the resin and rotated at room temperature for 4 h. . Mini-cleavage was done with 100-200 *μ*L of the cocktail (TFA/triisopropylsilane/2,2-(ethylenedioxy)­diethanethiol/water, 92.5:2.5:2.5:2.5, v/v/v/v %). LC-MS was used to check the molecular weight of the product. Once successful coupling had been established, 5 mL of 20% piperidine in DMF was used to deprotect the *N*-terminal Fmoc group of the resin twice. Three milliliter of DMF and DCM were used to wash the resin alternately after every step.

#### Synthesis of Binary Peptide-Fragment Hybrids with N-Terminal Amide Bond

Five equivalents of the acid analog of the selected fragment were first dissolved in 3 mL of dry DMF and activated using 9 equiv. Ghosez’s Reagent in a sealed round-bottom flask with N_2_ protection at 50 °C for 3 h. After the activation, the mixture, together with 9 equiv. DIPEA was added into the above resin and rotated at room temperature for 4 h. The coupling reaction was repeated 2 to 3 times. Mini-cleavage was performed with 1 mL of the cocktail (TFA/triisopropylsilane/2,2-(ethylenedioxy)­diethanethiol/water, 92.5:2.5:2.5:2.5, v/v/v/v %). LC-MS was used to check the molecular weight of the binary peptide-fragment hybrids.

#### Synthesis of Ternary Peptide-Fragment Hybrids

After the general cleavage, the crude powder of the binary peptide-fragment hybrids was obtained by using the freeze-dryer. Similar to the steps for synthesizing the *C*-terminal hydrazone, the final products were obtained by mixing 10 equiv. of aldehyde fragments with peptide hydrazides in DMSO. LC-MS was used to monitor the conversion rate of the reactions. Once completed, the crude products were purified by preparative HPLC.

#### Protein Expression and Purification

Human SHANK1 PDZ domain (656–762) was prepared as described previously.^43^ Briefly, the domain was cloned into the pGEX-6P-2 expression vector and transformed into BL21 gold cell lines for expression. Ten milliliter overnight starter culture was inoculated in 1L commercially available LB broth (Miller) containing 50 μg/mL chloramphenicol. Cells were incubated with shaking at 37 °C until OD_600_ ∼ 0.6–0.8, then induced with 0.1 mM IPTG overnight at 18 °C. Cell pellets were harvested by centrifugation with a Beckman Coulter JA-10 rotor at 16,000 g relative centrifugal force (RCF) for 60 min at 4 °C using an Avanti JXN-30 Beckman Coulter centrifuge and resuspended in 20 mM Tris, pH 8, 500 mM NaCl buffer containing 1 mg lysozyme, 0.5 mg DNase, and 1/6 cOmplete^TM^, mini EDTA-free protease inhibitor cocktail tablet. Cells were then lysed by sonication (8 cycles, 20 s on 40 s off, 10 μA) and centrifuged with a Thermo Scientific TX-400 rotor at 1,500 g RCF for 25 min at 4 °C using a Fisherbrand GT 2R centrifuge. The supernatant was filtered using a 0.45 μm membrane and applied to glutathione beads. Ten column volumes of 20 mM Tris, pH 8, 500 mM NaCl buffer were used to wash the beads, then 20 column volumes of elution buffer consisting of 20 mM Tris, pH 8, 150 mM NaCl, and 25 mM glutathione. Collected fractions were analyzed by sodium dodecyl sulfate-polyacrylamide gel electrophoresis (SDS-PAGE). PreScission protease was added to the elution fraction, and the GST tag was cleaved overnight at 4 °C. The elution fraction was then concentrated and reapplied to glutathione beads. The newly eluted fraction was purified by size-exclusion chromatography on an S75 26/60 pg column in 20 mM Tris, 150 mM NaCl, pH 7.5 buffer. Pure protein was analyzed by high-resolution mass spectrometry: expected *m*/*z* = 12326.3, measured *m*/*z* = 12325.6. Concentration was determined by Nanodrop using 8480 M^–1^cm^–1^ as the extinction coefficient.

### Fluorescence Anisotropy Assays

#### Direct Titration

Direct titration assays were performed in 384-well plates (Greiner Bio-One). 200 μM SHANK1 protein was dialyzed into the assay buffer before use. Assay buffer (20 μL, 50 mM ammonium acetate NH_4_OAc, pH 6.5) was first added to each well. Target protein (20 μL) sample was then added to the first column, followed by a 2-fold serial dilution over 24 points. Tracer peptide (20 μL, 50 nM) or assay buffer (20 μL) was added to the corresponding row wells. The titration was performed in triplicate. Plates were read immediately, and after an hour or after 24 h on a PerkinElmer EnVision^TM^ 2103 MultiLabel plate reader, with excitation at 480 nm (30 nm bandwidth), polarized dichroic mirror at 505 nm, and emission at 535 nm (40 nm bandwidth, S and P polarized) at a controlled temperature of 25 °C. The P (perpendicular intensity) and S (parallel intensity) channels’ raw data were obtained. The intensity and anisotropy were calculated by using eqs 1 and 2. The data were fitted to a sigmoidal logistic model to obtain *r*_*min*_ and *r*_*max*_, and *L_b_* was further calculated (eq 3). Finally, the data were fitted to a nonlinear curve model (eq 4) to obtain the value of *K_D_*.

1 I=(2PG)+S

2 r=(S−PG)/I

3 $$L_{b}=\left(r-r_{min}\right)/\left[\lambda \left(r_{max}-r\right)+\left(r-r_{min}\right)\right]$$

4 $$y=\left\{\left(k+X+\left[FL\right]\right)-\sqrt{\left\{k+X+{[FL]}^{2}-4^{*}\left[FL\right]\right\}}\right\}/2$$

*I* = total intensity, *r* = anisotropy, *P* = perpendicular intensity, *S* = parallel intensity, *G* is an instrument gain factor, *L_b_* = fraction ligand bound, *λ* = I_bound_/I_unbound_ = 1, [*FL*] = concentration of fluorescent ligand, *k* = *K_D_* and *x* = [added titrant].

#### Competition Assays

Fluorescent anisotropy competition assays were also performed in 50 mM NH_4_OAc, pH 6.5 buffer, in 384-well plates. Assay buffer (20 μL) was first added to the wells. Competitor peptides (20 μL, 5000 μM) was added to the first columns, followed by a 2-fold serial dilution over 16 points. SHANK1 protein (3 μM) was dialyzed into the assay buffer before use. SHANK1 protein (20 μL) was added to each well to give a final protein concentration of 1 μM. Tracer peptides (20 μL, 50 nM) or assay buffer (20 μL) was added to the corresponding row wells. The titration was performed in triplicate. Plates were read immediately with an excitation and emission wavelength of 480 and 535 nm, respectively (dichroic mirror 505 nm), and after an hour or after 24 h on the plate reader with the same parameters as described above. The calculated average anisotropy values and their standard deviation were fitted using a sigmoidal logistic model (eq 5).

5 $$y=r_{\max}+\left(r_{min}-r_{\max}\right)/\left(1+{\left(X/X_{0}\right)}^{p}\right)$$

*y* = *r* = anisotropy, *x*_0_ = midpoint of the curve between the *r*_*max*_ and *r*_*min*_ plateau.

#### Hydrazone Exchange Reactions

Hydrazone exchange was performed in a buffer comprising 50 mM NH_4_OAc, 10 mM aniline, pH 6.5. 100 μM of peptide hydrazones were mixed with 5 equiv. of aldehyde fragments at ambient temperature. LC-MS was used to monitor the conversion rate of the reactions at 1, 2, 4, 8, 12, 24, and 48 h. The proportions of reactant and resultant hydrazones were calculated by integrating and normalizing the peak areas for extracted ion chromatograms. The visualization of graphics was achieved by using Origin2020.

#### Hydrazone Exchange Screening

Screening was performed in a buffer comprising 50 mM NH_4_OAc, 10 mM aniline, pH 6.5. Before the experiments, SHANK1-PDZ (656–762) protein and the reactant phenylhydrazone were first dialyzed into the buffer. The reactant phenylhydrazones (10 μL) was added into each well, followed by the addition of 5 equiv. of the 165 aldehyde fragments in duplicate. Twenty microliter of the SHANK1-PDZ protein was then pipetted into each well, and the plates were sealed with a final concentration of 1 μM and placed at ambient temperature. After 24 h, 50 nM of the tracer peptide FAM-Ahx-EESTSFQGP-CONH_2_ or the buffer was added into each well. For control groups, 10 μL of the samples and DMSO were added into each well, followed by the addition of 20 μL of 3 μM protein. Plates were read immediately on a PerkinElmer EnVision^TM^ 2103 MultiLabel plate reader.

#### Molecular Dynamics (MD) Analyses

All peptide–protein complexes were subjected to duplicate MD simulations using YASARA Structure.^44^ Starting from the reported X-ray crystal structure of one molecule of SHANK1 PDZ in complex with a template SLiM internal ligand (PDB: 8S1R), single amino acid modifications were introduced by direct replacement or deletion of the relevant atoms within the structure. *N*-terminal and *C*-terminal fragments were manually modeled into the peptide prior to analysis, and then minimized structures were generated using the energy minimization function with default settings. The modeled complexes were subjected to MD simulations using the YASARA Structure macro for fast MD runs (www.yasara.org/md_runfast.mcr).^45^ Briefly, the AMBER14 force field was used, and the temperature was set to 298.0 K, with the time step set at 1 × 2.50 fs per frame. Each complex was run in independent duplicates for 125 ns (500 frames). Minimum energy, average structures, and the individual contacts between the corresponding fragments and the PDZ protein were analyzed from these experiments (www.yasara.org/md_analyze.mcr), and figures were created using the same software.

#### Isothermal Titration Calorimetry (ITC) Analyses

ITC experiments were carried out using a MicroCal PEAQ-ITC system (Malvern Panalytical) at 25 °C. A stock solution of SHANK-1 PDZ was prepared by dialysis of the protein into ITC buffer overnight (25 mM Tris buffer, pH= 7.5; 150 mM NaCl). The same buffer was employed to resuspend a solid portion of the testing peptides into 1 mM stock solutions. For each experiment, initially, the cell was filled with 275 μL of the protein solution [25 μM], and the syringe was loaded with the corresponding peptide at a 500 μM (N1C3T-[N086] and N1C3T-[N086–C047]) or 1 mM concentration in the case of N1C3T-[C047]. An initial injection of 0.2 μL was then followed by 19 injections of 3 μL every 150 s, with a constant syringe rotation speed of 750 rpm throughout. The signal for the enthalpy of dilution of each peptide into the buffer was independently measured in a similar control titration experiment in the absence of protein and then subtracted from the experimental binding traces. The *K*_d_, ΔG°, ΔH°, and −TΔS° values were determined via the MicroCal PEAQ-ITC analysis software (Malvern Panalytical) using a single-site binding model. The values reported are the mean of duplicate measurements. We note that when employing the doubly capped peptide, N1C3T-[N086–C047], protein saturation could not be achieved.

## Results and Discussion

In addition to C-terminal PBMs, internal PBMs have also been identified and characterized. Davey et al. proposed a novel consensus TxF motif for SHANK1-PDZ non-C-terminal ligands and discovered several new or previously validated internal PBMs using proteomic peptide phage display (ProP-PD).^46^ Ali et al. established a consensus motif x-T-x-F-x (x is any amino acid) for SHANK1 internal PBMs and characterized several internal PBMs, among which the peptide ARAP3 had good SHANK1-PDZ binding potency (K_d_ = 3.0 (± 0.3) μM).^47^ The dominant sequence identified through position-specific scoring matrices (PSSMs), representing the binding-enriched sequence from ProP-PD, was Glu-Glu-Ser-Thr-Ser-Phe-Gln-Gly-Pro. We subsequently carried out biophysical and structural studies on this sequence (K_d_ = 0.81 ± 0.08 μM).^48^ For the current study, we selected this sequence to perform truncation and subsequently dynamic hydrazone-fragment ligation screening, given its slightly higher potency and good solubility that facilitated biophysical studies in our prior study.

We next truncated the template sequence from its N-terminus, C-terminus, or both sides to identify the shortest sequences that retained measurable SHANK1-PDZ binding affinity, and for which hydrazone-fragment ligation would be feasible. Seven N-terminally acetylated sequences with C-terminal amidation were obtained and screened in competition fluorescence anisotropy (FA) assays using the FAM-Ahx-EESTSFQGP-NH_2_/SHANK1-PDZ (Figure 2). In fluorescence anisotropy, polarized light is used to measure the rotation of a molecule between the absorption and emission of photons, allowing analyses of its movement, orientation, size, and shape. When a fluorescently labeled peptide (the tracer) binds to a protein, its anisotropy tends to increase due to the effective increase in molecular weight and, therefore, the slower rotation it experiences. For competition FA, the anisotropy loss upon displacement of the tracer by the competitor is used to determine potency.^49^

**Figure 2:**
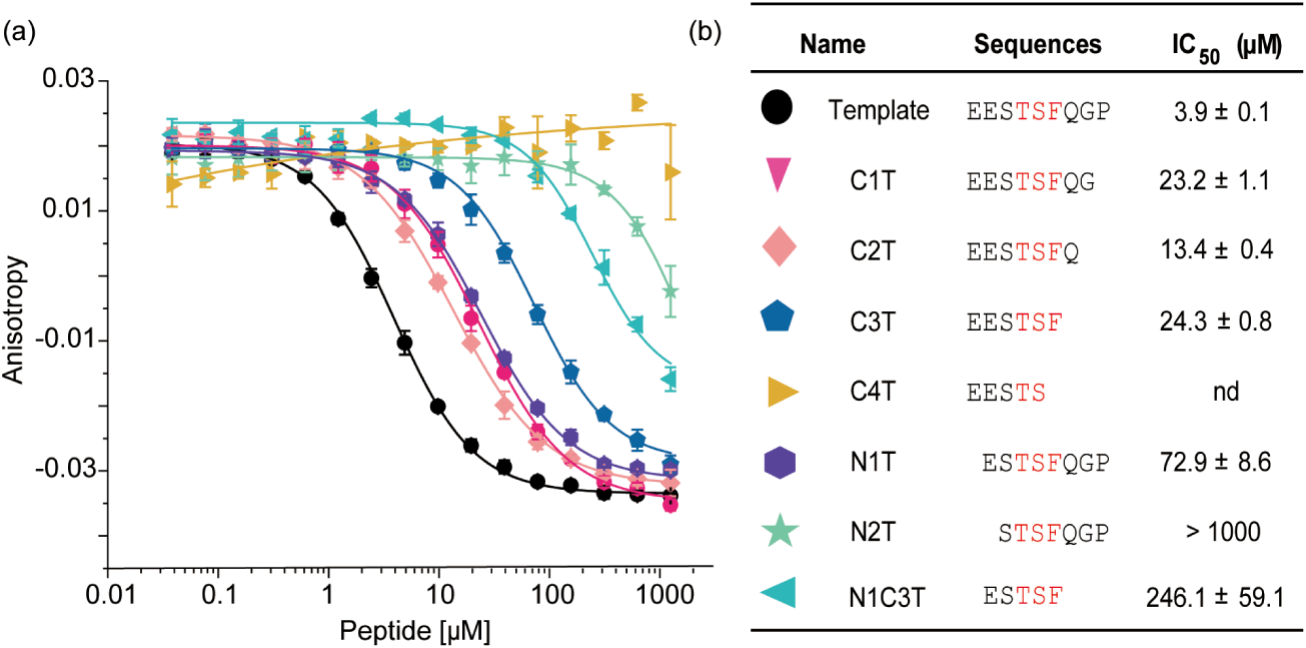
(a) FA competition assay titration data of the full-length internal ligand (template) and the truncated sequences (3 μM SHANK1-PDZ, 50 nM FAM-Ahx-EESTSFQGP-NH_2_ 50 mM NH_4_OAc, pH 6.5, using as tracer; sequences prepared as C-terminal amides with N-terminal acetylation). (b) IC_50_ values for the given sequences.

Removal of three C-terminal residues, Gln (1), Gly (2), and Pro (3), from the template peptide generated sequences C1T, C2T, and C3T that retained affinity (IC_50_ = 23.2 ± 1.1 μM, 13.4 ± 0.4 μM, and 24.3 ± 0.8 μM), whereas removal of Phe (0) resulted in complete loss of inhibition. The results suggest that none of Gln (1), Gly (2), and Pro (3) make strong noncovalent interactions with SHANK1-PDZ (consistent with structural analyses; see Figure S1). The loss in potency relative to the full-length sequence (IC_50_ = 3.9 ± 0.1 μM) may arise due to differential solvation effects or loss of van der Waals contacts. At the N-terminus, peptides lacking one or two N-terminal glutamates (N1T and N2T) showed a decrease in inhibitory potency (IC_50_ = 72.9 ± 8.6 μM and >1 mM, respectively). Both Glu (−5) and Glu (−4) form multiple hydrogen bonds with the target residues on SHANK1-PDZ, offering an explication for the sharp decrease in inhibitory potencies. Truncation at both termini, giving peptide N1C3T retaining the key residue Phe (0) but lacking one N-terminal glutamate, led to the minimal sequence with inhibitory potency (IC_50_ = 246.1 ± 59.1 μM), which we considered to be a suitable starting point for screening.

To obtain acylhydrazones, we chose to freshly prepare Fmoc-capped 2-chlorotrityl hydrazine resin and carried out solid-phase peptide synthesis. Following cleavage, crude peptides were reacted with a simple aldehyde fragment (benzaldehyde) to generate three acylhydrazone analogues: C1T-[C001], C2T-[C001], and C3T-[C001] (Figure S2). The inhibitory activities in the FAM-Ahx-EESTSFQGP-NH2/SHANK1-PDZ FA competition assay (Figure S3) were comparable to the parent C-terminal sequence (IC_50_ = 17.6 ± 1.5 μM for C1T-[C001], 11.0 ± 1.4 μM for C2T-[C001], and 19.9 ± 1.0 μM for C3T-[C001]).

We then explored hydrazone exchange of the corresponding acylhydrazone peptides using our previously assembled 165-fragment aldehyde library.^33^ C- or N-terminally truncated peptide-hydrazone candidates were identified with low-micromolar inhibitory activities comparable to the starting sequence (Ac-EESTSFQGP-NH_2_). Briefly, fragment selection followed a relaxed “rule of three”,^33,50^ to account for the incorporation of formyl groups in the structure (parameter ranges: 80 Da < M_W_ < 400 Da, hydrogen bond acceptor (HBA) and hydrogen bond donor (HBD) ≤ 3, clogP ≤ 5). DataWarrior (openmolecules.org) was used to remove structurally similar compounds, leading to a structurally dissimilar subset of fragments. Following further exclusion of undesired functional groups, 165 commercially available compounds were finally selected for the aldehyde library. Using C1T-[C001], C2T-[C001], and C3T-[C001], hydrazone exchange experiments were performed with a 5-fold excess of exemplar aldehyde fragments in 50 mM NH_4_OAc, 10 mM aniline, pH 6.5, at ambient temperature.^51^ Time course experiments (Figure S4a–c) established that 24 h was practical e.g., for the exchange reaction, the conversion rate achieved was 86–88% and 87–91% within 24 and 48 h, respectively.

Screening was performed with a 5-fold excess of aldehyde fragments in 384-well plates in 50 mM NH_4_OAc, 10 mM aniline, pH 6.5. The final concentrations of the anchor acylhydrazone peptide, SHANK1-PDZ, and the tracer (FAM-Ahx-EESTSFQGP-NH_2_) used in these experiments were based on the FA competition assay results (C1T-[C001] = 20 μM, C2T-[C001] = 10 μM, C3T-[C001] = 20 μM, SHANK1-PDZ = 3 μM, and FAM-Ahx-EESTSFQGP-NH_2_ = 50 nM; see Figure S5a–c). In all screening experiments, anisotropy values (*r*) were expressed relative to the buffer, and errors were calculated based on the standard deviation of sample duplicates. Two thresholds (negative or positive) were set: the mean relative *r* values of the buffer or corresponding anchor acylhydrazone peptides, by subtracting three standard deviations.

Fifteen hits were obtained from the screen with C1T-[C001], of which 8 hits were located below the threshold of the positive control (C1T-[C001] r_rel‑3σ_, green, Figure S5a). The most promising peptide-fragment hybrids were synthesized, purified, and assessed in dose-response FA competition assays. Their structures and IC_50_ values are shown in Table 1 (see ESI Figure S6 for FA competition curves). Among them, two hits, C1T-[C023] (IC_50_ = 3.6 ± 0.6 μM) and C1T-[C088] (IC_50_ = 5.8 ± 0.7 μM), showed the best inhibitory activities. The identified fragments contain aromatic rings and may compensate for the loss of the pyrrolidine ring of Pro(3). The hits C1T-[C007] andC1T-[C023] contain a carboxylate group in their structure, and the two most potent hybrids, C1T-[C023] and C1T-[C088], contain an additional hydroxyl group, both of which may facilitate noncovalent interactions with PDZ residues. For C2T-[C001], four hits—C2T-[C007], C2T-[C012], C2T-[C047], and C2T-[C101]—were obtained (Figure S5b). Hit validation of purified hits using FA competition assays established low-micromolar inhibitory potencies that were comparable to the full-length internal ligand Ac-EESTSFQGP-CONH_2_ (IC_50_ = 2.3 ± 0.4 μM for C2T-[C007], 1.9 ± 0.2 μM for C2T-[C012], 3.0 ± 0.6 μM for C2T-[C047], and 1.3 ± 0.3 μM for C2T-[C101], Table 1, Figure S6). All four hits have at least one aromatic ring to compensate for the truncated C-terminal motif “Gly (2)-Pro (3)”. While fragment 007 appeared for both C1T and C2T, as for C1T, it contains functional groups that may make additional noncovalent contacts with the PDZ domain. Finally, for C3T-[C001], two hits were obtained (Figure S5c). FA competition assays validated these and established inhibitory activities (IC_50_ = 7.0 ± 0.7 μM for C3T-[C047] and 19.9 ± 1.0 μM for C3T-[C109], Table 1, Figure S6). As with the screens using C1T-[C001] and C2T-[C001], the two hits obtained from the screen of C3T-[C001] also contain two aromatic rings. However, the two fragments’ structures show little similarity to the truncated residue Gln (1). Compared to C3T-[C109], there is an additional side-chain isopropyl group in C3T-[C047], which may occupy more space at the interface with the SHANK1-PDZ backbones and therefore show relatively higher inhibitory potency (compound C3T-[047] may be a mixture of regioisomers because both carbonyl groups can potentially react). To explore correlations between potency and molecular properties, we plotted relative anisotropy values for the hits against molecular weight and calculated LogS. Hits (green and red balls) from all three screens are randomly distributed, indicative of little correlation between potency and physicochemical characteristics. Hits (red balls) had molecular weights around 150 to 250 Da and negative LogS values (Figure 3a–c and Figure S7).

**Table 1:**
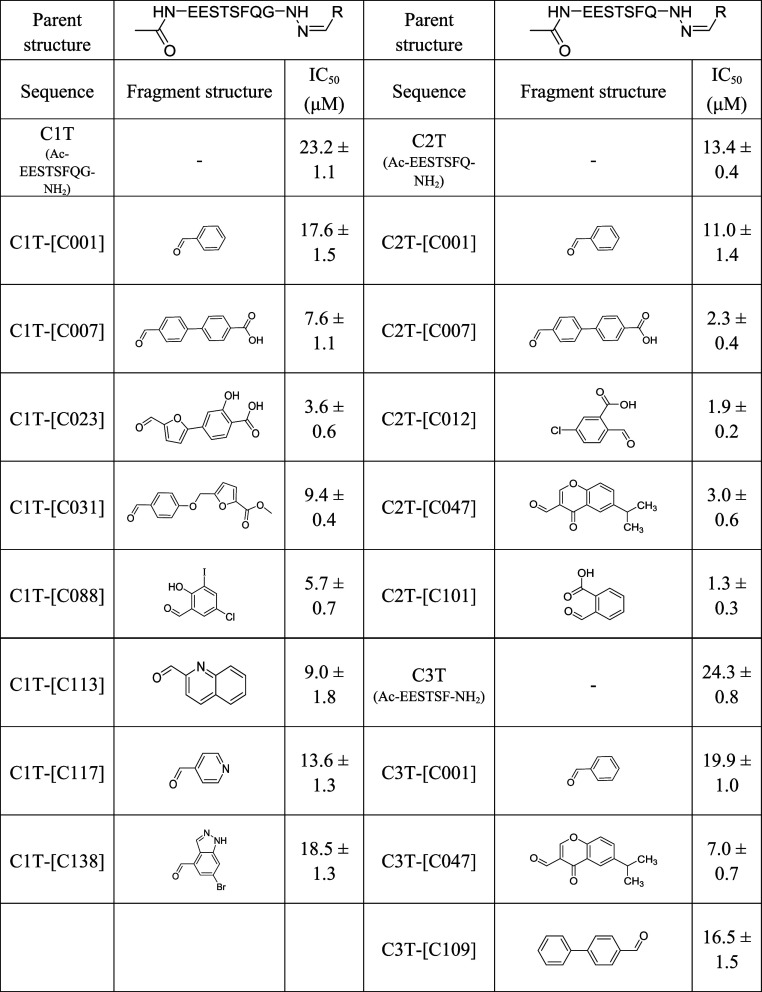
Summary of the Hits’ Structures and Potencies from Screening on C1T-[C001], C2T-[C001], and C3T-[N001]

**Figure 3:**
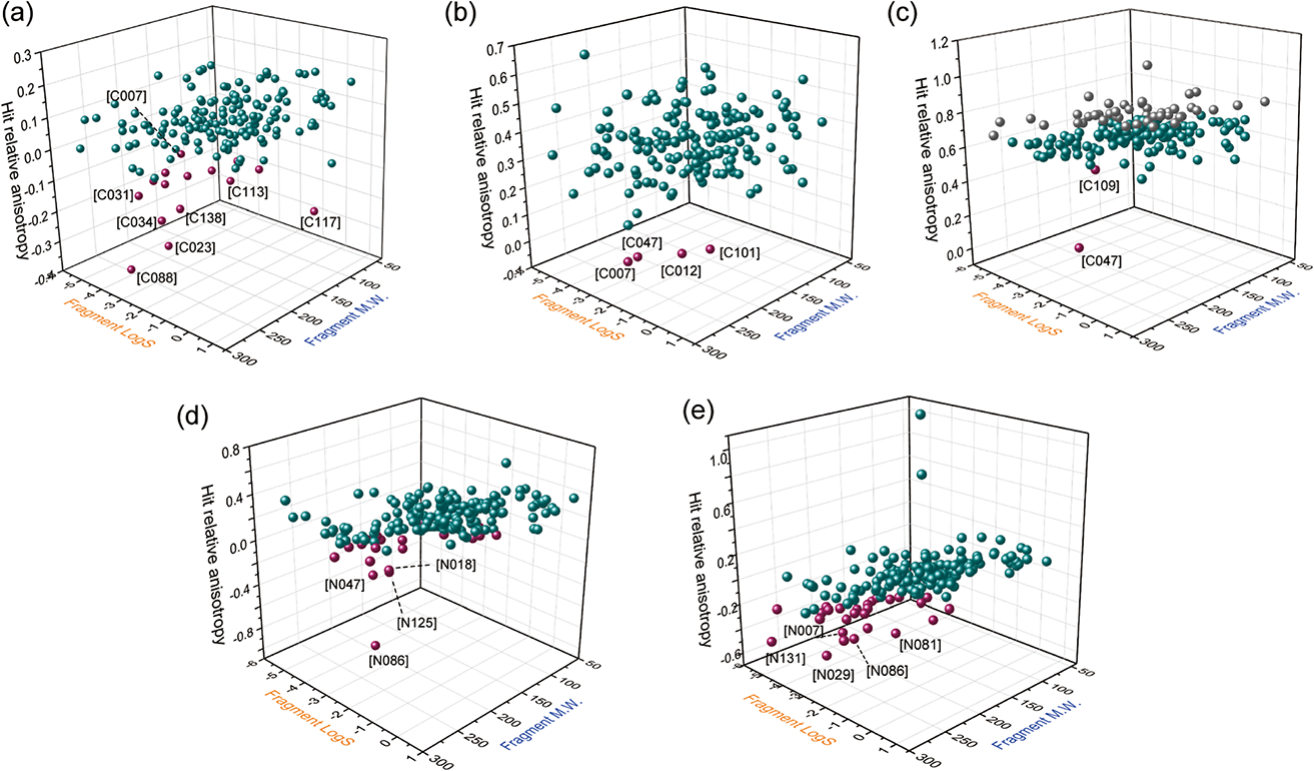
Plots of LogS and molecular weights against relative anisotropy obtained from dynamic hydrazone exchange screening experiments against SHANK1 PDZ based on: (a) C1T-[C001], (b) C2T-[C001], (c) C3T-[C001], (d) N1T-[N001], and (e) N1C3T-[N001].

Overall, the C-terminal dynamic ligation screening (DLS) generated a series of hits, all of which showed superior inhibitory potency in comparison to their precursor acylhydrazone analogues and, in some instances, comparable potency to the original 9-residue peptide. A significant proportion of the hit fragment structures contained a carboxylate group or hydroxyl group, which may offer potential for additional noncovalent contacts with the target protein. The successful application of DLS to the C-terminus of the internal PBM illustrated the compatibility of this strategy for the discovery of SHANK1-PDZ inhibitors.

We next sought to explore the effects of screening for peptide-fragment hybrids using N-terminal hydrazones. Given the promising results from the C-terminal screening with C2T-[C001], the sequence EESTSFQ was selected to prepare the corresponding N-terminal acylhydrazone C2T-[N001] to screen for fragments that might contact the SHANK1-PDZ loop Gly680-Gln700 in proximity to the N-terminus of the PDZ binding motif. In addition, by removing the N-terminal residue Glu (−5), sequences ESTSFQGP and ESTSF were selected to prepare the corresponding N-terminal acylhydrazones N1T-[N001] and N1C3T-[N001], allowing a further assessment of the effects of truncating the C-terminus in the context of the N-terminal screening.

To facilitate N-terminal acylhydrazone functionalization, an extra linker, 1-[2-[(1,1-dimethylethoxy)­carbonyl]­hydrazide], was first prepared, which could be introduced on the N-terminus to yield hydrazide analogues following SPPS using a rink amide resin (Figure S8). Anchor acylhydrazone peptides C2T-[N001], N1T-[N001], and N1C3T-[N001] were generated by reacting with a 10-fold excess of benzaldehyde. Kinetic analyses for aldehyde exchange were carried out in the same manner as for the C-terminal hydrazones (see Figure S4c) and established that ∼24 h was sufficient for equilibration. FA competition assays against the FAM-Ahx-EESTSFQGP-CONH_2_/SHANK1-PDZ interaction were performed to ascertain the inhibitory potency of the N-terminal acylhydrazone peptides (Figure S9). C2T (IC_50_ = 13.4 ± 0.4 μM) was more potent than the N-terminal hybrid C2T-[N001] (IC_50_ = 26.1 ± 2.7 μM). For the N-terminally truncated peptide N1T (IC_50_ = 72.9 ± 8.6 μM), the corresponding N-terminal acylhydrazone N1T-[N001] was nearly 2-fold more potent (IC_50_ = 43.8 ± 4.3 μM). A similar increase in inhibition was observed for the peptide truncated on both sides, N1C3T (IC_50_ = 246.1 ± 59.1 μM), in comparison to the N-terminal acylhydrazone variant N1C3T-[N001] (IC_50_ = 110.2 ± 50.0 μM).

Screening was performed based on C2T-[N001] at a 25 μM concentration with a 5-fold excess of 165 aldehyde fragments; however, no hits were obtained (data not shown). We then screened N1T-[N001] (40 μM) and obtained 20 hits (Figure S5d). Among the 20 hits, the four hits with the lowest relative anisotropy values all contained at least one aromatic ring. N1T-[N018] bears a carboxylate group in its structure, which may compensate for the loss of the N-terminal Glu from the parent ligand. The other three hits all have different alkane substituents on their aromatic rings, suggesting that the peptide-fragment hybrid can make van der Waals contacts with the PDZ domain and/or that solvophobic factors can be important. The final screen was performed with N1C3T-[N001]. Here, we obtained 28 hits (Figure S5e), of which five hits were selected for validation and full dose-response studies (Table 2): N1C3T-[N007] (24.2 ± 2.5 μM), N1C3T-[N029] (34.2 ± 1.8 μM), N1C3T-[N081] (44.7 ± 3.0 μM), N1C3T-[N086] (29.0 ± 1.5 μM), and N1C3T-[N131] (48.9 ± 5.5 μM), providing up to a 10-fold increase in potency compared to the precursor peptide N1C3T (Figure S10). Twelve of the 28 fragments contained an aromatic ring and a carboxylate group, implying that the selected fragment compensates for the loss of these interactions, which would otherwise be made by Glu (−5). Significantly, of the hits with the largest response, only one (N086) was common to the screens carried out with N1T-[N001] and N1C3T-[N001], indicating that the C-terminus has the potential to influence interactions at the N-terminus (see later). As for the C-terminal truncations, we plotted the relative anisotropy values of the hits against their LogS and molecular weight to assess any correlations (Figure 3d–e and Figure S11). There were no obvious preferences for these hits (green and red balls) based on either molecular weight or LogS. As for the hits with the largest response in both screens, most of them showed lower LogS (<−1) and larger molecular weights ranging from 150 to 300.

**Table 2:**
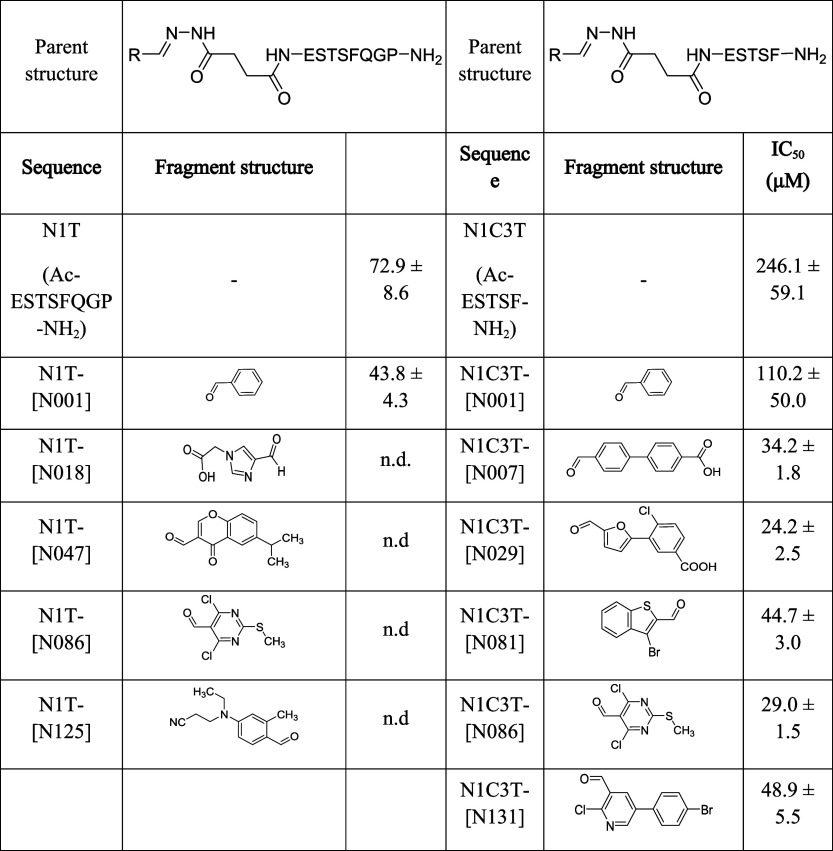
Summary of the Hits’ Structures and Potencies from Screening on N1T-[N001] and N1C3T-[N001]

We next sought to ascertain if C- and N-terminal modifications could be combined to generate ternary hybrids. Given the successful screening outcomes following the removal of the C-terminal Pro and Gly, and that a single Glu could be removed from the N-terminus with favorable screening outcomes, we selected N1C3T and N1C2T as templates; however, orthogonal conjugation of one fragment was therefore required. Prior reports on acylhydrazide bioisosteres have identified amide, ethyl, and amine as effective alternatives (Figure 4a, b).^52^ For synthetic practicality, and in part determined by the availability of appropriately functionalized fragments, we used an amide bond to replace the N-terminal hydrazone moiety. A new linker, Fmoc-5ava–OH, was connected to the N-terminus of the peptide motif ESTSF, which was prepared by SPPS using Fmoc-2-chlorotrityl-hydrazide resin (Figure S12). An acid analog of the fragment [N086] was used to generate an N-terminus functionalized with the fragment: N1C3T-[N086]-NHNH_2_. While N1C3T-[029] showed superior inhibitory activities, the acid analog of the fragment [029] was not commercially available. FA competition assay established that this amide analog, had decreased inhibitory potency in comparison to N1C3T-[N086] (Figure S13a, (Table 3)). While the introduced linker replicates the length of that present in N1C3T-[N086], the positioning of the carbonyl differs and may account for the loss in potency. Based on the screening results with N1C3T-[C001] (see Table 1), fragment [047] was selected to be attached to the C-terminus of N1C3T-[N086]-NHNH_2_. The inhibitory activity of N1C3T-[N086–C047] was further determined by FA competition assay (IC_50_ = 112.3 ± 32.7 μM, Figure S13b, (Table 3)), which is significantly poorer than the binary peptide-fragment hybrid N1C3T-[N086] and implies that attachment of the N-terminal fragment [N086] imposes a constraint on the dynamics or mobility of the peptide such that the C-terminal fragment [C047] cannot bind effectively to SHANK1-PDZ at the same time as the N-terminal fragment (and vice versa) (Figure 4c). Poorer solubility of N1C3T-[N086–C047] may also contribute to the diminished potency.

**Figure 4:**
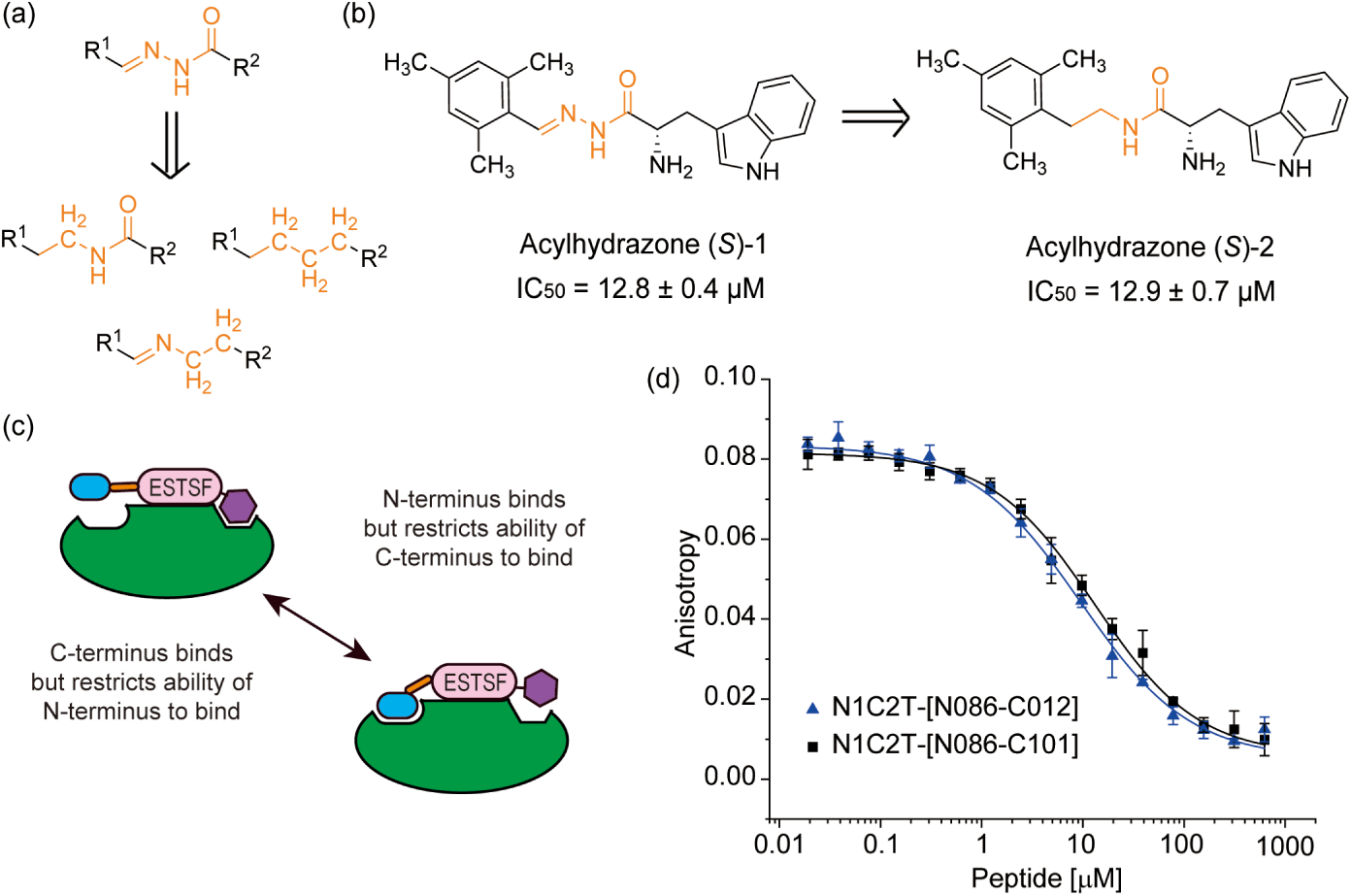
(a) Bioisostere examples of the imine moiety. (b) Illustrated bioisosteric acylhydrazone-based inhibitors of the aspartic protease endothiapepsin described previously.^52^ (c) Cartoon schematic illustrating potential conflict between the N- and C-terminal fragments on the peptide-fragment hybrid, whereby one fragment suppresses the ability of the other to bind. (d) FA competition assays data for N1C2T-[N086–C012] and N1C2T-[N086–C047] (3 μM SHANK1-PDZ, 50 nM FAM-Ahx-EESTSFQGP-NH_2_ 50 mM NH_4_OAc, pH 6.5, using as tracer).

**Table 3:**
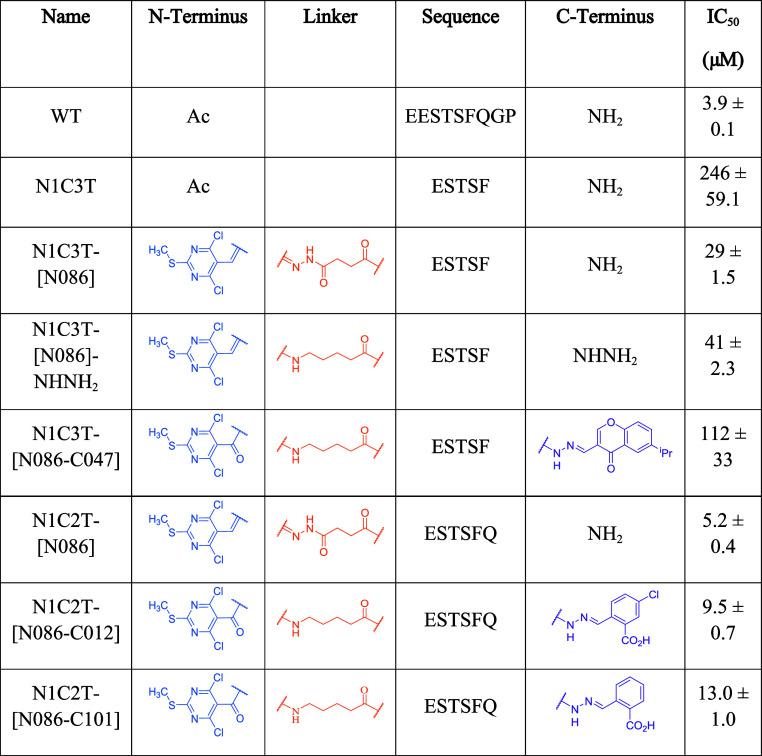
Comparison of the Structures and Inhibitory Activities of N1C3T- and N1C2T-Based Binary or Ternary Hybrids.

We carried out molecular dynamics (MD) simulations for N1C3T-[N086]­hydrazone and N1C3T-[N086]­amide in complex with SHANK-1 PDZ (Figures S14–S17). These show a similar binding mode for both peptides with the protein (Figures S1 and S3). The average structures calculated during the simulation closely resemble those calculated for the energy minimum structures, suggesting that the N-terminal fragment (either as a hydrazone or as an amide) does not explore a large conformational space. MD simulations for N1C3T-[C047] indicate that the peptide-fragment hybrid binds the protein in a similar conformation to that observed in the SHANK1-PDZ/SLiM complex crystal structure (PDB: 8S1R), with the fragment close to the position of the original proline residue. However, a significant departure from the calculated energy minimum structure was observed during the simulation (Figure S18 vs Figure S19). The tendency to drift away from the energy minimum structure was more pronounced in the presence of the additional N-terminal fragment (Figures S20–S22). When compared to the SHANK1-PDZ/N1C3T-[C047] complex, an increased conformational motion proximal to the [C047] C-terminus cap was observed for N1C3T-[N086–C047]. We also assessed the SHANK1-PDZ secondary structure during the simulations. The Gln667-Gly675 loop and the Gly680-Gln700 loop are dynamic^48^ and are located toward the N-terminal and C-terminal peptide binding sites. While these regions were dynamic during the simulations, there were no pronounced differences between them (Figures S23,24). Finally, we carried out ITC analyses on these peptide-fragment hybrids (Table S1, Figures S25–28). N1C3T-[N086] bound with slightly higher affinity (ΔG = −25.5 kJ mol^–1^) than N1C3T-[C047], which bound with slightly higher affinity (ΔG = −24.4 kJ mol^–1^) than N1C3T-[N086–C047] (ΔG = −24 kJ mol^–1^). Although the data should be treated with caution given the higher N value (i.e., binding stoichiometry) for N1C3T-[N086–C047] (see SI), this peptide gave a significantly different thermodynamic signature with a less favorable enthalpic contribution to the binding and a more favorable entropic contribution to the binding (ΔH = −13.1 kJ mol^–1^ TΔ*S*= −10.9 kJ mol^–1^) in comparison to N1C3T-[N086] (ΔH = −31.9 kJ mol^–1^ TΔ*S*= 6.4 kJ mol^–1^) and N1C3T-[C047] (ΔH = −38.3 kJ mol^–1^ TΔ*S*= 13.8 kJ mol^–1^). This may indicate a less ordered or “fuzzy” interaction for N1C3T-[N086–C047], which bears fragment modifications at each terminus. Overall, the simulations support the hypothesis that the interaction of one fragment at one end of the peptide interferes with the ability of the fragment at the other end of the peptide to bind to the protein.

Informed by these results, we extended the C-terminus and carried out studies on N1C2T (ESTSFQ). In the initial screen on C2T, C2T-[C007], C2T-[C012], C2T-[C047], and C2T-[C101] showed comparable inhibitory potencies. Therefore, all four fragments were selected for the C-terminal hydrazone functionalization. We considered it acceptable to use the same N-terminal modification, given that N1C2T and N1C3T share the same N terminus, and fragment [086] was identified in the screen for N1T and N1C3T (see Figure S13c for FA competition data for N1C2T-[N086]). Four ternary hybrids—N1C2T-[N086–C007], N1C2T-[N086–C012], N1C2T-[N086–C047], and N1C2T-[N086–C101]—were generated (Table 3). The ternary peptide-fragment hybrid N1C2T-[N086–C101] had a low micromolar inhibitory activity (IC_50_ = 13.1 ± 1.0 μM). N1C2T-[N086–C007] could not be purified, while N1C2T-[N086–C012] was obtained in ∼89% purity and was similarly potent (IC_50_ = 9.5 ± 0.7 μM), and N1C2T-[N086–C047] had very poor solubility. Both tested compounds contained a free carboxylate group on the C terminus, which may improve solubility and form additional noncovalent interactions with SHANK1-PDZ. Compound N1C2T-[N086–C101] has the lowest MW (1162 Da) and CLogP value (−4.77) as well as the highest LogS value (−6.78), indicating the highest relative aqueous solubility in line with its low micromolar inhibitory activity (Table 3).

## Conclusion

In the present study, we developed peptide-fragment hybrids for a model β-strand-mediated protein–protein interaction that involves the SHANK1-PDZ domain (656–762). To do so, we truncated an internal PDZ binding motif (i.e., lacking the canonical C-terminal carboxylate) and demonstrated that this could be appended with fragments to restore inhibitory potency. We used successive reversible acylhydrazone exchange experiments with aldehyde-appended fragments. C- or N-terminally fragment-capped acylhydrazones with single-digit micromolar IC_50_’s could be identified using this approach. Selected fragments tended to have higher molecular weight and larger negative LogS values, but few common structural/compositional attributes, justifying the use of a target-agnostic approach such as fragment screening. The ability to select C-terminal fragments is significant—the majority of PDZ binding ligands possess a C-terminal carboxylate, which offers less desirable properties from a medicinal chemistry and drug discovery perspective. Being able to select at the C-terminus using an internal PDZ binding motif therefore circumvents this issue and offers access to more of the PDZ domains’ solvent-exposed surface, which might be advantageous in terms of optimizing potency/selectivity. To combine selected fragments required one of the fragments to be linked permanently to the truncated peptide, so as not to interfere with the second fragment conjugation using the remaining acylhydrazide group. To achieve this, we coupled the N-terminal fragment through a simpler amide linkage. Although this was similar in length to the original linker used for the screen, it was not isoatomic; inhibitory potencies were found to be slightly lower, indicating a nontrivial role of the linker during screening. Our initial attempts to combine fragments highlighted that, for certain truncations, N- and C-terminal fragments exhibited negative co-operativity when combined. Our second attempt, using a slightly longer C-terminal truncation, was more successful and yielded low double-digit micromolar inhibitors bearing fragments at the N- and C-termini. Although these compounds were ultimately less potent than some of the single N- or C-terminal fragment modifications, the difference in linker structure means that direct comparison is inappropriate; however, peptidomimetics bearing fragment modifications at both N- and C-termini approached the potencies obtained for individual modifications but had lower molecular weight, suggesting they may be more ligand-efficient. Significantly, a major source of peptide/protein degradation arises due to the action of amino and carboxypeptides, which the fragments at the N- and C-termini would suppress. Thus, our approach offers potential to generate more ligand-efficient ligands that mimic a β-strand. We and others are therefore primed to further develop β-strand mimicry against a broader range of therapeutic targets in due course.

## Supplementary Material



## Notes

Additional data figures; experimental methods and details; and peptide and protein characterization data (PDF)
