# Supplementary material for: Discovery of SHANK1-PDZ Peptide-Fragment Inhibitors Using a Dynamic Ligation Screening Strategy
Source: Biochemistry. 2026 Apr 7;65(8):1202–13. doi: 10.1021/acs.biochem.5c00804 (PMC13104028; doi:10.1021/acs.biochem.5c00804)
Supplement: Supplementary file 1 [file bi5c00804_si_001.pdf]

Supporting Information

Discovery of SHANK1-PDZ Peptide-fragment Inhibitors Using a Dynamic Ligation Screening Strategy

Yue Li[1,2,3], Diana Gimenez,[1] Stuart L. Warriner[1,2], Andrew J. Wilson*[1,2,3]

[1] School of Chemistry, University of Leeds, Woodhouse Lane, Leeds LS2 9JT, UK

[2] Astbury Centre for Structural Molecular Biology, University of Leeds, Woodhouse Lane, Leeds LS2 9JT, UK

[3] School of Chemistry, University of Birmingham, Edgbaston, Birmingham B15 2TT, UK

Correspondence: Andrew J. Wilson, School of Chemistry, University of Birmingham, Edgbaston, Birmingham B15 2TT, UK. Email: a.j.wilson.1@bham.ac.uk

Table of Contents

Supplementary Tables and Figures S3

Protein Characterization S30

Chromatographic conditions for preparative HPLC S31

Chromatographic conditions for analytical HPLC S31

Characterisation Data S32

Aldehyde library compounds S109

Supplementary Tables and Figures

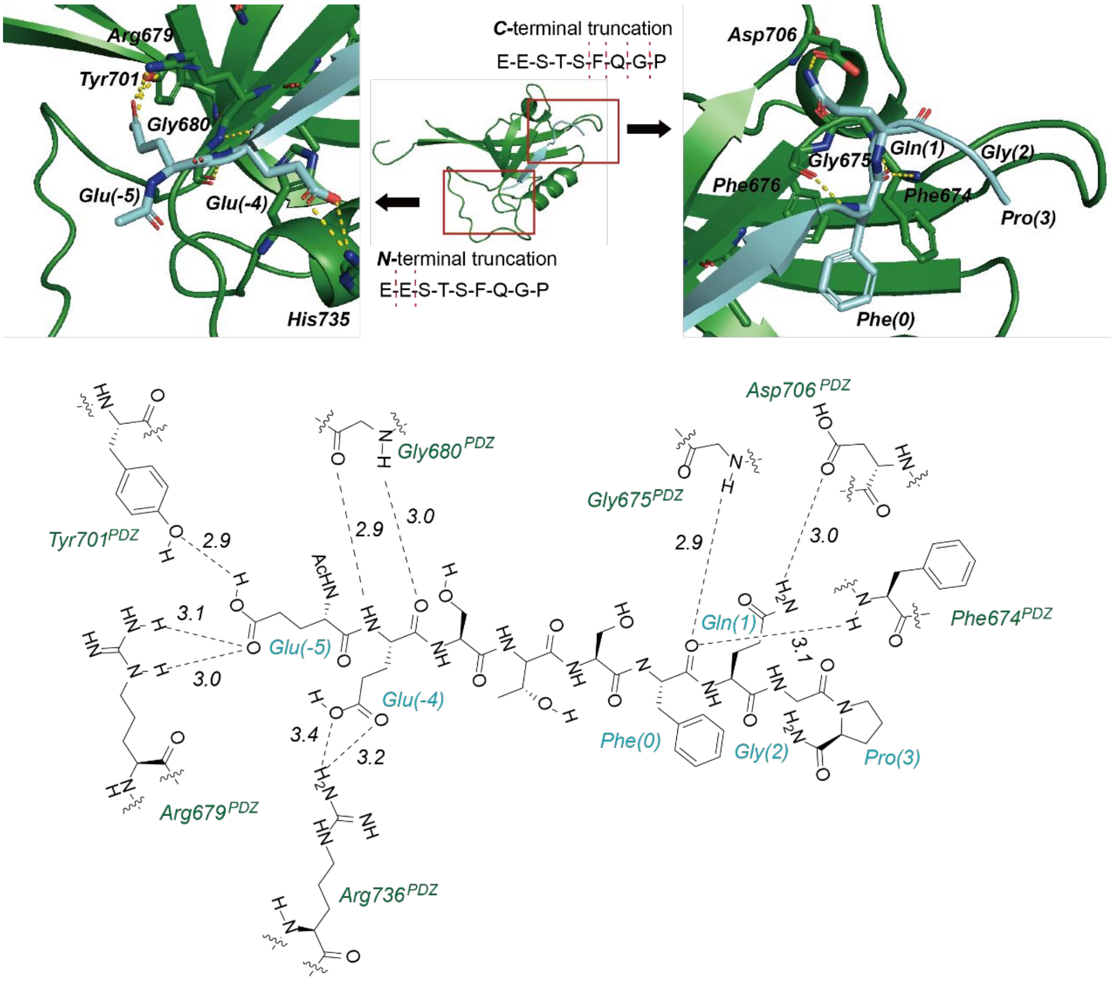

Figure S1. Schematic illustration of the truncated *C*- or *N*- terminal residues and their interactions with the SHANK1-PDZ backbones.

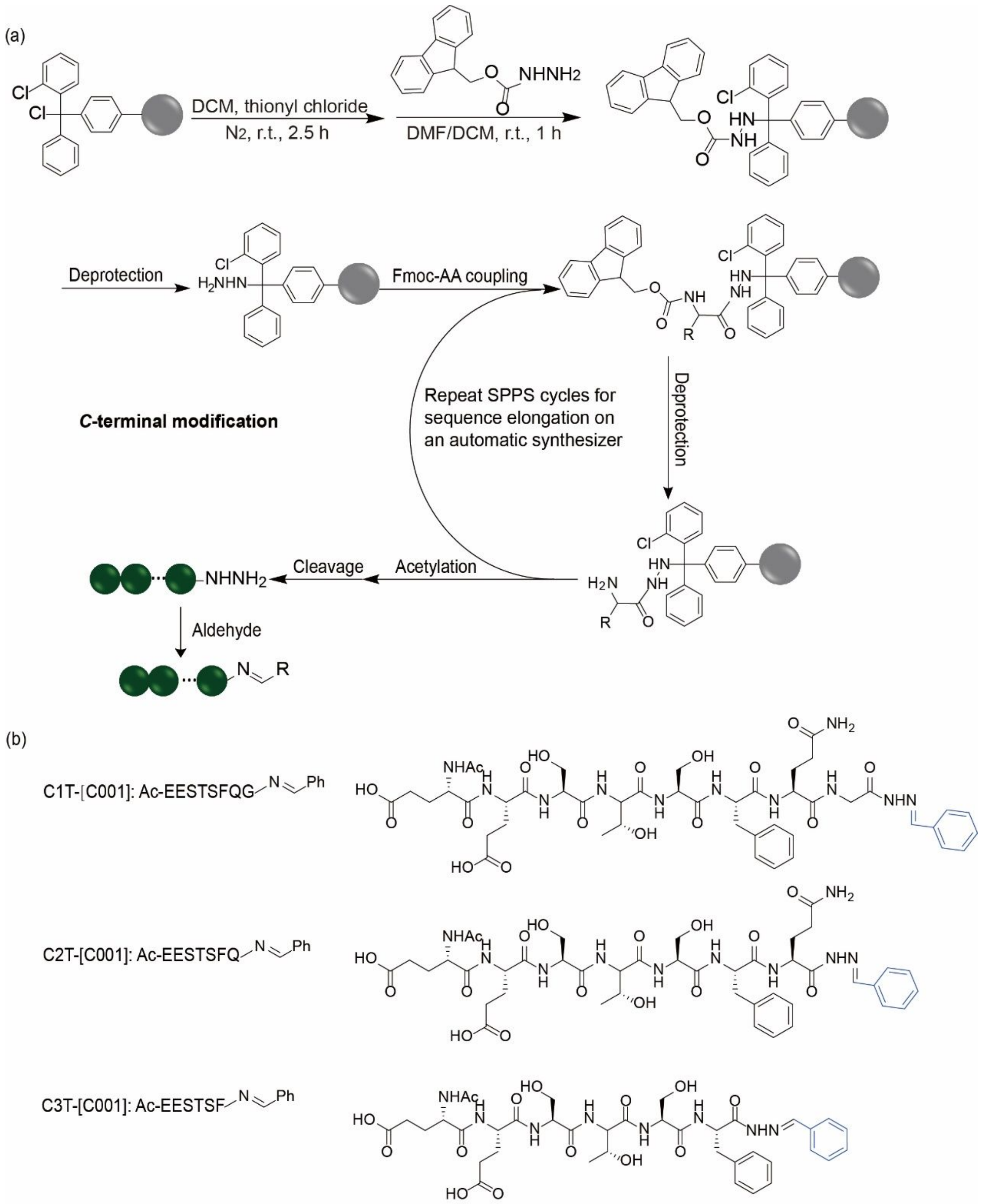

Figure S2. Synthetic route and structures for C-terminal acyl hydrazones: (a) Synthesis of the Fmoc-2-Chlorotrityl hydrazine resin, grey balls represent 2-Chlorotrityl hydrazine resin, followed by SPPS of the C-terminally hydrazone-functionalized peptides, forest green balls represent truncated amino acid sequences; (b) structures of CXT-[C001] series.

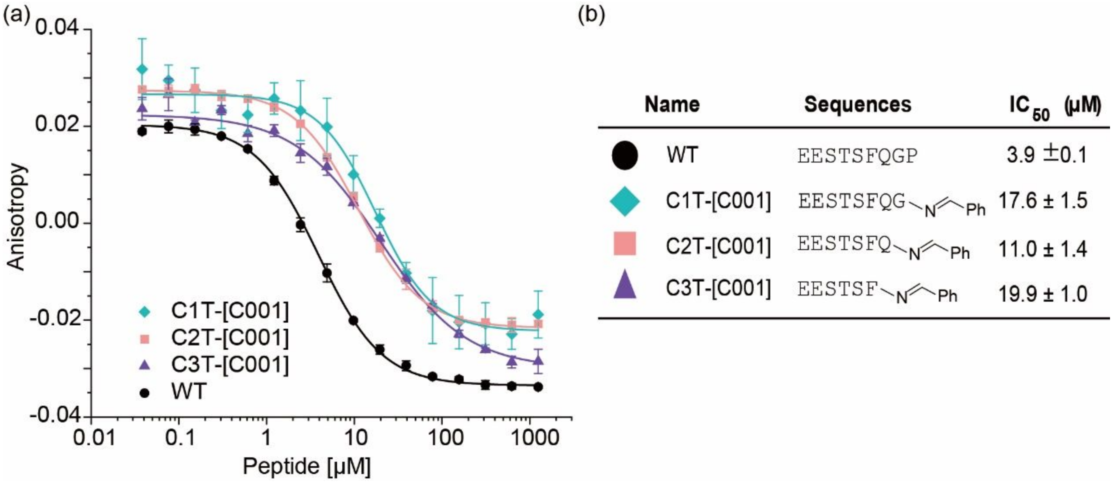

Figure S3. (a) FA competition assays and (b) IC50 values of the anchor acylhydrazone peptides C1T-[C001], C2T-[C001], and C3T-[C001] (3 μM SHANK1-PDZ, 50 nM FAM-Ahx-EESTSFQGP-CONH2, 50 mM NH4OAc, pH 6.5, sequences N-terminally acetylated.

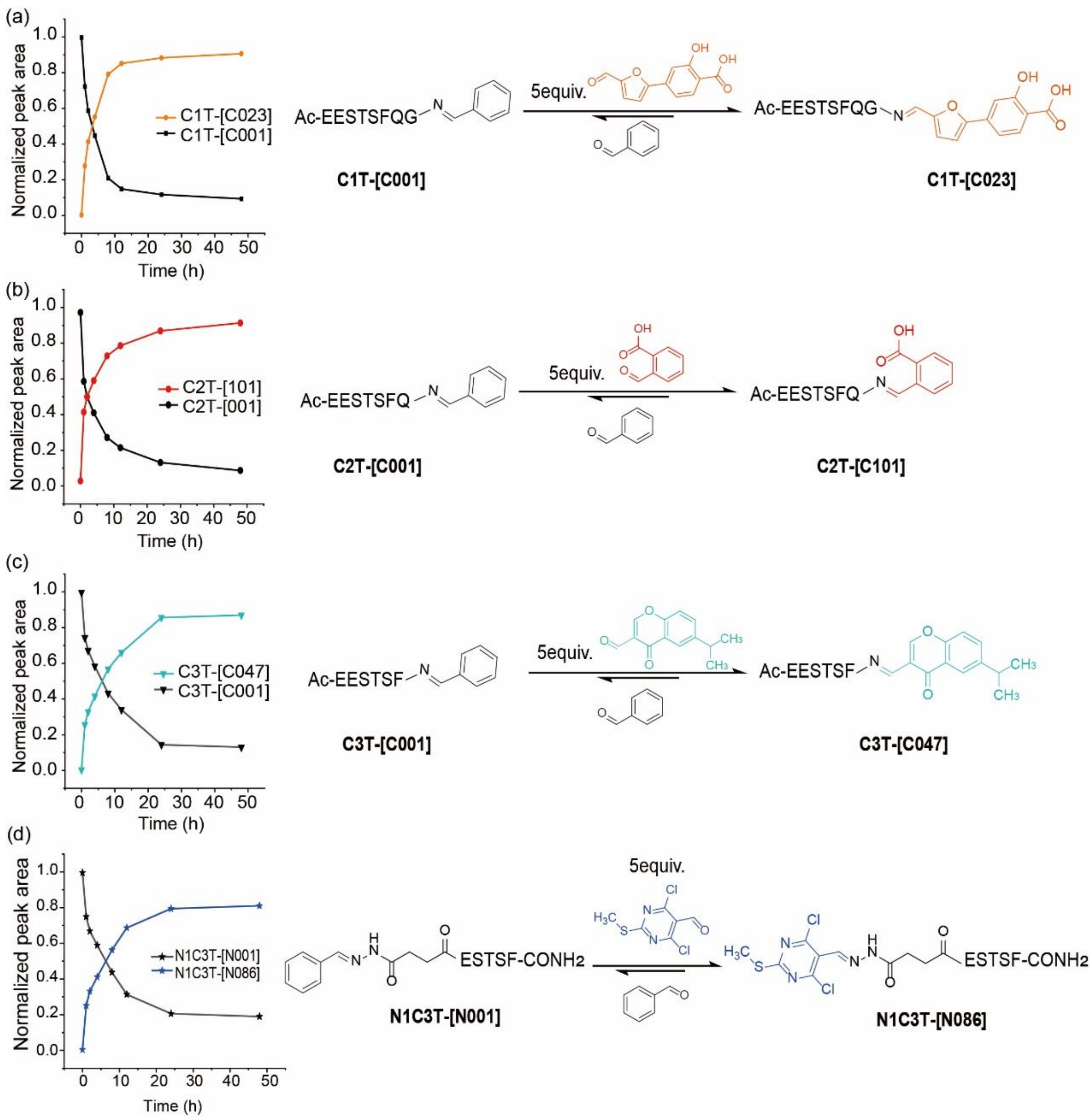

Figure S4. Kinetic analyses of hydrazone exchange experiments for three representative anchor C-terminal acyhydrazone peptides with 5-fold excess of different fragments, (a) C1T-[C001] to C1T-[C023], (b) C2T-[C001] to C2T-[C101], (c) C3T-[C001] to C3T-[C047] and (d) N1C3T-[N001] to N1C3T-[N086] ([acylhydrazone peptide] = 100 μM, 50 mM NH_4OAc, 10 mM aniline, pH 6.5 at ambient temperature).

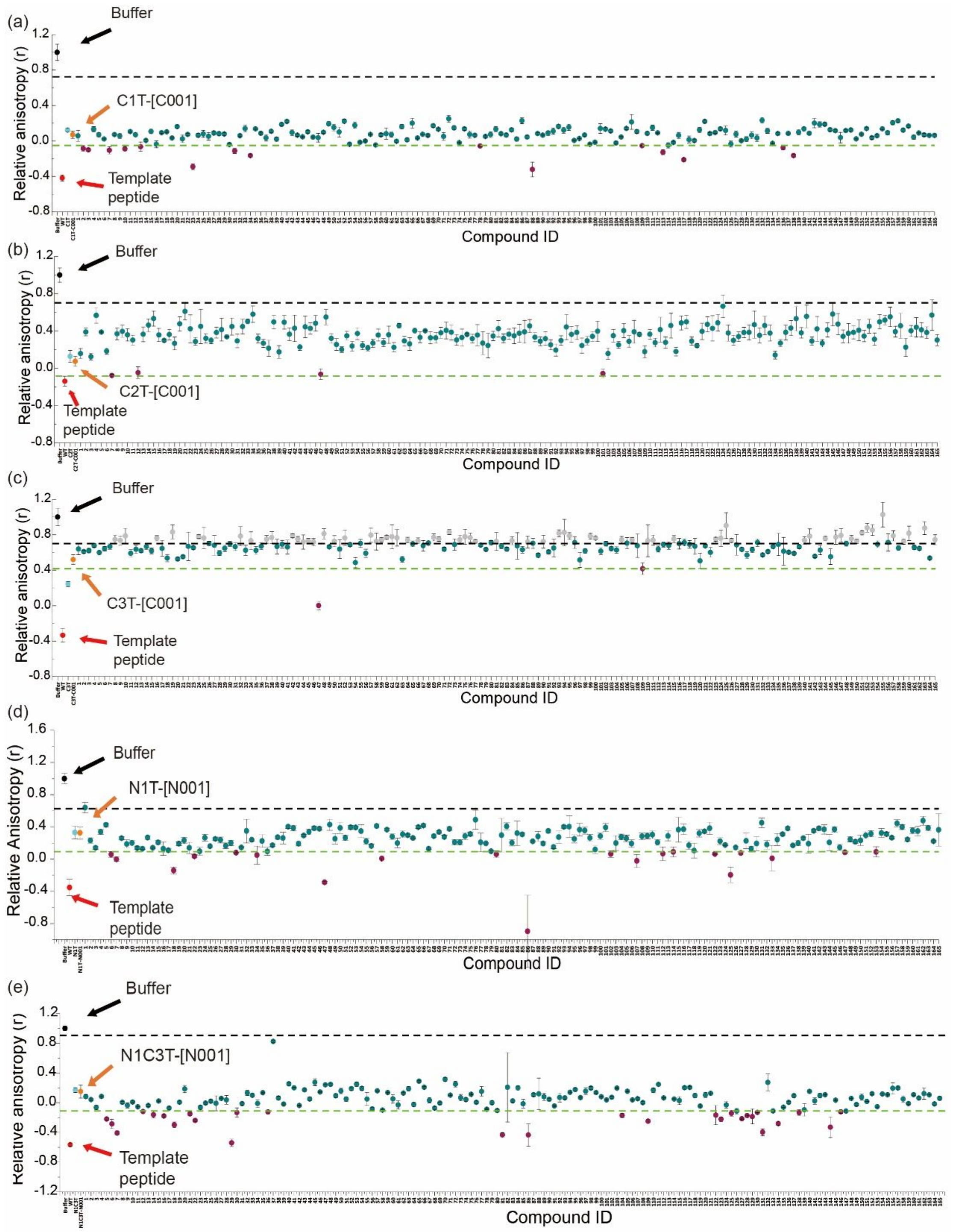

Figure S5. Single concentration screening results based on anchor acylhydrazone peptides (a) C1T-[C001] = 20 µM; (b) C2T-[C001] = 10 µM; (c) C3T-[C001] = 20 µM (d) N1T-[N001] = 40 µM; (e) N1C3T-[N001] = 100 µM; (SHANK1-PDZ = 3 µM and FAM-Ahx-EESTSFQGP-CONH$_2$ = 50 nM, 50 mM NH_4OAc, 10 mM aniline, pH 6.5). The assay included buffer (black), Ac-EESTSFQGP-CONH$_2$ (red), unmodified peptide (cyan), benzyl hydrazone peptide (orange) controls. Each point on the graph represents the relative anisotropy value (r) of a peptide-fragment hybrid. Hit thresholds were defined as (i) buffer r_{rel}-3σ (black dashed line) and (ii) benzyl hydrazone peptide r_{rel}-3σ (green dashed line).

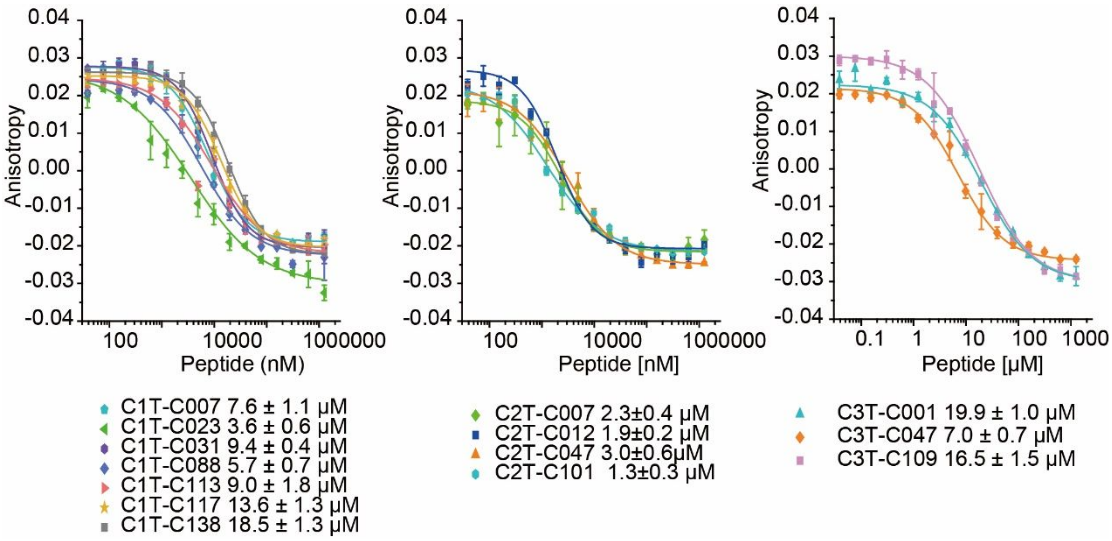

Figure S6. FA competition assays and IC_{50} values of the acylhydrazone peptide series: C1T-[C00X], C2T-[C00X], and C3T-[C00X] (3 µM SHANK1-PDZ, 50 nM FAM-Ahx-EESTSFQGP-$CONH_2$, 50 mM NH_4OAc, pH 6.5, sequences N-terminally acetylated.

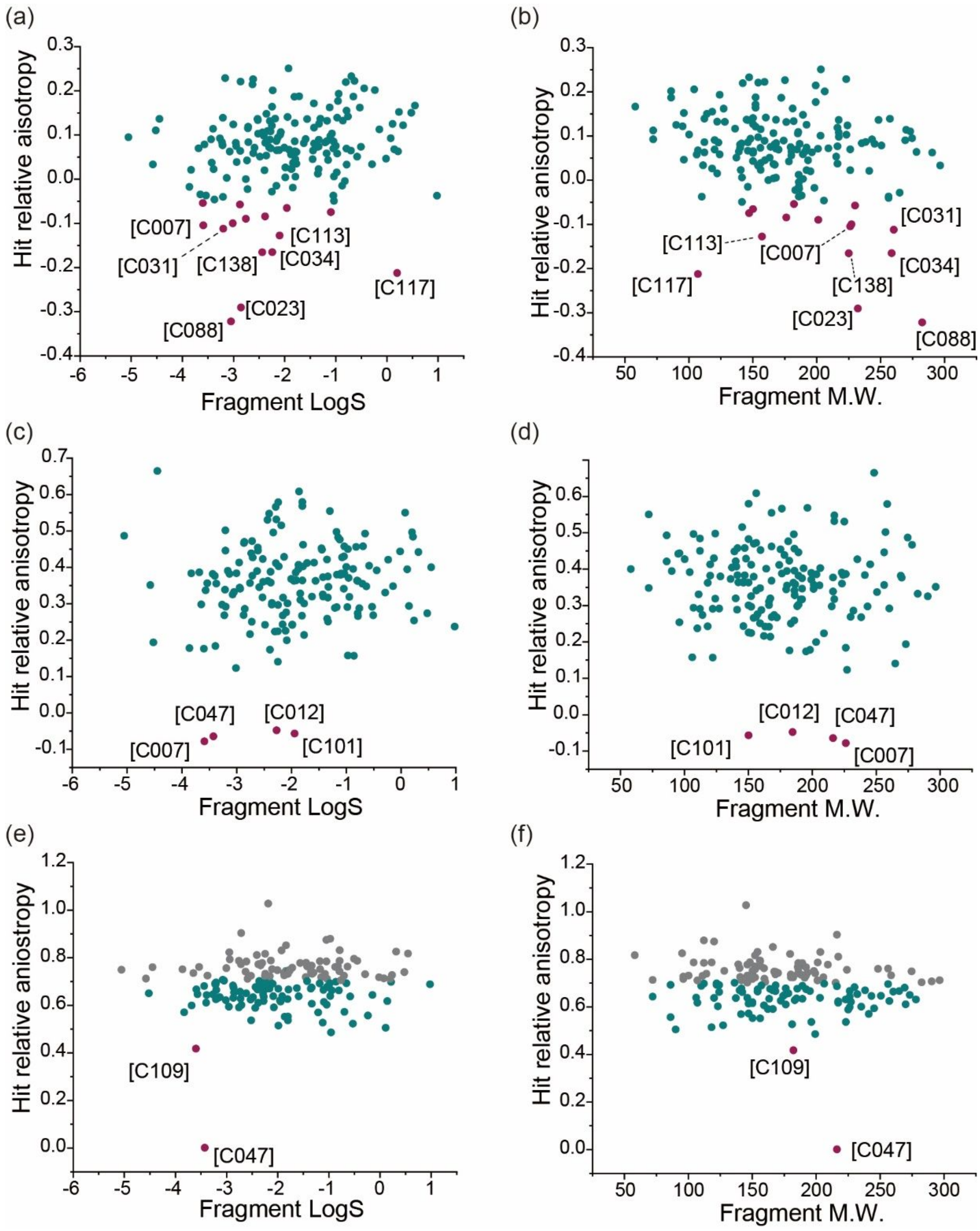

Figure S7. 2D Plots of (a,c,e) LogS and (b,d,f) molecular weights against relative anisotropy obtained from dynamic hydrazone exchange screening experiments against SHANK-1 PDZ based on (a,b) C1T-[C001], (c,d) C2T-[C001], and (e,f) C3T-[C001].

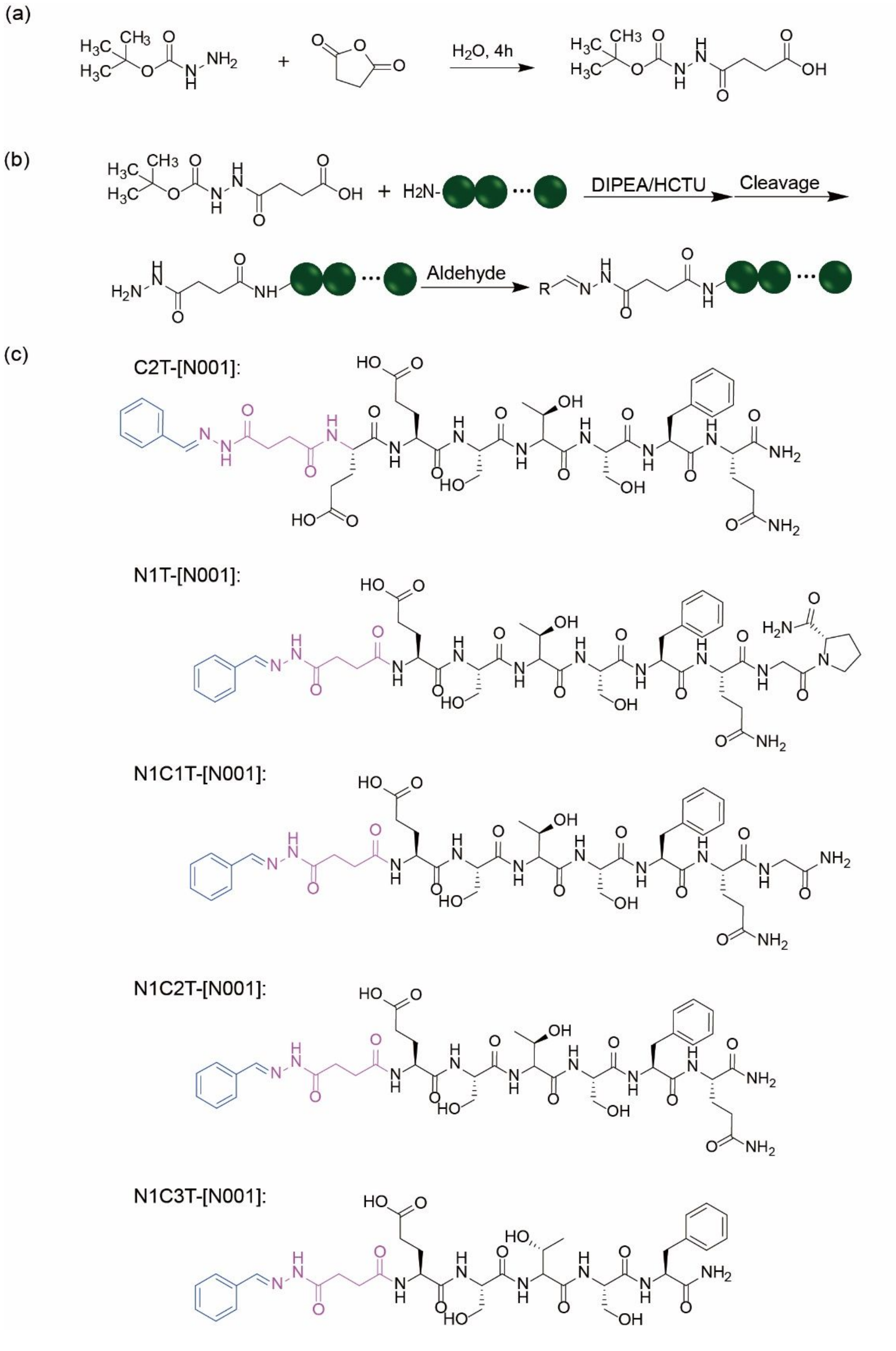

Figure S8. Synthetic routes to *N*-terminally functionalized peptides: (a) Synthesis of the linker 1-[2-[(1,1-dimethylethoxy)carbonyl]-hydrazide; (b) SPPS on rink amide followed by capping with 1-[2-[(1,1-dimethylethoxy)carbonyl]-hydrazide (green balls represent truncated peptide sequences; (c) Structures of the *N*-terminal hydrazones (linker is coloured in pink and the phenyl ring coloured in blue).

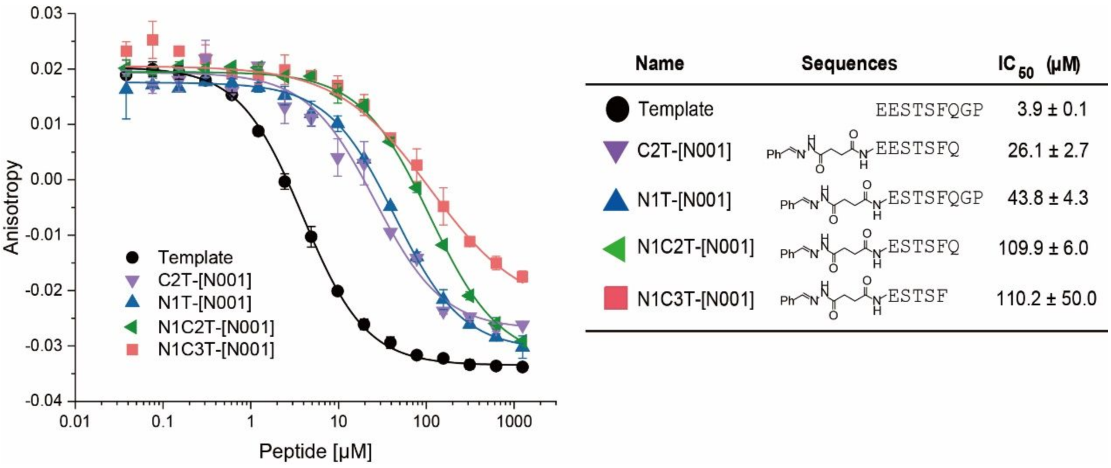

Figure S9. FA competition assays of the N-terminal acylhydrazone peptides C2T-[N001], N1T-[N001], N1C2T-[N001] and N1C3T-[N001] (3 μM SHANK1 PDZ, 50 nM FAM-Ahx-EESTSFQGP-CONH$_2$, 50 mM NH$_4$OAc, pH 6.5. All the sequences were C-terminal amides. The template peptide was also N-terminally acetylated.

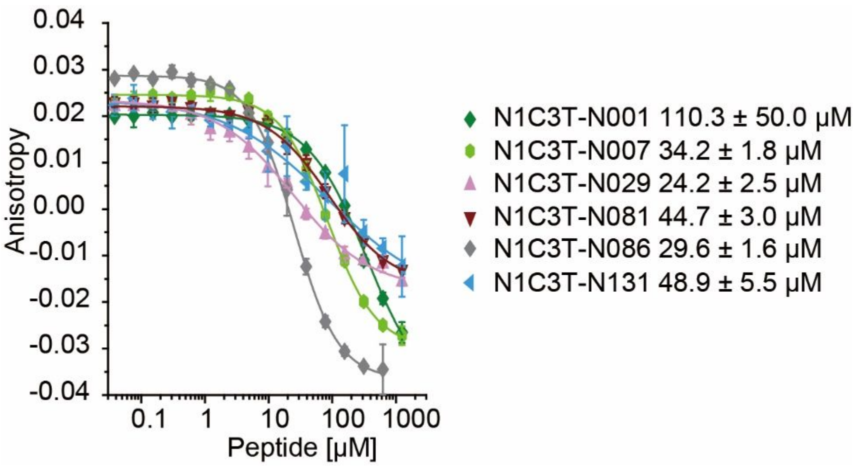

Figure S10. FA competition assays and IC$_{50}$ values of the acylhydrazone peptide series: N1C3T-[N00X] (3 μM SHANK1 PDZ, 50 nM FAM-Ahx-EESTSFQGP-CONH$_2$, 50 mM NH$_4$OAc, pH 6.5. All the sequences were C-terminal amides.

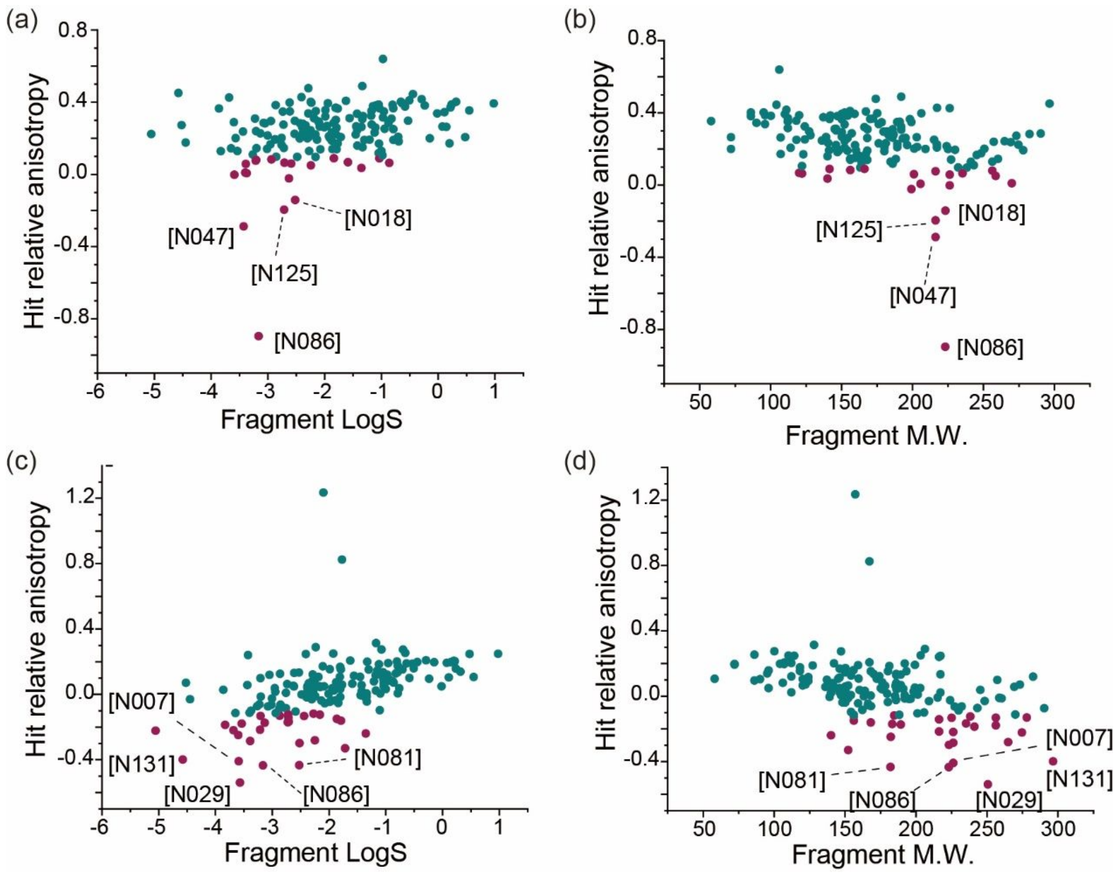

Figure S11. 2D Plots of (a,c) LogS and (b,d) molecular weights against relative anisotropy obtained from dynamic hydrazone exchange screening experiments against SHANK-1 PDZ based on (a,b) N1T-[N001] and (c,d) N1C3T-[N001],

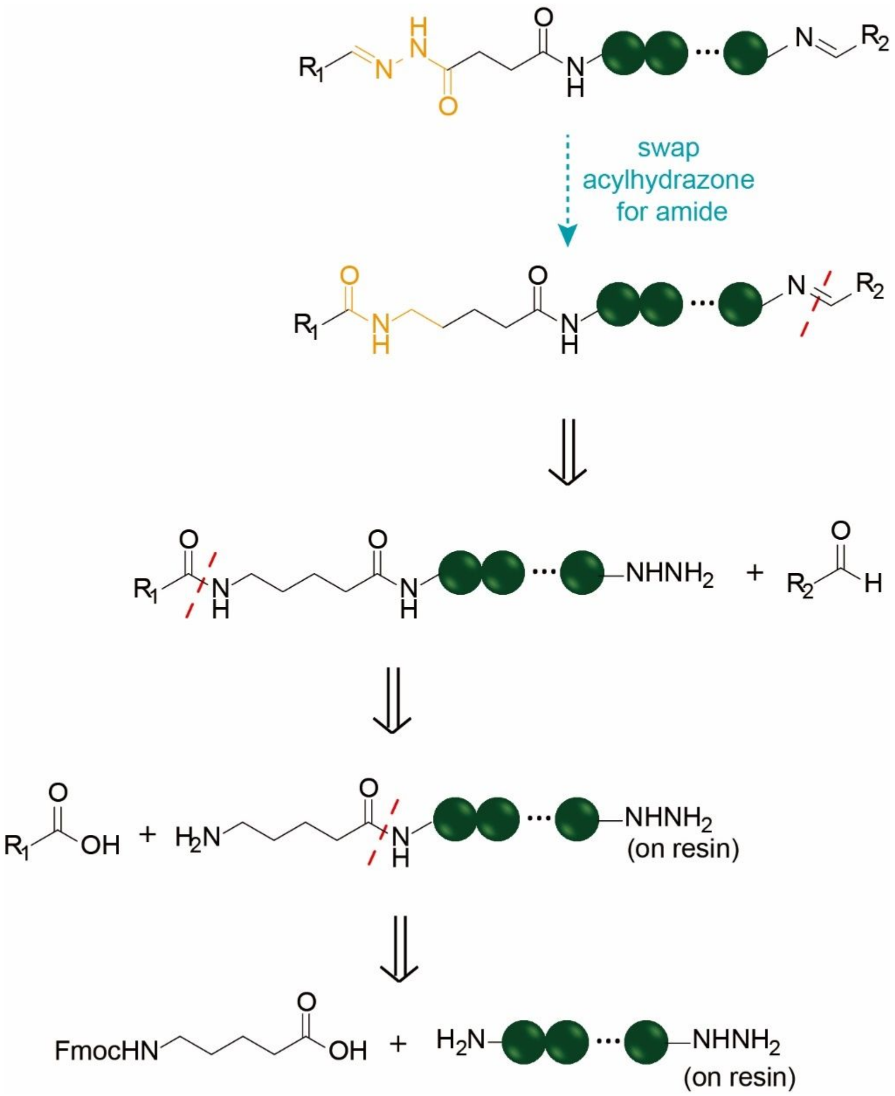

Figure S12. Ligand modifications (blue arrow) and retrosynthetic analysis for the approach to prepare ternary fragment-peptide hybrids bearing two fragments at the N- and C-terminus

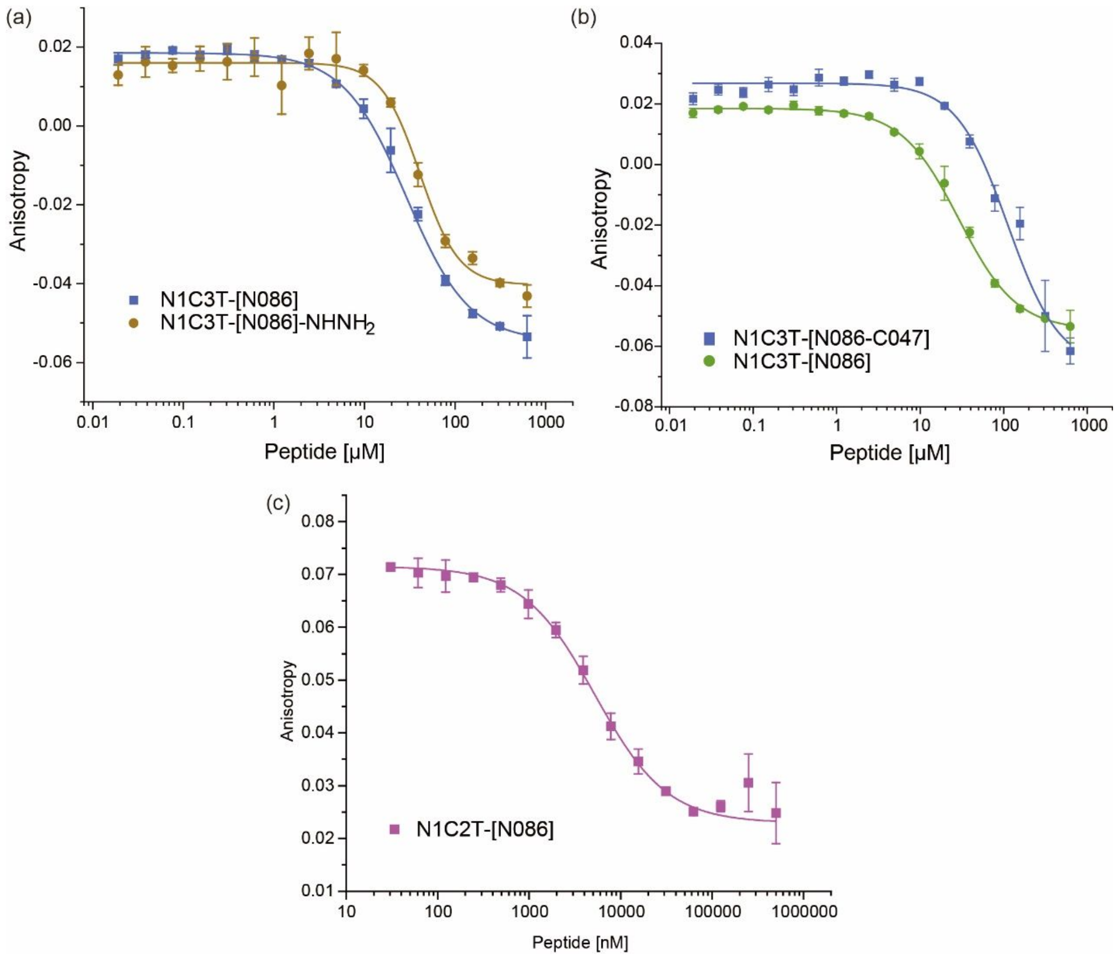

Figure S13. FA competition assays for (a) N1C3T-[N086] and N1C3T-[N086]-$NHNH_2$; (b) N1C3T-[N086-C047] and N1C3T-[N086]; and, N1C2T-[N086] (3 µM SHANK1 PDZ, 50 nM FAM-Ahx-EESTSFQGP-$CONH_2$, 50 mM NH4OAc, pH 6.5, using interaction.

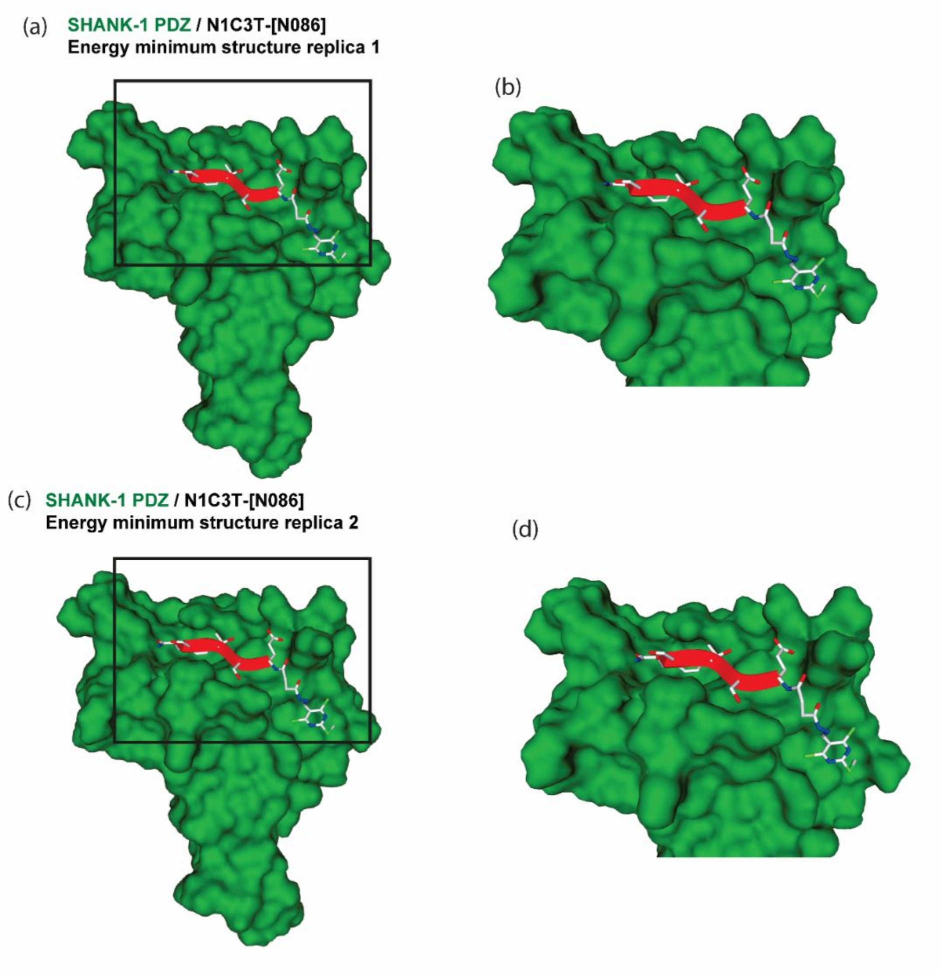

Figure S14. (a-c) Energy minimized structures of SHANK-1 PDZ in complex with N1C3T-[N086] after 125 ns of MD simulation (500 frames). (b-d) Insets showing the accommodation of peptide N1C3T-[N086] in its binding pocket.

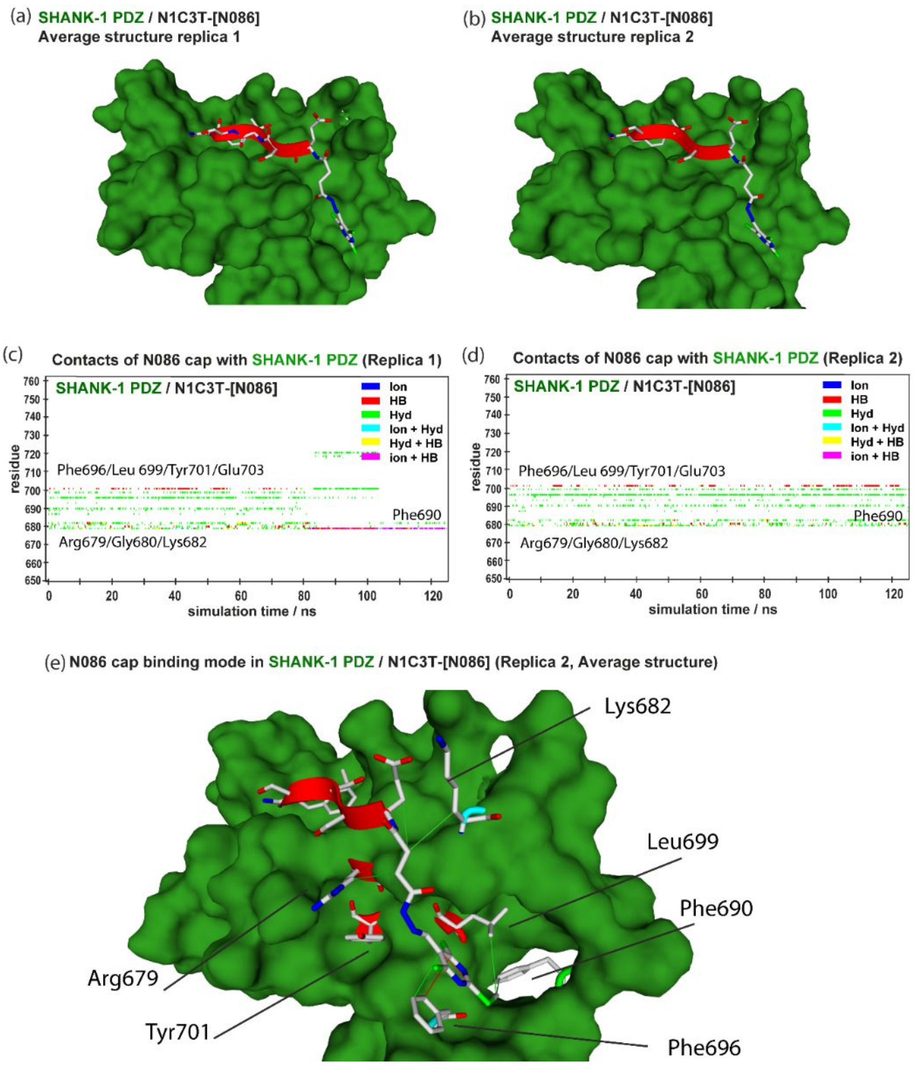

Figure S15. (a-b) Duplicate average structures of SHANK-1 PDZ in complex with N1C3T-[N086] after 125 ns of MD simulation (500 frames); (c-d) Graphical representations of the calculated contacts between the [N086] *N*-terminal cap and SHANK-1 PDZ during the simulation; (e) Inset showing the spine of contacts established during the simulation between *N*-terminal fragment [N086] and SHANK-1 PDZ, with hydrophobic contacts highlighted in green and π-π interactions highlighted in red.

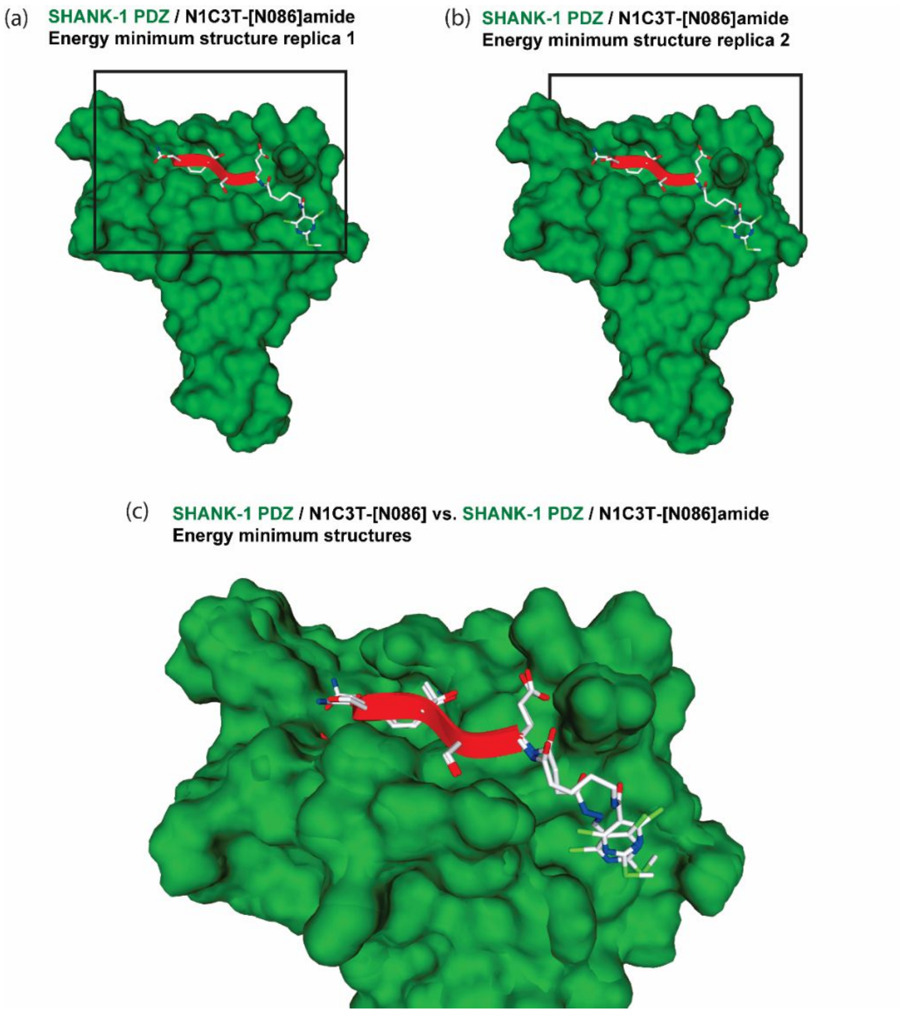

Figure S16. (a-b) Energy minimized structures of SHANK-1 PDZ in complex with N1C3T-[N086]-amide after 125 ns of MD simulation (500 frames); (c) Inset showing the overlaid energy minimum structures of SHANK-1 PDZ when bound to N1C3T-[N086] and N1C3T-[N086] amide.

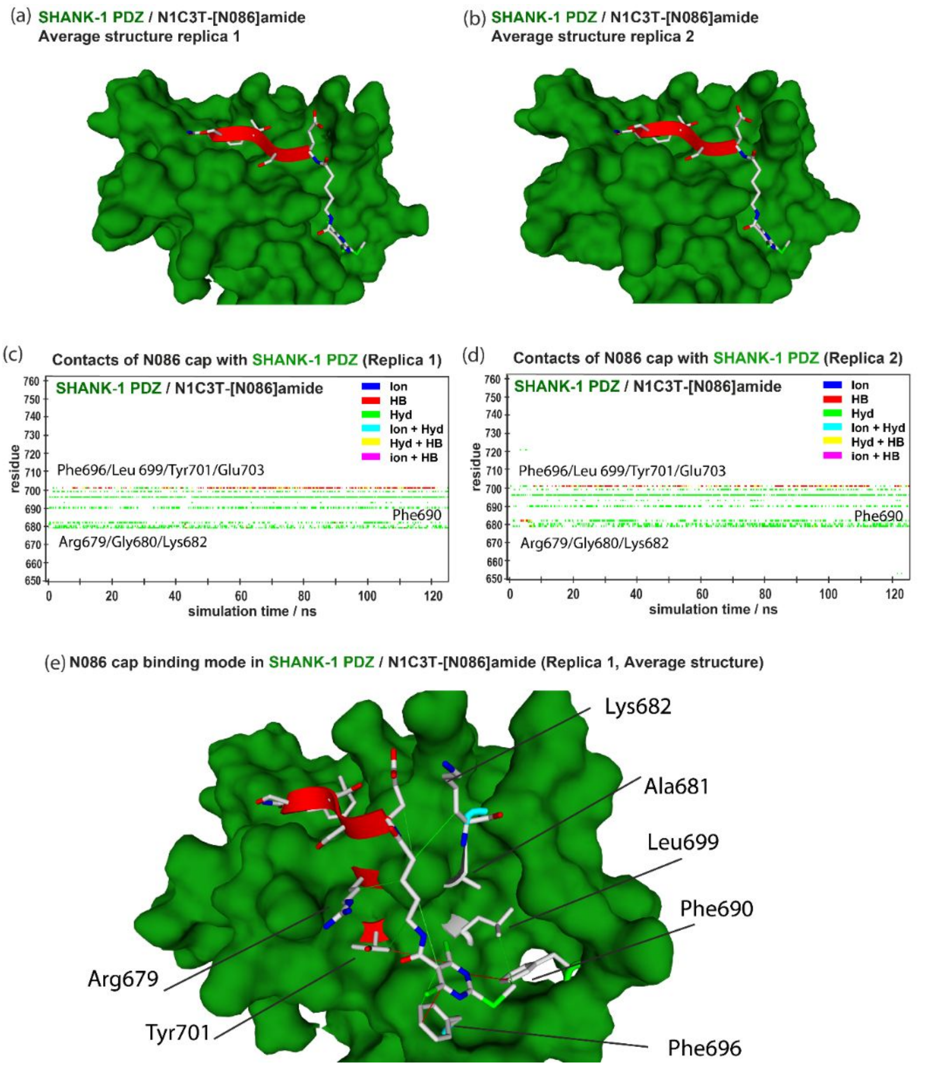

Figure S17. (a-b) Duplicate average structures of SHANK-1 PDZ in complex with N1C3T-[N086]amide after 125 ns of MD simulation (500 frames); (c-d) Graphical representations of the calculated contacts between the [N086]amide *N*-terminal fragment and SHANK-1 PDZ during the simulation. (e) Inset showing the spine of contacts established during the simulation period between *N*-terminus fragment [N086] and the SHANK-1 PDZ protein, with hydrophobic contacts highlighted in green and π-π interactions highlighted in red.

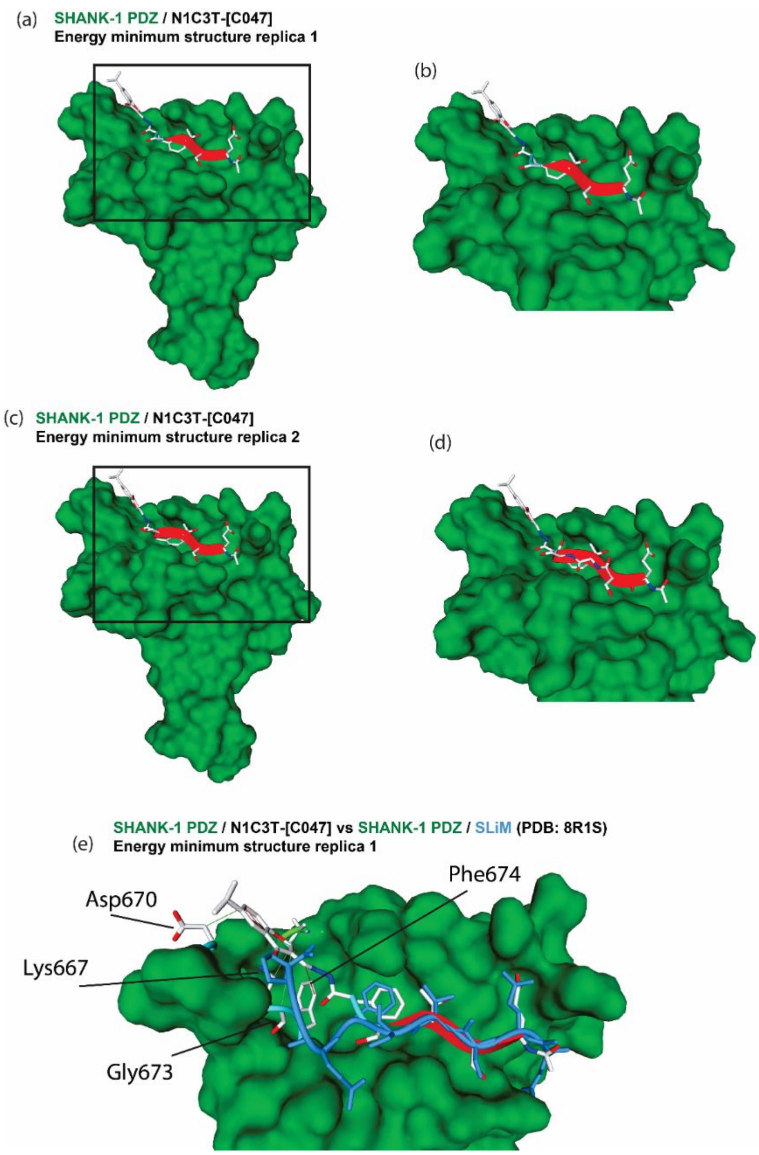

Figure S18. (a-c) Energy minimized structures of SHANK-1 PDZ in complex with N1C3T-[C047] after 125 ns of MD simulation (500 frames); (b-d) Insets showing the accommodation of the peptide N1C3T-[C047] in its binding pocket. (e) Inset showing the contacts between the fragment [C047] and the SHANK-1 PDZ protein in the energy minimum structure, with hydrophobic interactions highlighted in green, π-π interactions highlighted in red and cation-π interaction in red. In blue, the SHANK-1 PDZ/SLiM crystal structure is shown superimposed (PDB: 8S1R).

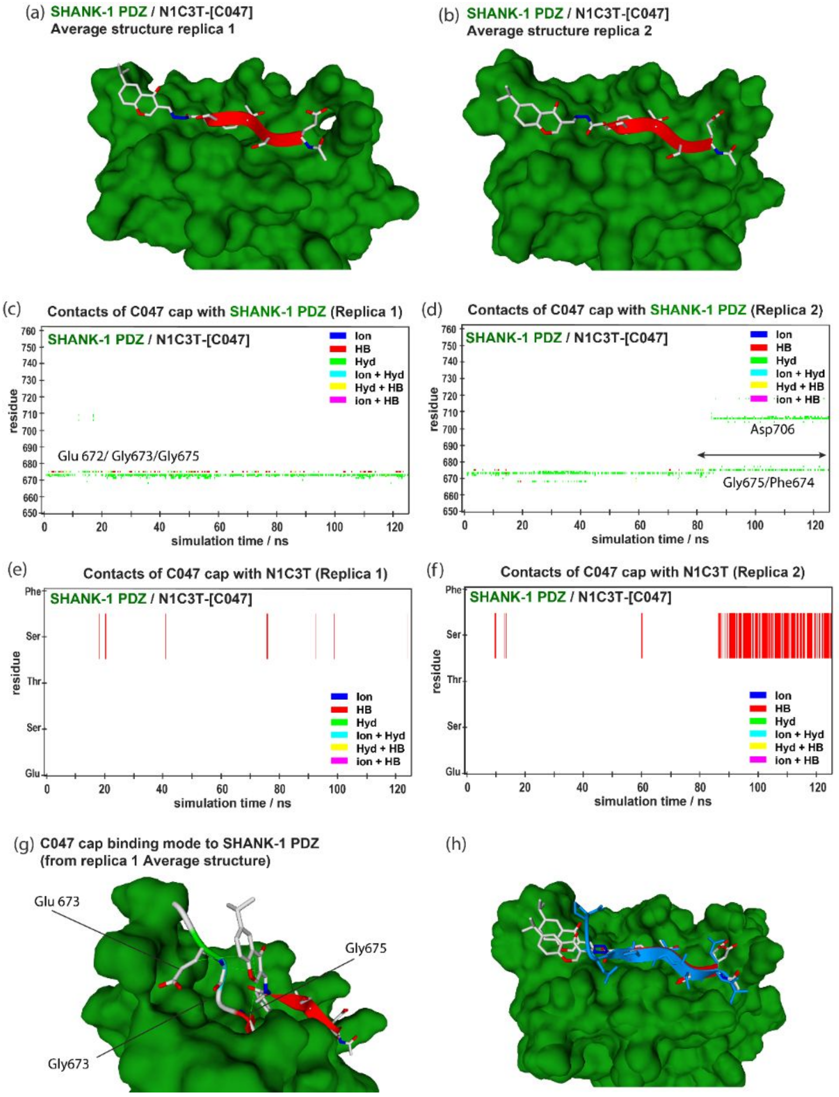

Figure S19. (a-b) Duplicate average structures of SHANK-1 PDZ in complex with N1C3T-[C047] after 125 ns of MD simulation (500 frames); (c-d) Graphical representations of the calculated contacts between the [C047] *C*-terminal fragment and SHANK-1 PDZ during the simulation; (e-f) Graphical representations of the calculated contacts between the [C047] *C*-terminal fragment and the peptide backbone during the simulation; (g) Inset showing contacts between the fragment [C047] and the SHANK-1 PDZ protein in the average structure, with hydrophobic contacts highlighted in green and π-π interactions highlighted in red. (h) Average structures superimposed on the original SHANK-1 PDZ/SLiM complex crystal structure (PDB: 8S1R).

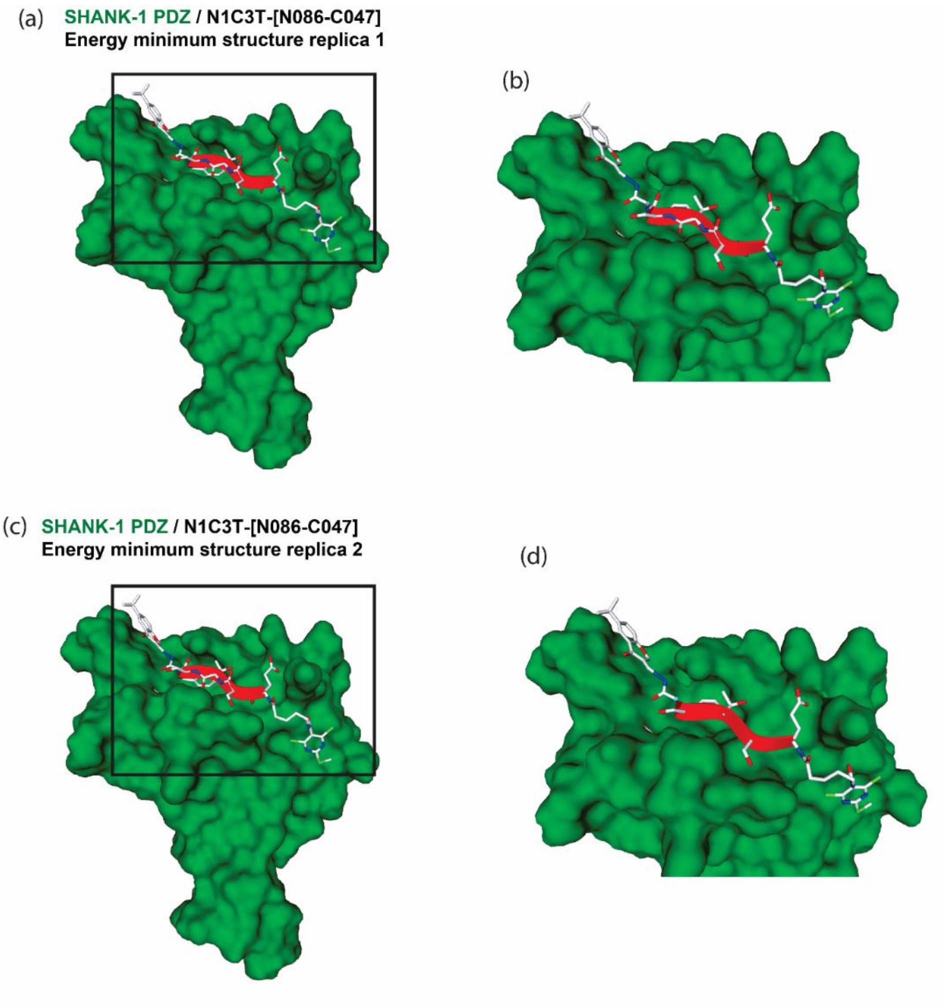

Figure S20. (a-c) Energy minimized structures of SHANK-1 PDZ in complex with fully modified N1C3T-[N086-C047] after 125 ns of MD simulation (500 frames); (b-d) Insets showing the accommodation of the peptide N1C3T-[N086-C047] in its binding pocket.

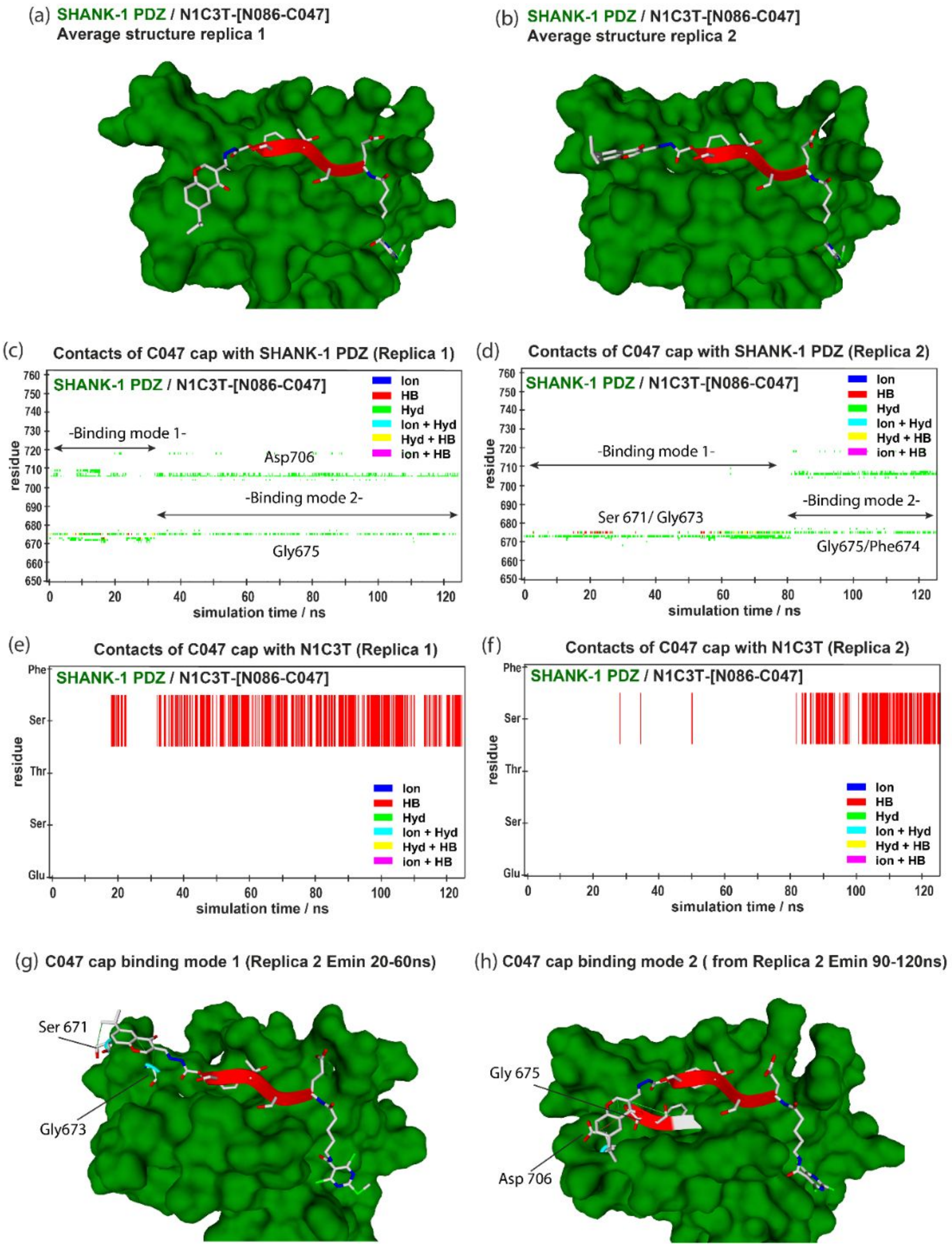

Figure S21. (a-b) Duplicate average structures of SHANK-1 PDZ in complex with N1C3T-[N086-C047] after 125 ns of MD simulation (500 frames); (c-d) Graphical representations of the calculated contacts between the [C047] *C*-terminal fragment and SHANK-1 PDZ during the simulation; (e-f) Graphical representations of the calculated contacts between the [C047] *C*-terminal fragment and the peptide backbone during the simulation; (g) Inset showing contacts between fragment [C047] and SHANK-1 PDZ in the first binding mode. (g) Inset showing contacts between the fragment [C047] and SHANK-1 PDZ in the alternative second binding mode. hydrophobic contacts are highlighted in green and π-π interactions in red.

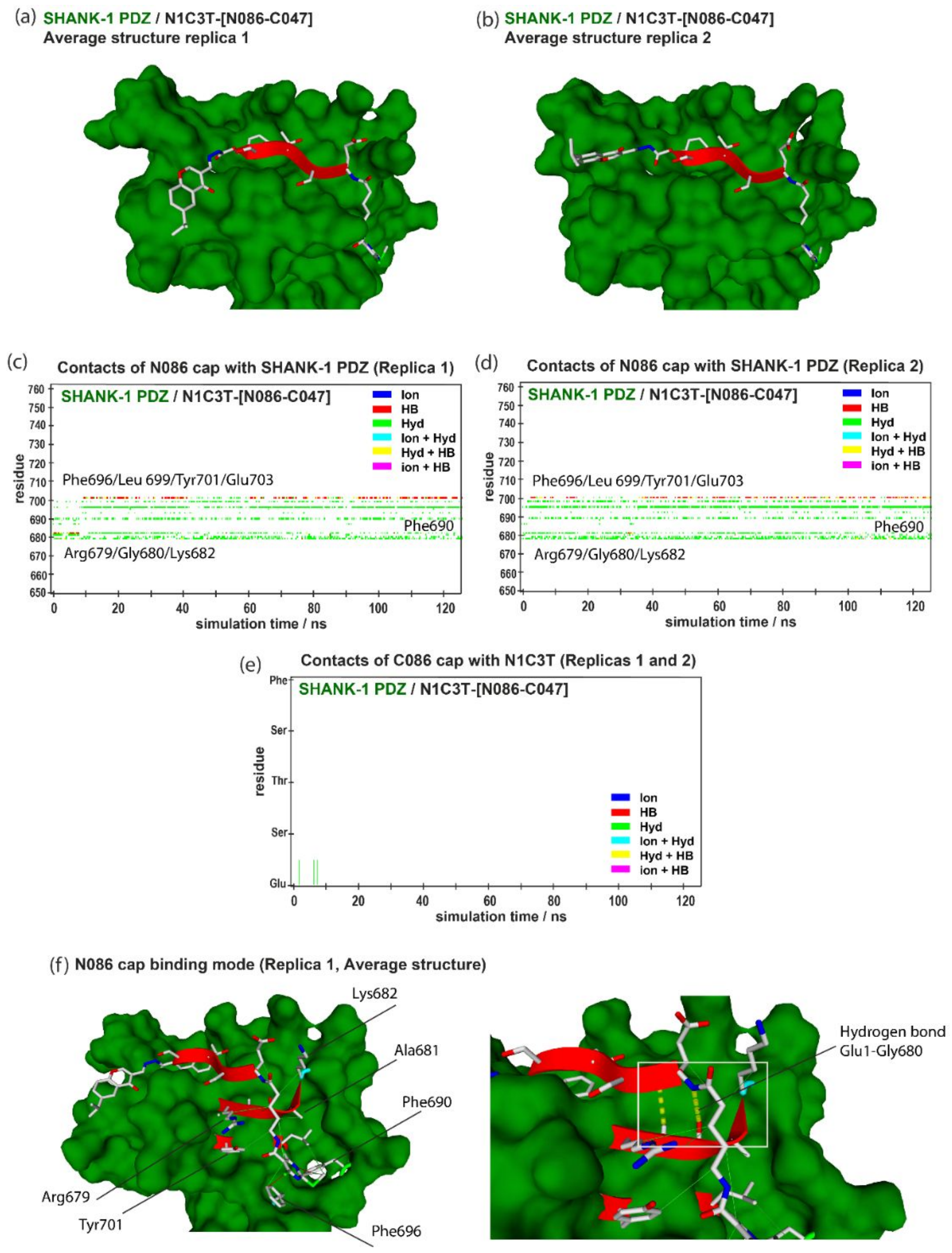

Figure S22. (a-b) Duplicate average structures of SHANK-1 PDZ in complex with N1C3T-[N086-C047] after 125 ns of MD simulation (500 frames); (c-d) Graphical representations of the calculated contacts between the [N086] *N*-terminal fragment and SHANK-1 PDZ during the simulation period; (e) Graphical representations of the calculated contacts between the [N086] *N*-terminal fragment and the peptide backbone during the simulation period; (f-g) Insets showing the spine of contacts established during the simulation period between *N*-terminal fragment [N086] and the SHANK-1 PDZ protein, with hydrophobic interactions highlighted in green and aromatic π-π interactions highlighted in red.

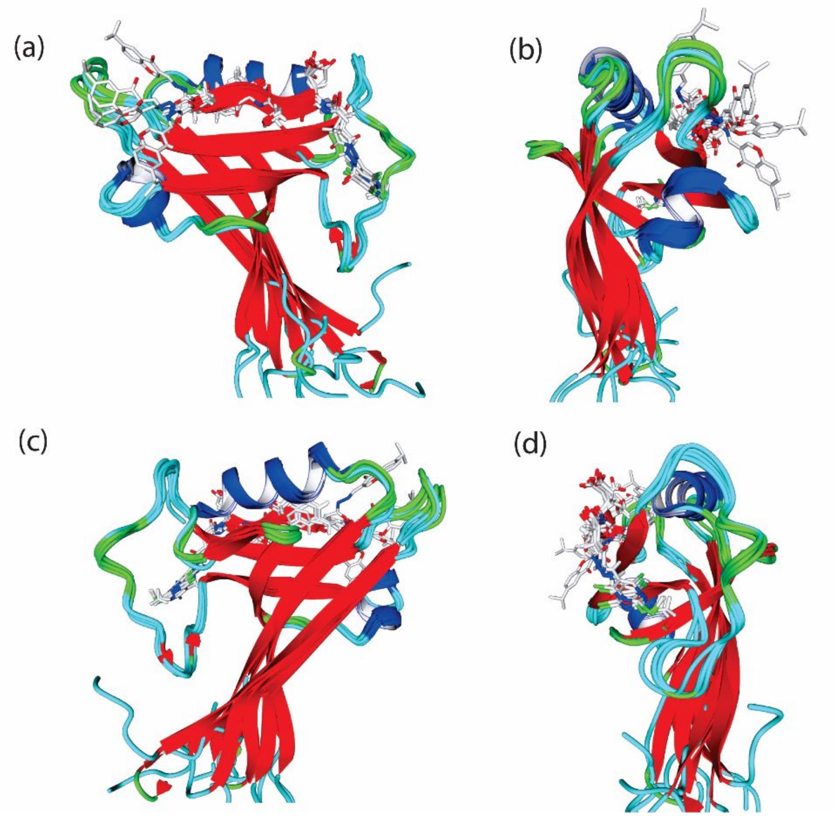

Figure S23. Superimposed calculated average structures of SHANK-1 PDZ in complex with N1C3T-[C047], N1C3T-[N086] and N1C3T-[N086-C047] highlighting the SHANK-1 PDZ secondary structure. (a) Front view, (b) left-hand side view, (c) back and (d) right-hand side view. In all figures, helical regions are highlighted in blue, sheet domains in red, turn motifs in green and coil regions are shown in cyan.

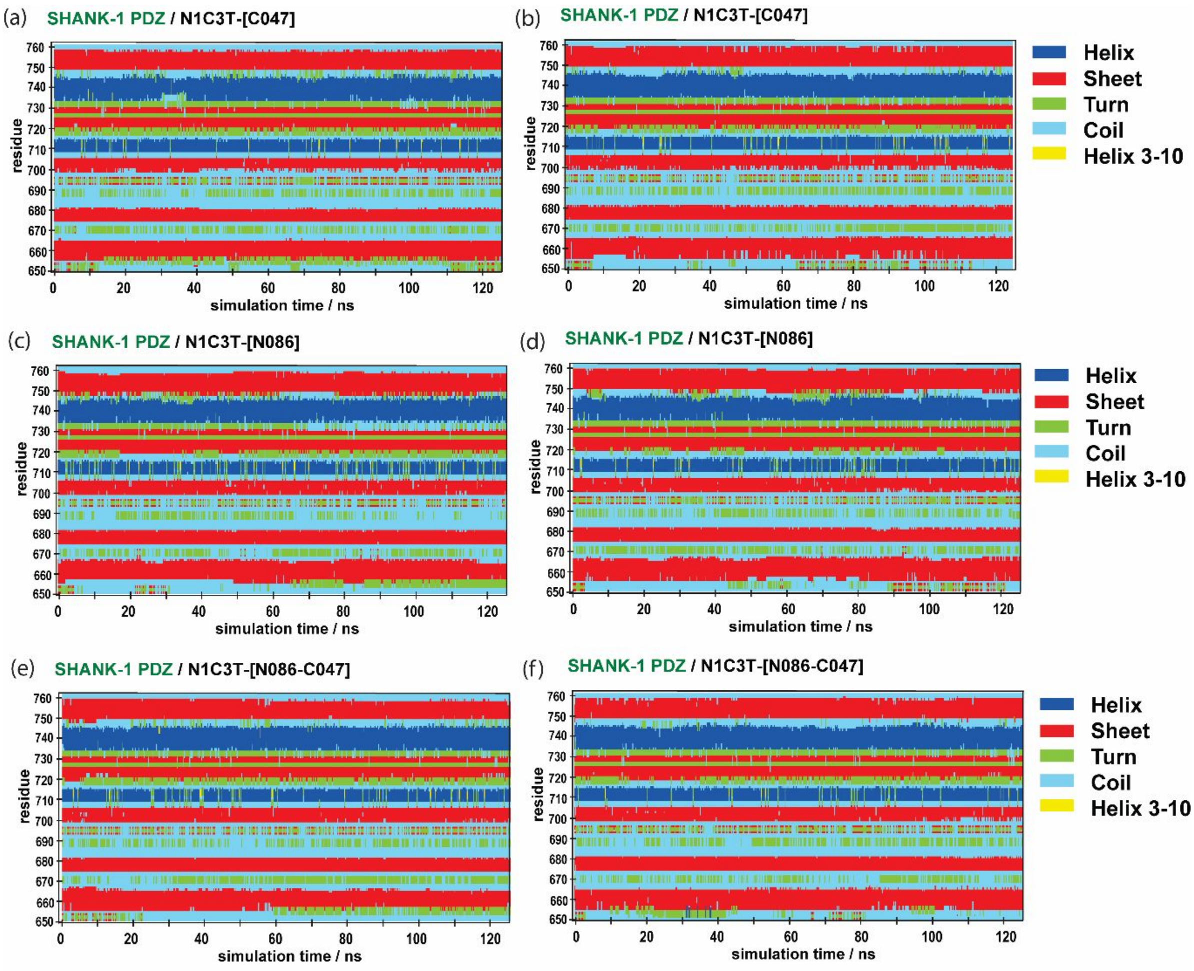

Figure S24. Graphical representations of the SHANK-1 PDZ *per residue* secondary structure during the simulation period with peptide N1C3T-[C047] (a-b), N1C3T-[N086] (c-d) and N1C3T-[N086-C047] (e-f).

		K_d (μM)	ΔG (kJ mol^{-1})	ΔH (kJ mol^{-1})	TΔS (kJ mol^{-1})	*N*
SHANK-1 PDZ + N1C3T-[N086]	Run1	35.5	-25.4	-32.6	7.2	0.8
	Run2	32.4	-25.6	-31.3	5.7	0.9
	**Average**	34.0	-25.5	-31.9	6.4	0.9
SHANK-1 PDZ + N1C3T-[C047]	Run1	47.9	-24.6	-34.9	10.2	1.1
	Run2	56.8	-24.2	-41.7	17.5	0.7
	**Average**	52.4	-24.4	-38.3	13.8	0.9
SHANK-1 PDZ + N1C3T-[N086-C047]	Run1	70.5	-23.7	-15.2	-8.5	2.4
	Run2	56.4	-24.3	-11.1	-13.2	3.1
	**Average**	63.5	-24.0	-13.1	-10.9	2.8

Table S1. ITC analysis and thermodynamic parameters for selected peptides binding to SHANK-1 PDZ. K_d values, ΔG°, ΔH°, and -TΔS° given as determined by isothermal titration of the protein (25 μM in the cell) with the corresponding peptides (in syringe) in 25 mM Tris, 150 mM NaCl; pH= 7.5 at 25 °C.

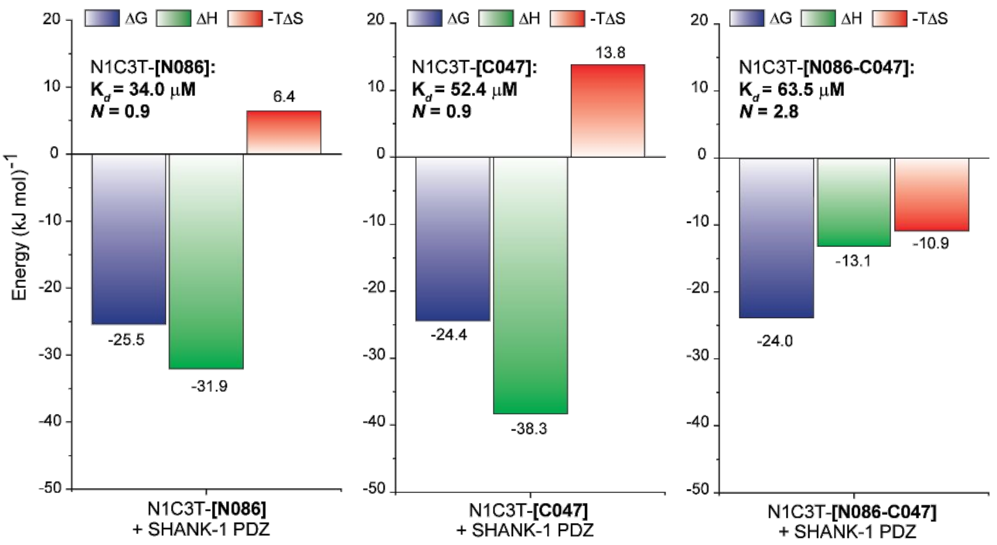

Figure S25. ITC thermodynamic signatures for selected peptides binding to SHANK-1 PDZ. Data shown is the average of two independent titrations of the protein in 25 mM Tris, 150 mM NaCl; pH = 7.5 at 25 °C.

N1C3T-[N086] titration into SHANK-1 PDZ

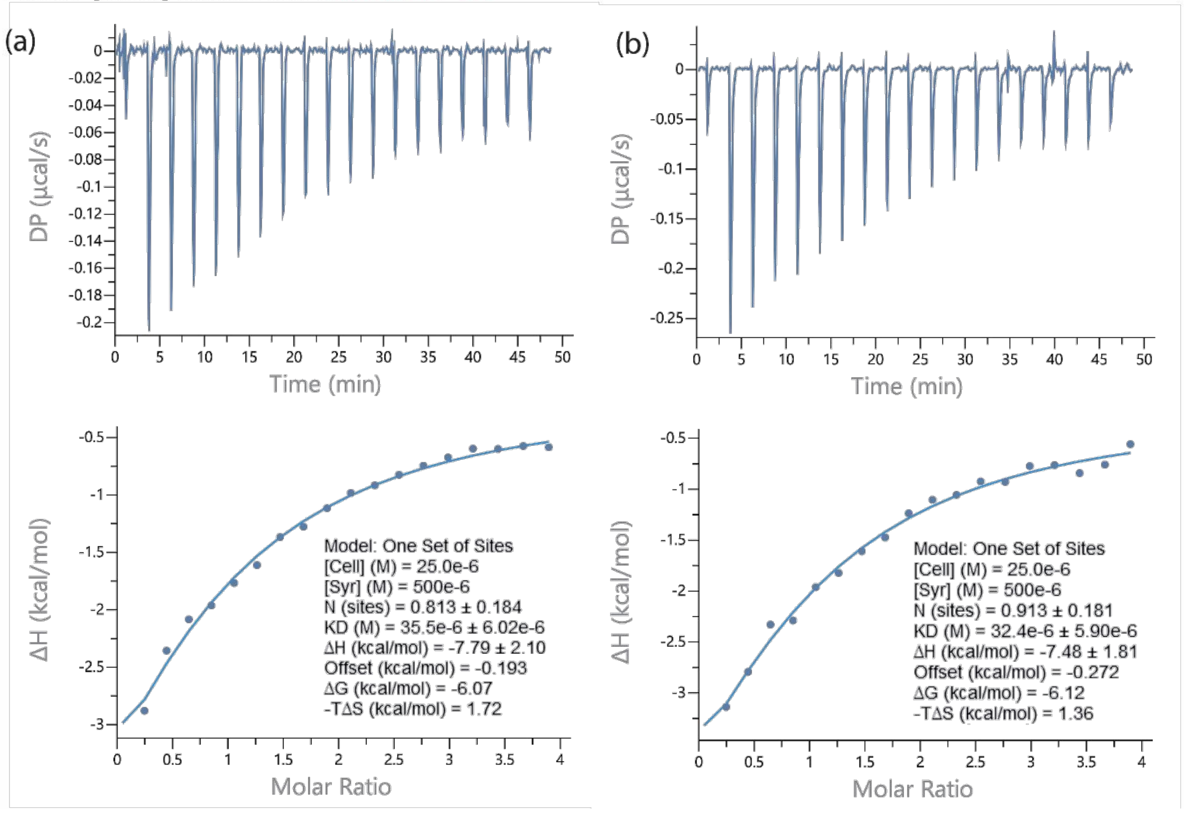

Figure S26. Duplicate isothermal titration calorimetry for N-terminally capped peptide N1C3T-[N086] with SHANK-1 PDZ. Shown in the upper panels are the raw heat plots and in the lower panels the corresponding integrated, baseline-corrected heats per injection. Listed for each titration are the concentrations of the protein in the syringe and in the cell, as well as the parameters of the fit (stoichiometry *N*, dissociation constant K_d).

N1C3T-[C047] titration into SHANK-1 PDZ

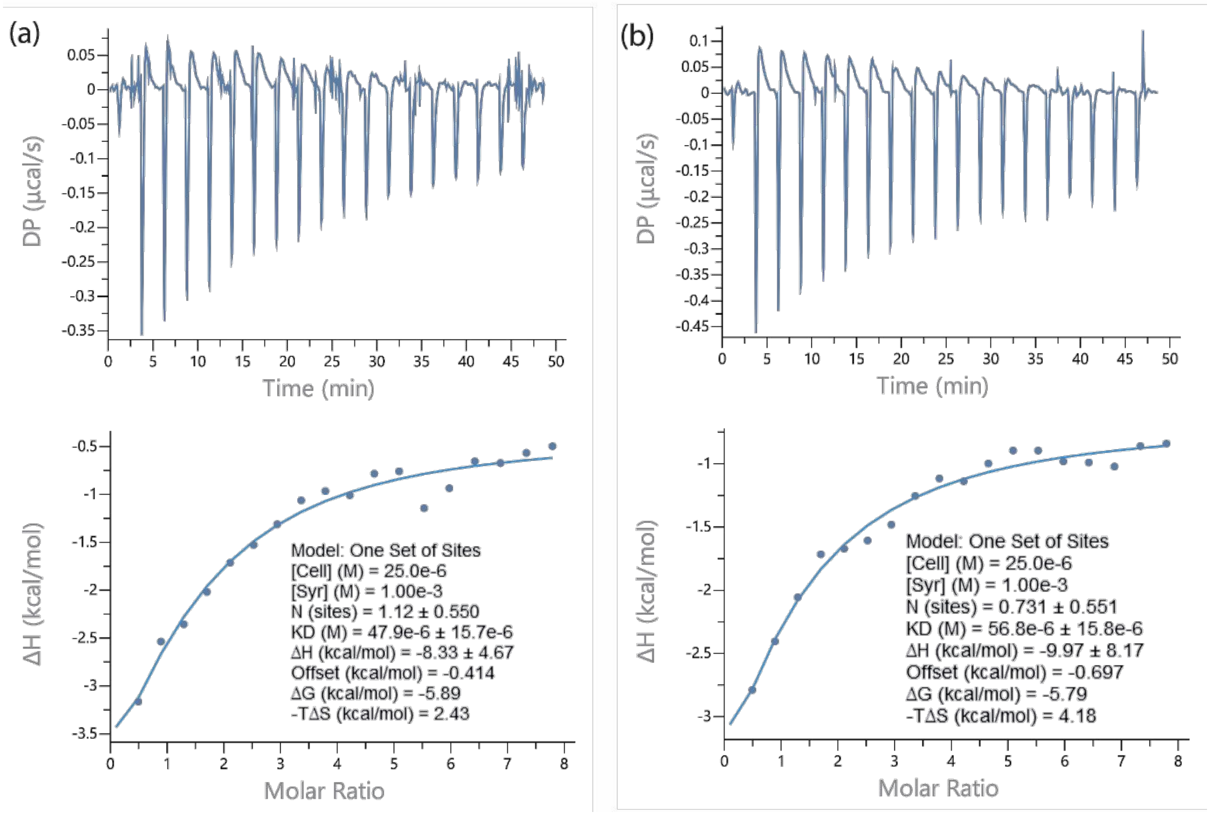

Figure S14. Duplicate isothermal titration calorimetry for C-terminally capped peptide N1C3T-[C047] with SHANK-1 PDZ. Shown in the upper panels are the raw heat plots and in the lower panels the corresponding integrated, baseline-corrected heats per injection. Listed for each titration are the concentrations of the protein in the syringe and in the cell, as well as the parameters of the fit (stoichiometry *N*, dissociation constant K_d).

N1C3T-[N086-C047] titration into SHANK-1 PDZ

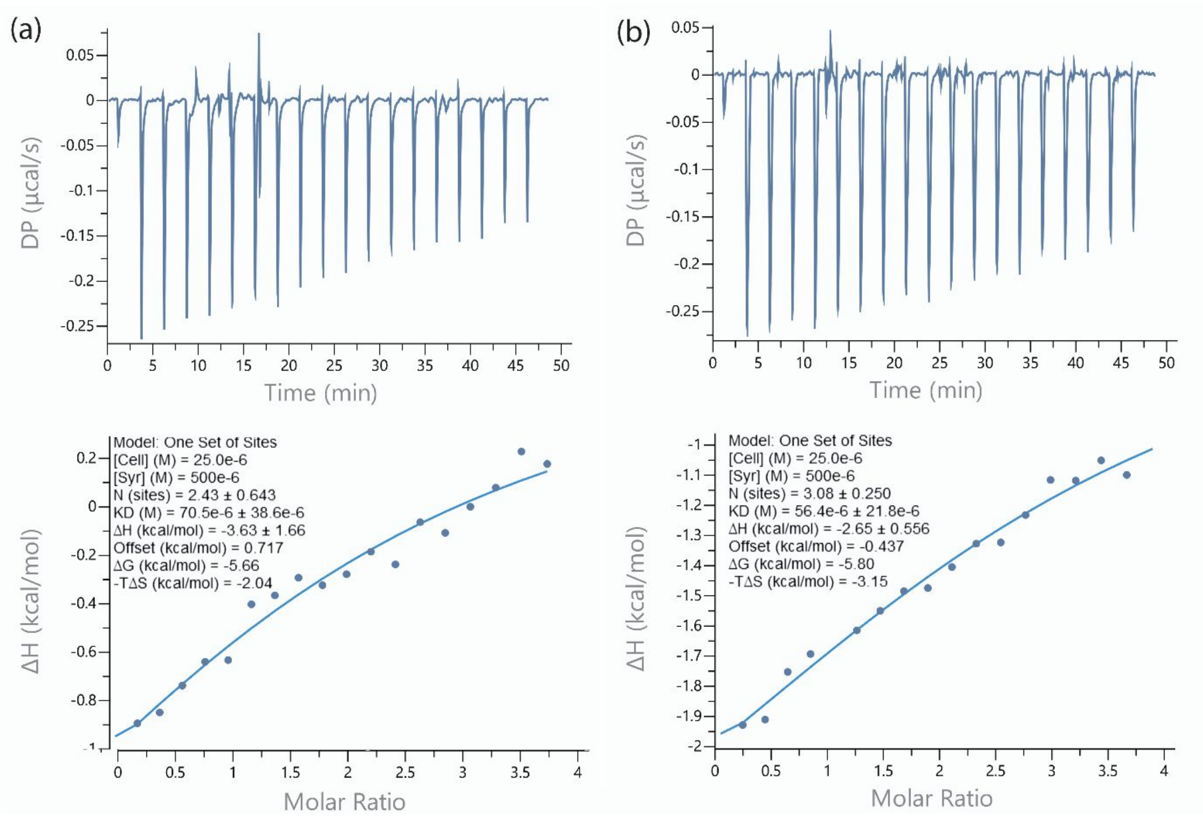

Figure S15. Duplicate isothermal titration calorimetry for N-terminally and C-terminally capped peptide N1C3T-[N086-C047] with SHANK-1 PDZ. Shown in the upper panels are the raw heat plots and in the lower panels the corresponding integrated, baseline-corrected heats per injection. Listed for each titration are the concentrations of the protein in the syringe and in the cell, as well as the parameters of the fit (stoichiometry N, dissociation constant K_d). Lower heats for this peptide lead to a shallower isotherm where saturation is difficult to discern; there is a higher N-value and therefore error on all parameters.

Protein Characterization

SDS-PAGE gel for the SHANK1 PDZ protein expression and purification

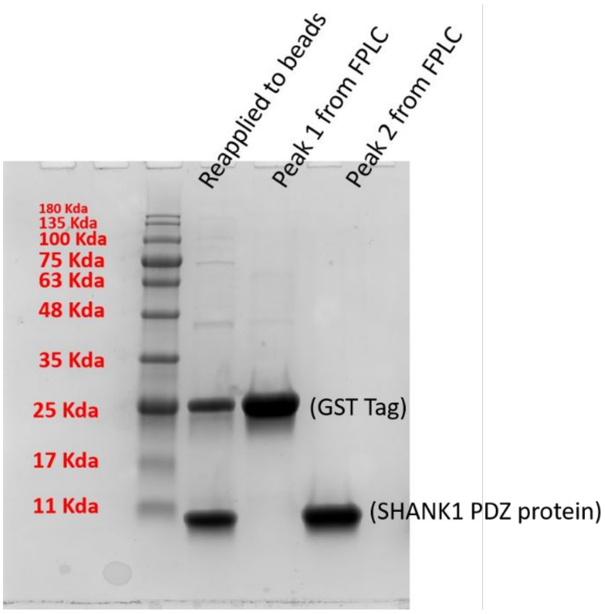

Sequence and mass spectra of the expressed SHANK1-PDZ protein.

SHANK1-PDZ$_{656\text{-}762}$:

$_{656}$DYIIKEKTVLLQKKDSEGFGFVLRGAKAQTPIEEFTPTPAFPALQYLESVDEGGVAWRAGL

RMGDFLIEVNGQNVVKVGHRQVVNMIRQGGNTLMVKVVMVTRHPDM$_{762}$

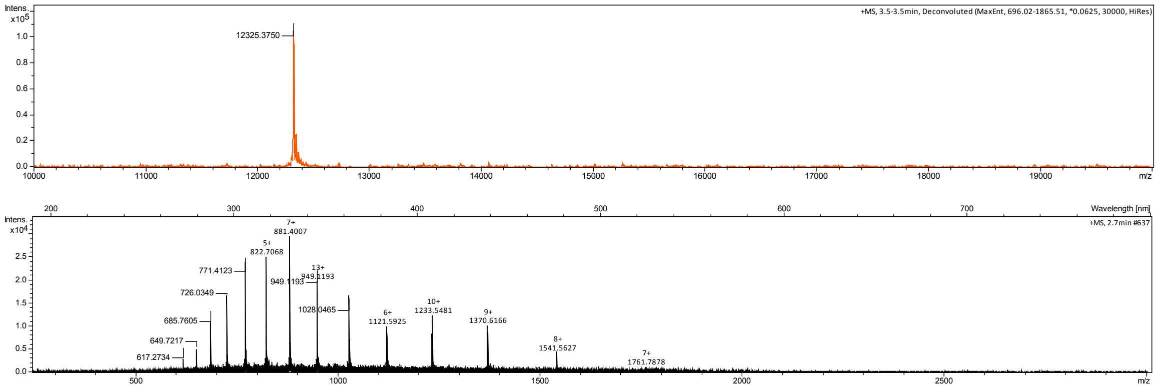

.

Chromatographic conditions for preparative HPLC

Table S1. Chromatographic conditions for preparative HPLC[α]

Time (min)	Mobile phase A (%)	Mobile phase B (%)	Flow (mL/min)
0	90	10	15
20	50	40	15
25	20	80	15
30	0	100	15
33	0	100	15
35	90	10	15

[α]. Reversed Phase C18 column, mobile phase A (0.1% TFA in water), mobile phase B (0.1% TFA in ACN).

Chromatographic conditions for analytical HPLC

Table S2. Chromatographic conditions for analytical HPLC[α]

Time (min)	Mobile phase A (%)	Mobile phase B (%)	Flow (mL/min)
0	90	10	15
40	0	100	15

[α]. Reversed Phase C18 column, mobile phase A (0.1% FA in water), mobile phase B (ACN).

Characterisation Data

FAM-Ahx-EESTSFQGFP-$CONH_2$ and **Ac-EESTSFQGP-$CONH_2$** were described previously

N1T

Exact Mass: 892.3927

**Expected $[M+H^+]^+$**	**Measured $[M+H^+]^+$**
893.3999	893.4053
**Expected $[M+2H^+]^{2+}$**	**Measured $[M+2H^+]^{2+}$**
447.2044	447.2035

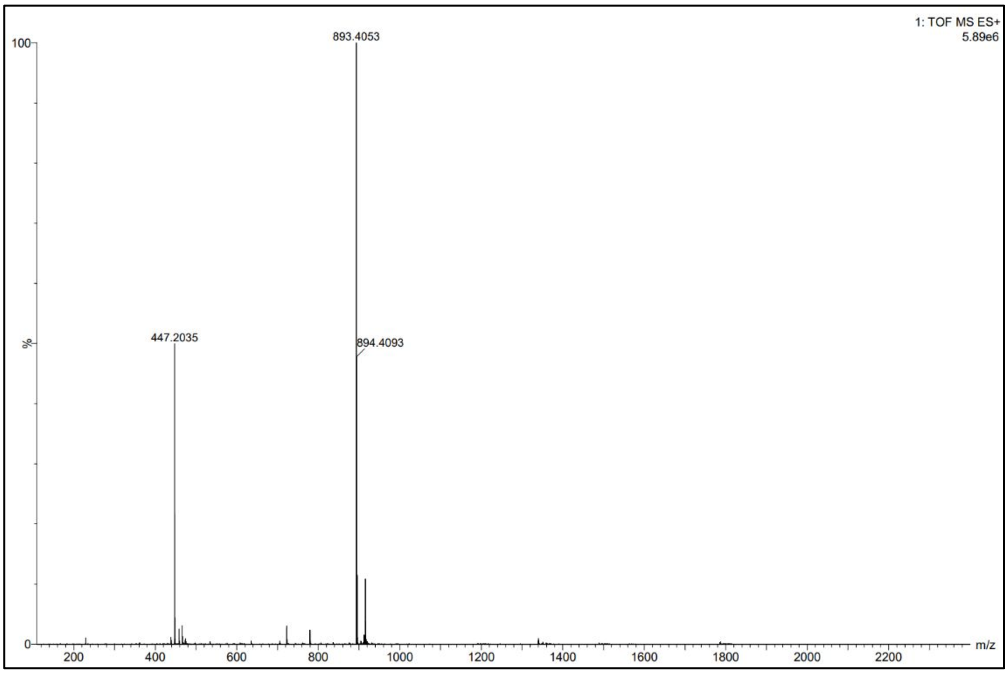

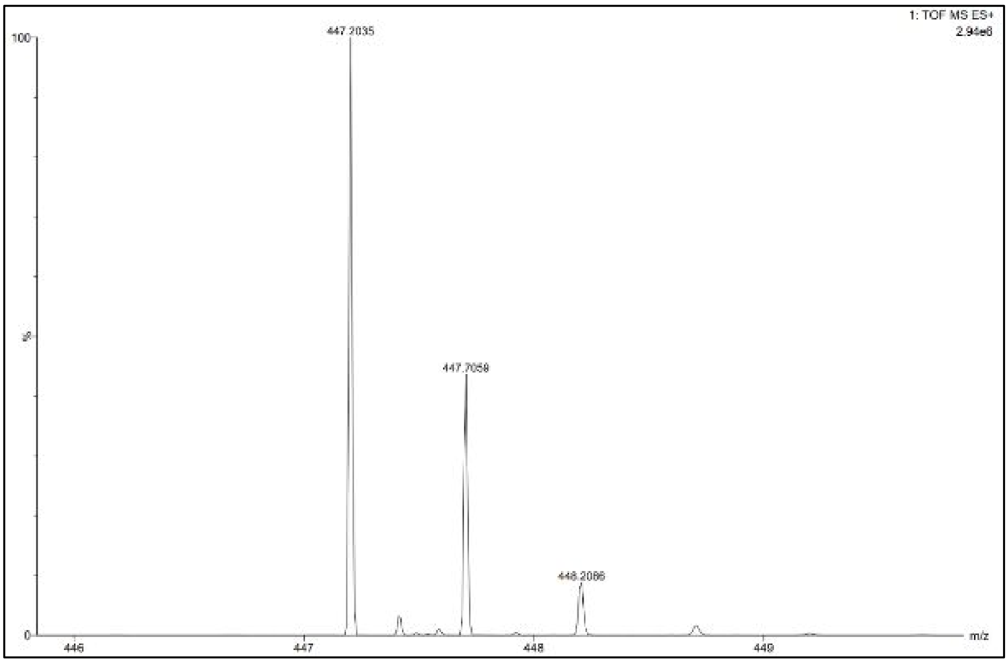
1: TOF MS ES+
2.94e6
100
%
0
447.2035
447.7059
448.2086
446
447
448
449
m/z

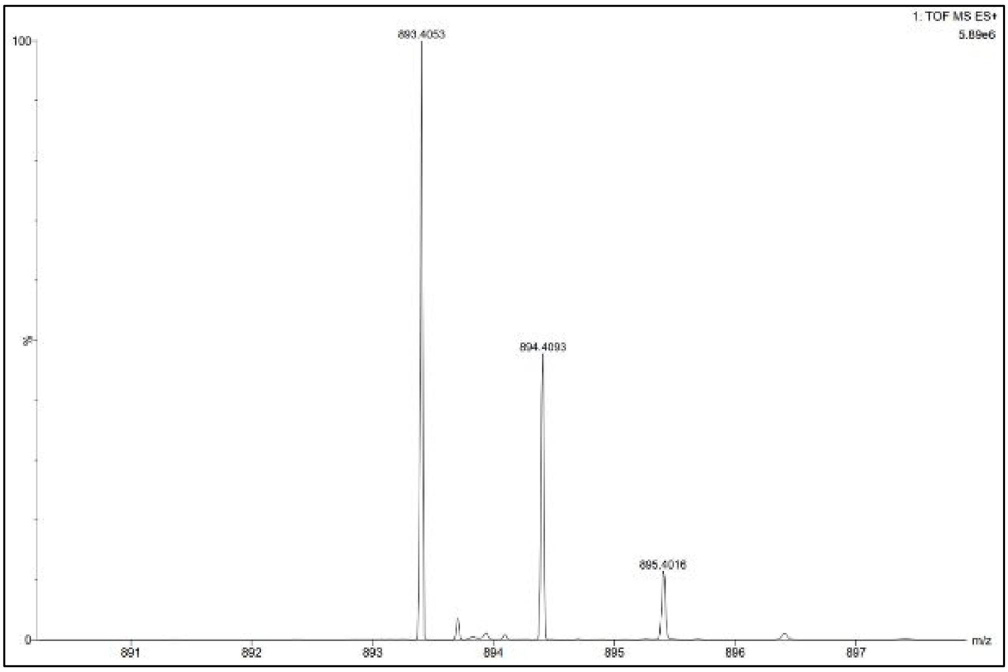
1: TOF MS ES+
5.89e6
100
%
0
893.4053
894.4093
895.4016
891
892
893
894
895
896
897
m/z

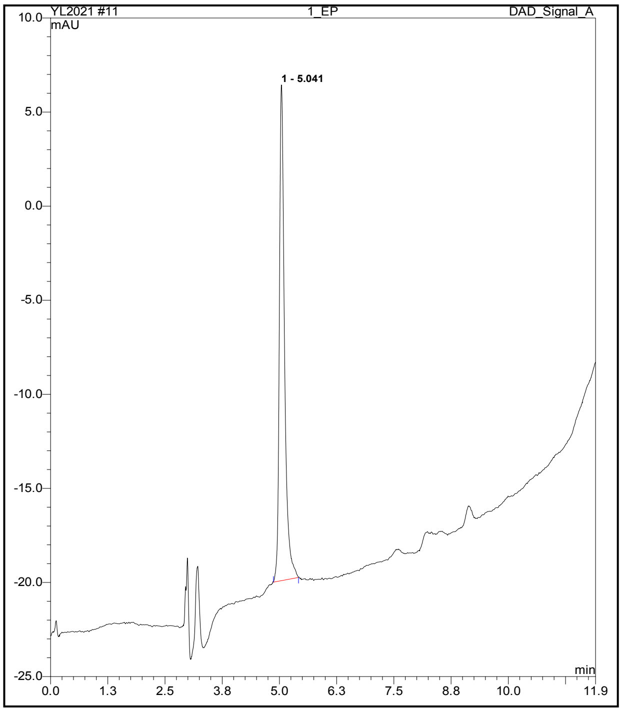

Analytical HPLC: Purity = 100%

N2T

Exact Mass: 763.3501

**Expected [M+H⁺]⁺**	**Measured [M+H⁺]⁺**
764.3573	764.3585
**Expected [M+2H⁺]²⁺**	**Measured [M+2H⁺]²⁺**
382.6831	382.6797
**Expected [M+Na⁺]⁺**	**Measured [M+Na⁺]⁺**
786.3393	786.3423

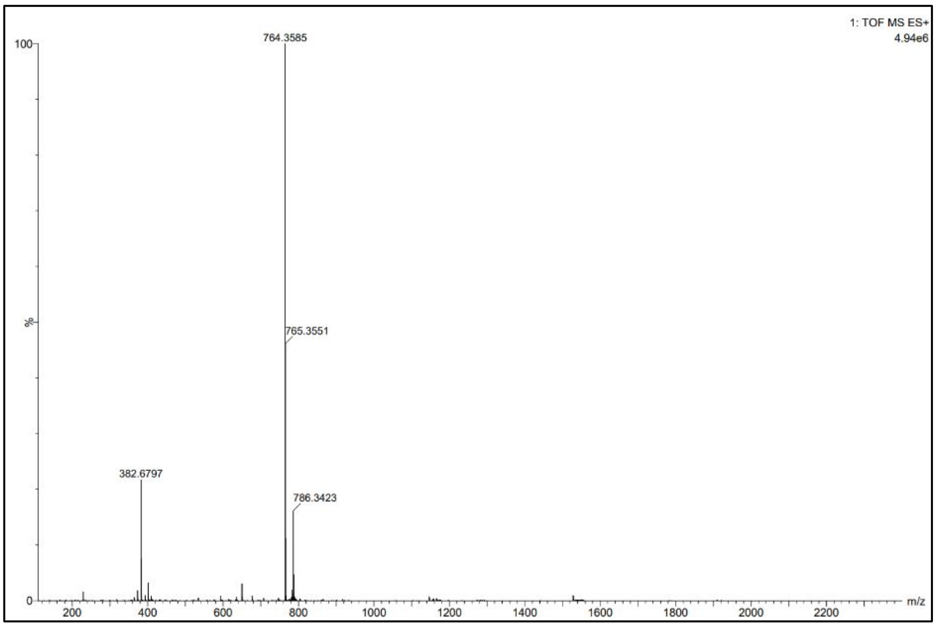

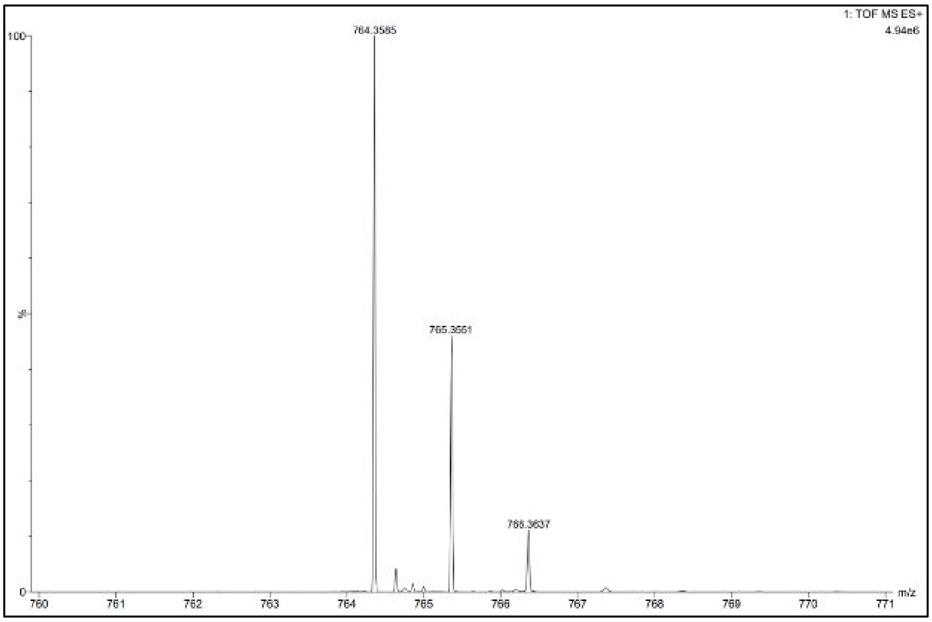

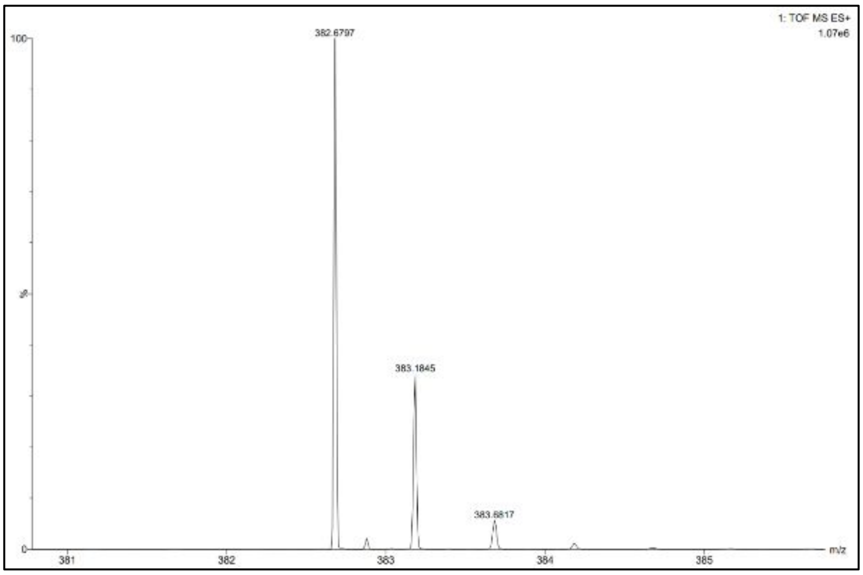

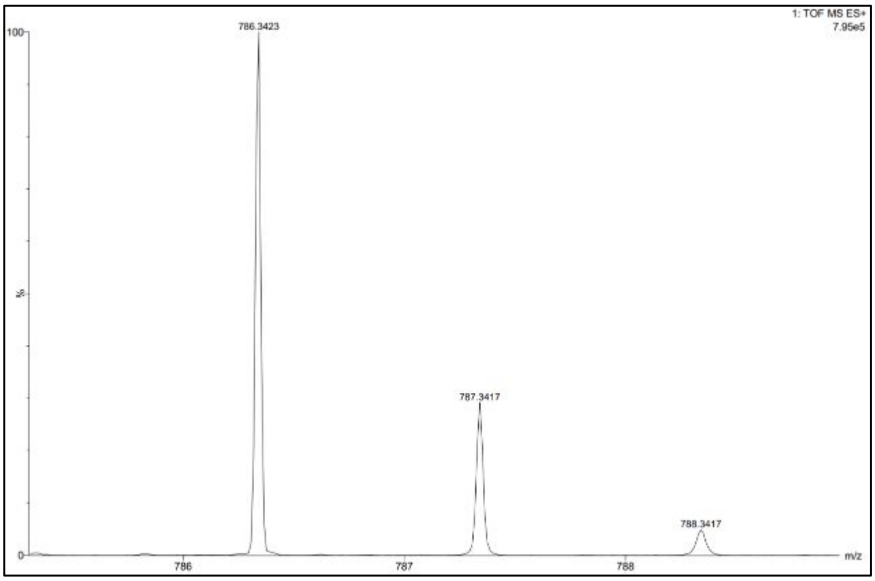

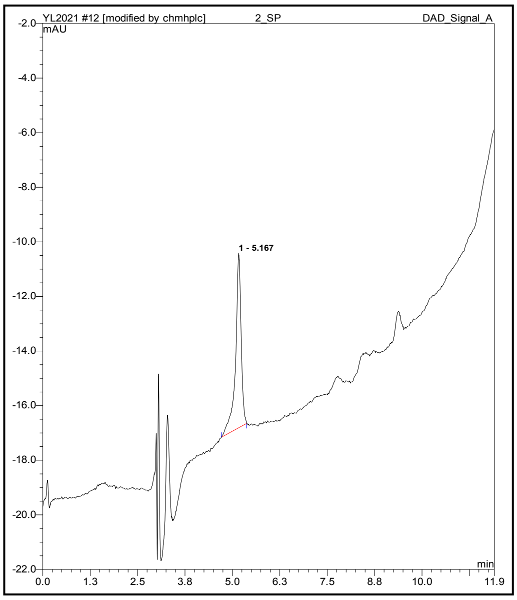

Analytical HPLC: Purity = 100%

C1T

Exact Mass: 924.3925

**Expected [M+H⁺]⁺**	**Measured [M+H⁺]⁺**
925.3898	925.3882
**Expected [M+2H⁺]²⁺**	**Measured [M+2H⁺]²⁺**
463.2043	463.2002

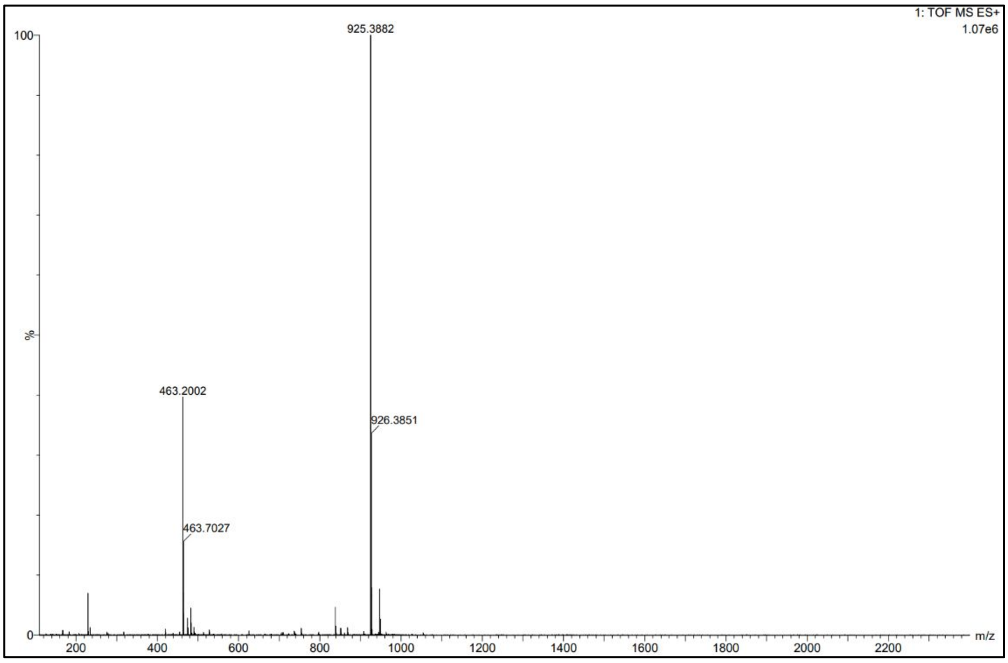

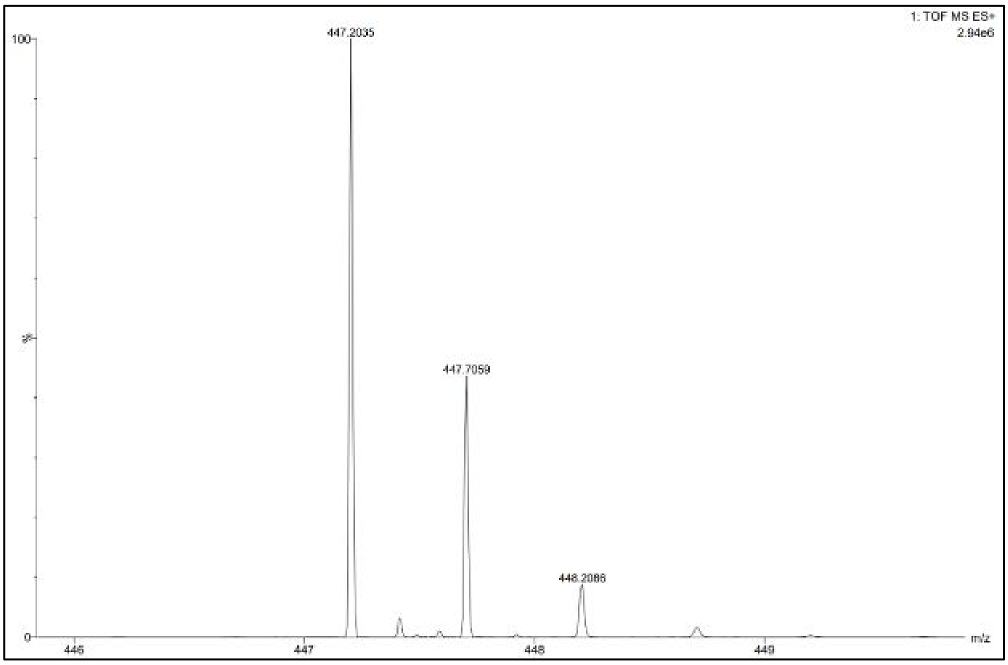

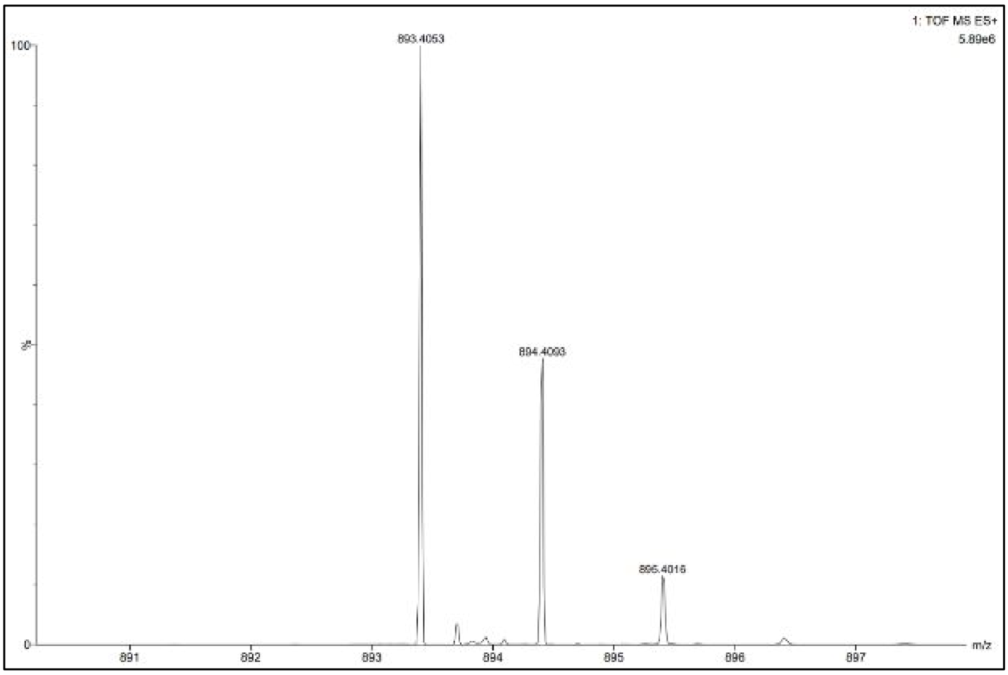

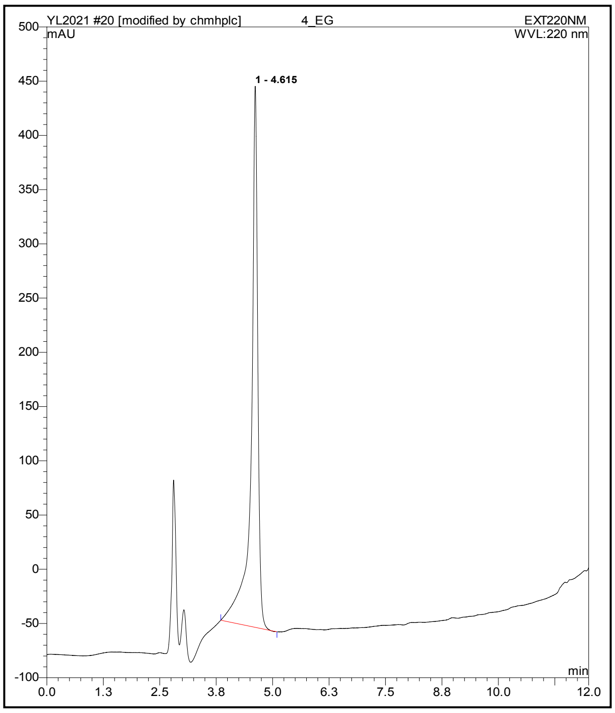

Analytical HPLC: Purity = 100%

C2T

Exact Mass: 867.3610

**Expected [M+H⁺]⁺**	**Measured [M+H⁺]⁺**
868.3683	868.3688
**Expected [M+2H⁺]²⁺**	**Measured [M+2H⁺]²⁺**
434.6878	434.6921

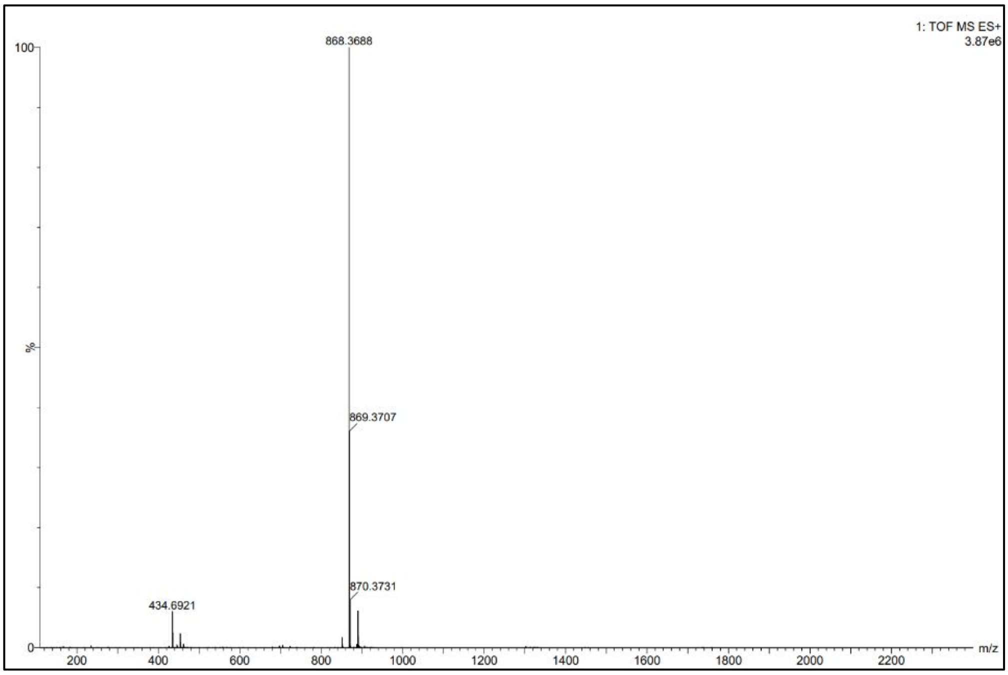

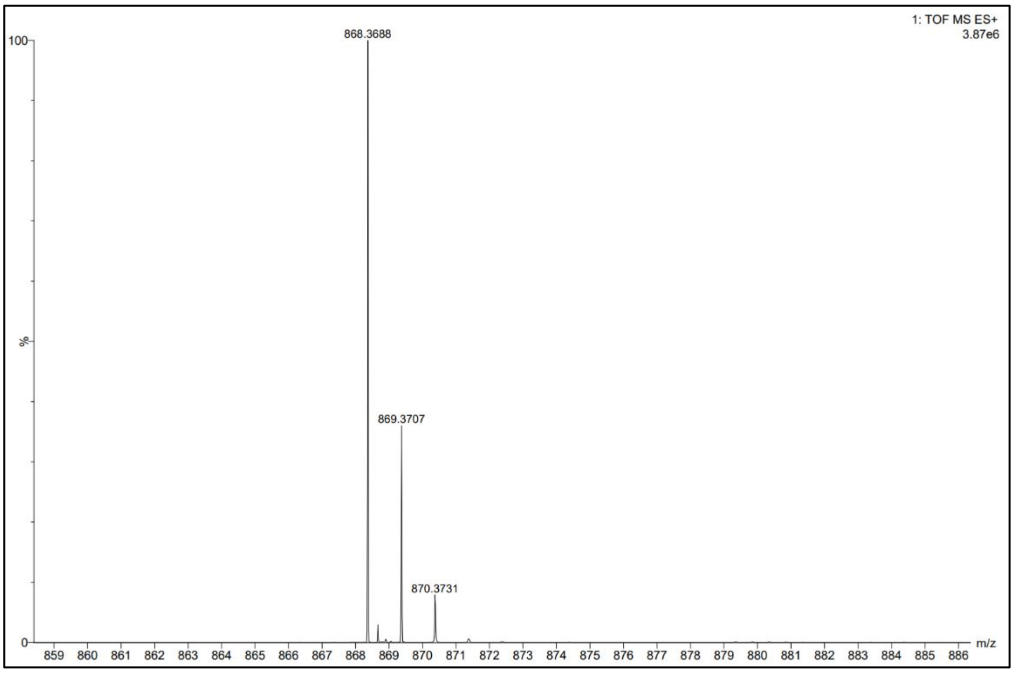

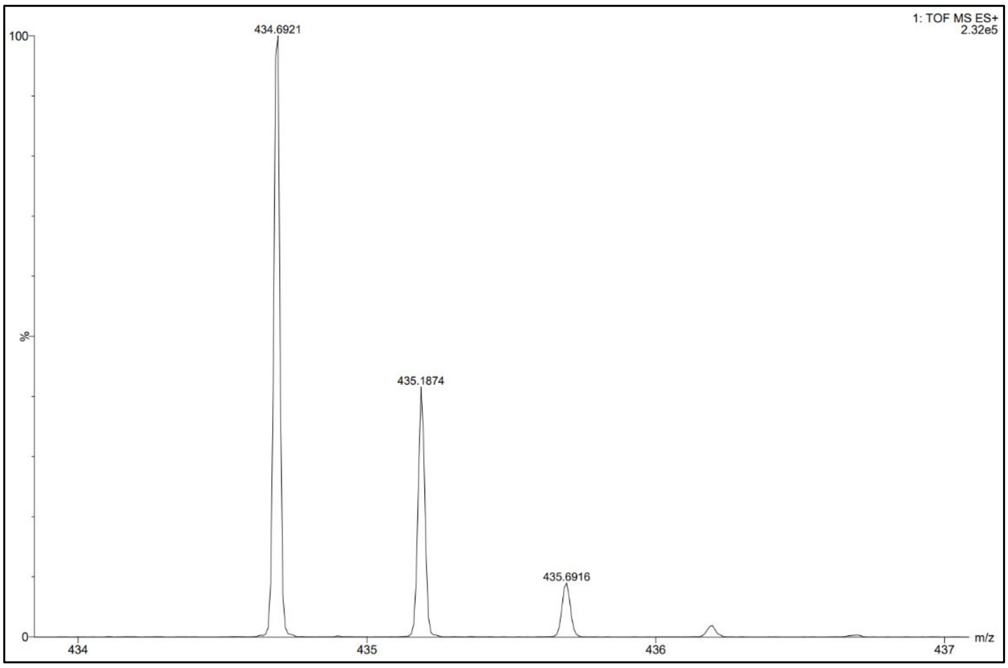

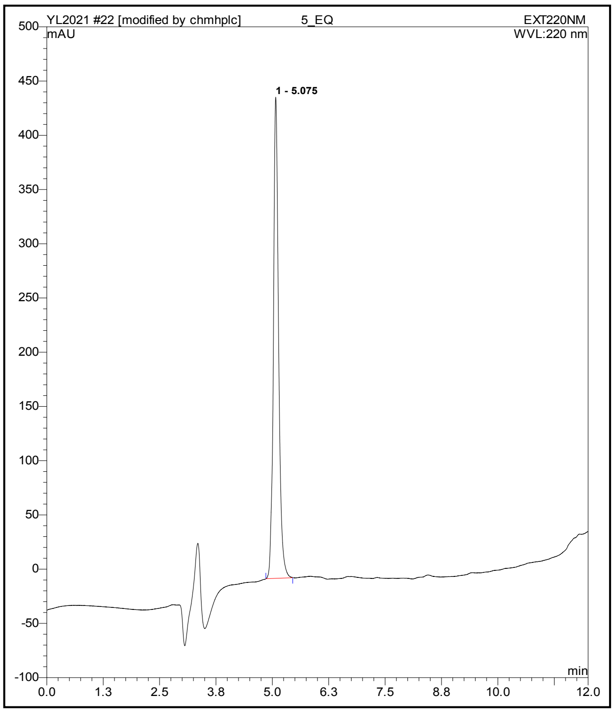

Analytical HPLC: Purity = 100%

C3T

Exact Mass: 739.3024

**Expected [M+H⁺]⁺**	**Measured [M+H⁺]⁺**
740.3097	740.3112
**Expected [M+Na⁺]⁺**	**Measured [M+Na⁺]⁺**
762.2917	762.2881
**Expected [M+K⁺+H⁺]²⁺**	**Measured [M+K⁺+H⁺]²⁺**
389.6365	389.6323

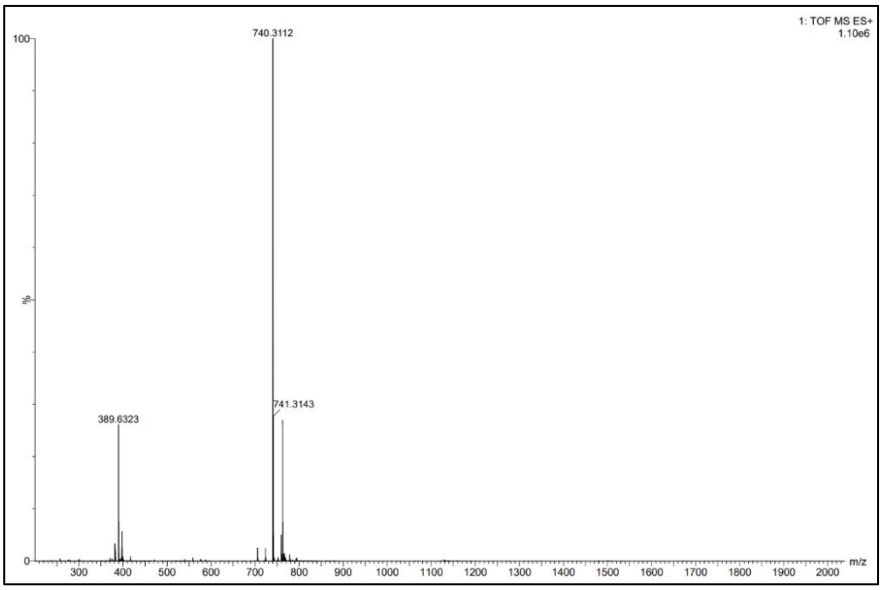

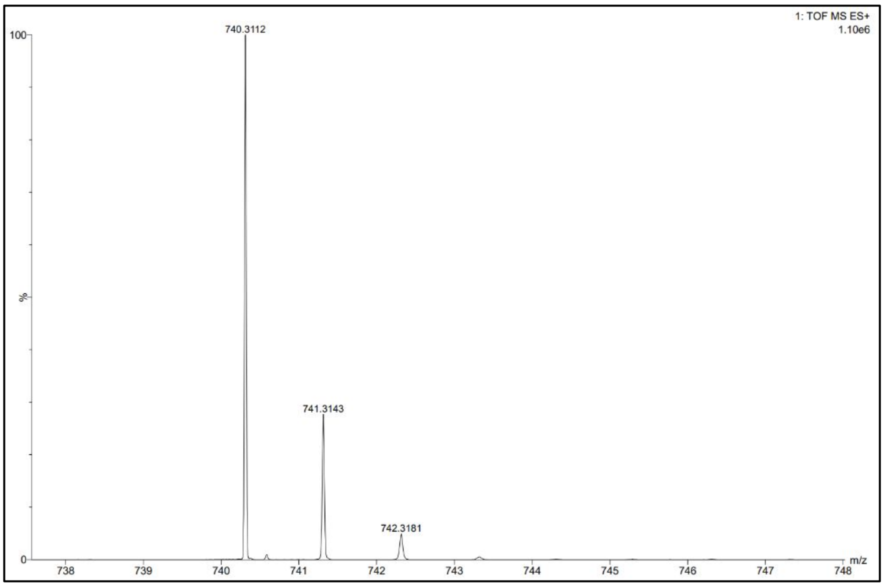

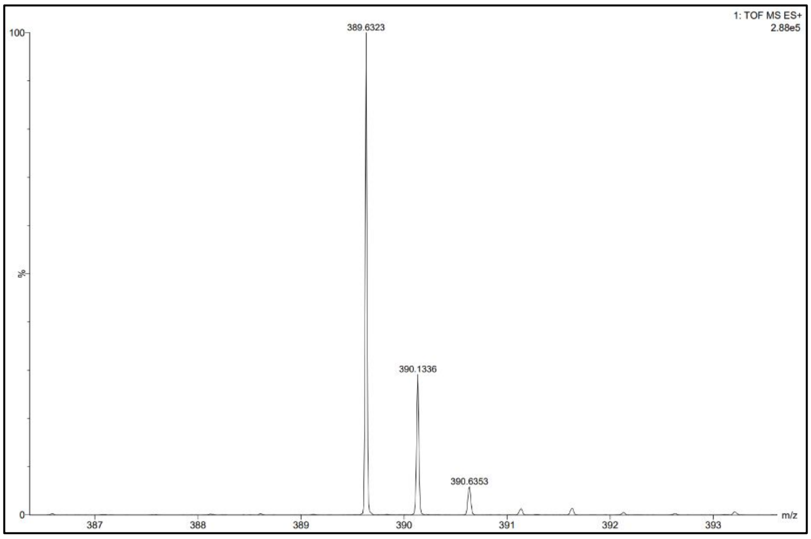

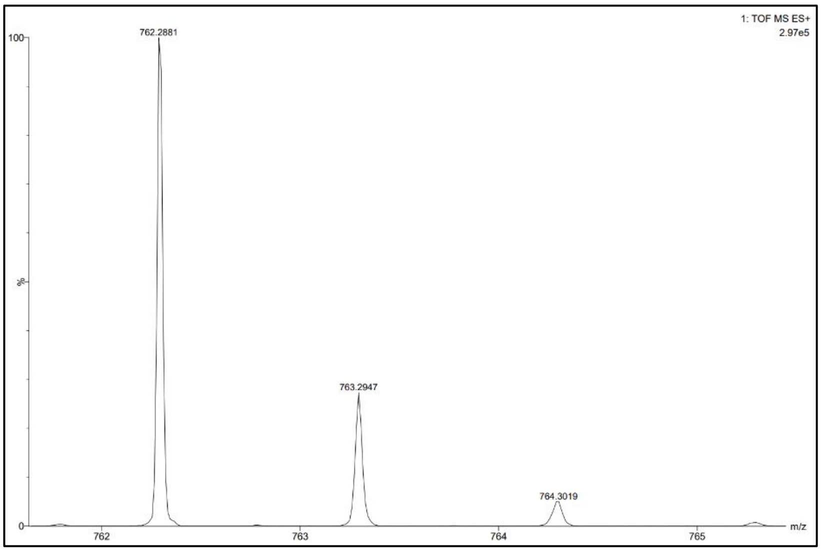

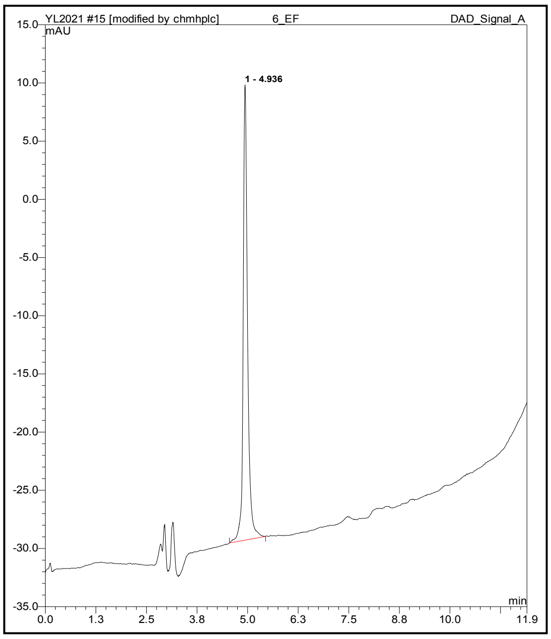

Analytical HPLC: Purity = 100%

C4T

Exact Mass: 592.2340

Expected [M+H⁺]⁺	Measured [M+H⁺]⁺
593.2413	593.2375
**Expected [M+Na⁺]⁺**	**Measured [M+Na⁺]⁺**
615.2233	615.2208

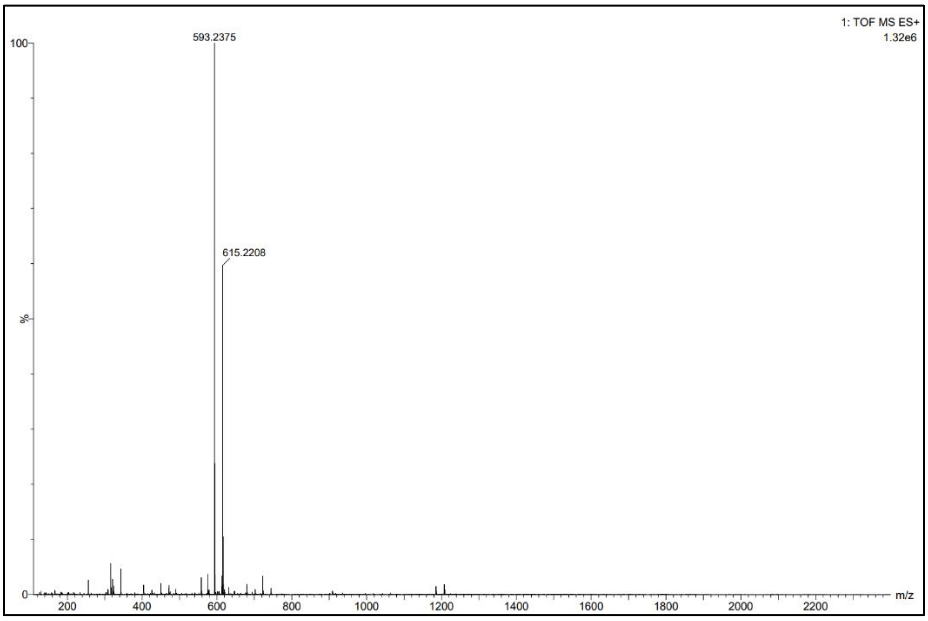

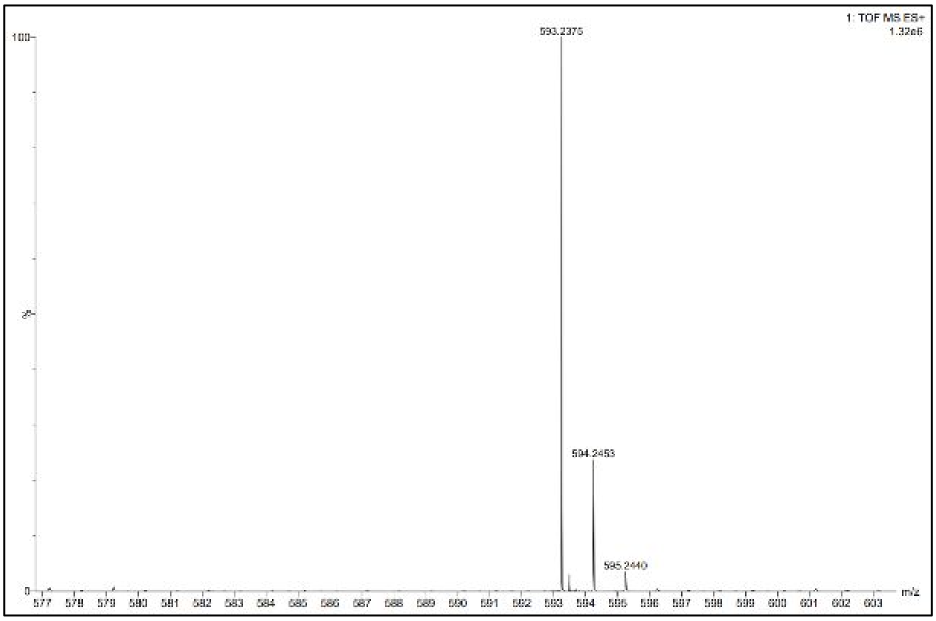

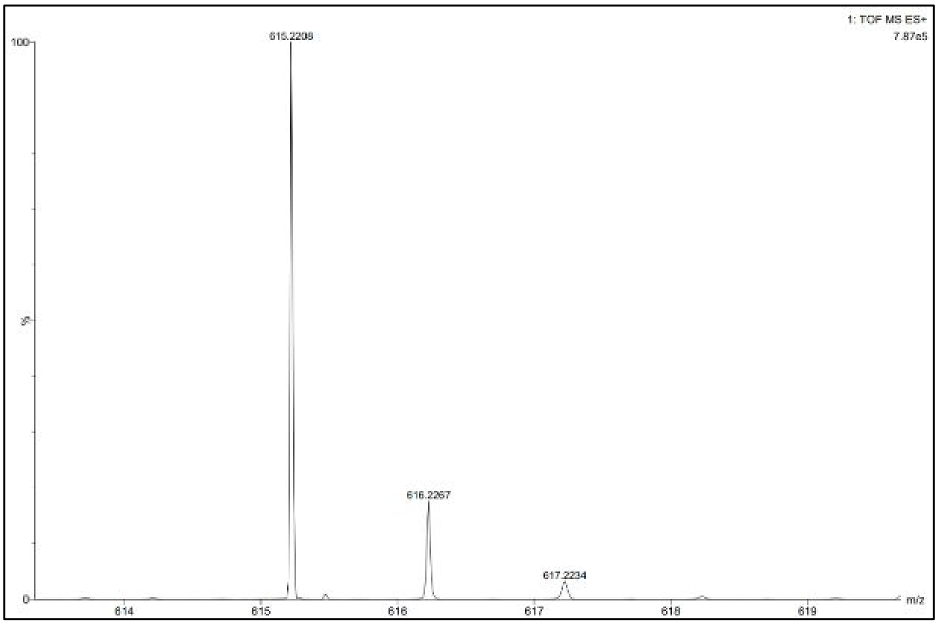

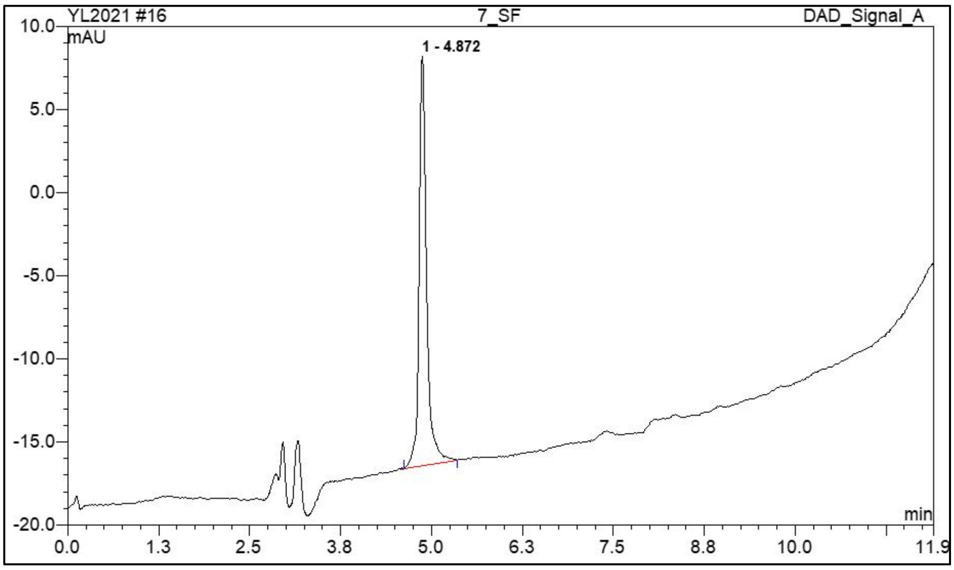

Analytical HPLC: Purity = 100%

N1C3T

Exact Mass: 610.2599

**Expected [M+H⁺]⁺**	**Measured [M+H⁺]⁺**
611.2671	611.2659
**Expected [M+Na⁺]⁺**	**Measured [M+Na⁺]⁺**
633.2491	633.2479

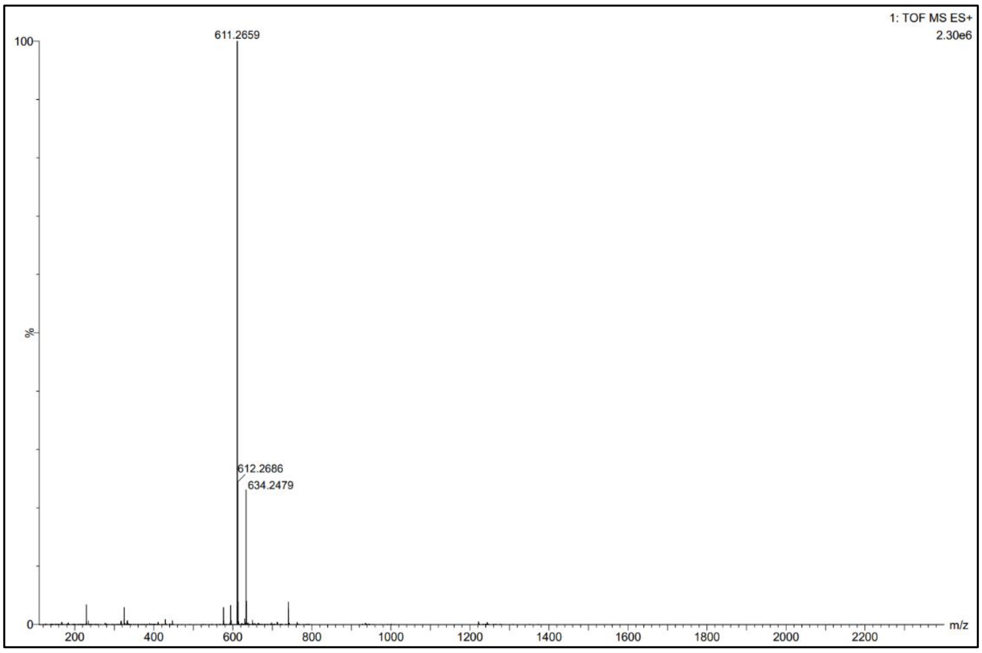

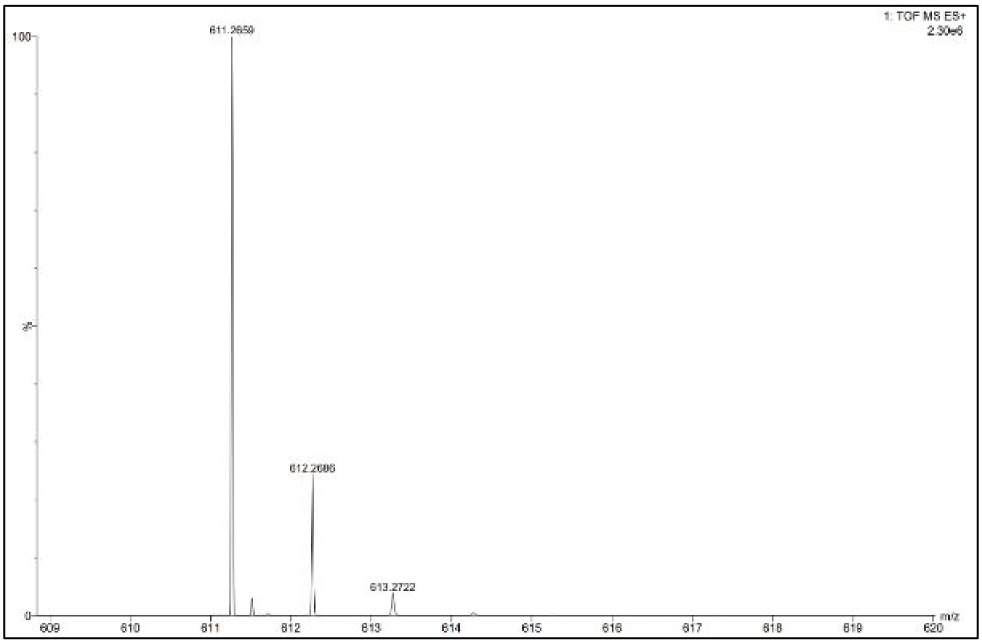

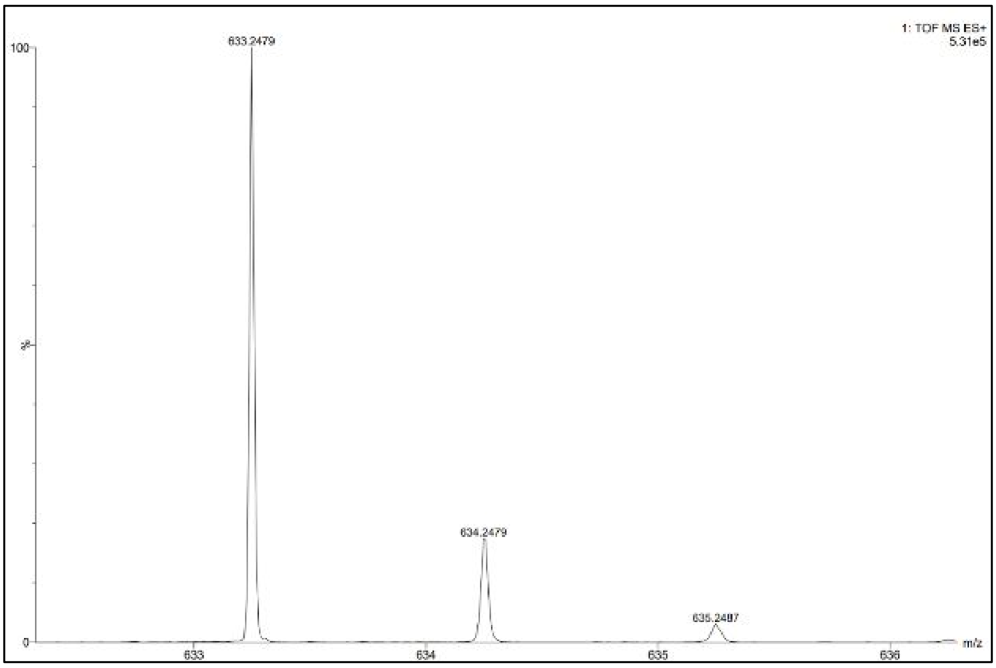

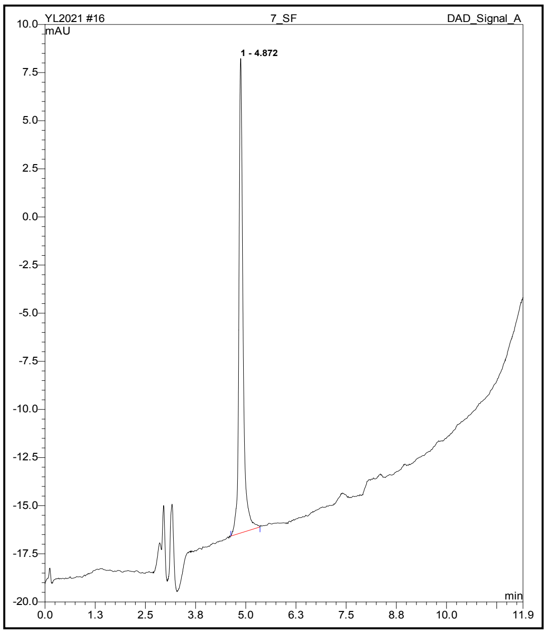

Analytical HPLC: Purity = 100%

C1T-[C001]

Exact Mass: 1027.4247

**Expected [M+H⁺]⁺**	**Measured [M+H⁺]⁺**
1028.4320	1028.4247
**Expected [M+2H⁺]²⁺**	**Measured [M+2H⁺]²⁺**
514.7196	514.7149
**Expected [M+Na⁺]⁺**	**Measured [M+Na⁺]⁺**
1050.4139	1050.4064
**Expected [M+H⁺+K⁺]²⁺**	**Measured [M+H⁺+K⁺]²⁺**
533.6976	533.6907

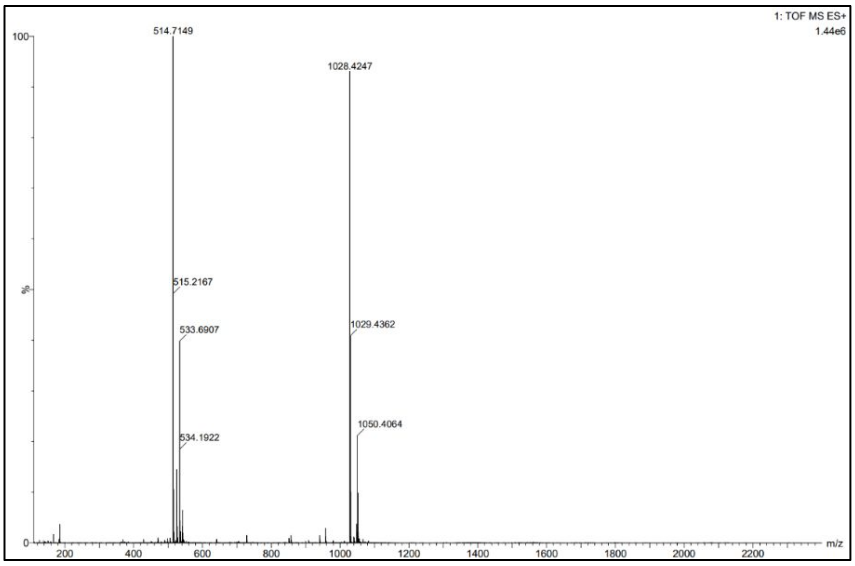

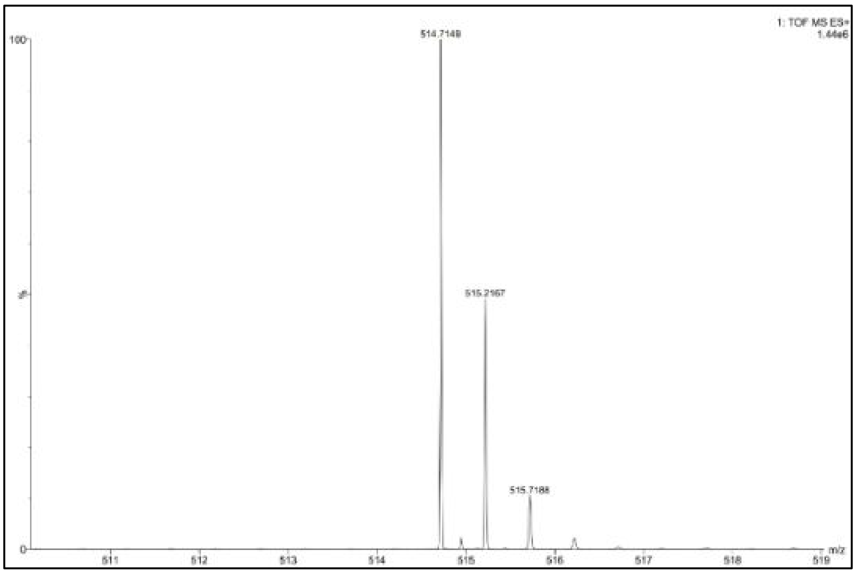

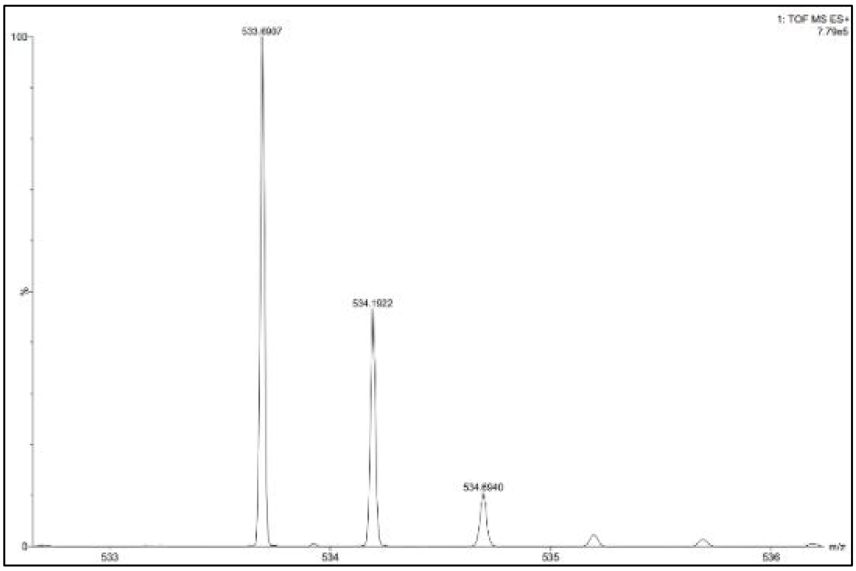
1: TOF MS ES+
7.79e5
533.6907
534.1922
534.6940
100
%
0
533
534
535
536
m/z

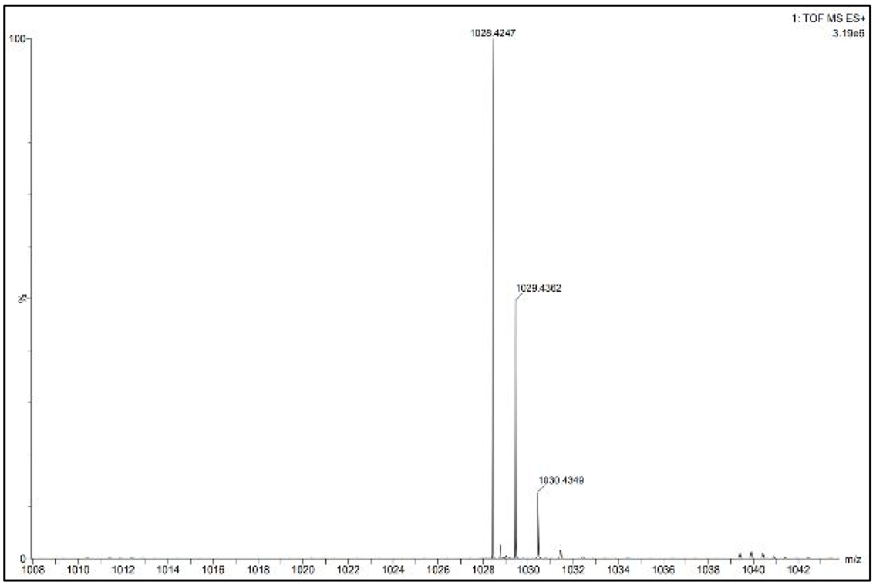
1: TOF MS ES+
3.19e6
1028.4247
1029.4362
1030.4349
100
%
0
1008
1010
1012
1014
1016
1018
1020
1022
1024
1026
1028
1030
1032
1034
1036
1038
1040
1042
m/z

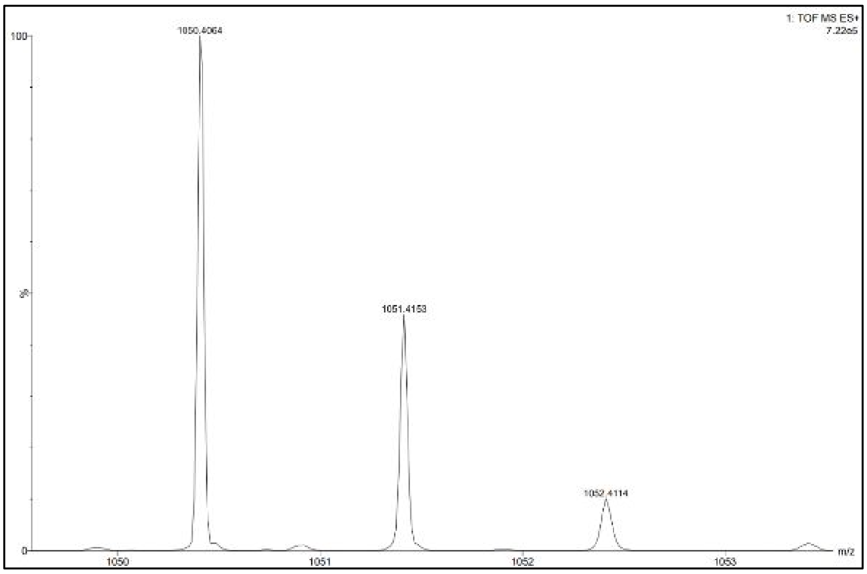
1: TOF MS ES+
7.22e5
1050.4064
1051.4153
1052.4114
100
%
0
1050
1051
1052
1053
m/z

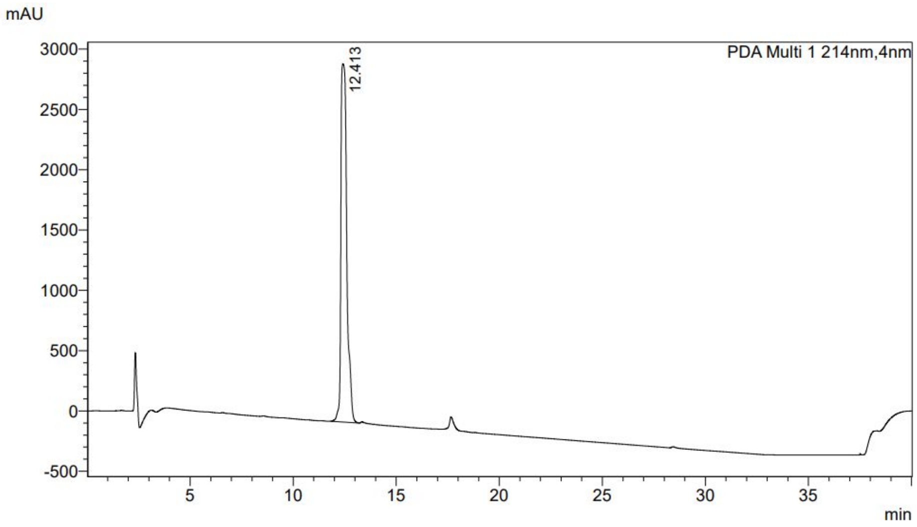

Analytical HPLC: purity = 99%

C2T-[C001]

Exact Mass: 970.4032

**Expected [M+H⁺]⁺**	**Measured [M+H⁺]⁺**
971.4105	971.4155
**Expected [M+Na⁺]⁺**	**Measured [M+Na⁺]⁺**
993.3924	993.3890
**Expected [M+2H⁺]²⁺**	**Measured [M+2H⁺]²⁺**
486.2096	486.2087

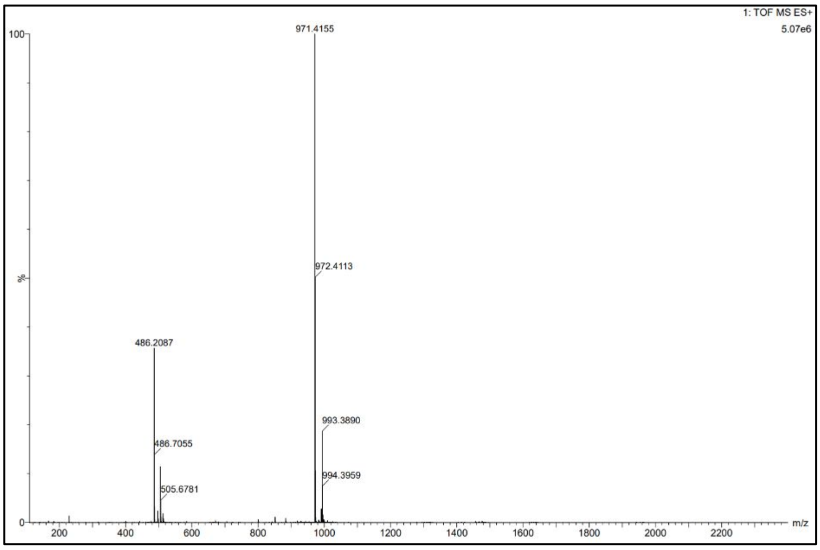

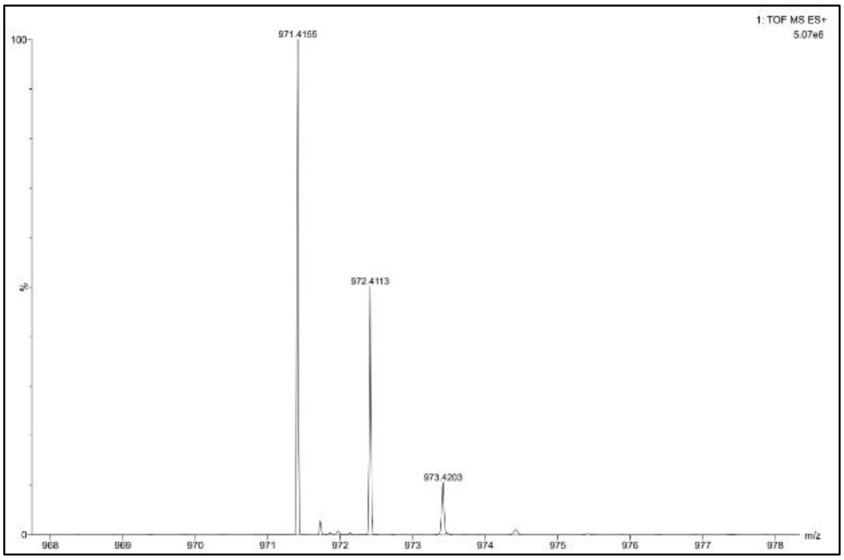

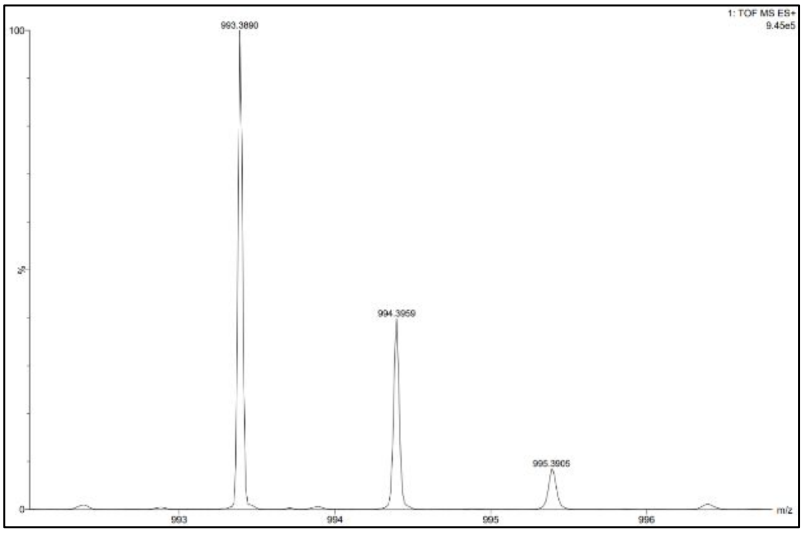

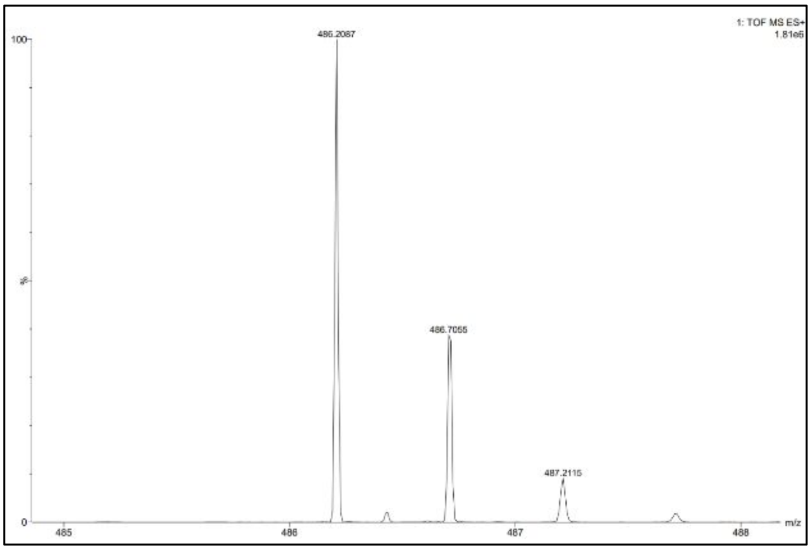

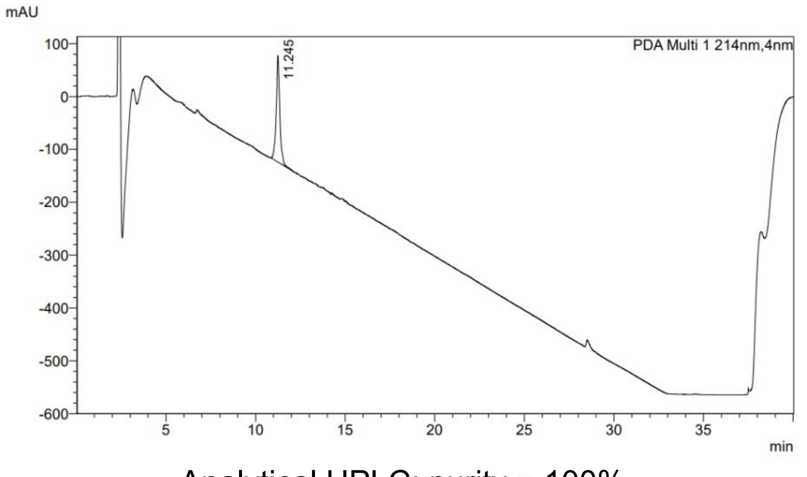

Analytical HPLC: purity = 100%

C3T-[C001]

Exact Mass: 842.3446

Expected $[M+H^+]^+$	Measured $[M+H^+]^+$
843.3519	843.3550
**Expected $[M+Na^+]^+$**	**Measured $[M+Na^+]^+$**
865.3339	865.3305
**Expected $[M+K^++H^+]^{2+}$**	**Measured $[M+K^++H^+]^{2+}$**
441.1576	441.1535

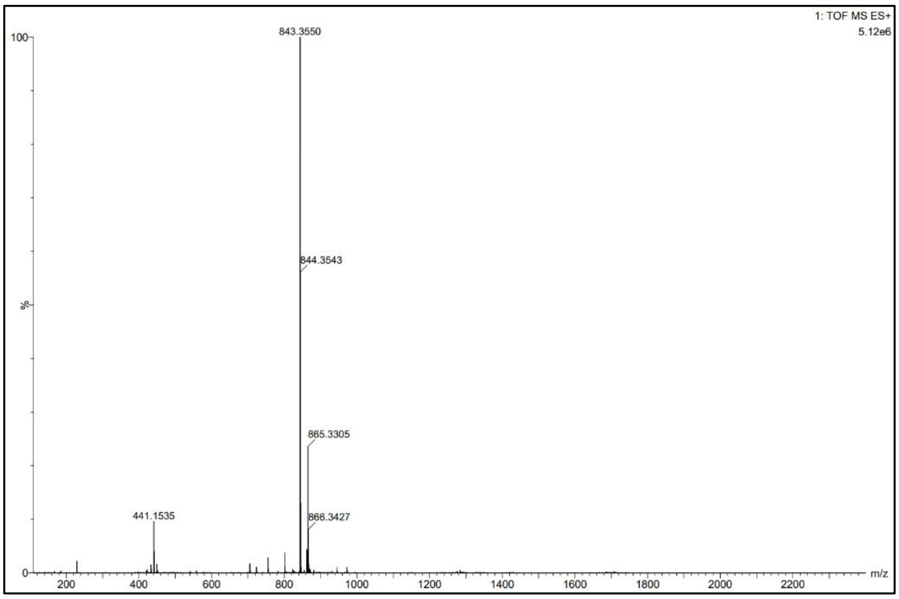

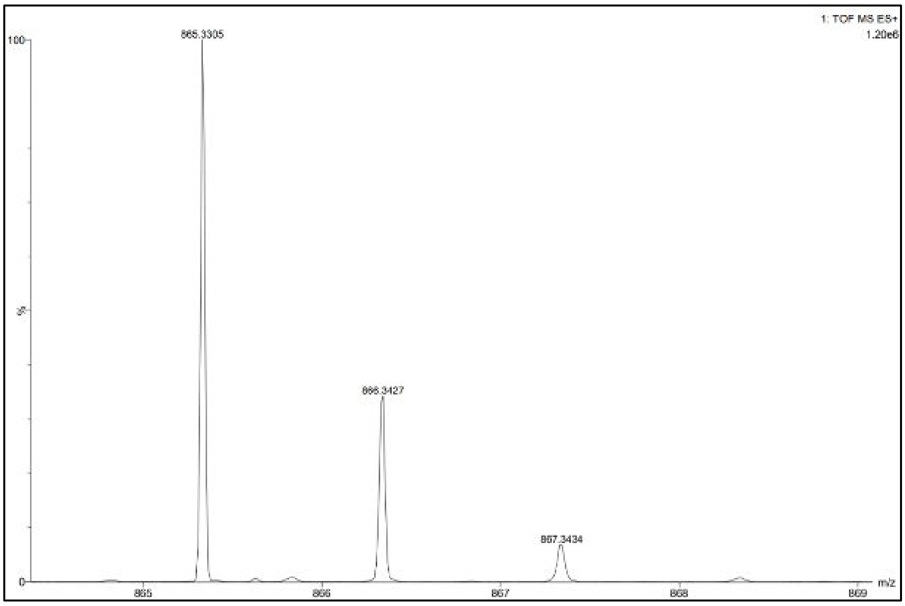

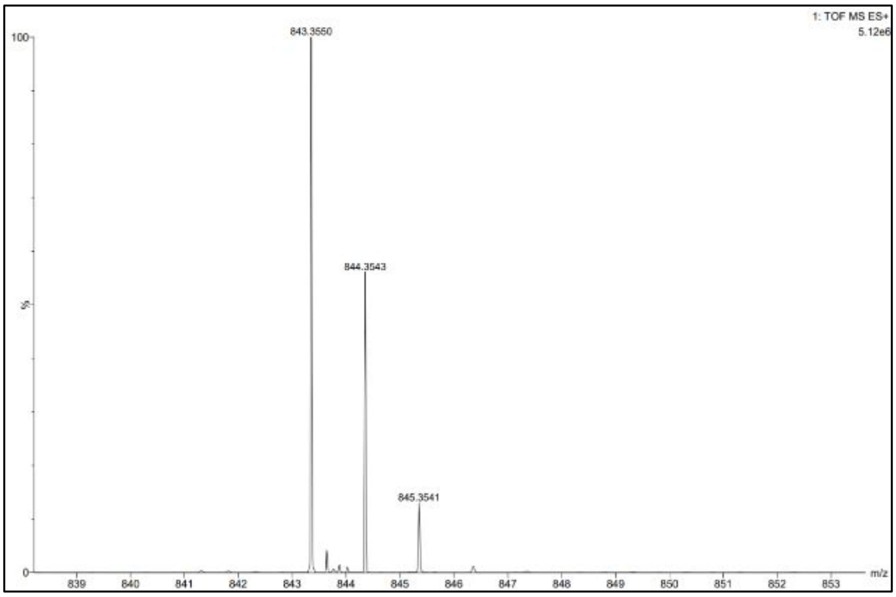

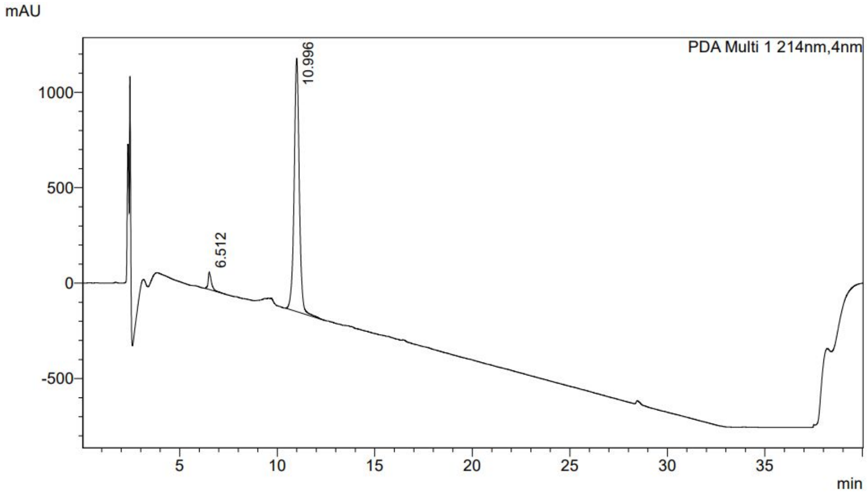

Analytical HPLC: purity = 99%

C1T-[C007]

Exact Mass: 1147.4458

Expected $[M+H^+]^+$	Measured $[M+H^+]^+$
1148.4531	1148.4465
**Expected $[M+Na^+]^+$**	**Measured $[M+Na^+]^+$**
1170.4350	1170.4229
**Expected $[M+2H^+]^{2+}$**	**Measured $[M+2H^+]^{2+}$**
574.7302	574.7308
**Expected $[M+K^++H^+]^{2+}$**	**Measured $[M+K^++H^+]^{2+}$**
593.7082	593.7055

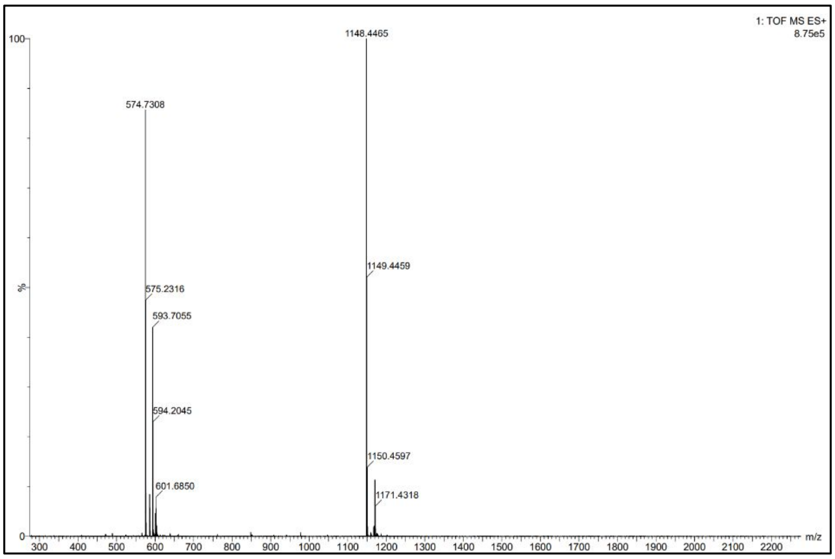

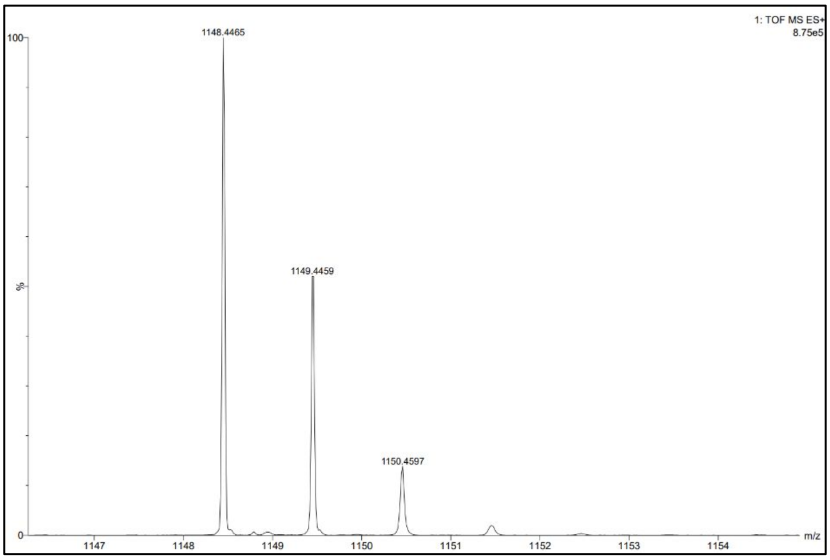

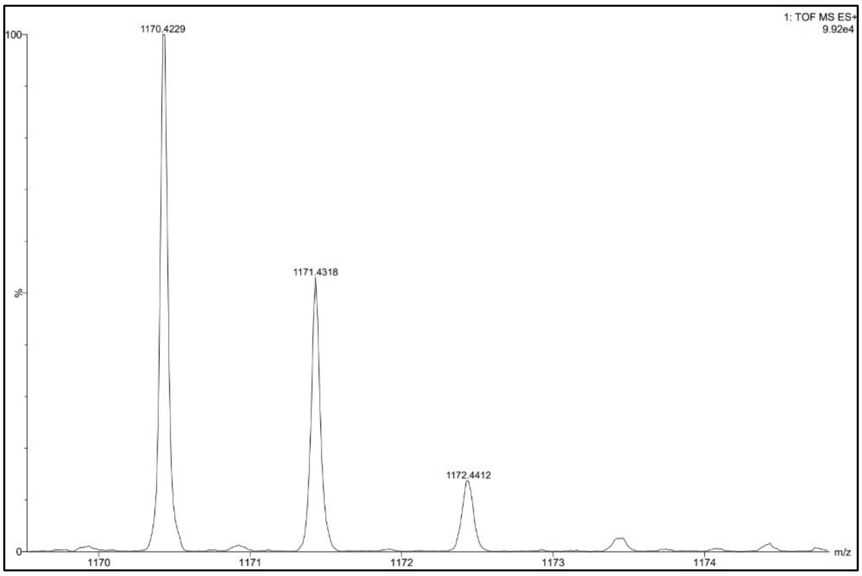
1: TOF MS ES+
9.92e4
100
%
0
1170.4229
1171.4318
1172.4412
1170
1171
1172
1173
1174
m/z

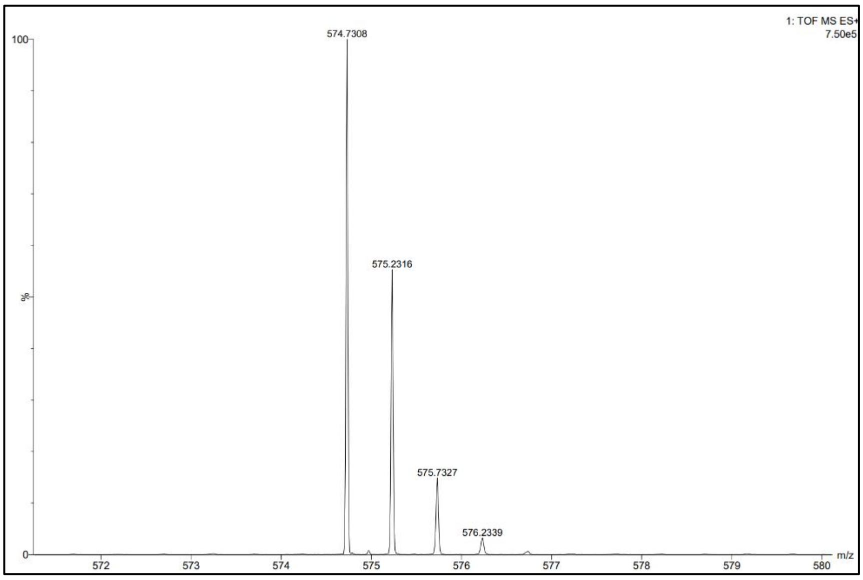
1: TOF MS ES+
7.50e5
100
%
0
574.7308
575.2316
575.7327
576.2339
572
573
574
575
576
577
578
579
580
m/z

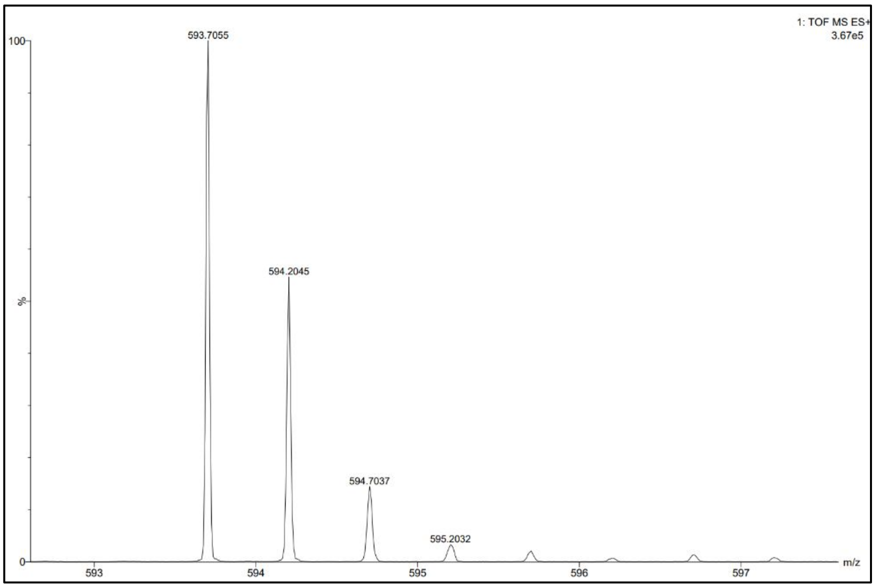
1: TOF MS ES+
3.67e5
100
%
0
593.7055
594.2045
594.7037
595.2032
593
594
595
596
597
m/z

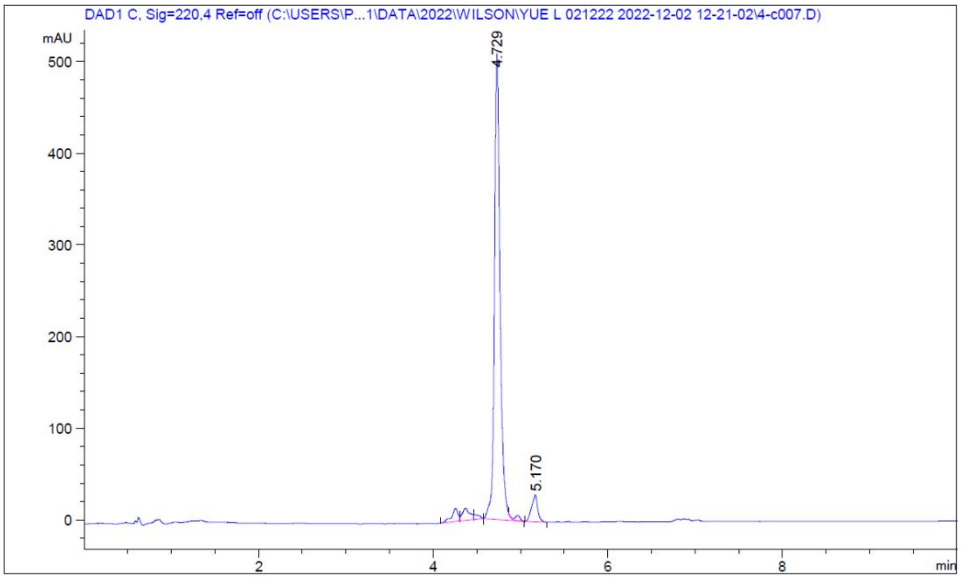

Analytical HPLC: purity = 96%

C1T-[C023]

Exact Mass: 1153.4200

Expected $[M+H^+]^+$	Measured $[M+H^+]^+$
1154.4273	1154.4219
**Expected $[M+Na^+]^+$**	**Measured $[M+Na^+]^+$**
1176.4092	1176.4128
**Expected $[M+2H^+]^{2+}$**	**Measured $[M+2H^+]^{2+}$**
577.7173	577.7192
**Expected $[M+Na^++H^+]^{2+}$**	**Measured $[M+Na^++H^+]^{2+}$**
588.7083	588.7068
**Expected $[M+K^++H^+]^{2+}$**	**Measured $[M+K^++H^+]^{2+}$**
596.6952	596.6928

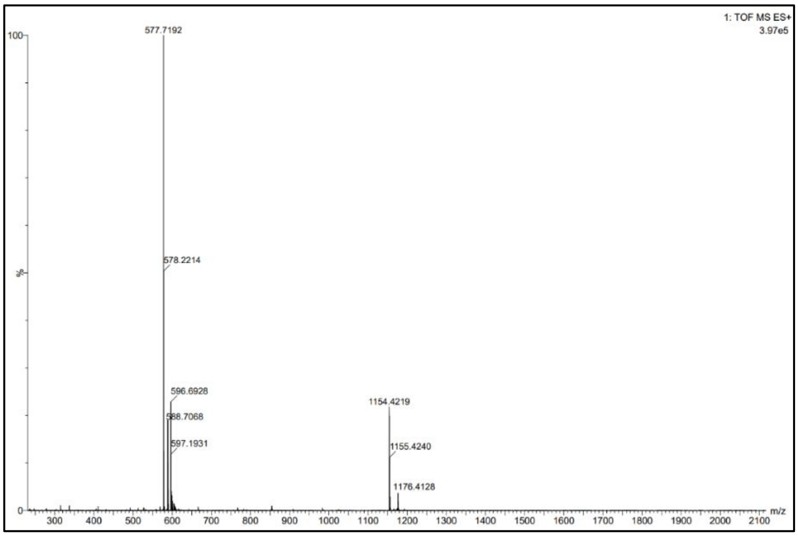

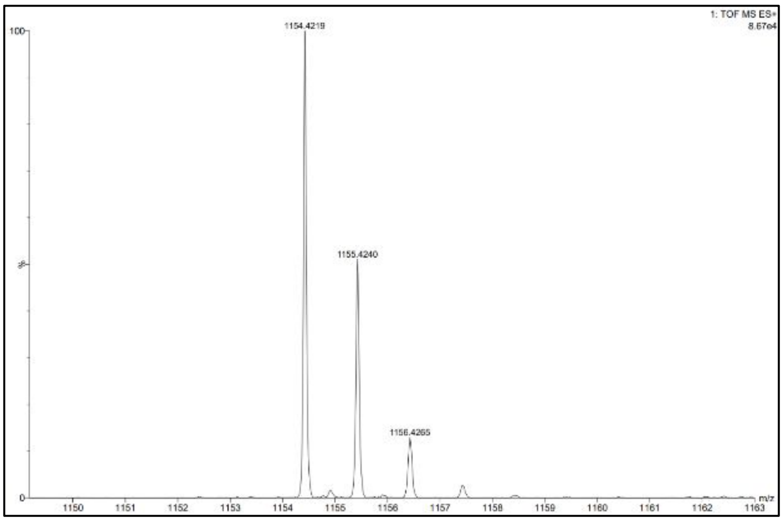

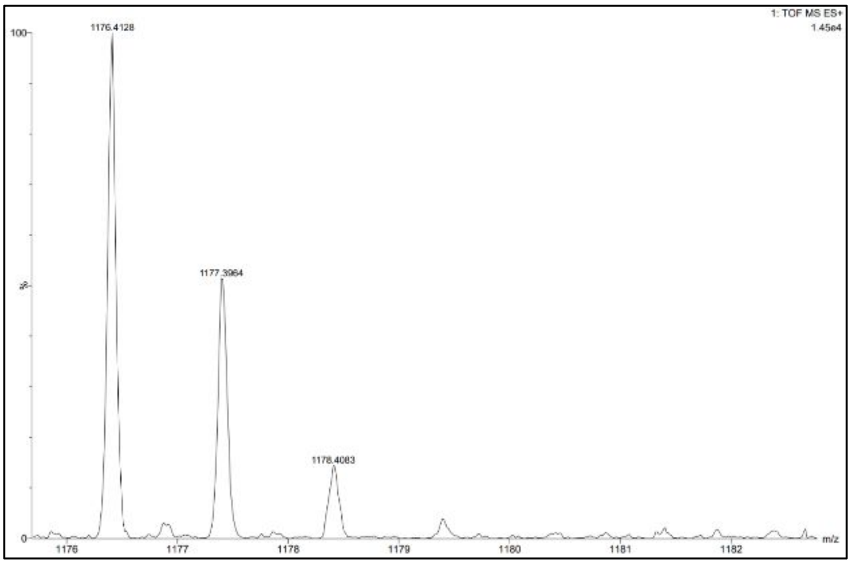
1: TOF MS ES+
1.45e4
1176.4128
1177.3964
1178.4083
100
%
0
1176
1177
1178
1179
1180
1181
1182
m/z

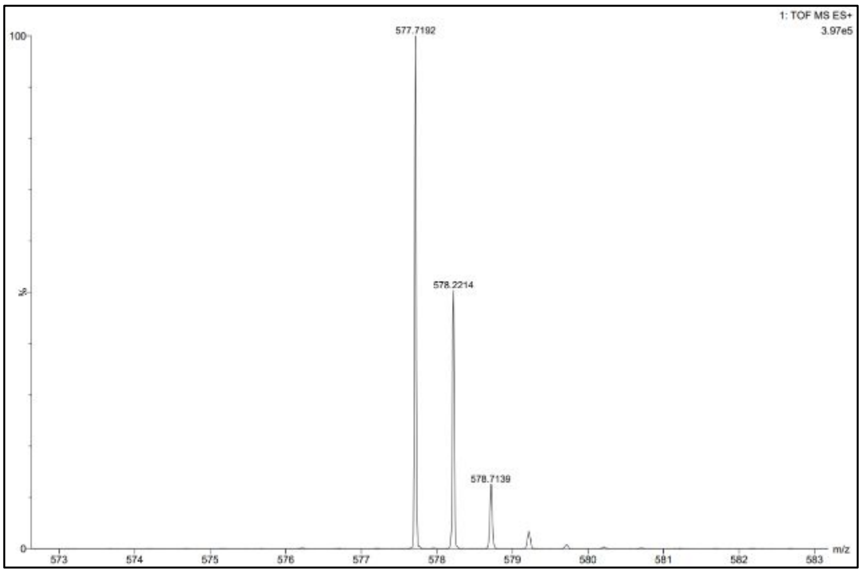
1: TOF MS ES+
3.97e5
577.7192
578.2214
578.7139
100
%
0
573
574
575
576
577
578
579
580
581
582
583
m/z

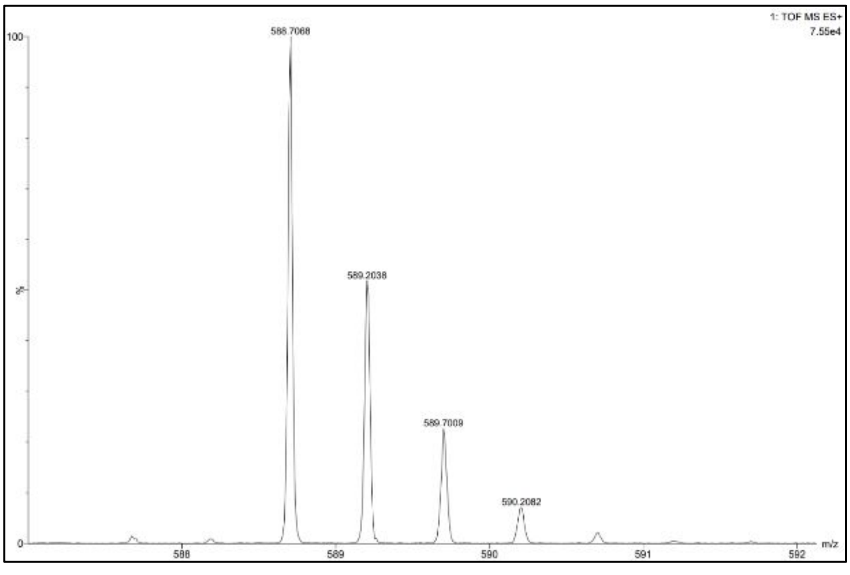
1: TOF MS ES+
7.55e4
588.7068
589.2038
589.7009
590.2082
100
%
0
588
589
590
591
592
m/z

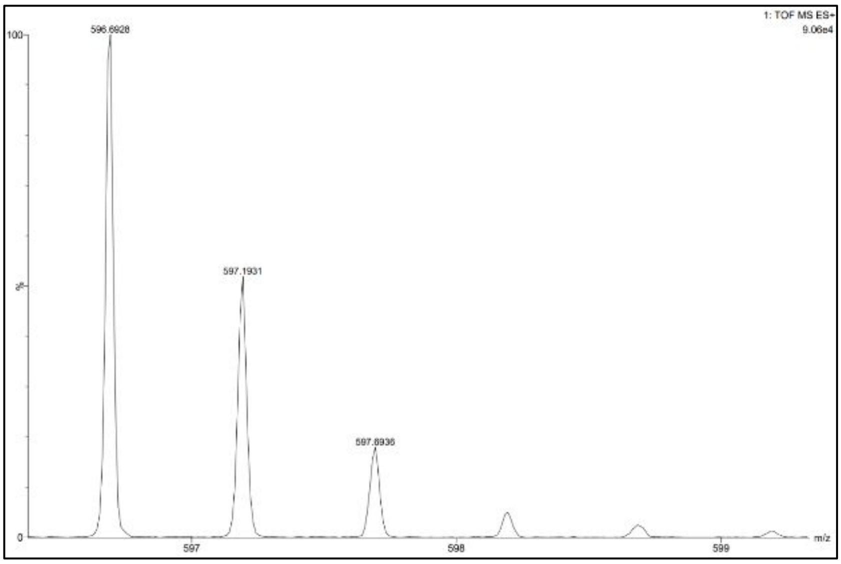

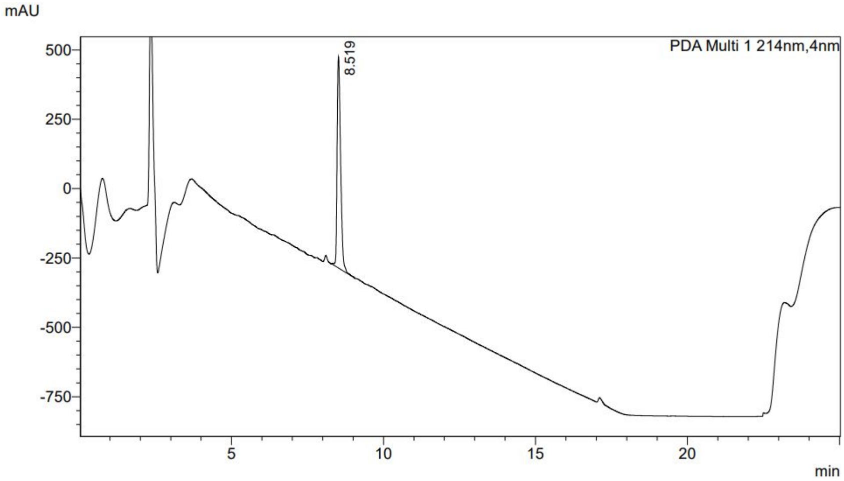

Analytical HPLC: purity = 100%

C1T-[C031]

Exact Mass:

**Expected $[M+H^+]^+$**	**Measured $[M+H^+]^+$**
1182.4586	1182.4556
**Expected $[M+Na^+]^+$**	**Measured $[M+Na^+]^+$**
1204.4405	1204.4396
**Expected $[M+2H^+]^{2+}$**	**Measured $[M+2H^+]^{2+}$**
591.7330	591.7324
**Expected $[M+Na^++H^+]^{2+}$**	**Measured $[M+Na^++H^+]^{2+}$**
602.7239	602.7205
**Expected $[M+K^++H^+]^{2+}$**	**Measured $[M+K^++H^+]^{2+}$**
610.7109	610.7092

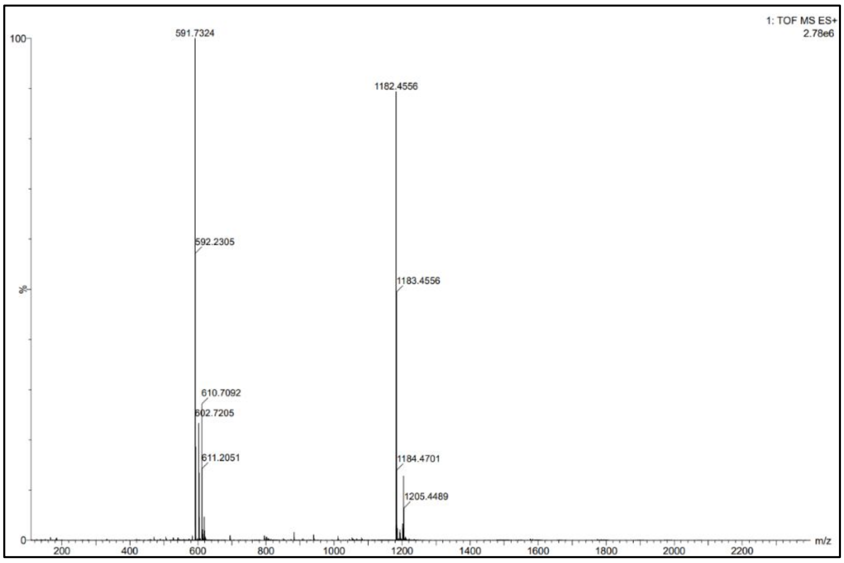

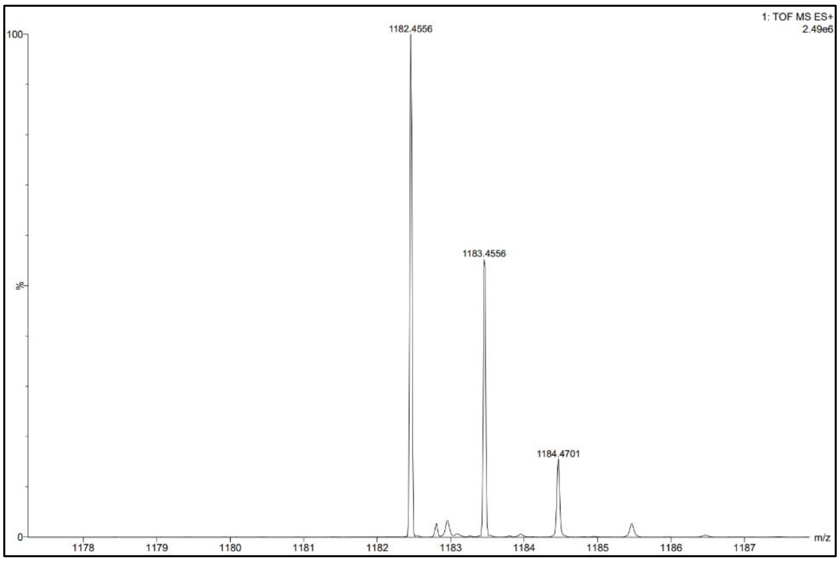
1: TOF MS ES+
2.49e6
1182.4556
1183.4556
1184.4701
100
%
0
1178
1179
1180
1181
1182
1183
1184
1185
1186
1187
m/z

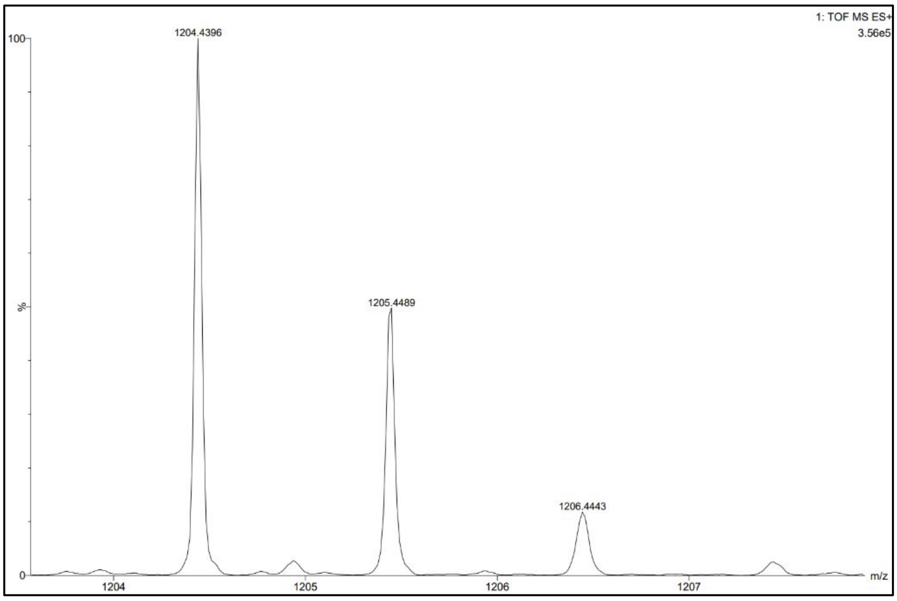
1: TOF MS ES+
3.56e5
1204.4396
1205.4489
1206.4443
100
%
0
1204
1205
1206
1207
m/z

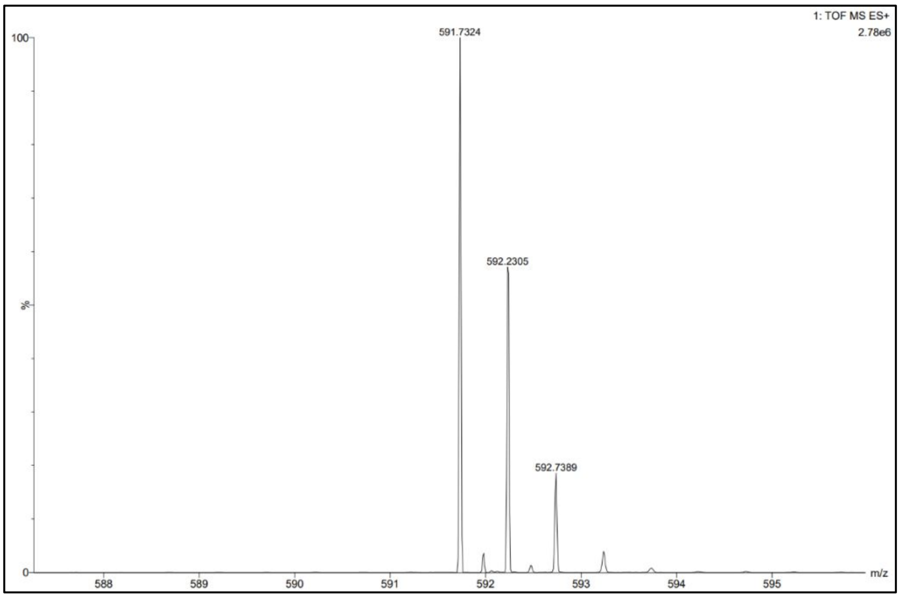
1: TOF MS ES+
2.78e6
591.7324
592.2305
592.7389
100
%
0
588
589
590
591
592
593
594
595
m/z

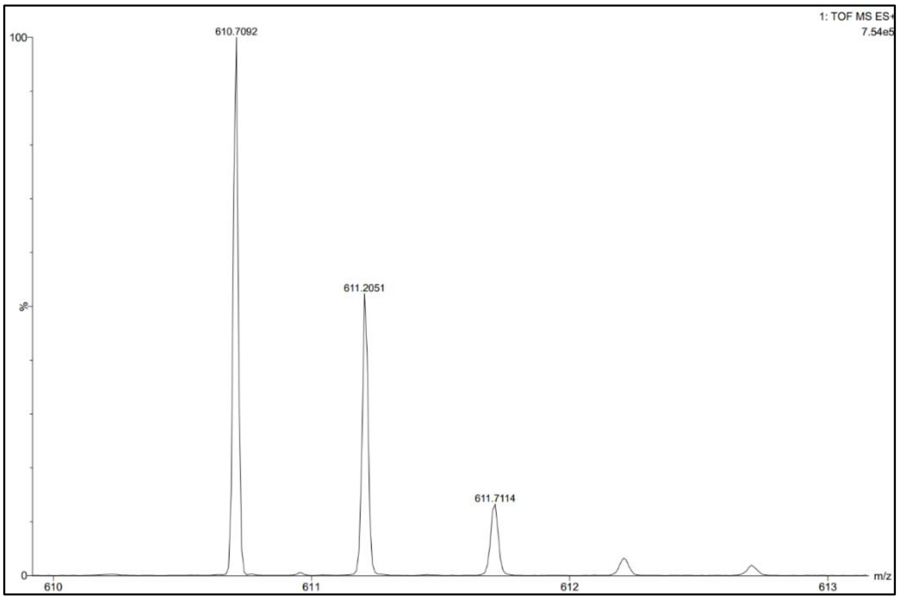

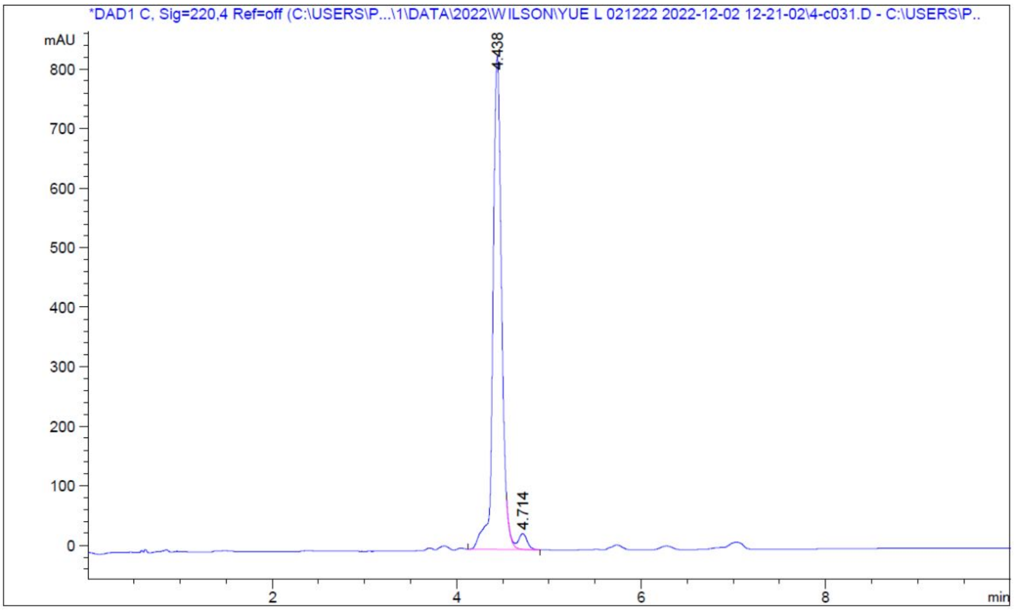

Analytical HPLC: purity = 99%

C1T-[C088]

Exact Mass: 1203.2773

**Expected [M+H⁺]⁺**	**Measured [M+H⁺]⁺**
1204.2846	1204.2896
**Expected [M+2H⁺]²⁺**	**Measured [M+2H⁺]²⁺**
602.6459	602.6496

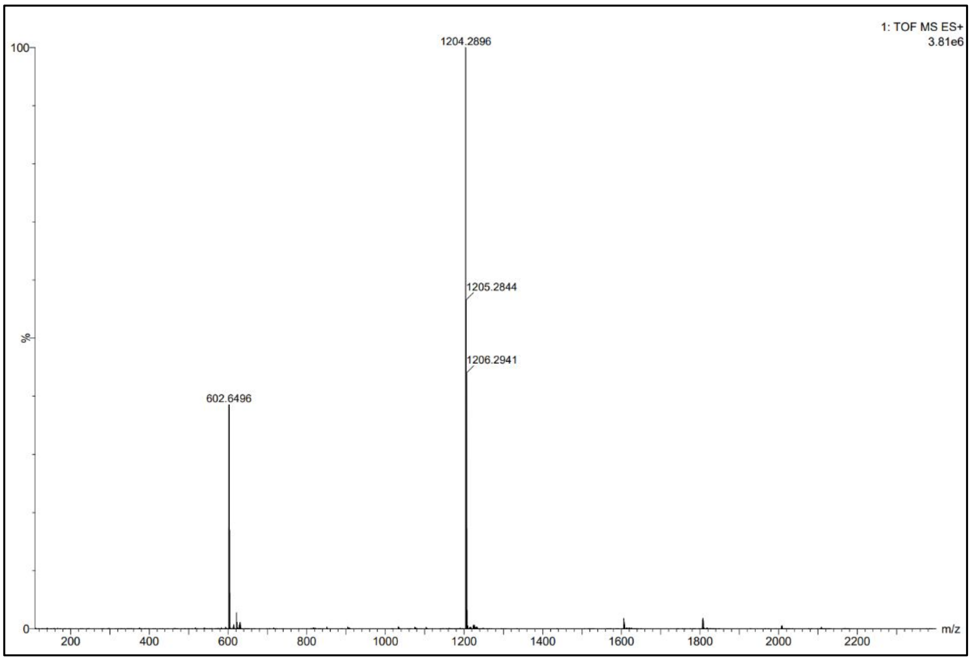

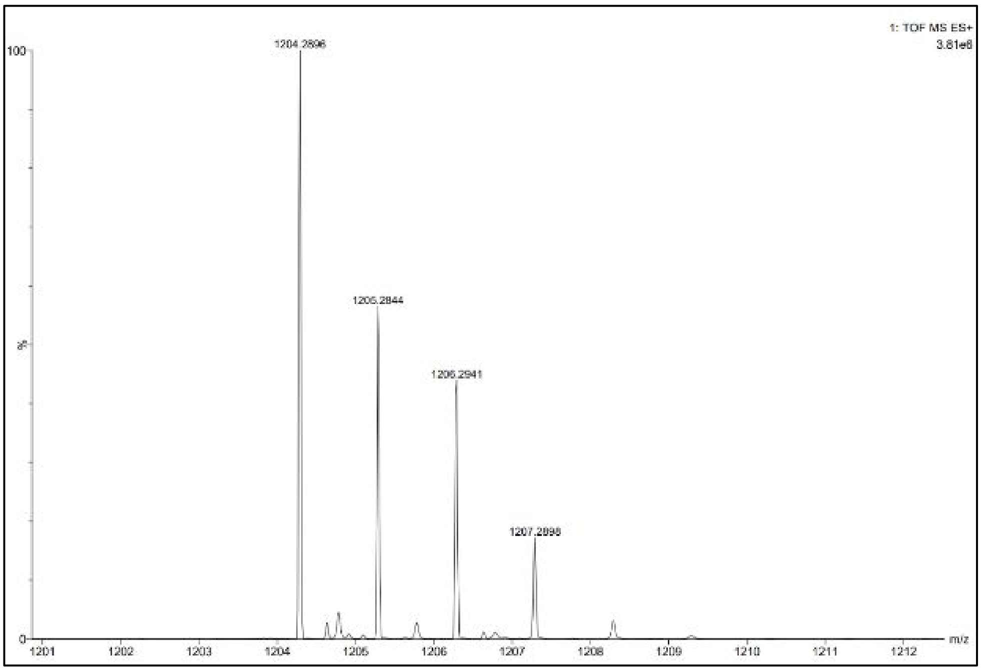

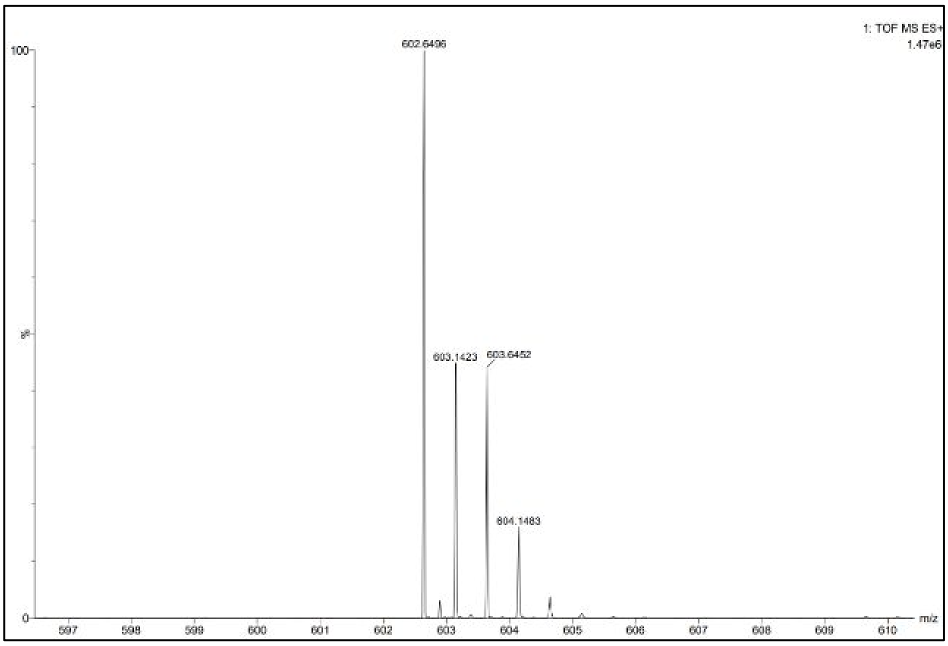

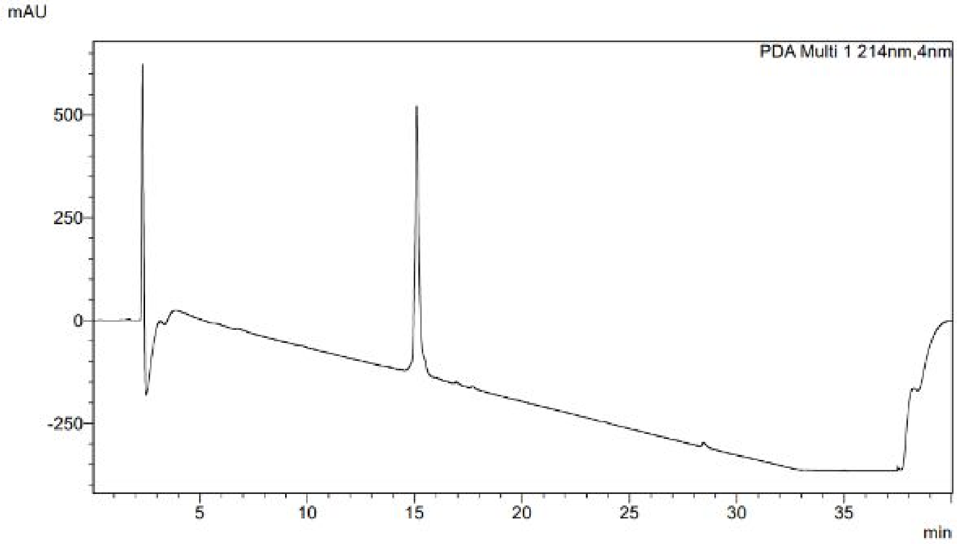

Analytical HPLC: purity = 100%

C1T-[C117]

Exact Mass: 1028.4199

Expected $[M+H^+]^+$	Measured $[M+H^+]^+$
1029.4272	1029.4202
**Expected $[M+2H^+]^{2+}$**	**Measured $[M+2H^+]^{2+}$**
515.2173	515.2122
**Expected $[M+Na^++H^+]^{2+}$**	**Measured $[M+Na^++H^+]^{2+}$**
526.2082	526.2017
**Expected $[M+K^++H^+]^{2+}$**	**Measured $[M+K^++H^+]^{2+}$**
534.1952	534.1885

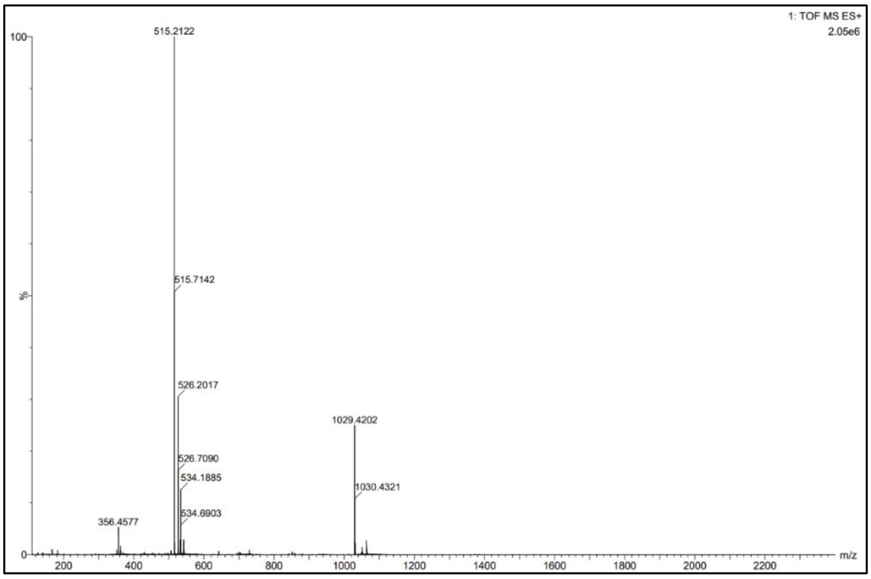

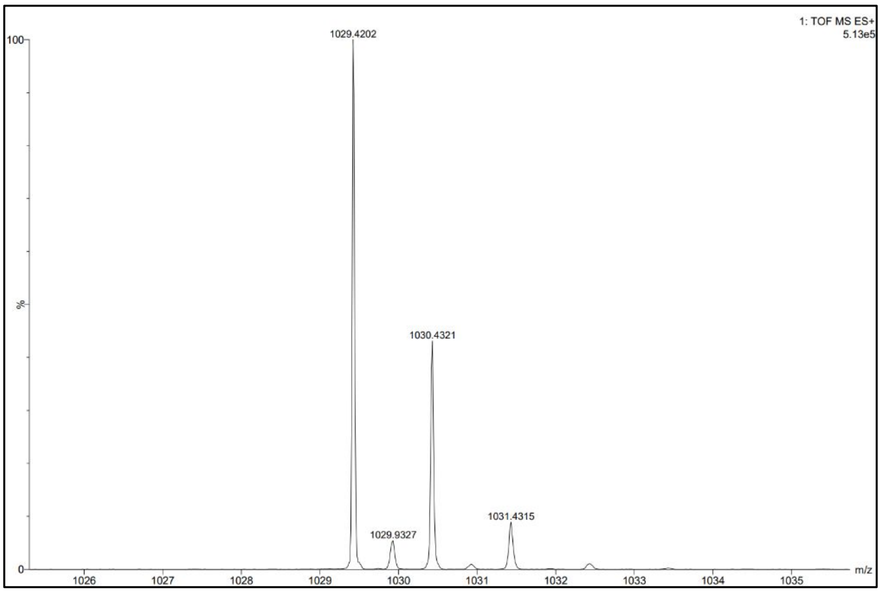

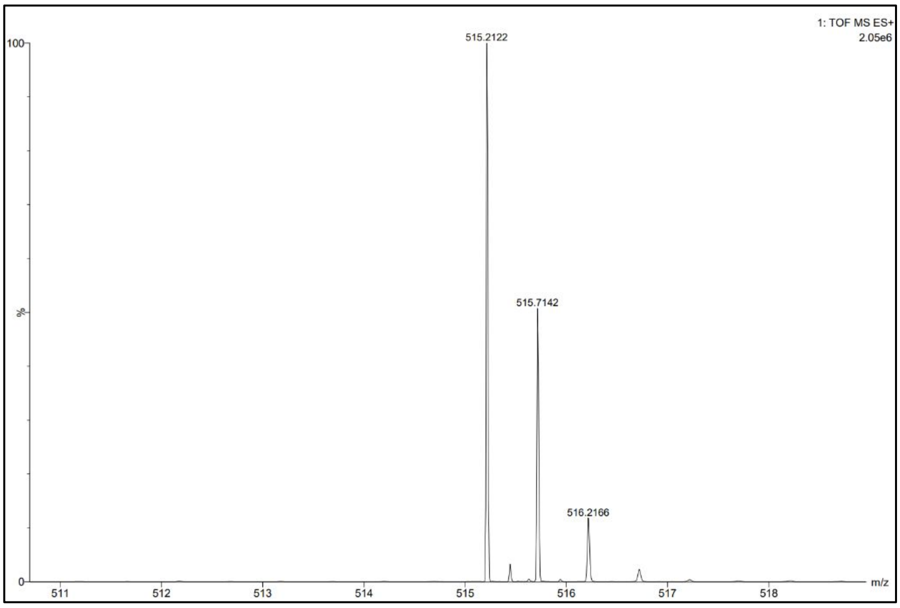
1: TOF MS ES+
2.05e6
515.2122
515.7142
516.2166
100
%
0
511
512
513
514
515
516
517
518
m/z

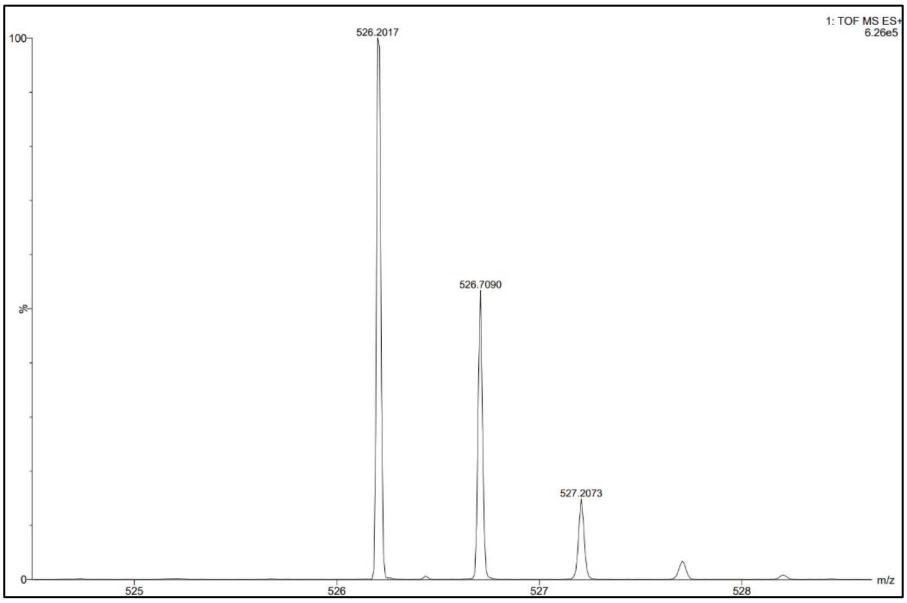
1: TOF MS ES+
6.26e5
526.2017
526.7090
527.2073
100
%
0
525
526
527
528
m/z

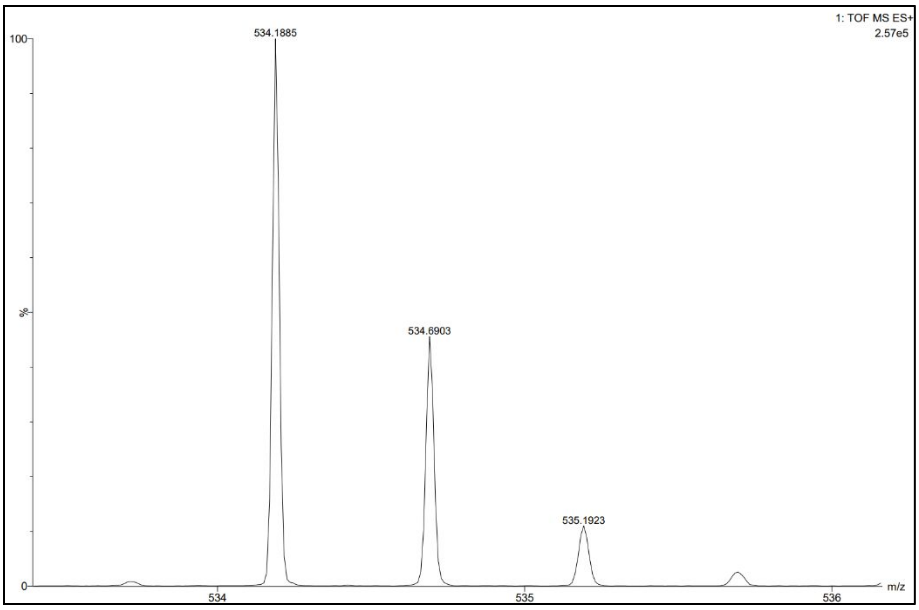

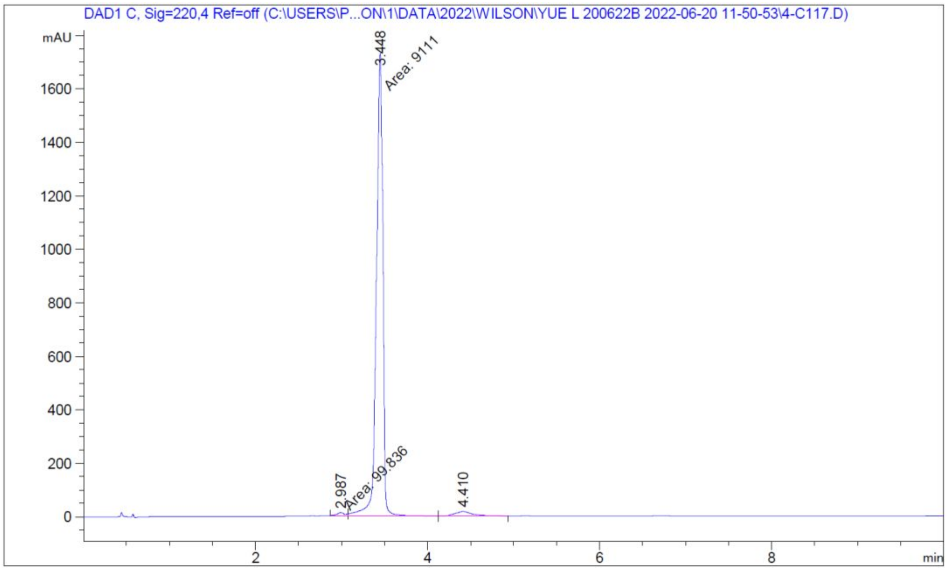

Analytical HPLC: purity = 100%

C1T-[C138]

Exact Mass: 1145.3414

Expected $[M+H^+]^+$	Measured $[M+H^+]^+$
1146.3486	1146.3518
**Expected $[M+2H^+]^{2+}$**	**Measured $[M+2H^+]^{2+}$**
573.6780	573.6808

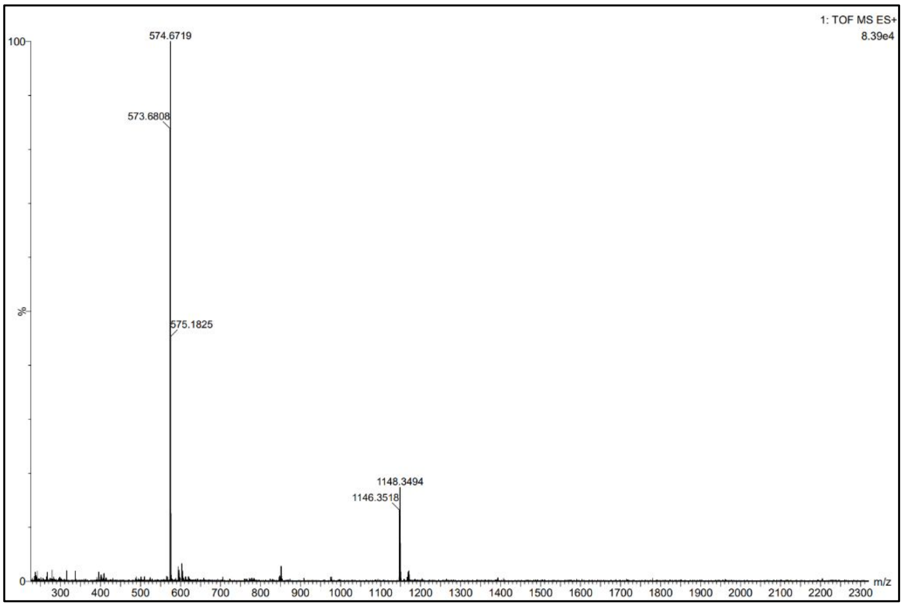

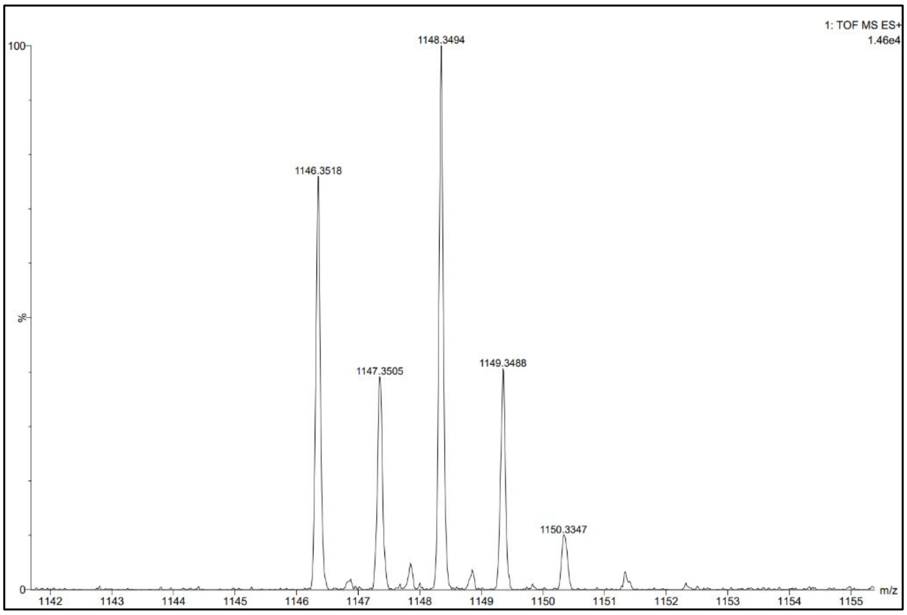

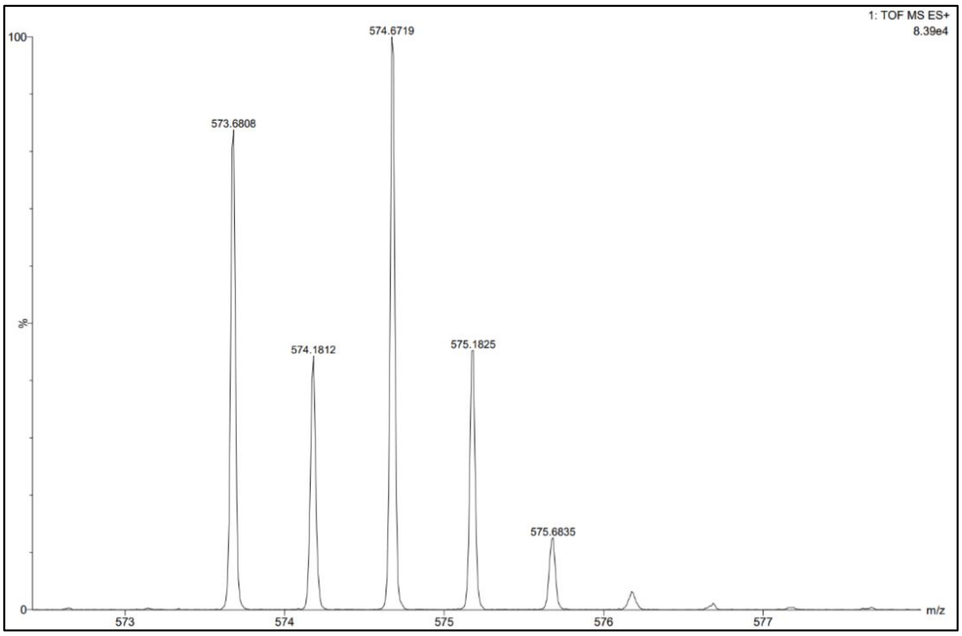

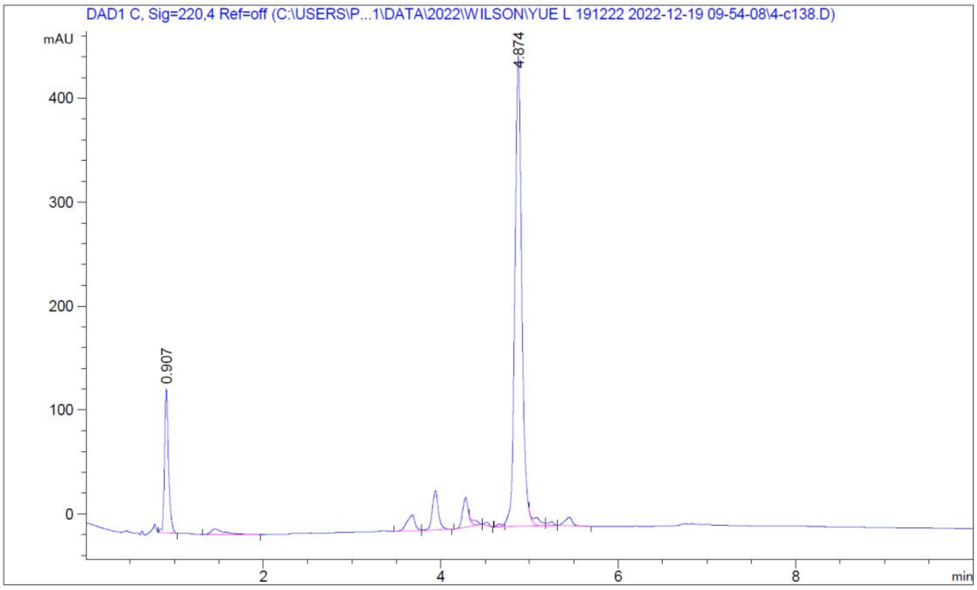

Analytical HPLC: purity = 86%

C2T-[C007]

Exact Mass: 1090.4244

Expected [M+H⁺]⁺	Measured [M+H⁺]⁺
1091.4316	1091.4363
**Expected $[M+2H^+]^{2+}$**	**Measured $[M+2H^+]^{2+}$**
546.2195	546.2249

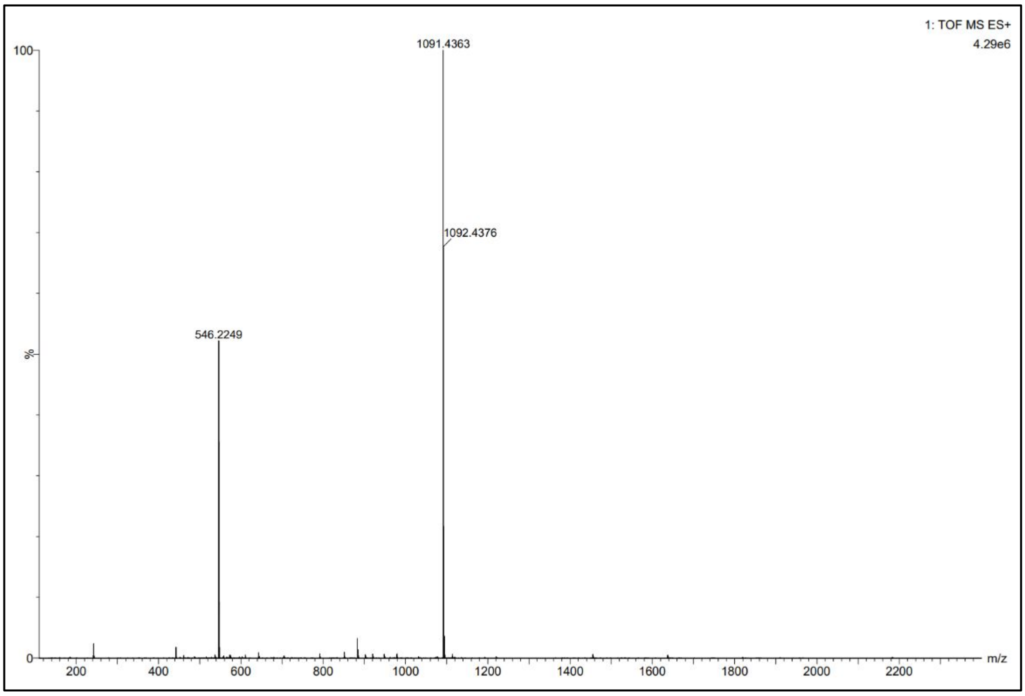

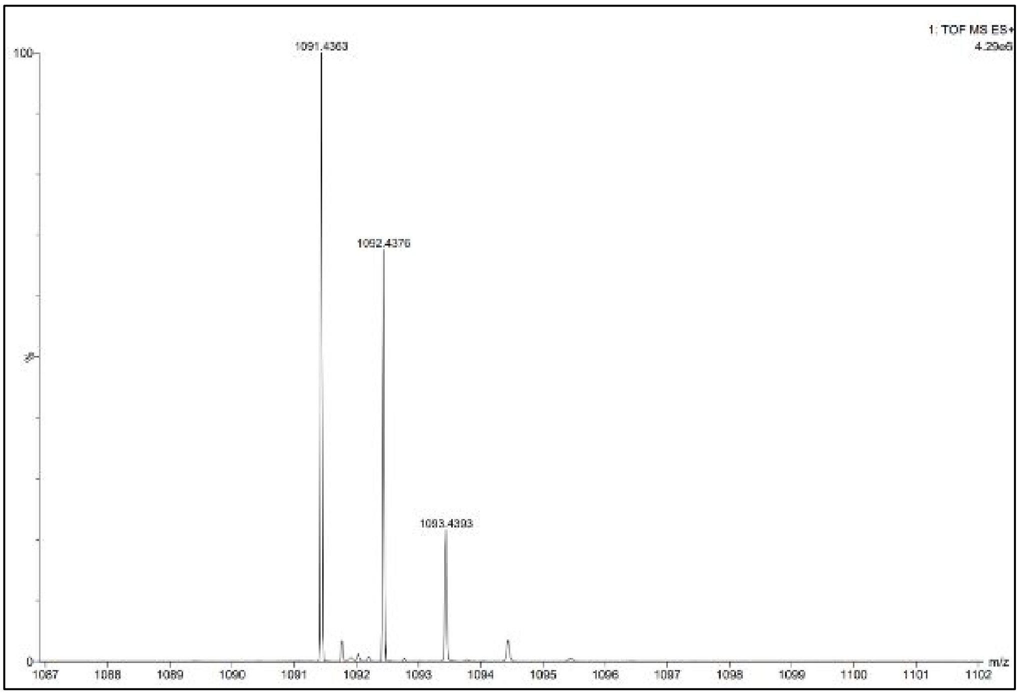

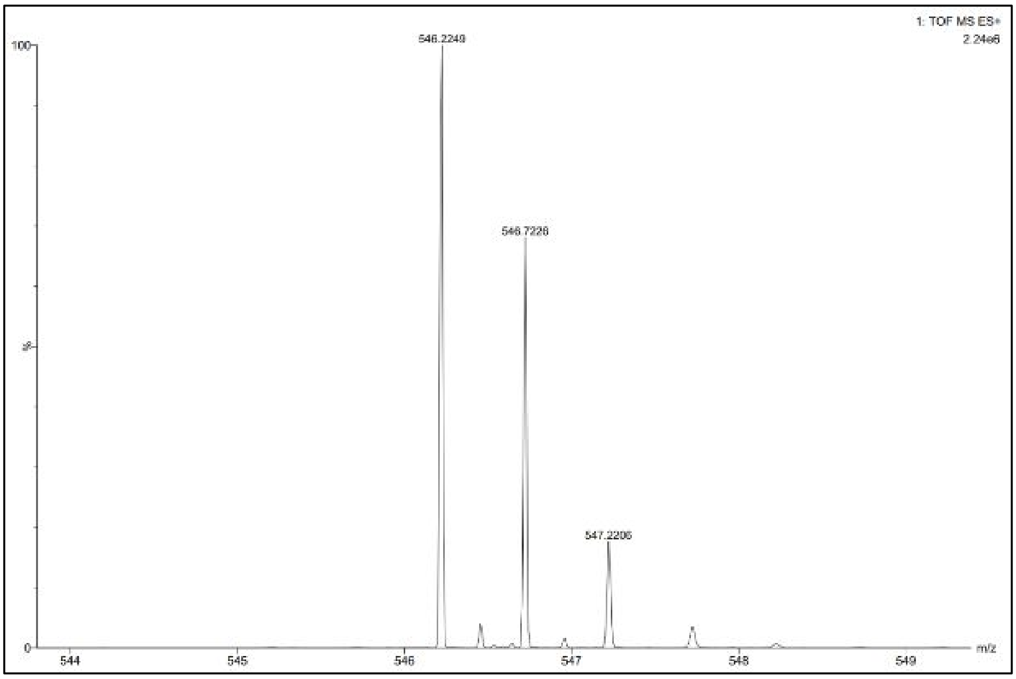

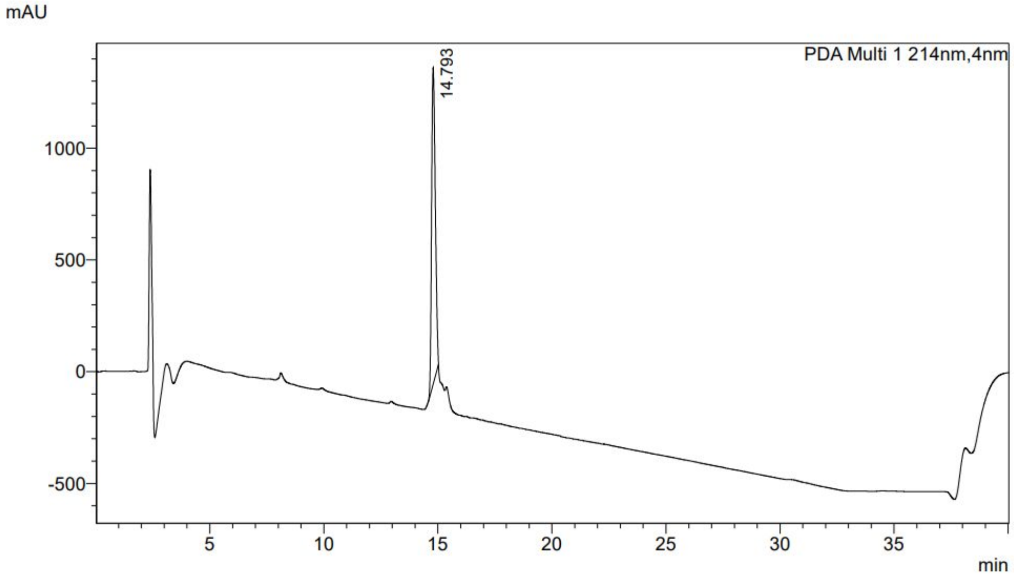

Analytical HPLC: purity = 99%

C2T-[C012]

Exact Mass: 1048.3541

**Expected [M+H⁺]⁺**	**Measured [M+H⁺]⁺**
1049.3614	1049.3582
**Expected [M+2H⁺]²⁺**	**Measured [M+2H⁺]²⁺**
525.1843	525.1823
**Expected [M+Na⁺]⁺**	**Measured [M+Na⁺]⁺**
1071.3433	1071.3467
**Expected [M+H⁺+K⁺]²⁺**	**Measured [M+H⁺+K⁺]²⁺**
544.1623	544.1573

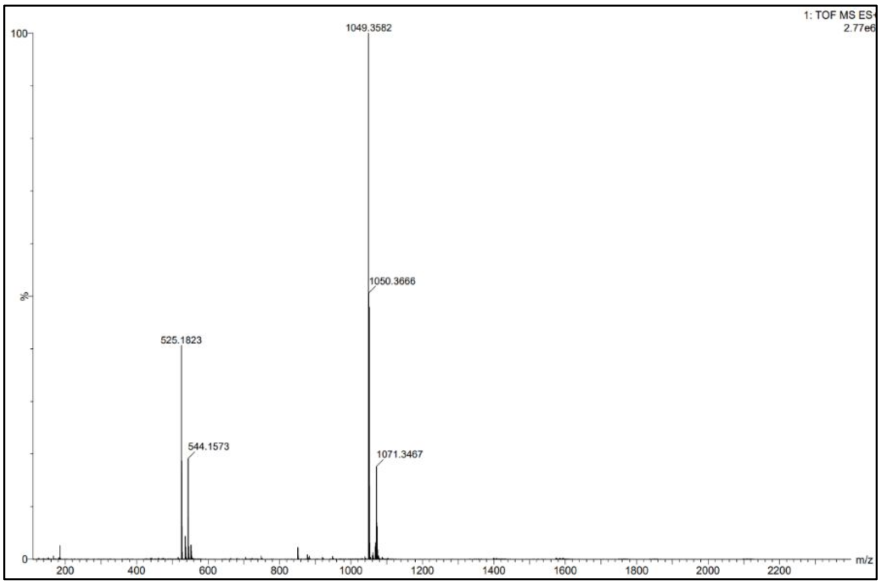

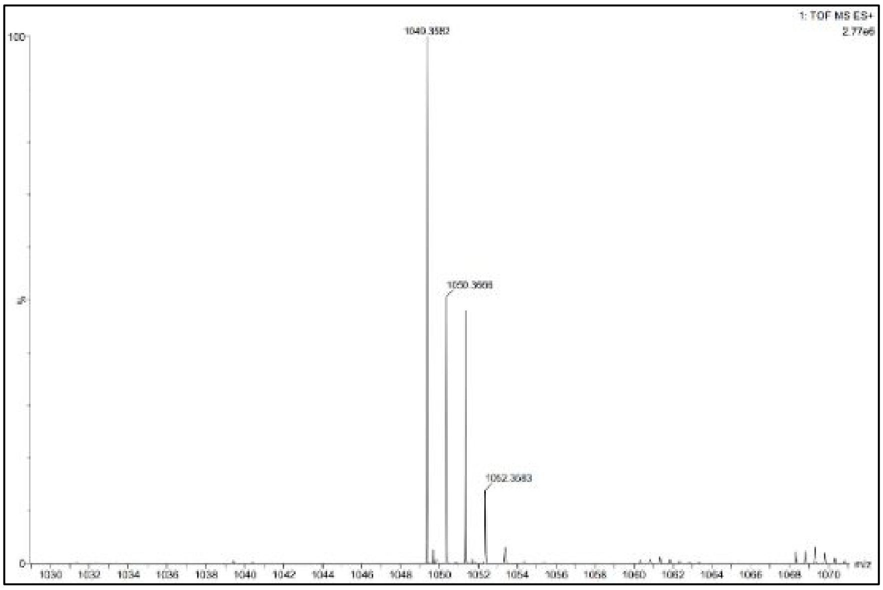

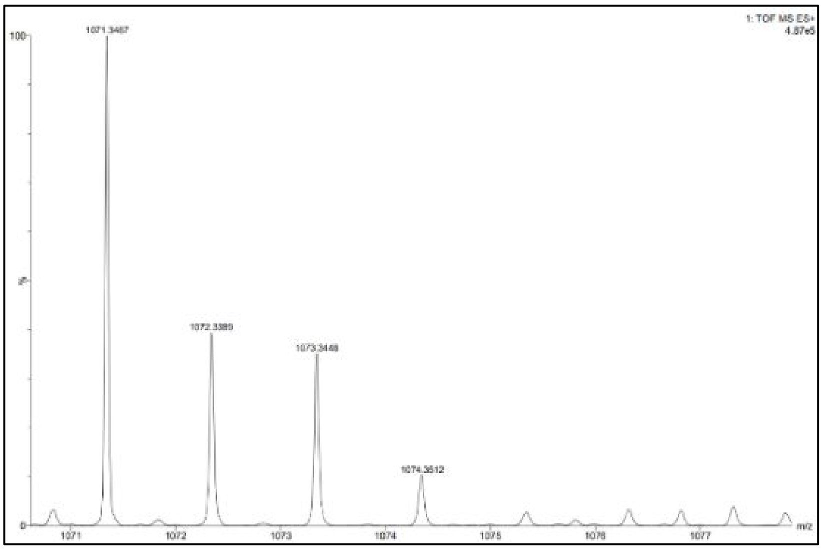
1: TOF MS ES+
4.87e5
100
%
0
1071.3467
1072.3389
1073.3448
1074.3512
1071
1072
1073
1074
1075
1076
1077
m/z

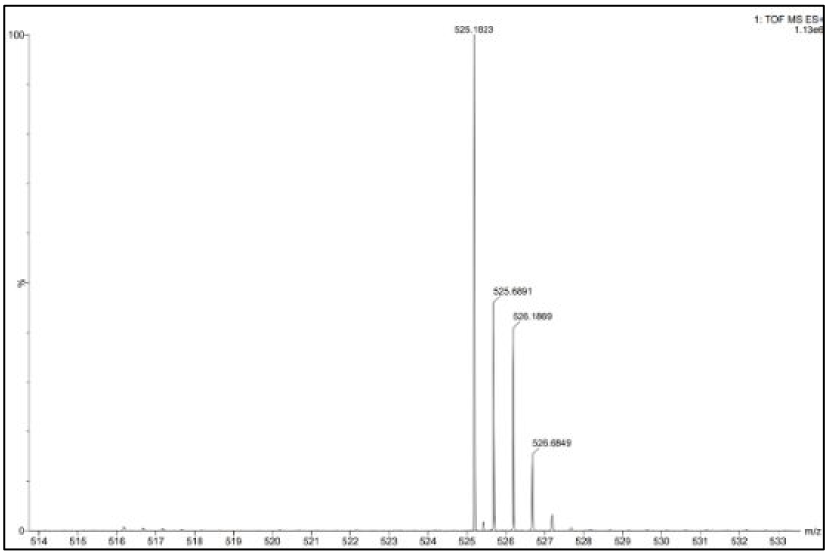
1: TOF MS ES+
1.13e6
100
%
0
525.1823
525.6891
526.1869
526.6849
514
515
516
517
518
519
520
521
522
523
524
525
526
527
528
529
530
531
532
533
m/z

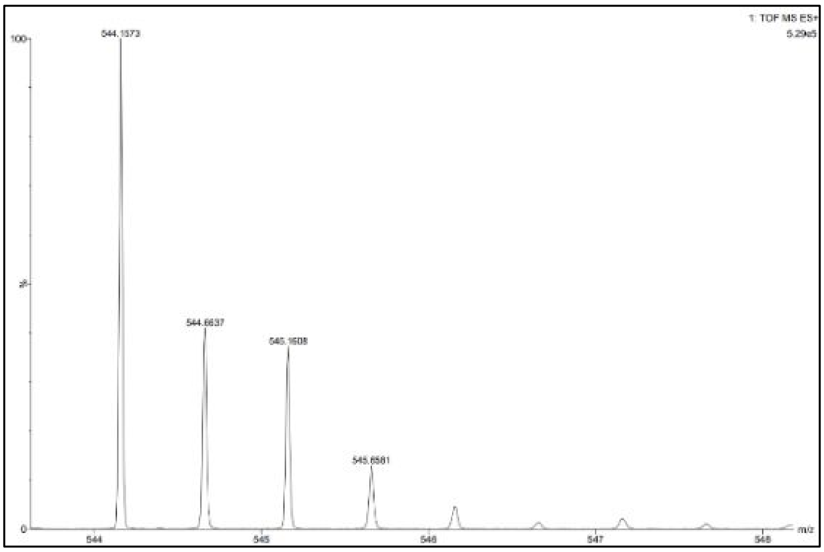
1: TOF MS ES+
5.29e5
100
%
0
544.1573
544.6637
545.1608
545.6581
544
545
546
547
548
m/z

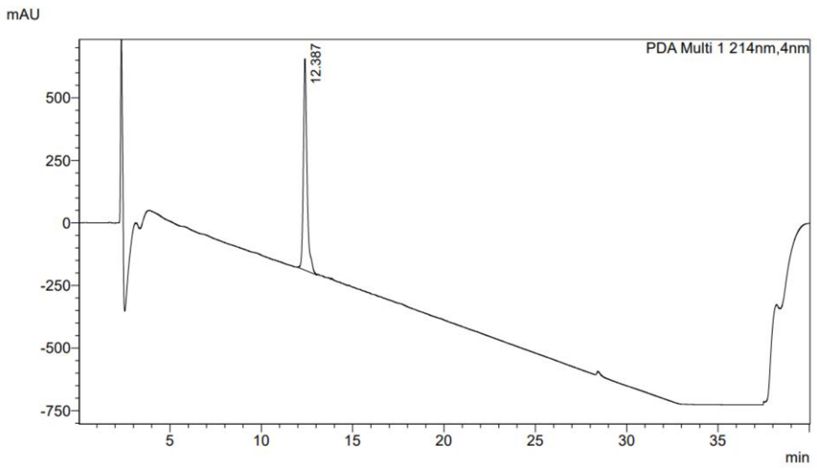

Analytical HPLC: purity = 100%

C2T-[C047]

Exact Mass: 1080.4400

**Expected $[M+H^+]^+$**	**Measured $[M+H^+]^+$**
1081.4473	1081.4497
**Expected $[M+Na^+]^+$**	**Measured $[M+Na^+]^+$**
1103.4292	1103.4313
**Expected $[M+2H^+]^{2+}$**	**Measured $[M+2H^+]^{2+}$**
541.2273	541.2252
**Expected $[M+Na^++H^+]^{2+}$**	**Measured $[M+Na^++H^+]^{2+}$**
552.2183	552.2178
**Expected $[M+K^++H^+]^{2+}$**	**Measured $[M+K^++H^+]^{2+}$**
560.2052	5060.1954

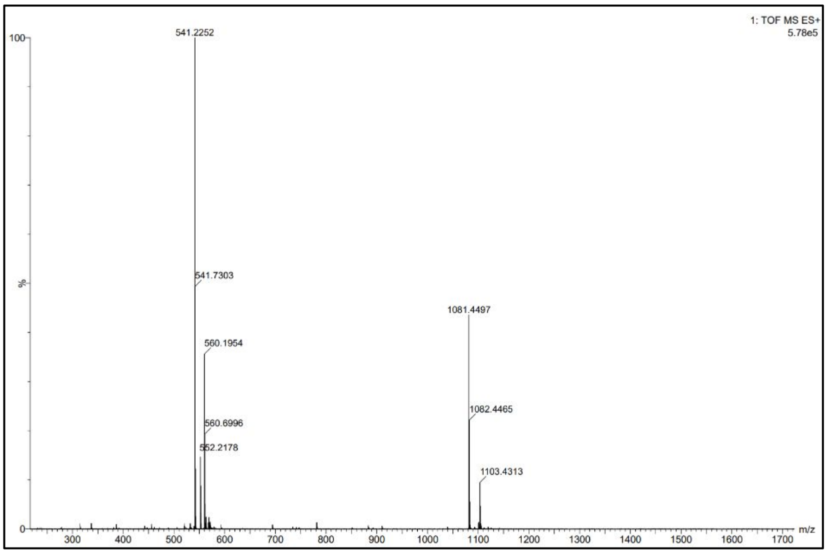

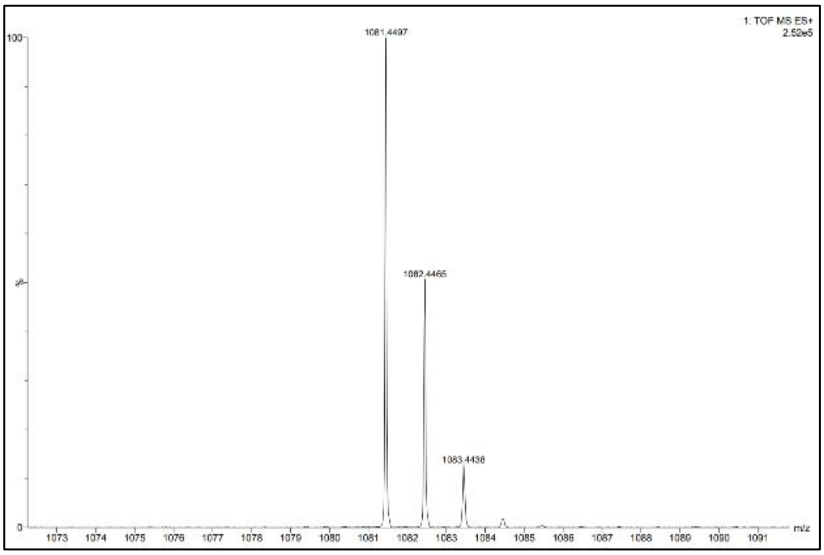
1: TOF MS ES+
2.52e5
1081.4497
1082.4465
1083.4438
m/z

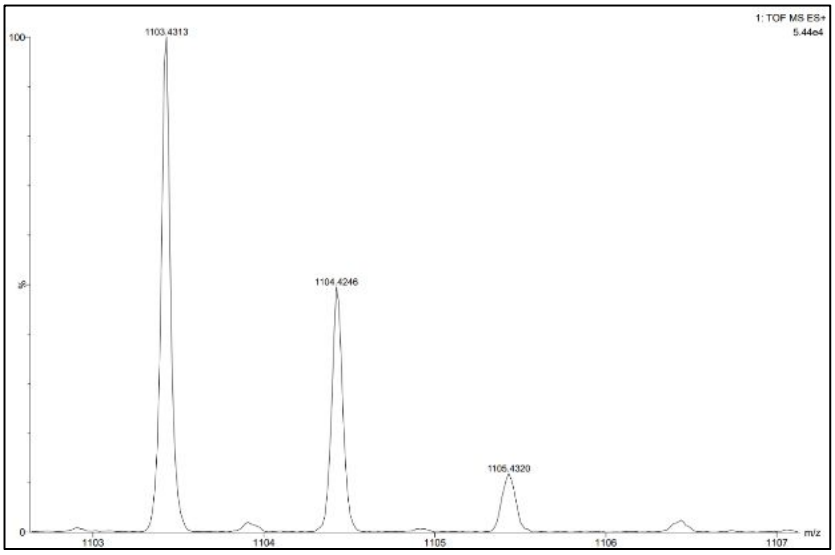
1: TOF MS ES+
5.44e4
1103.4313
1104.4246
1105.4320
m/z

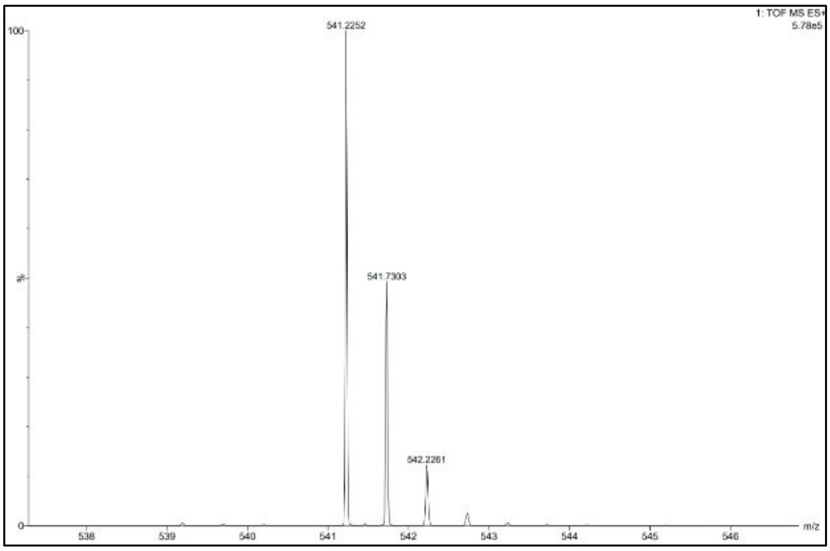
1: TOF MS ES+
5.78e5
541.2252
541.7303
542.2261
m/z

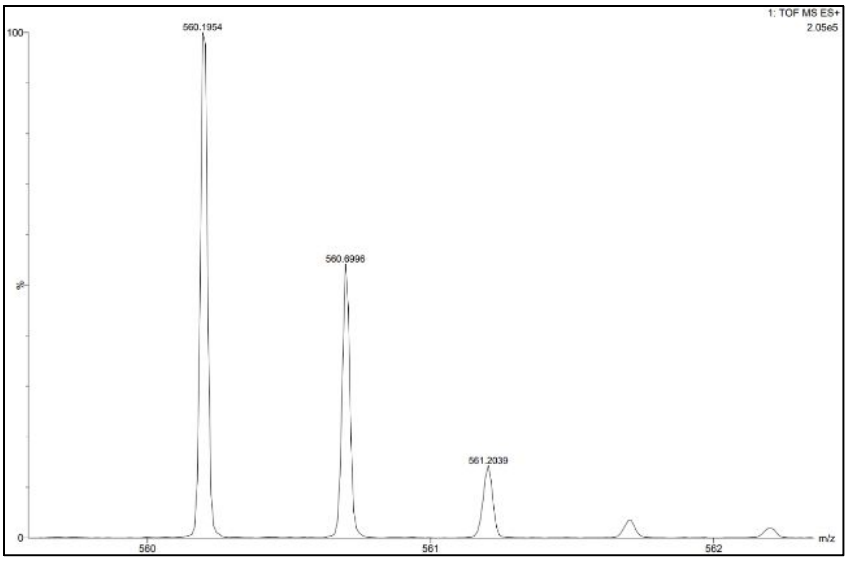

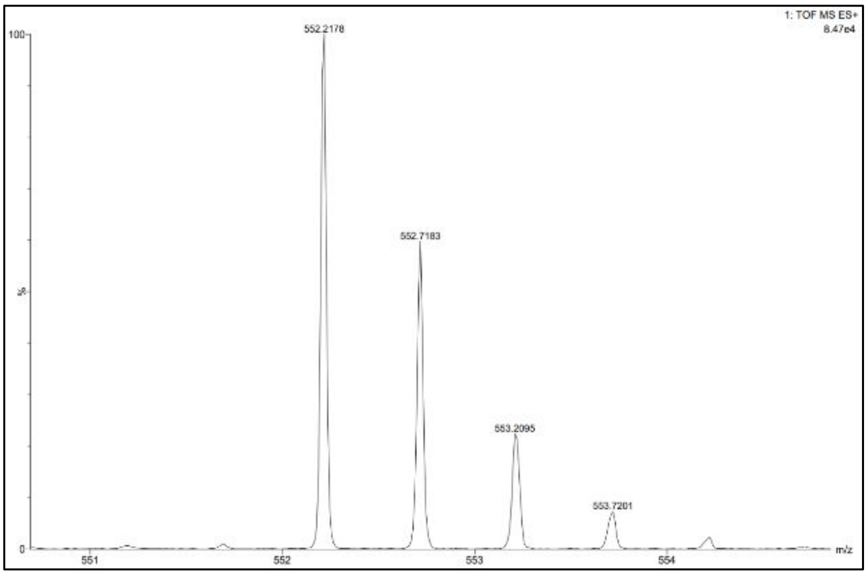

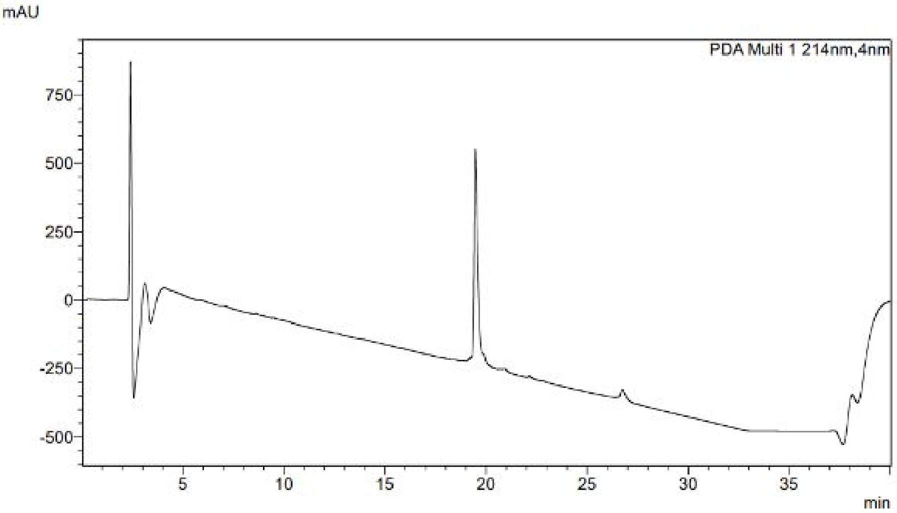

Analytical HPLC: purity = 100%

C2T-[C101]

Exact Mass: 1014.3931

**Expected $[M+H^+]^+$**	**Measured $[M+H^+]^+$**
1015.4003	1015.3960
**Expected $[M+Na^+]^+$**	**Measured $[M+Na^+]^+$**
1037.3823	1037.3849
**Expected $[M+2H^+]^{2+}$**	**Measured $[M+2H^+]^{2+}$**
508.2038	508.1995
**Expected $[M+Na^++H^+]^{2+}$**	**Measured $[M+Na^++H^+]^{2+}$**
519.1948	519.1890
**Expected $[M+K^++H^+]^{2+}$**	**Measured $[M+K^++H^+]^{2+}$**
527.1818	527.1791

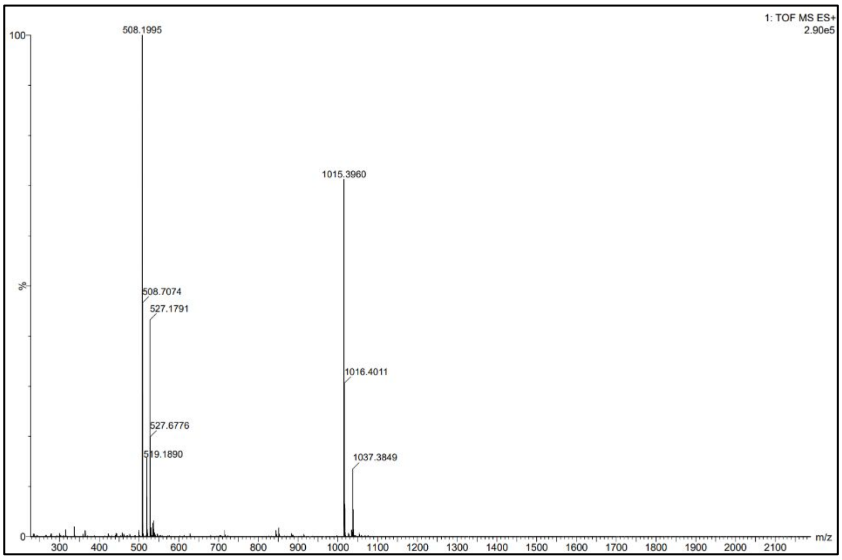

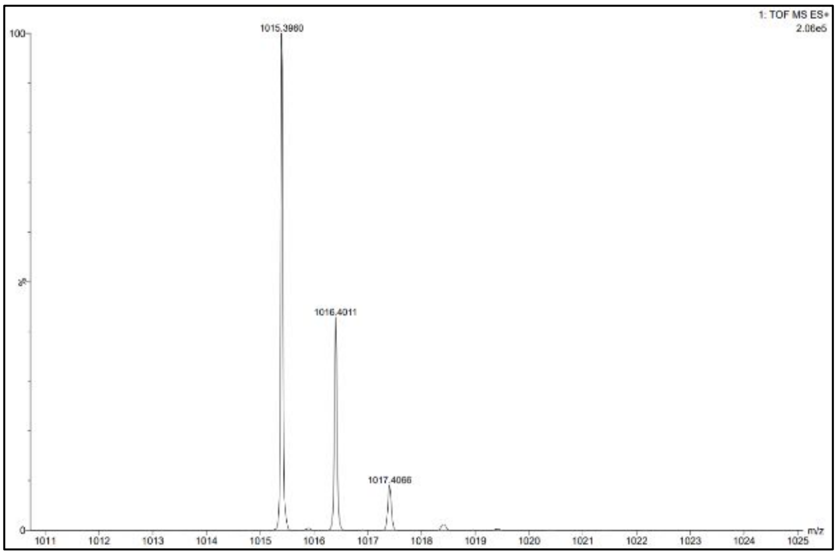
1: TOF MS ES+
2.06e5
100
%
0
1015.3960
1016.4011
1017.4066
1011 1012 1013 1014 1015 1016 1017 1018 1019 1020 1021 1022 1023 1024 1025
m/z

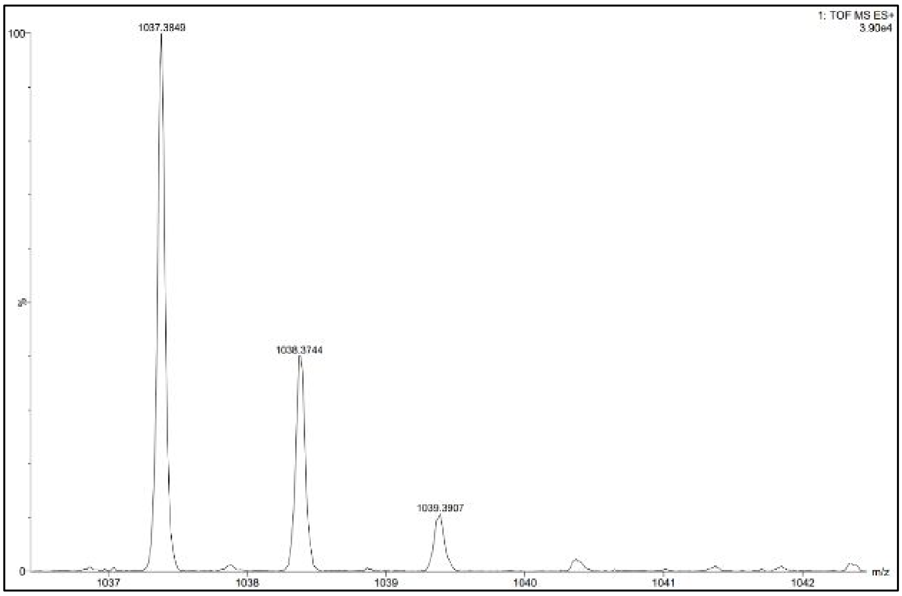
1: TOF MS ES+
3.90e4
100
%
0
1037.3849
1038.3744
1039.3907
1037 1038 1039 1040 1041 1042
m/z

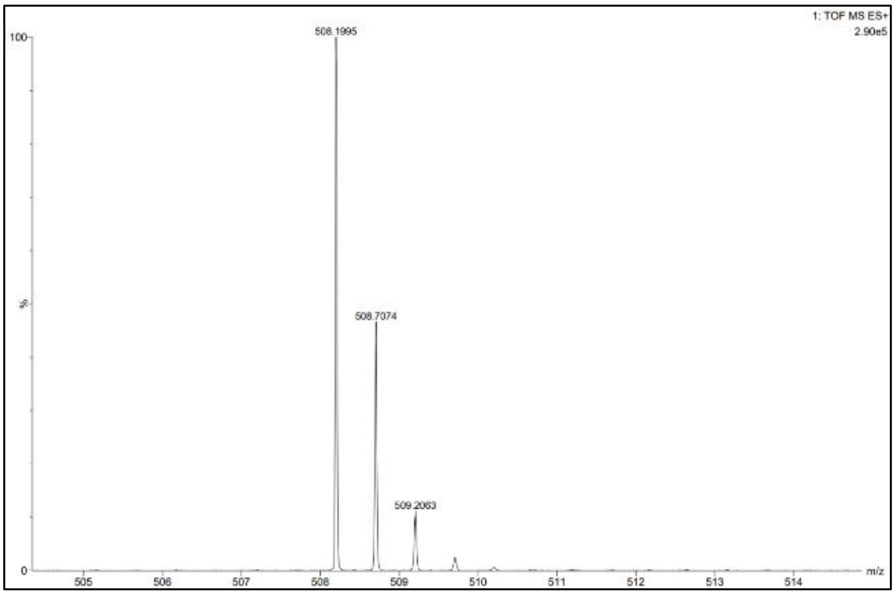
1: TOF MS ES+
2.90e5
100
%
0
508.1995
508.7074
509.2063
505 506 507 508 509 510 511 512 513 514
m/z

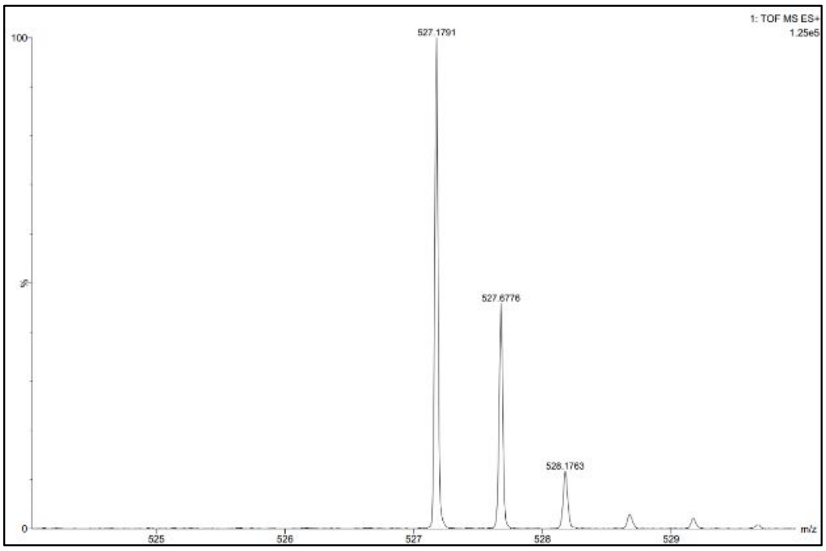

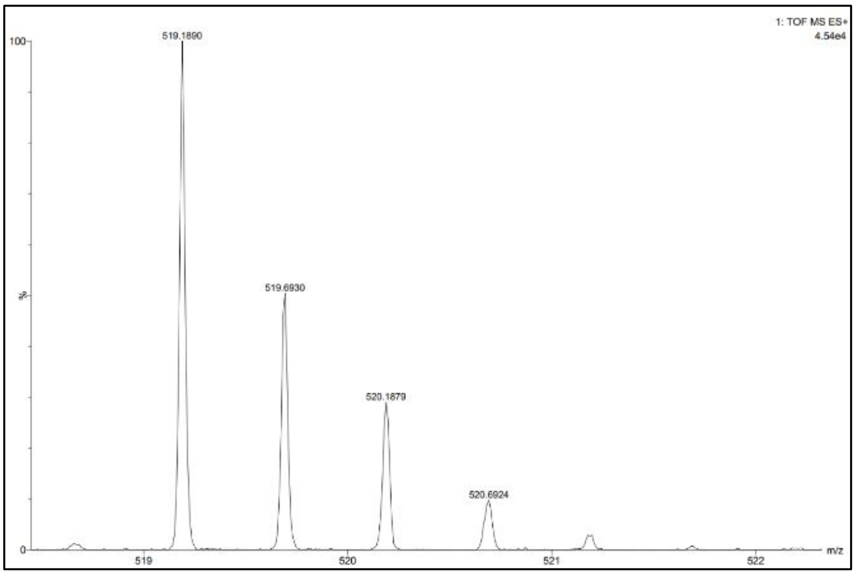

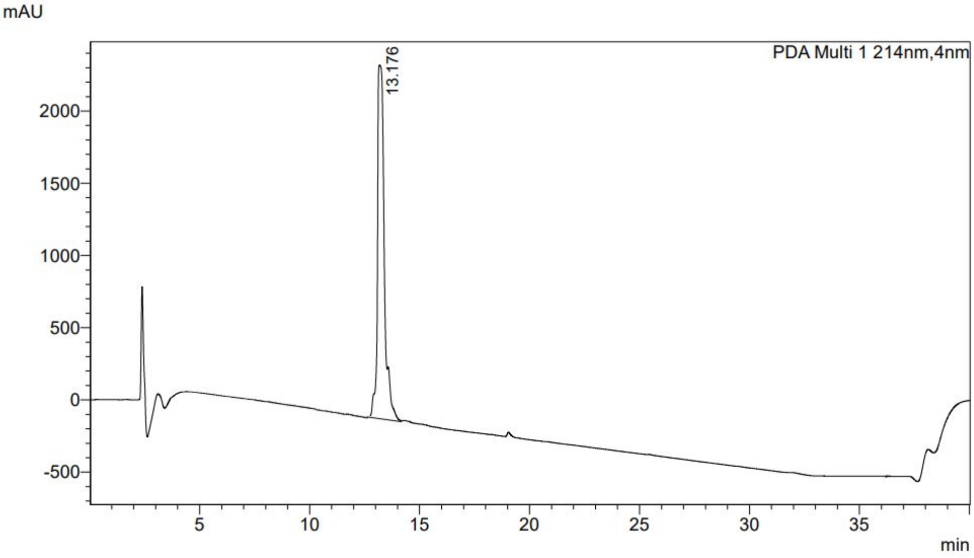

Analytical HPLC: purity = 100%

C3T-[C047]

Exact Mass: 952.3814

**Expected [M+H⁺]⁺**	**Measured [M+H⁺]⁺**
953.3887	953.3937
**Expected [M+2H⁺]²⁺**	**Measured [M+2H⁺]²⁺**
477.1980	477.1997

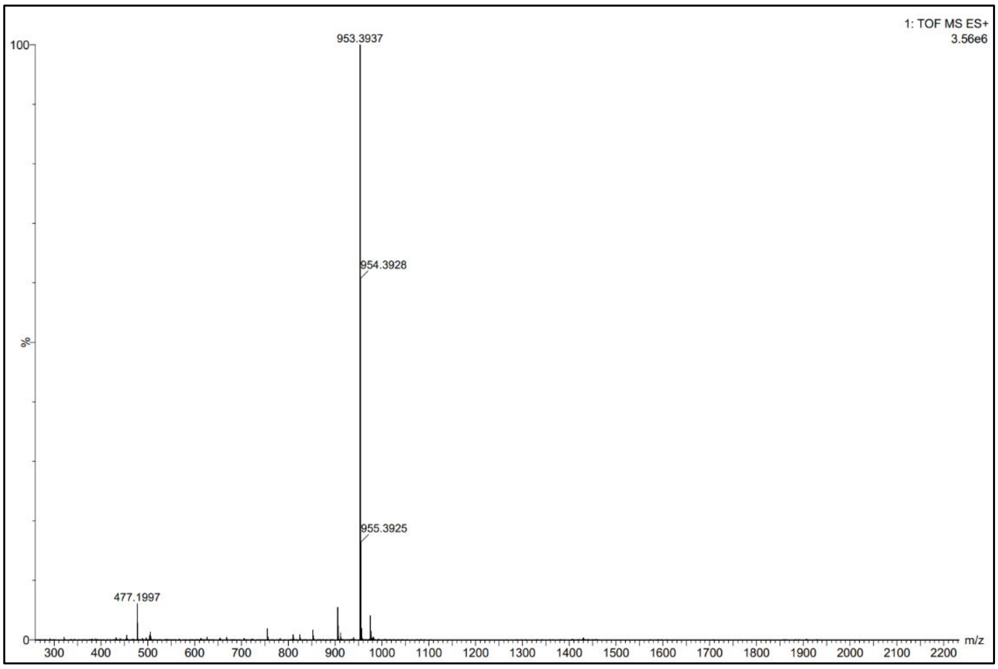

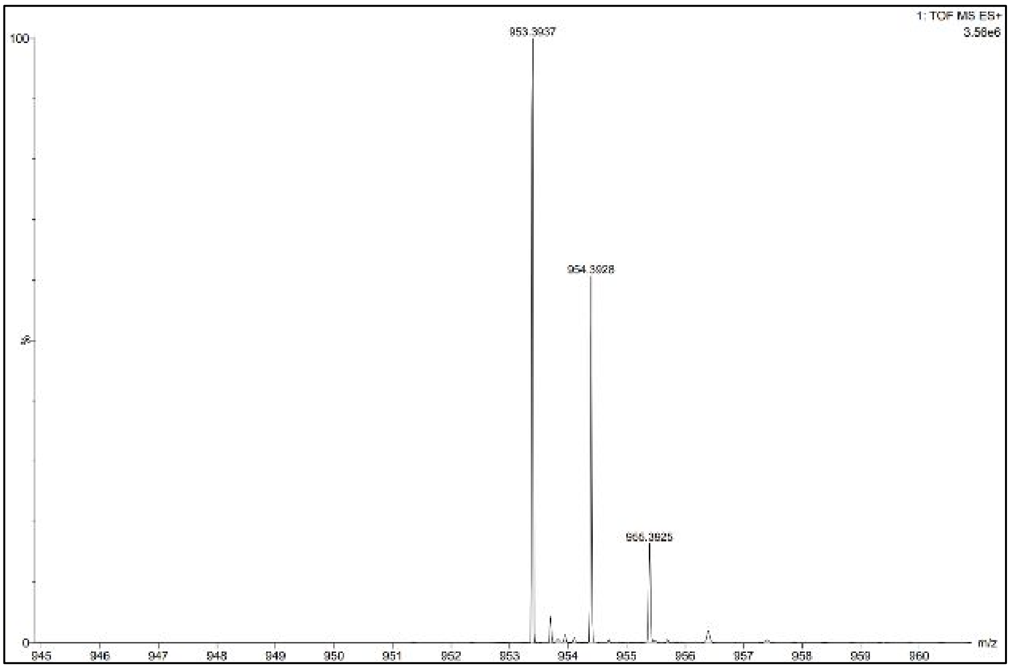

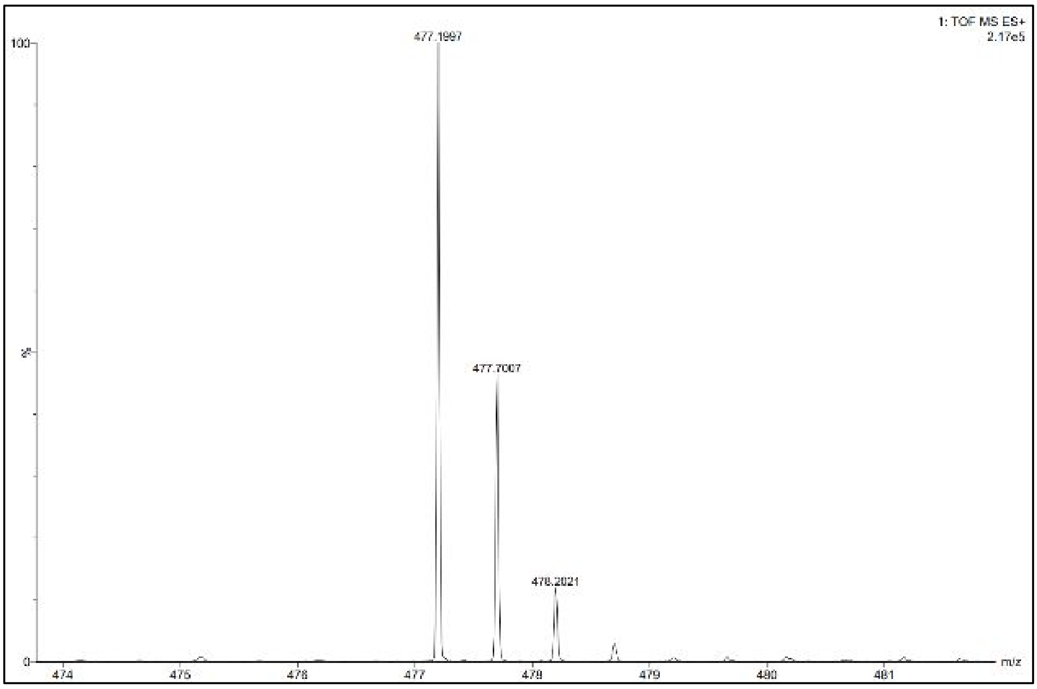

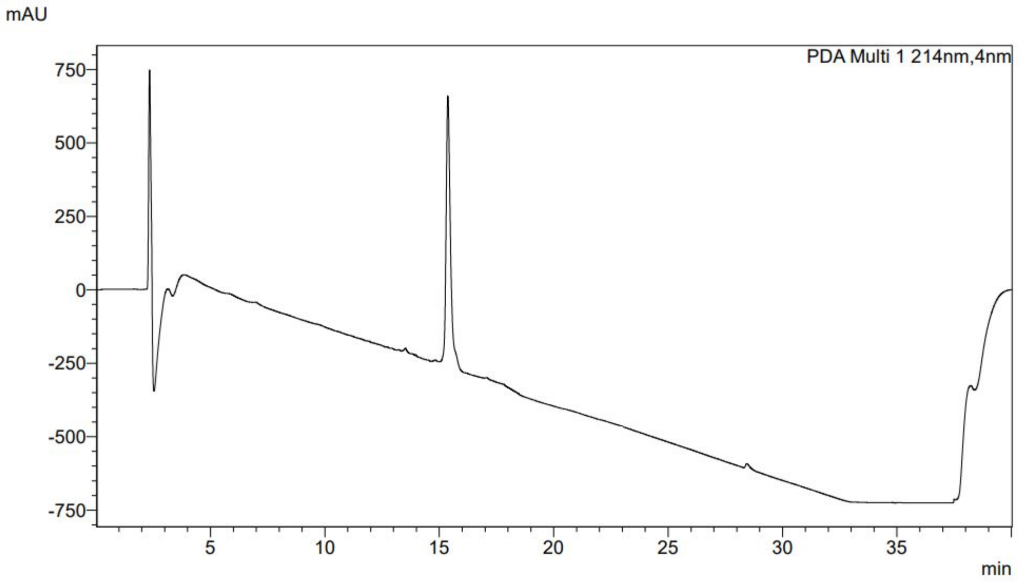

Analytical HPLC: purity = 100%

C2T-[N001]

Exact Mass: 1027.4247

**Expected [M+H⁺]⁺**	**Measured [M+H⁺]⁺**
1028.4320	1028.4348
**Expected [M+Na⁺]⁺**	**Measured [M+Na⁺]⁺**
514.7196	514.7200

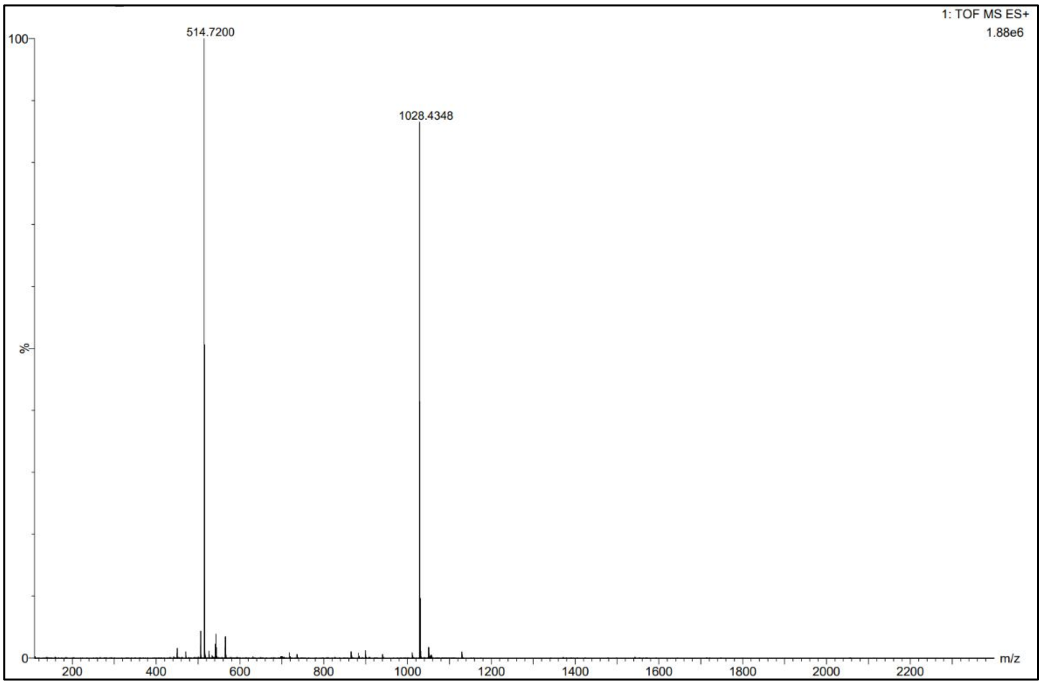

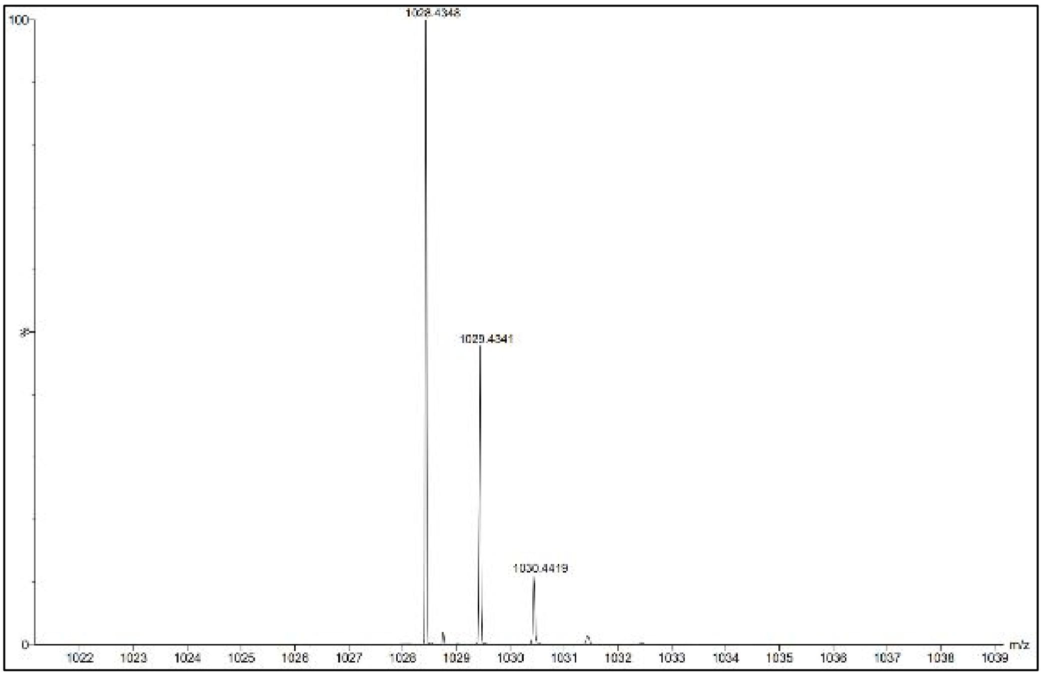

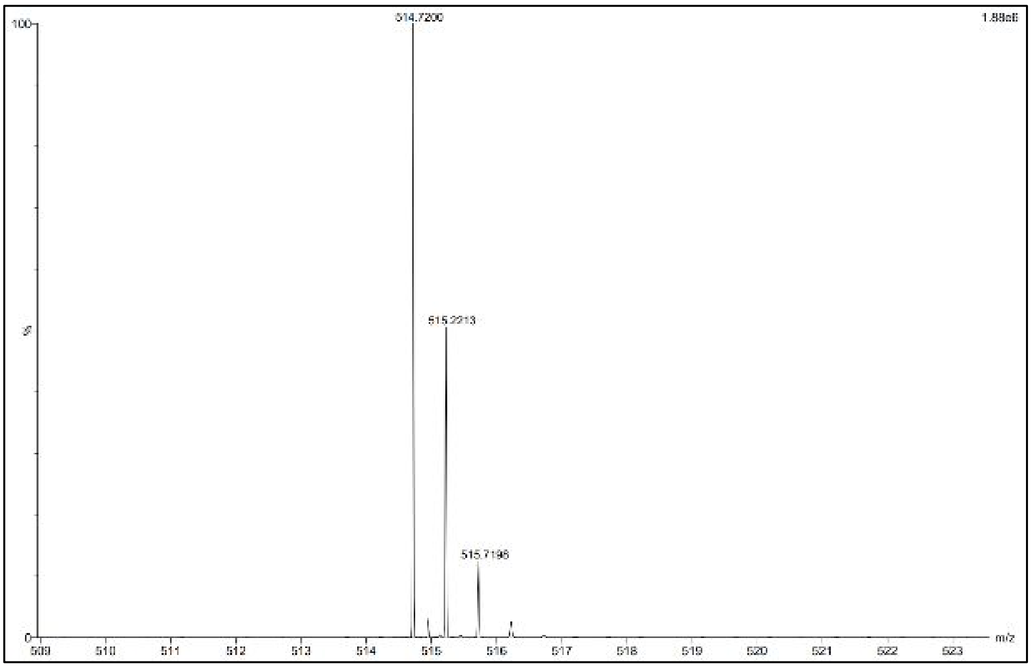

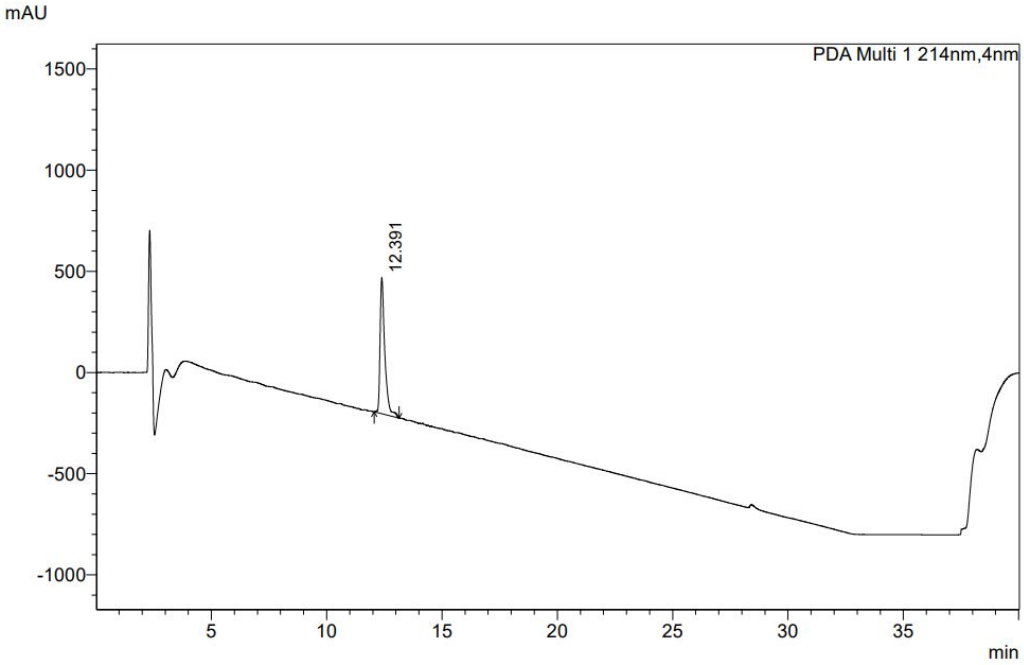

Analytical HPLC: purity = 100%

N1T-[N001]

Exact Mass: 1052.4563

**Expected [M+H⁺]⁺**	**Measured [M+H⁺]⁺**
1053.4636	1053.4744
**Expected [M+Na⁺]⁺**	**Measured [M+Na⁺]⁺**
527.2355	527.2427

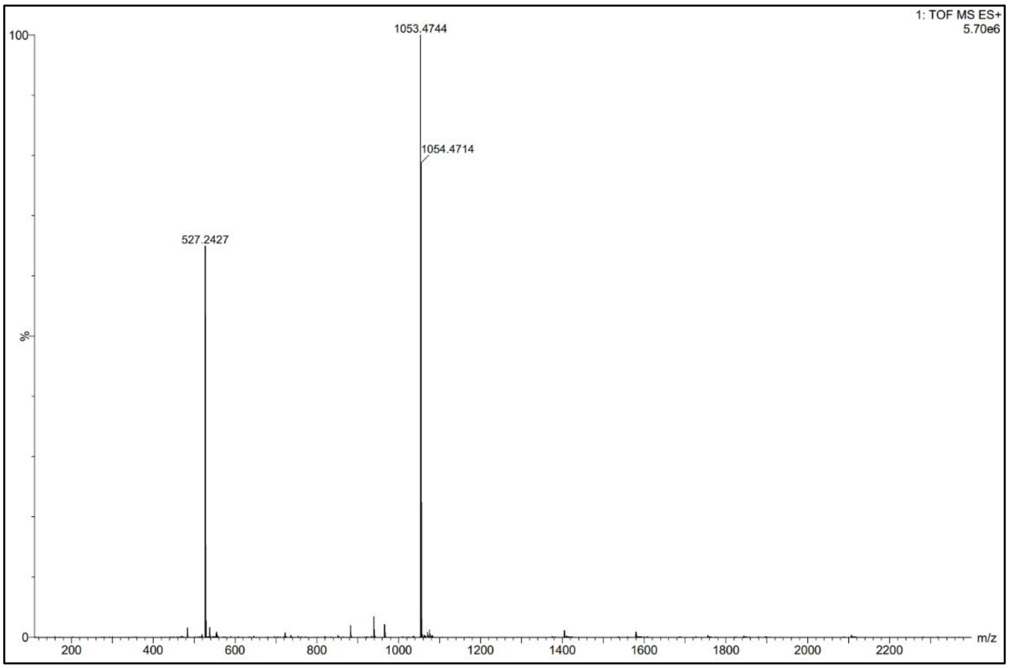

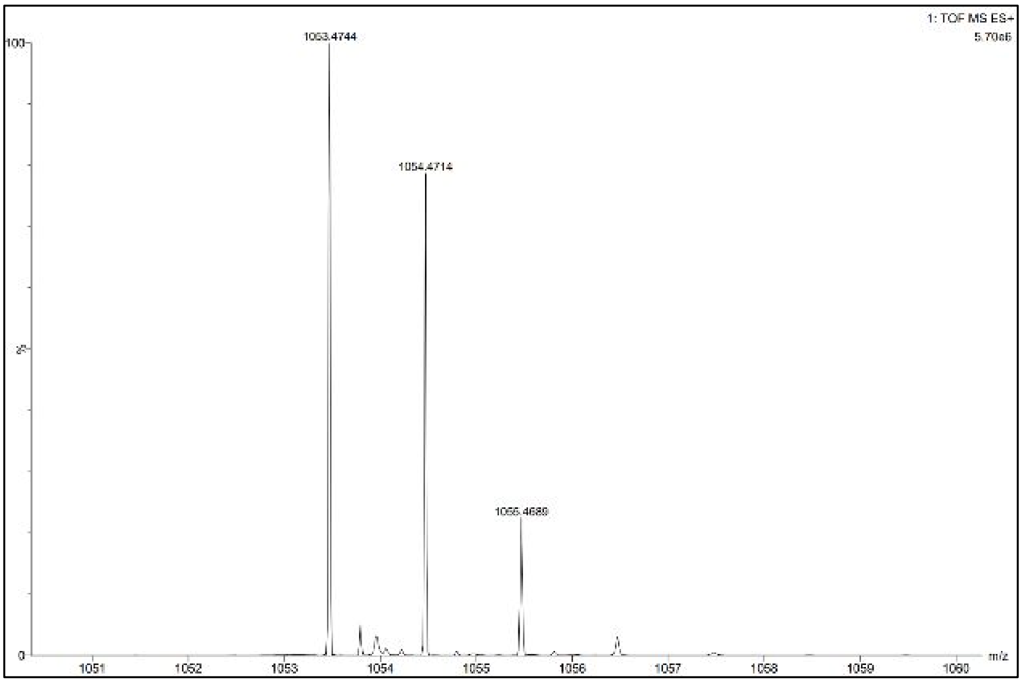

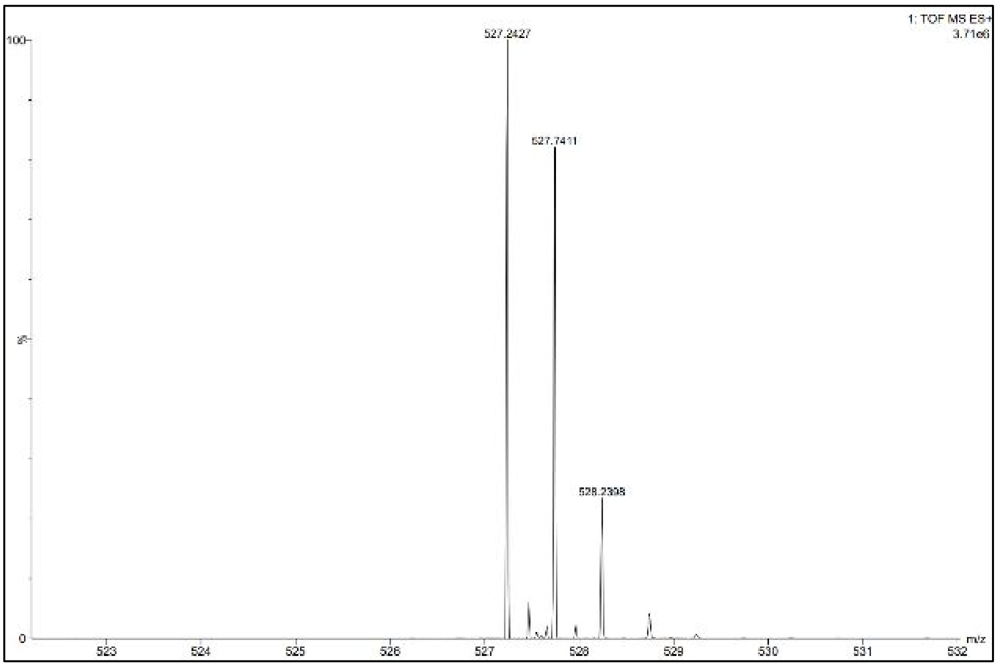

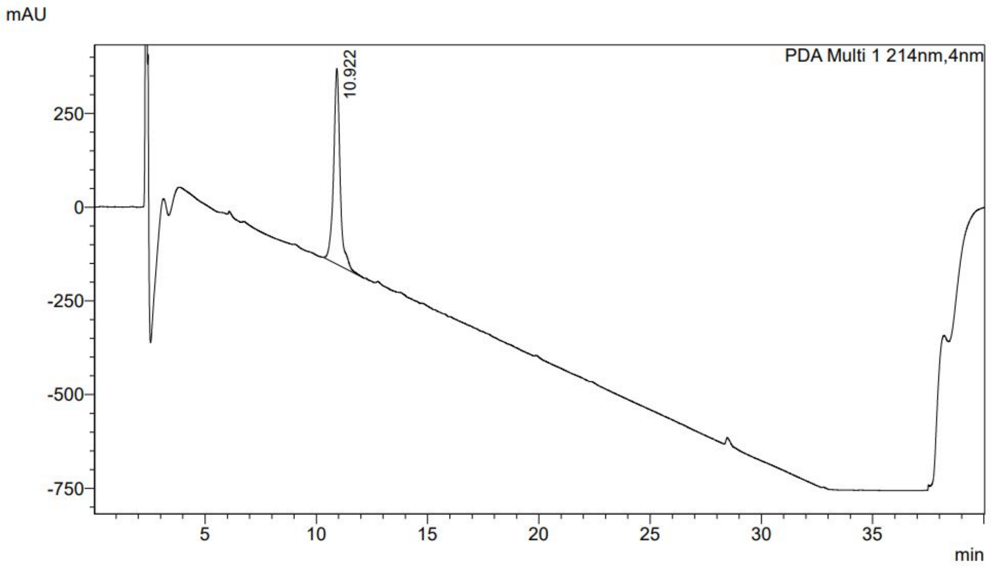

Analytical HPLC: purity = 100%

N1C2T-[N001]

Exact Mass: 898.3821

Expected $[M+H^+]^+$	Measured $[M+H^+]^+$
899.3894	899.3959
**Expected $[M+Na^+]^+$**	**Measured $[M+Na^+]^+$**
450.1983	450.2028

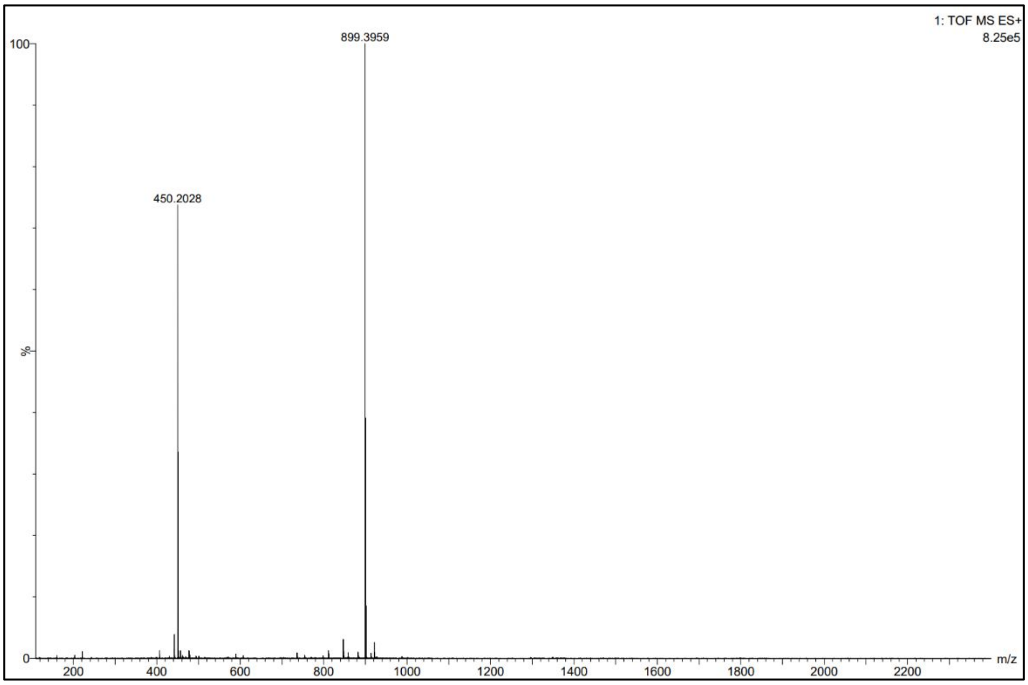

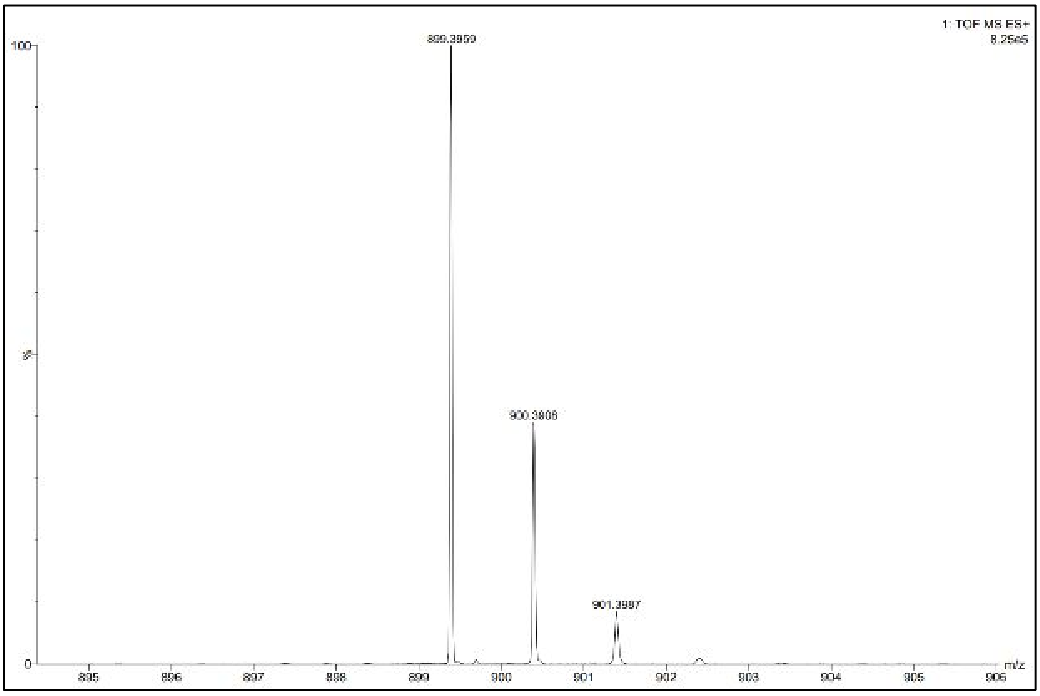

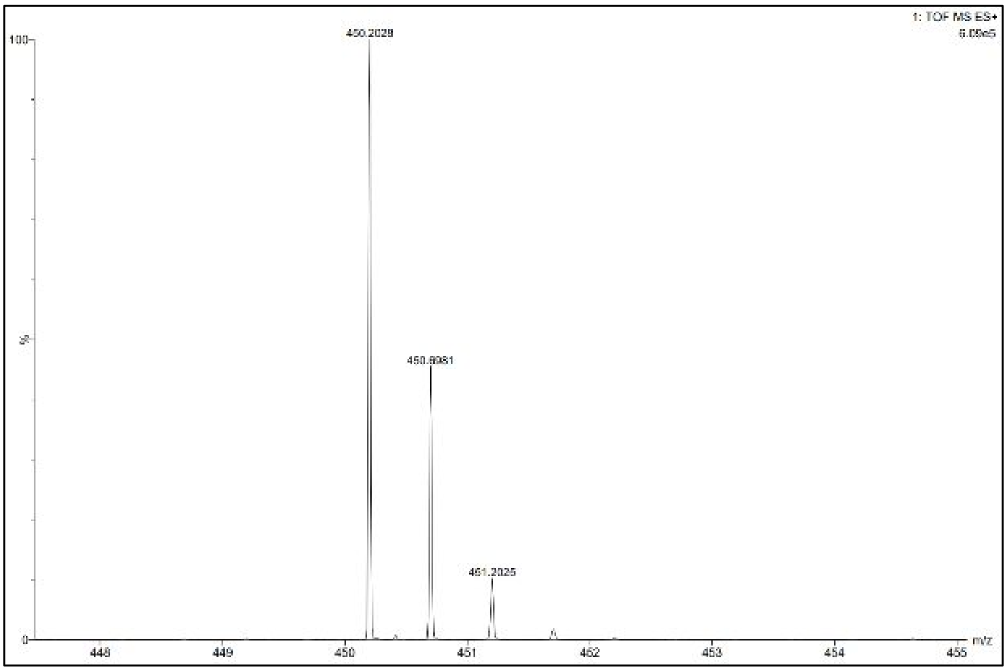

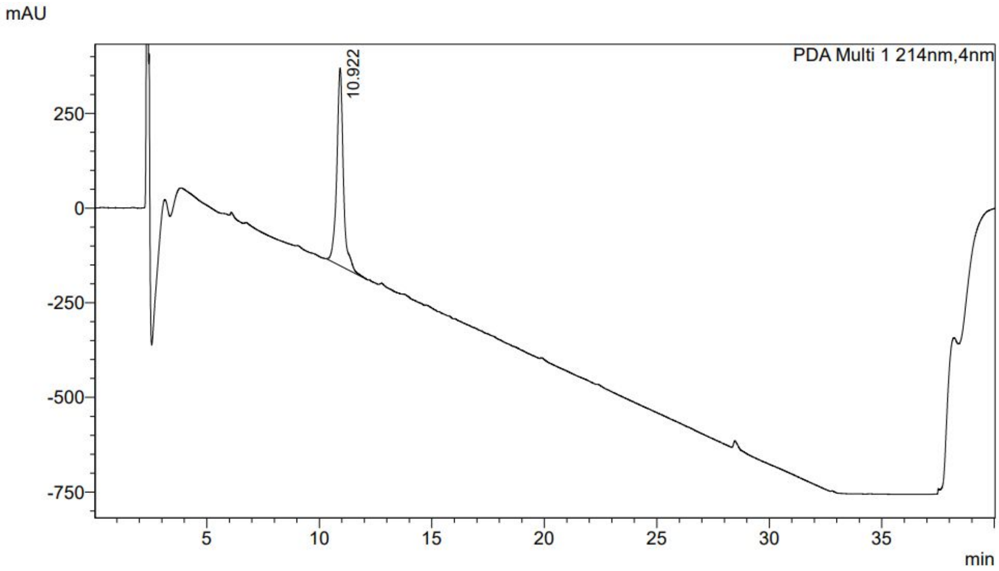

Analytical HPLC: purity = 100%

N1C3T-[N001]

Exact Mass: 770.3235

Expected [M+H+]+	Measured [M+H+]+
771.3308	771.3275
**Expected [M+Na+]+**	**Measured [M+Na+]+**
386.1691	386.1655

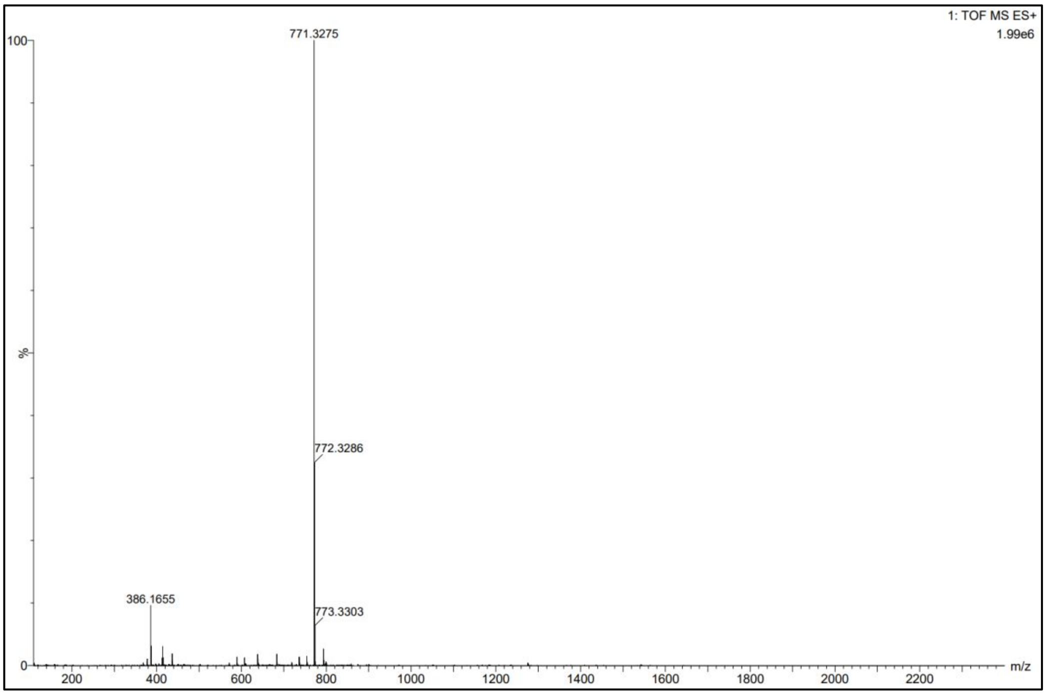

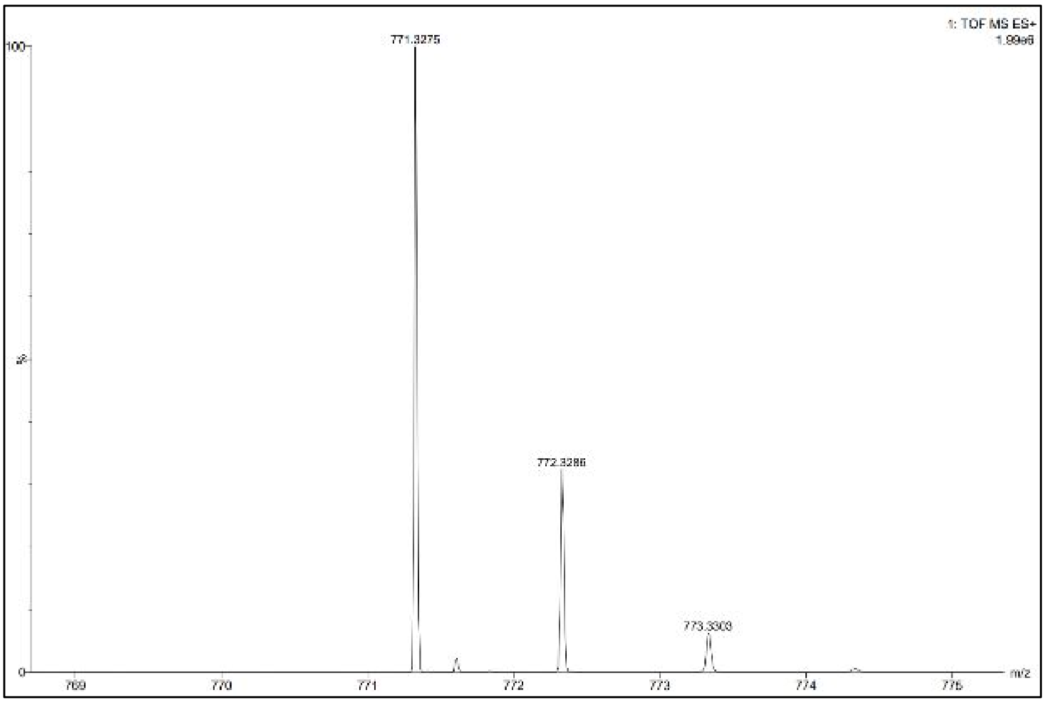

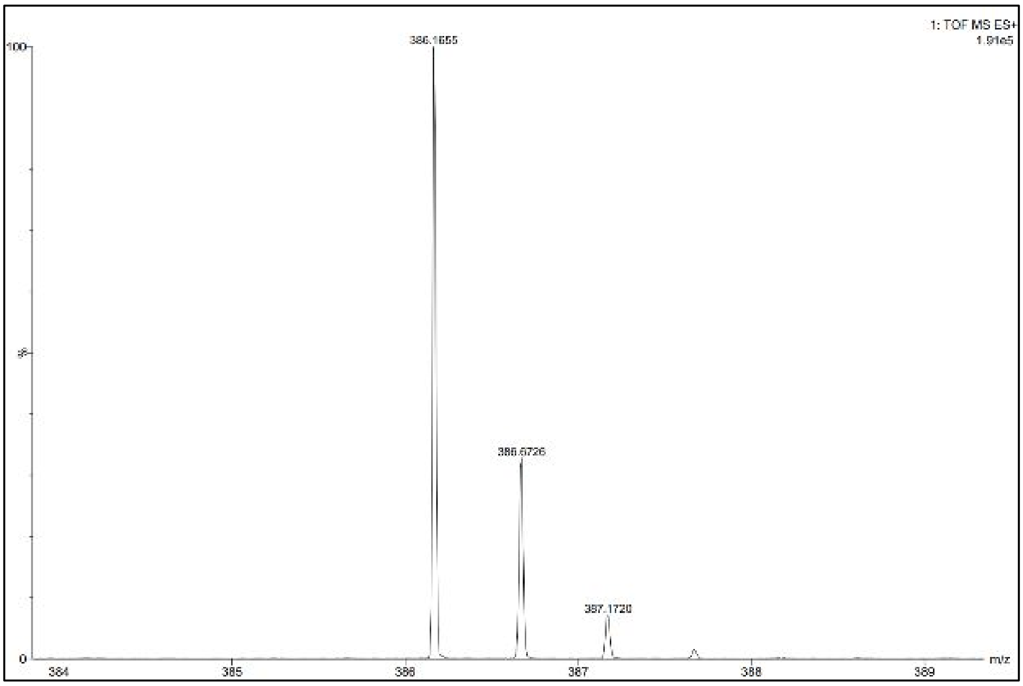

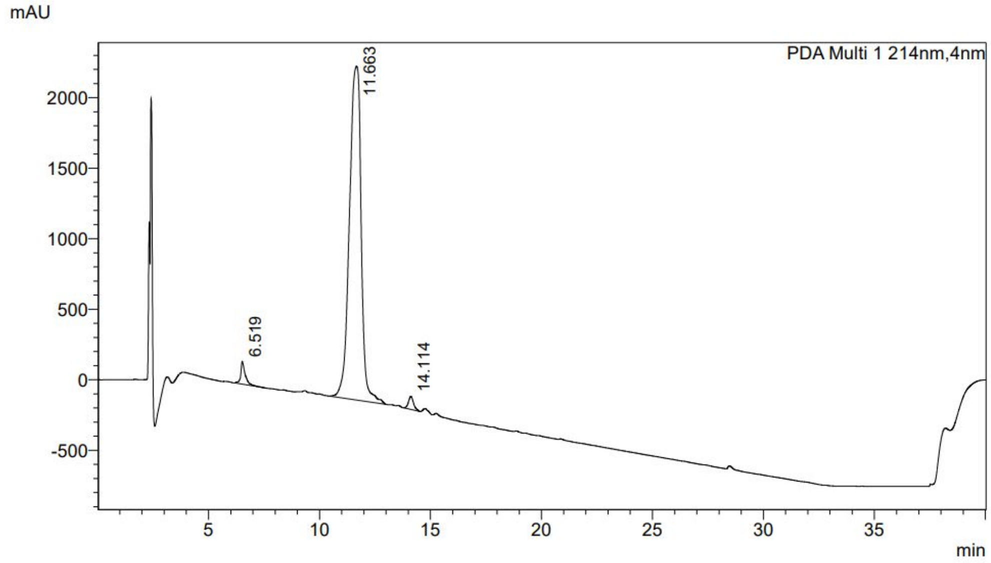

Analytical HPLC: purity = 98%

N1C3T-[N007]

Exact Mass: 890.3446

Expected $[M+H^+]^+$	Measured $[M+H^+]^+$
891.3519	891.3488

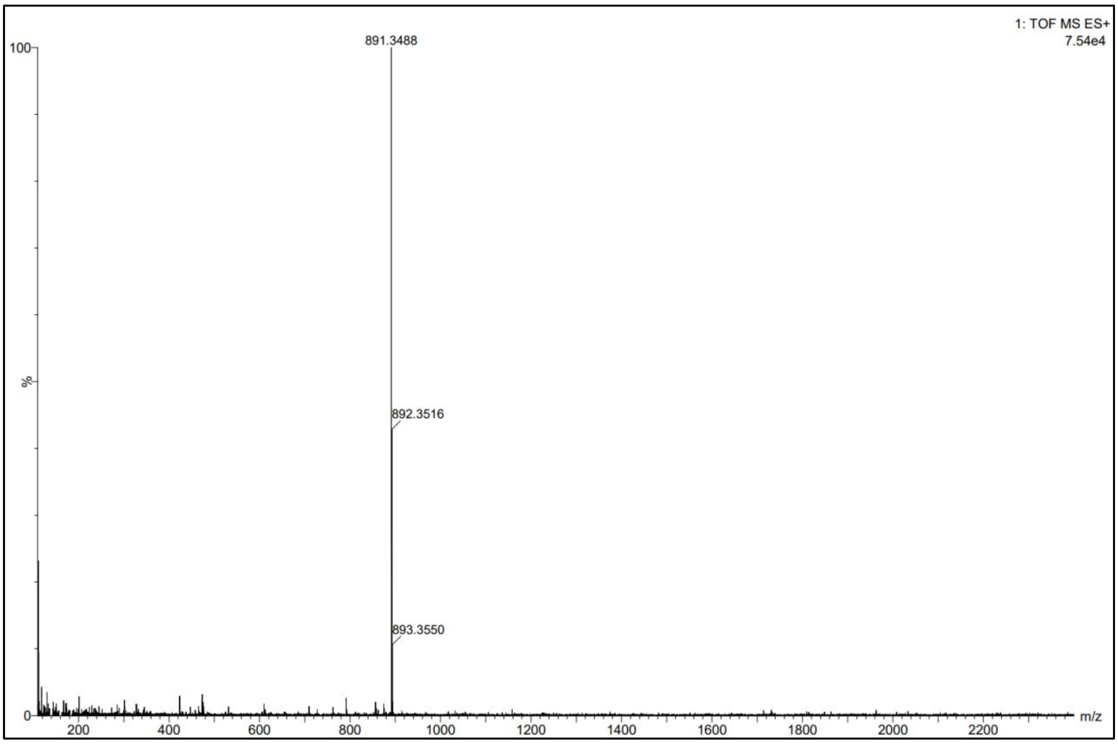

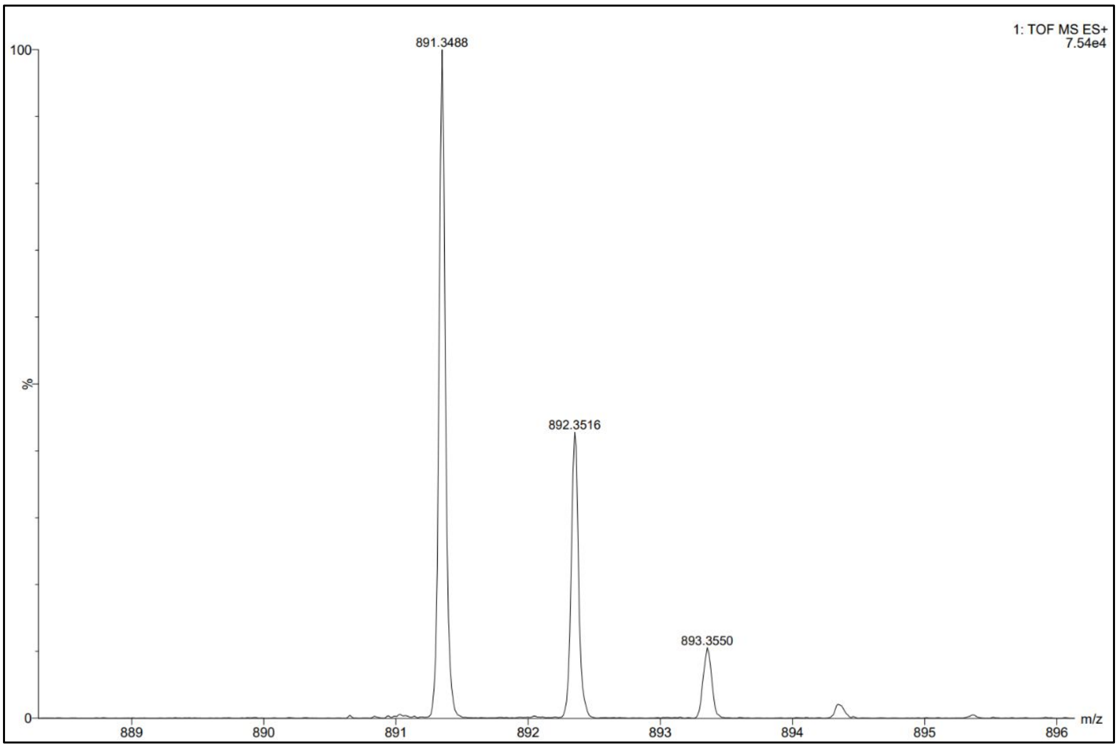

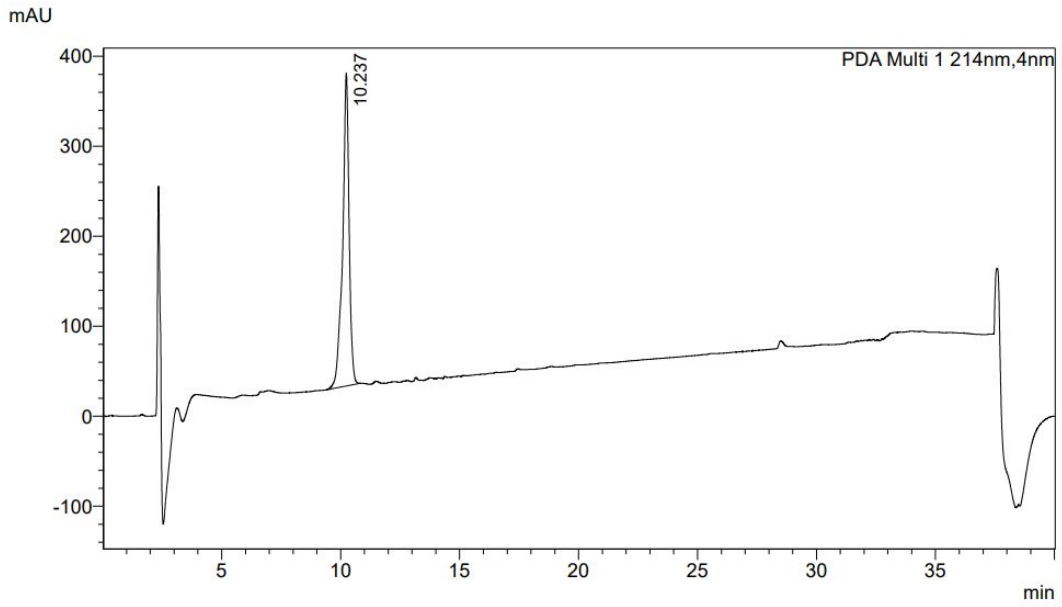

Analytical HPLC: purity = 100%

N1C3T-[N029]

Exact Mass: 914.2849

**Expected [M+H⁺]⁺**	**Measured [M+H⁺]⁺**
915.2922	915.2949
**Expected [M+2H⁺]²⁺**	**Measured [M+2H⁺]²⁺**
458.1498	458.1527

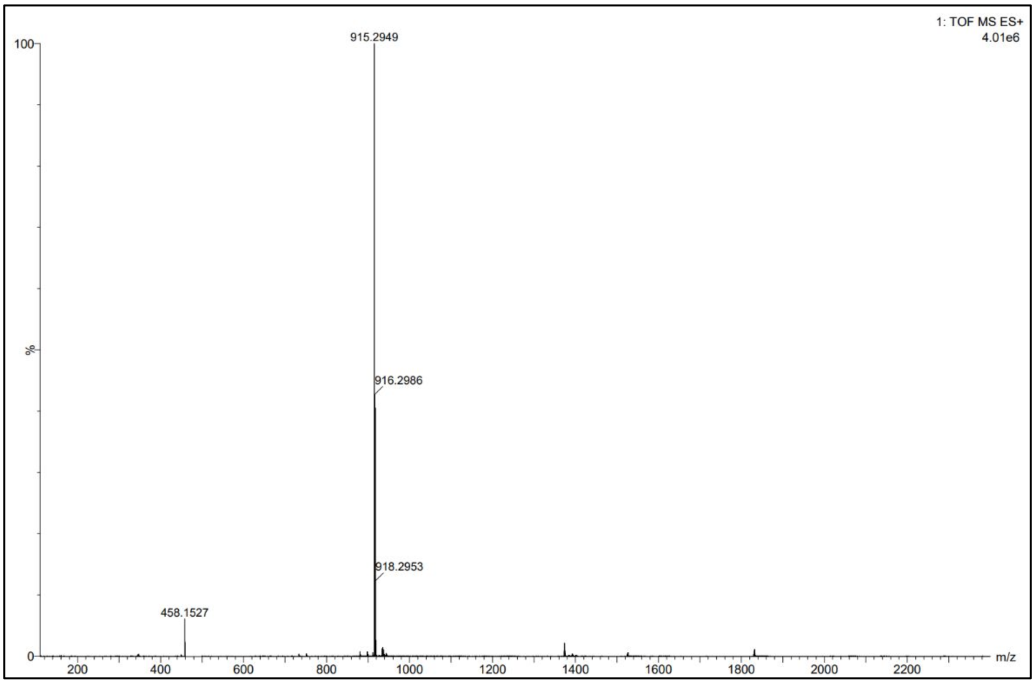

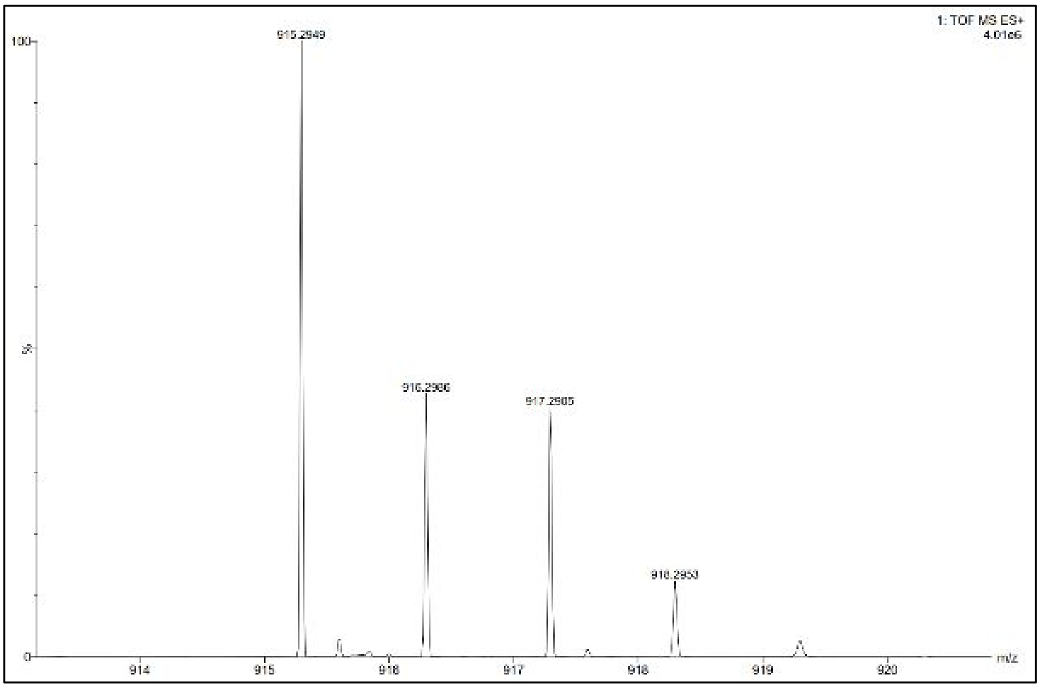

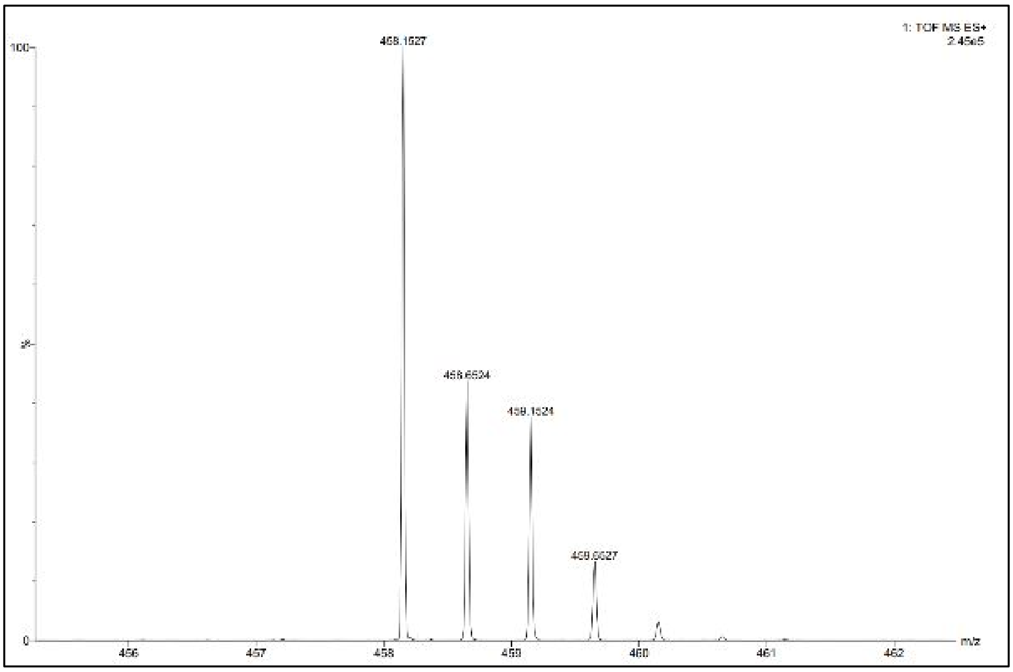

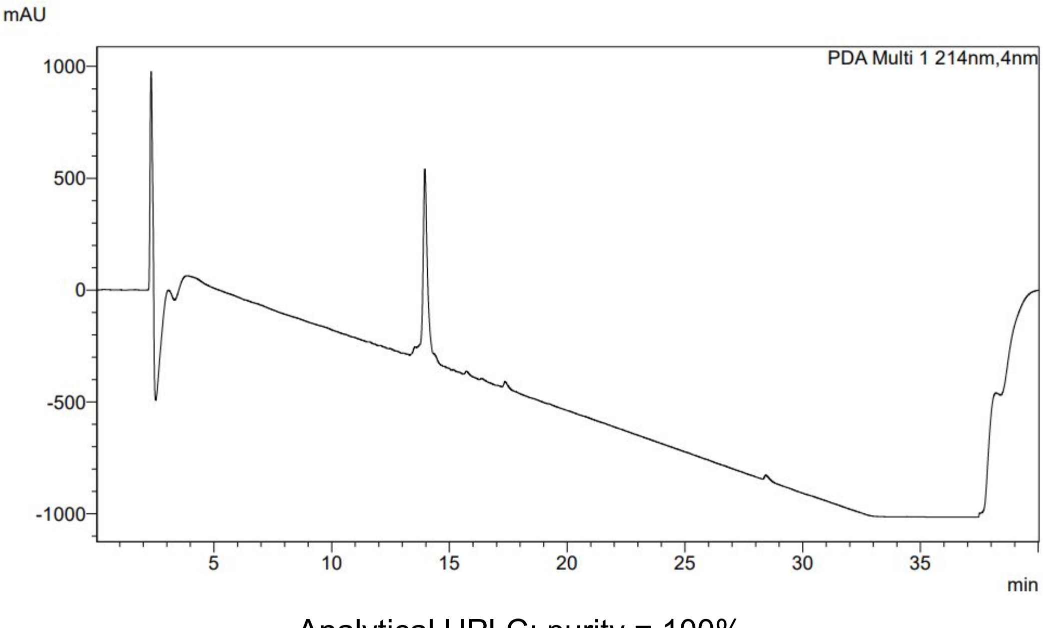

Analytical HPLC: purity = 100%

N1C3T-[N081]

Exact Mass: 845.1972

Expected $[M+H^+]^+$	Measured $[M+H^+]^+$
846.2045	846.2095

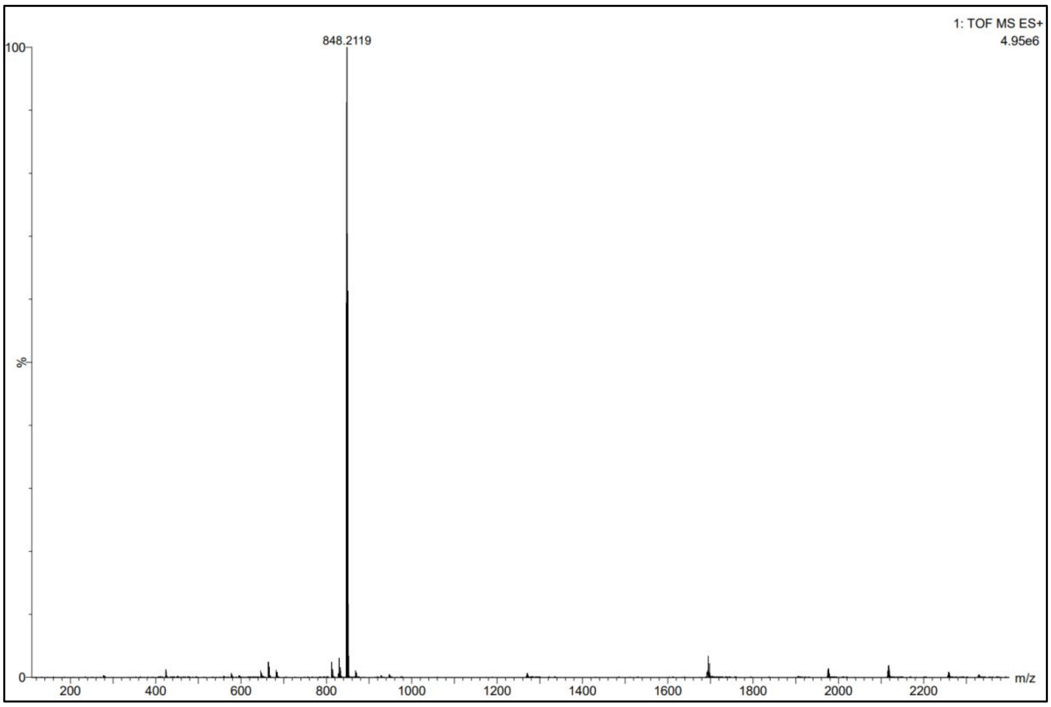

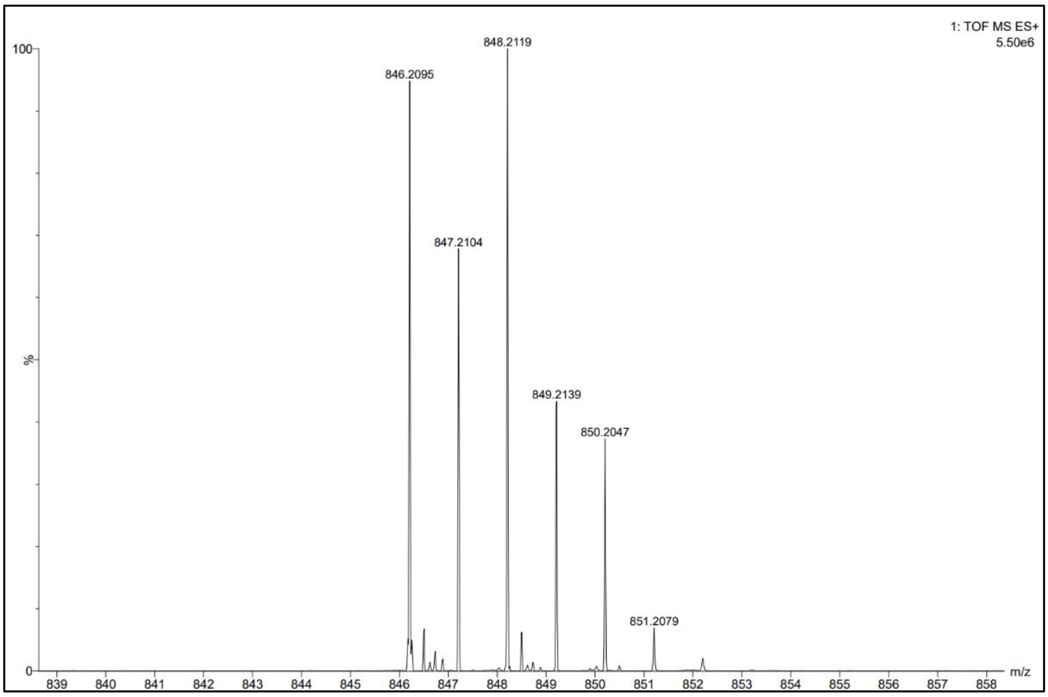

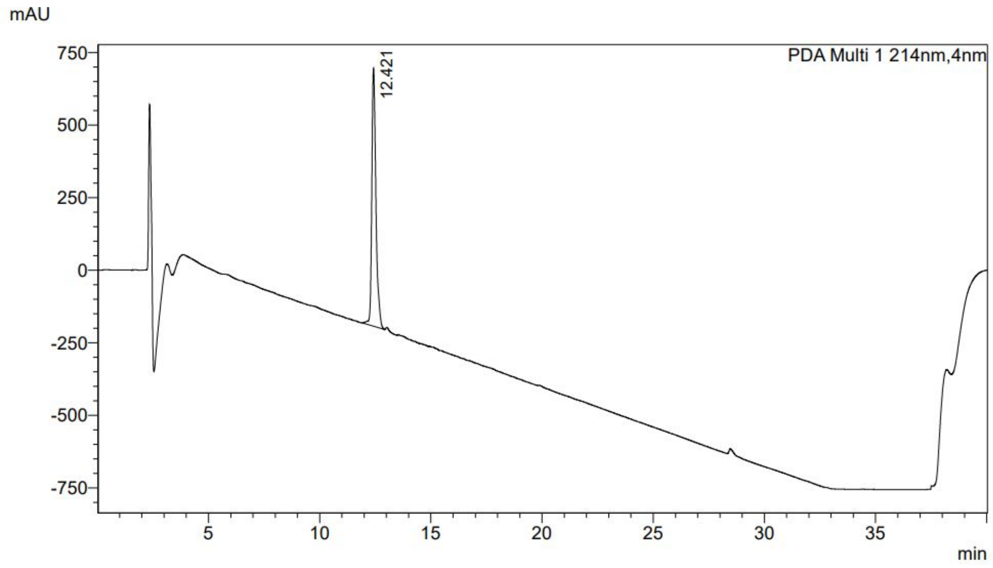

Analytical HPLC: purity = 100%

N1C3T-[N086]

Exact Mass: 886.2238

Expected [M+H⁺]⁺	Measured [M+H⁺]⁺
887.2311	887.2428

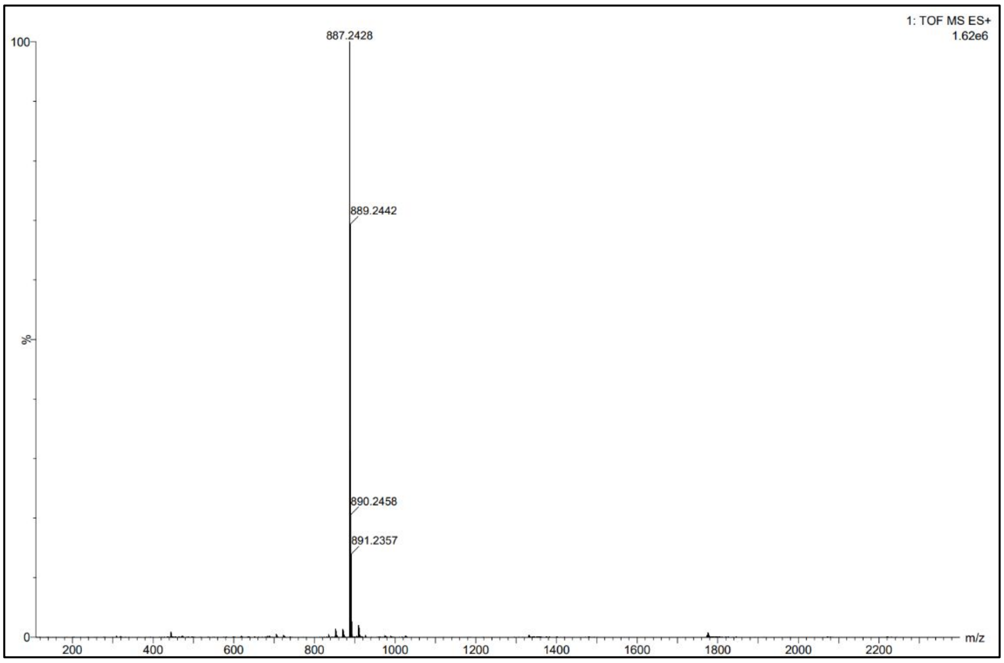

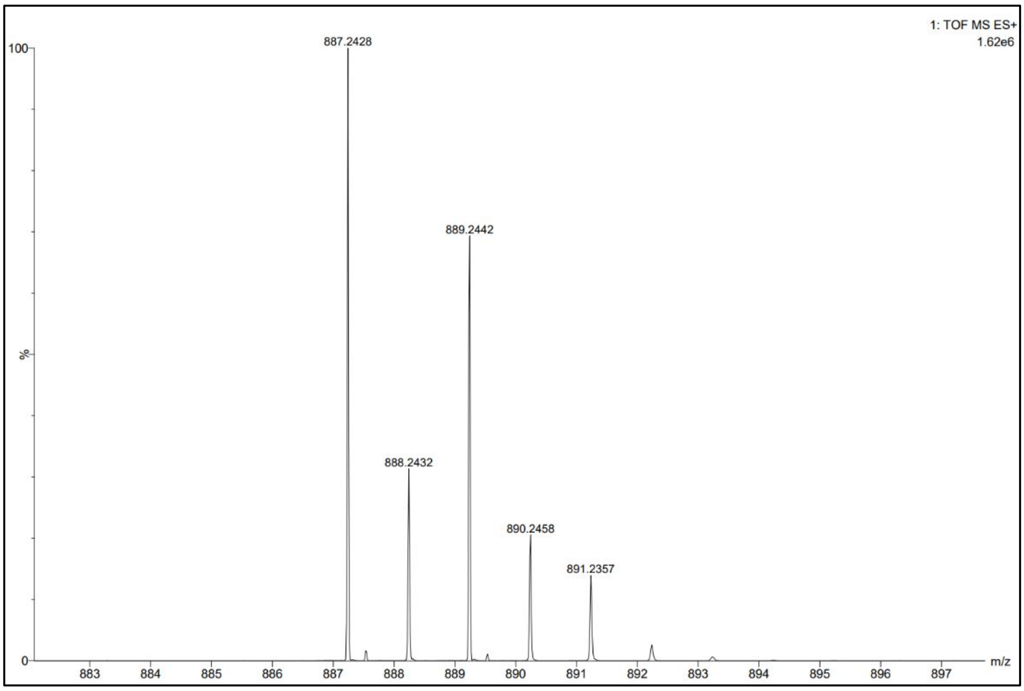

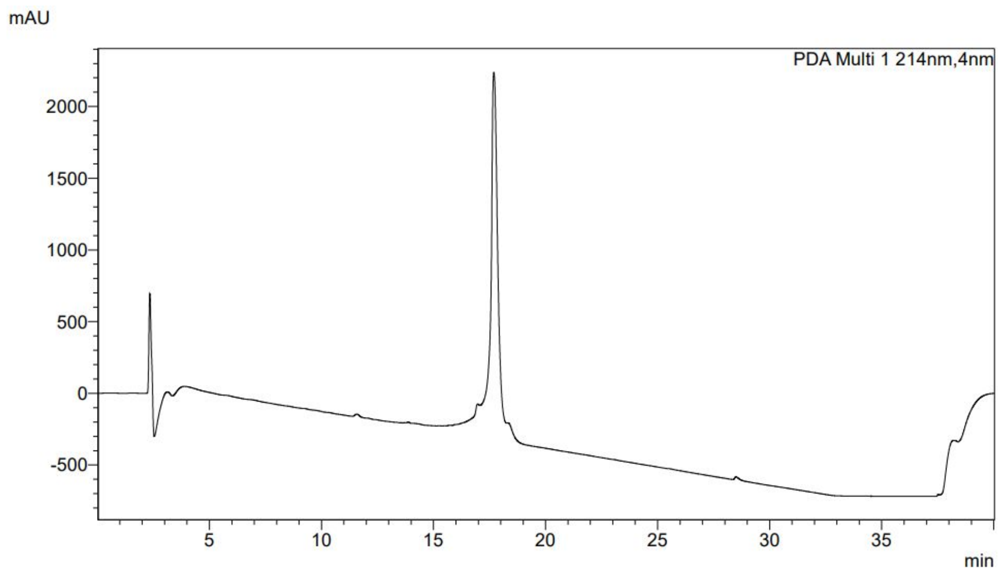

Analytical HPLC: purity = 100%

N1C3T-[N131]

Exact Mass: 959.2216

Expected [M+H$^+$]$^+$	Measured [M+H$^+$]$^+$
960.2289	960.2335

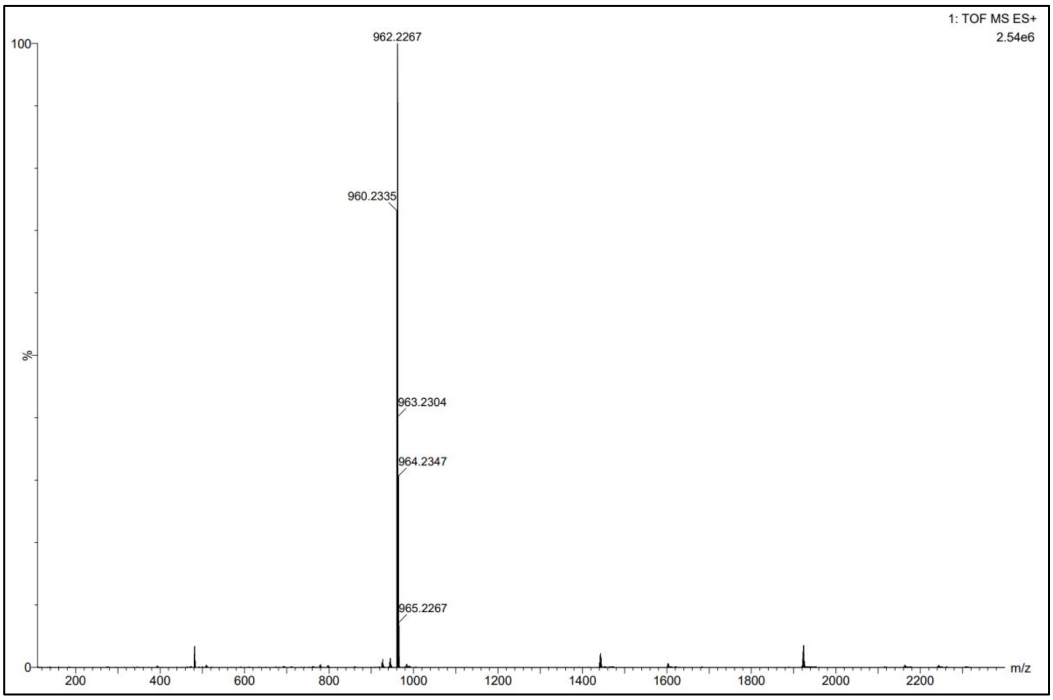

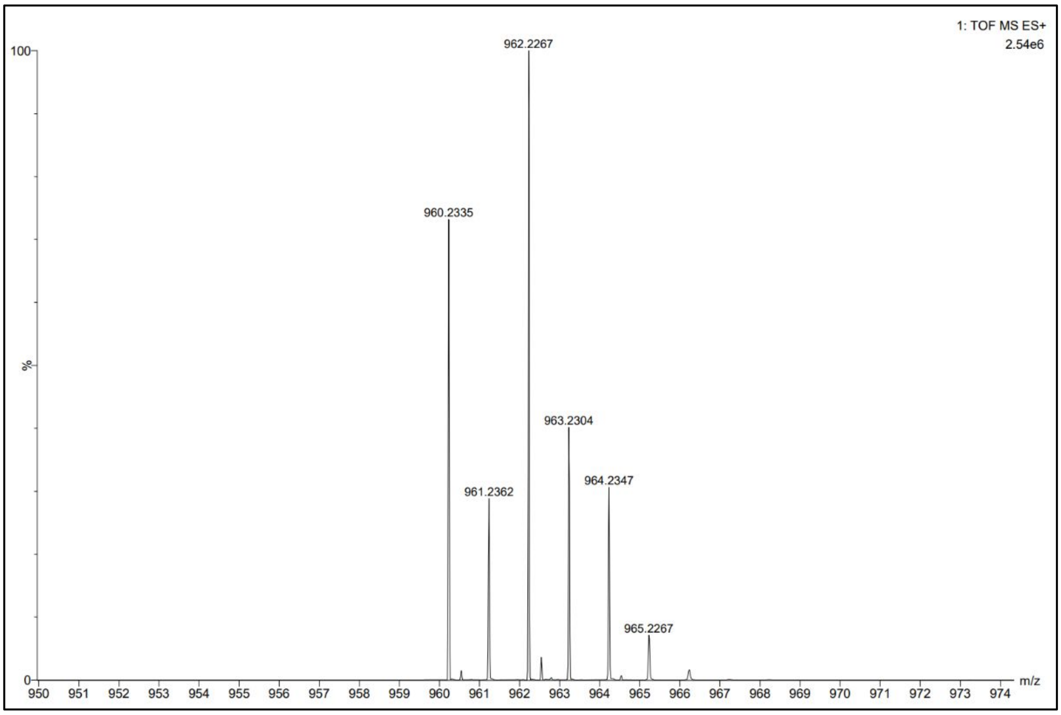

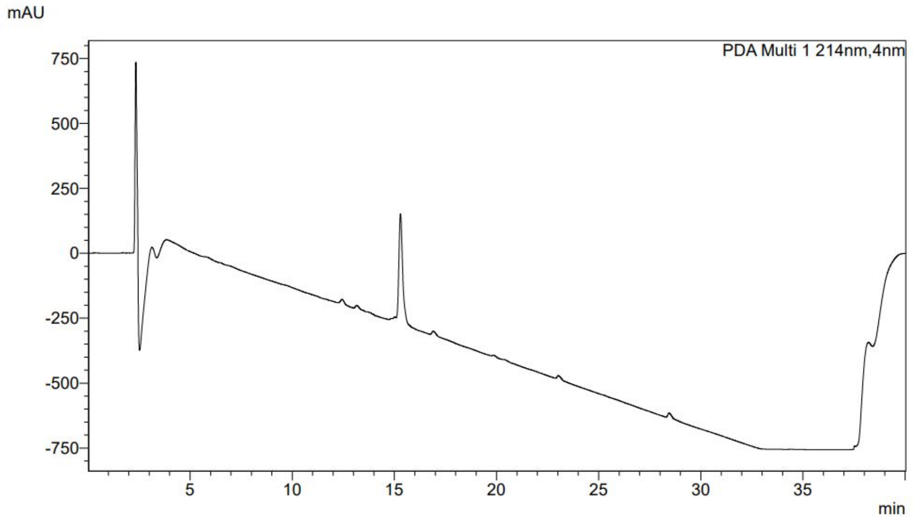

Analytical HPLC: purity = 100%

N1C3T-[N086]-$NHNH_2$

Exact Mass: 902.2551

Expected $[M+H^+]^+$	Measured $[M+H^+]^+$
903.2624	903.2630

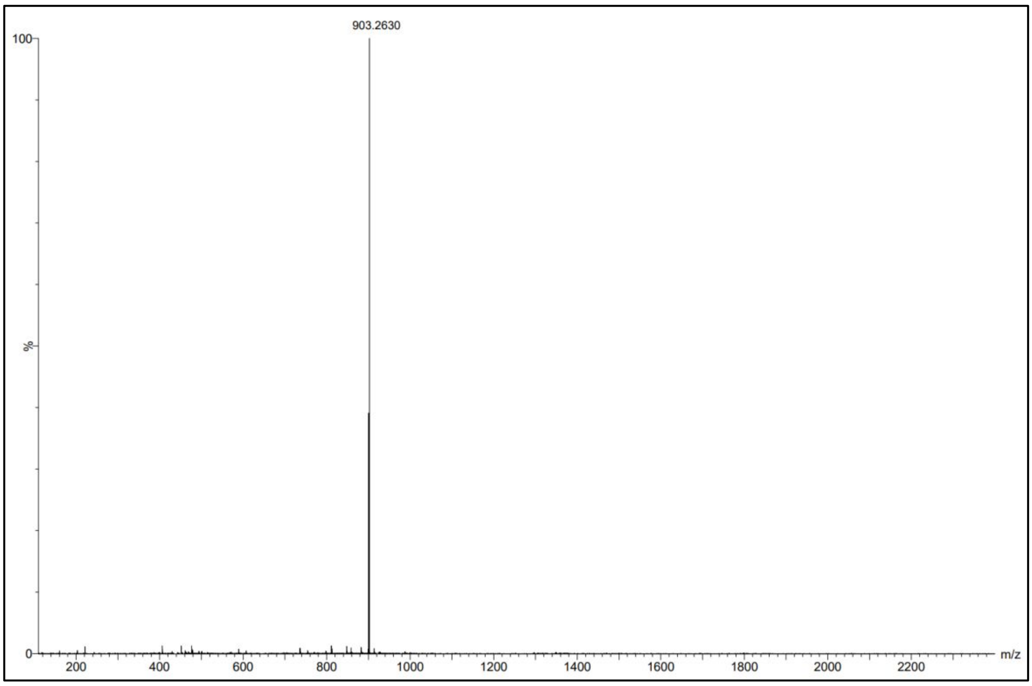

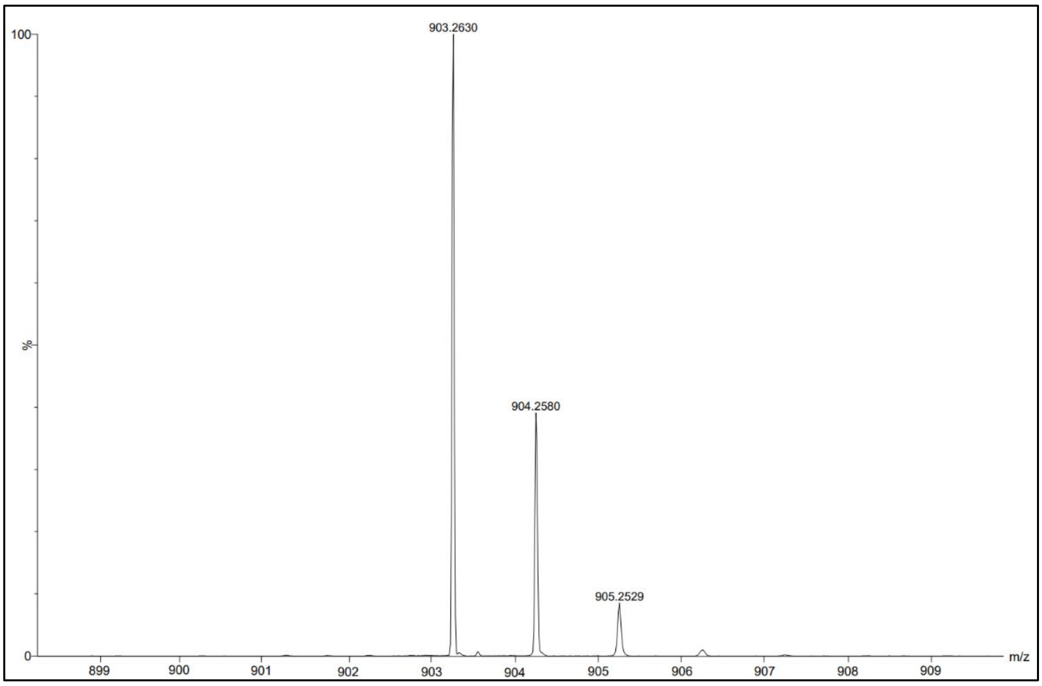

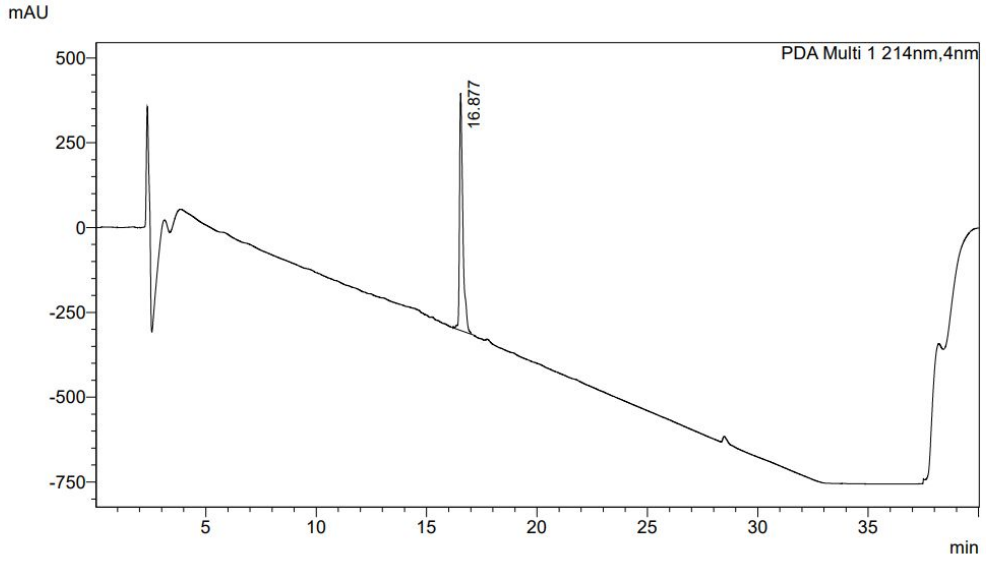

Analytical HPLC: purity = 100%

N1C3T-[N086-C047]

Exact Mass: 1100.3232

Expected $[M+H^+]^+$	Measured $[M+H^+]^+$
1101.3304	1101.3322

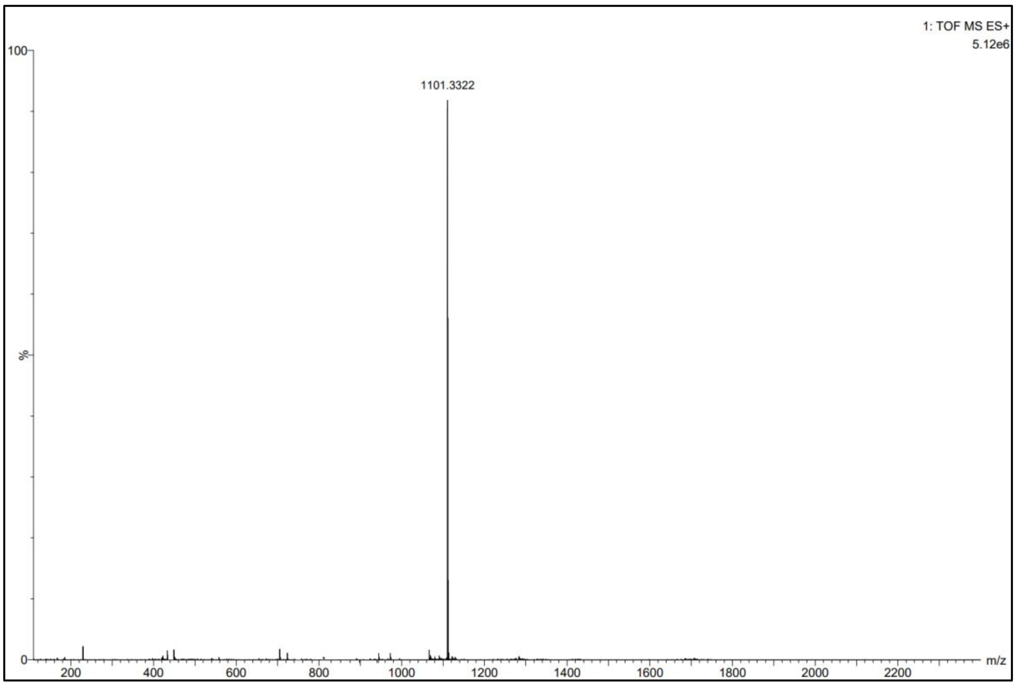

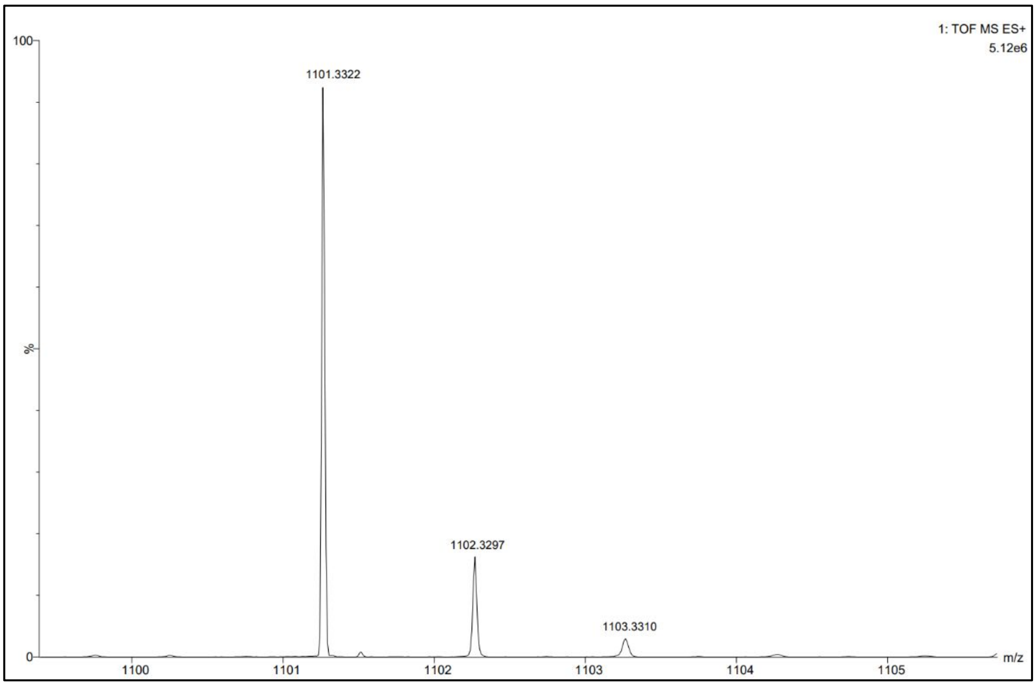

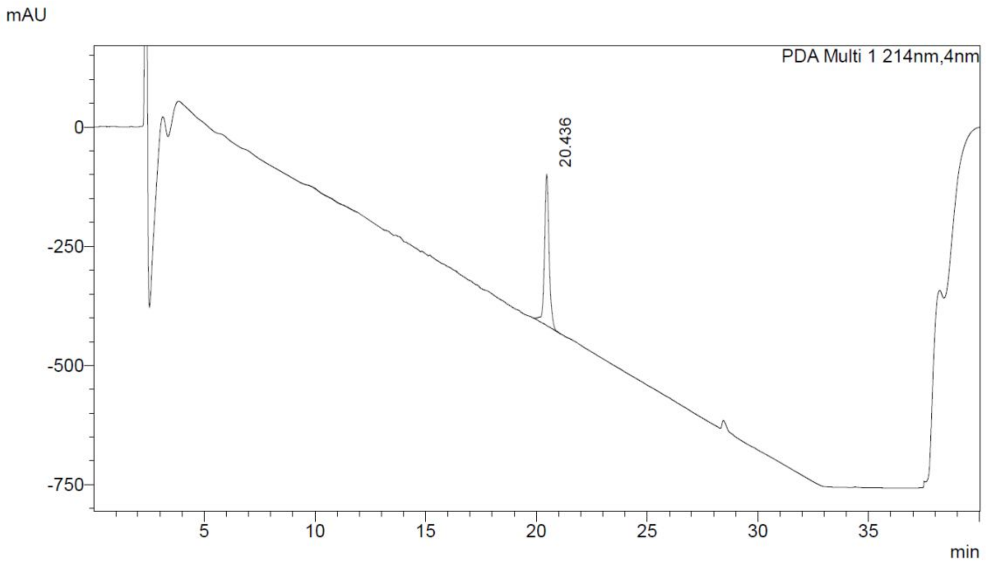

Analytical HPLC: purity = 100%

N1C2T-[N086-C012]

Exact Mass: 1196.2958

**Expected [M+H⁺]⁺**	**Measured [M+H⁺]⁺**
1197.3031	1197.2998
**Expected $[M+2H^+]^{2+}$**	**Measured $[M+2H^+]^{2+}$**
599.1552	599.1559

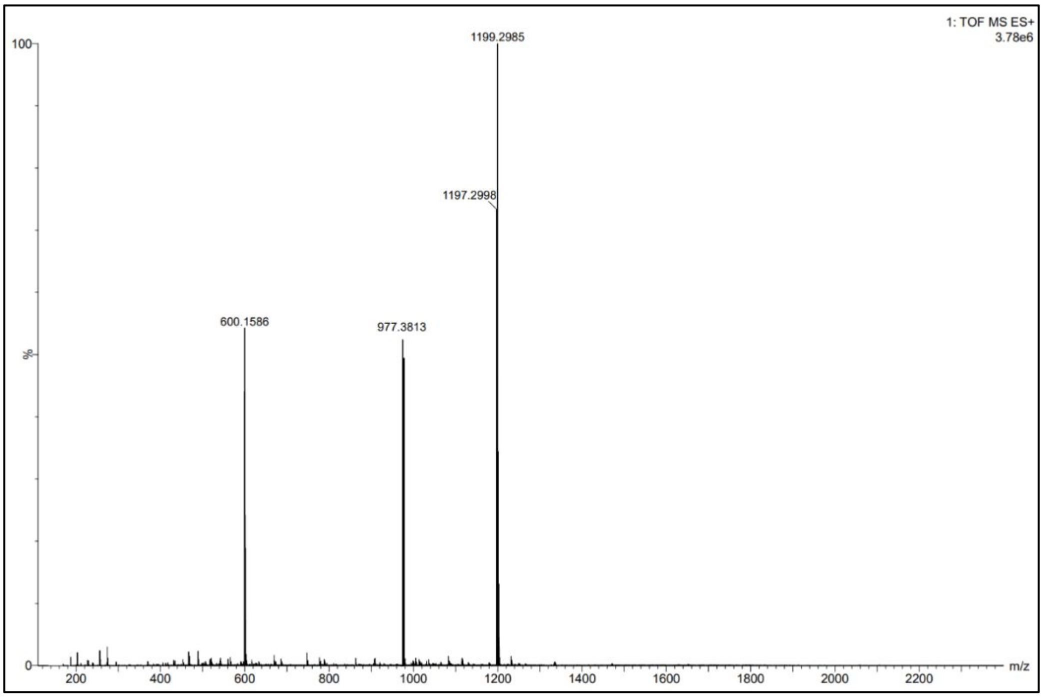

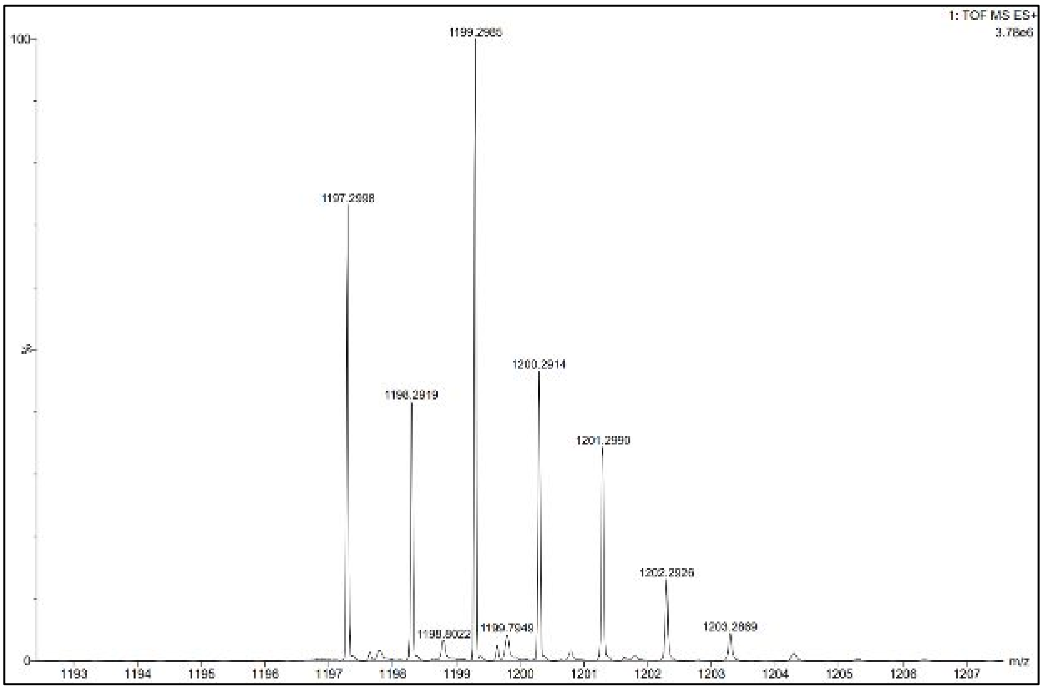

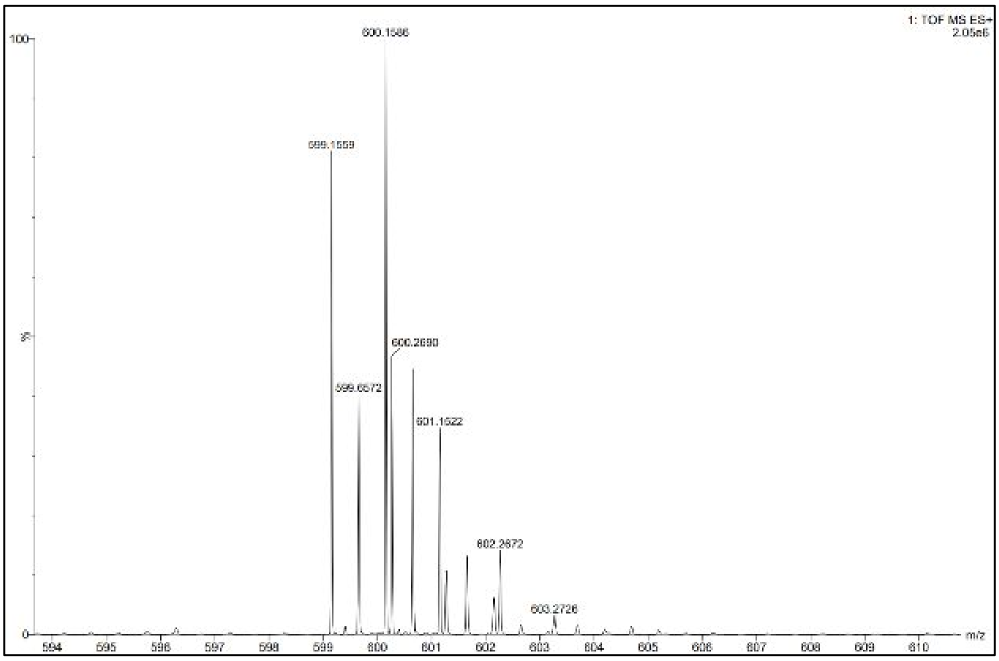

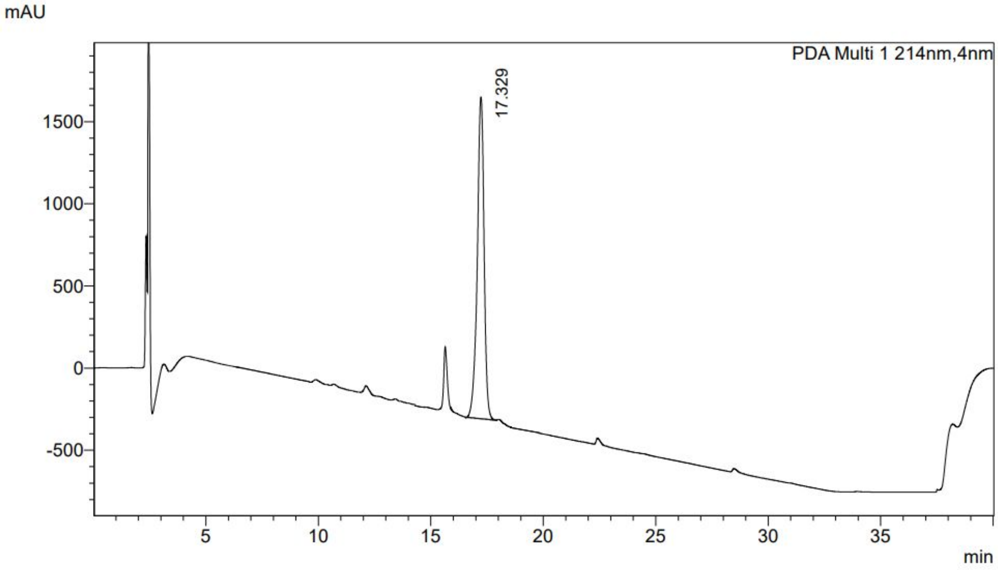

Analytical HPLC: purity = 89%

N1C2T-[N086-C101]

Exact Mass: 1162.3348

**Expected [M+H⁺]⁺**	**Measured [M+H⁺]⁺**
1163.3421	1163.3385
**Expected [M+2H⁺]²⁺**	**Measured [M+2H⁺]²⁺**
582.1747	582.1775

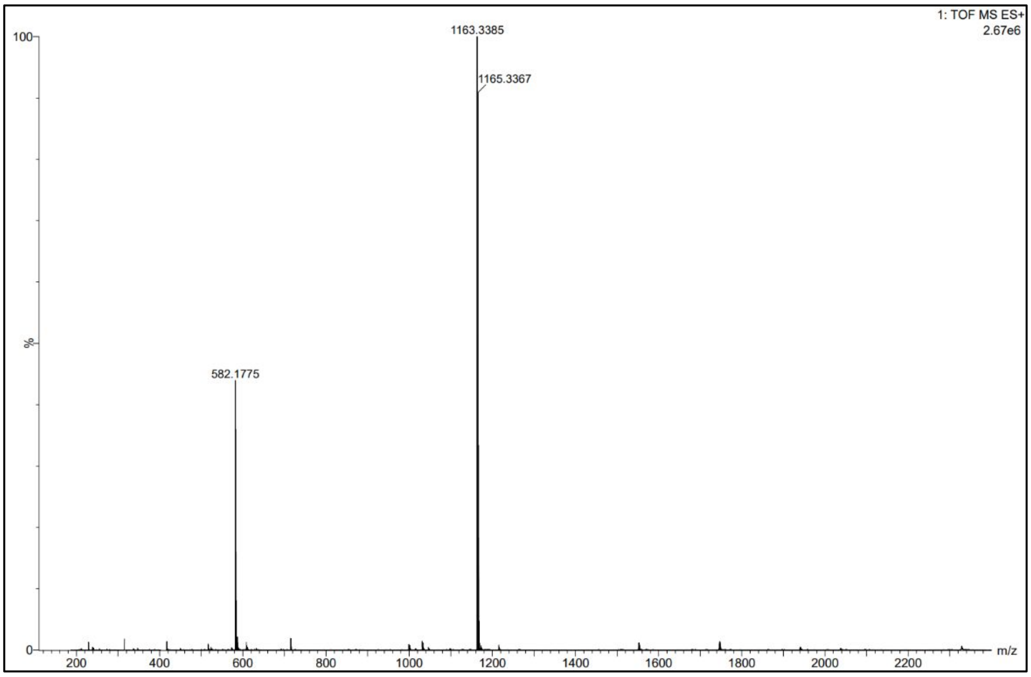

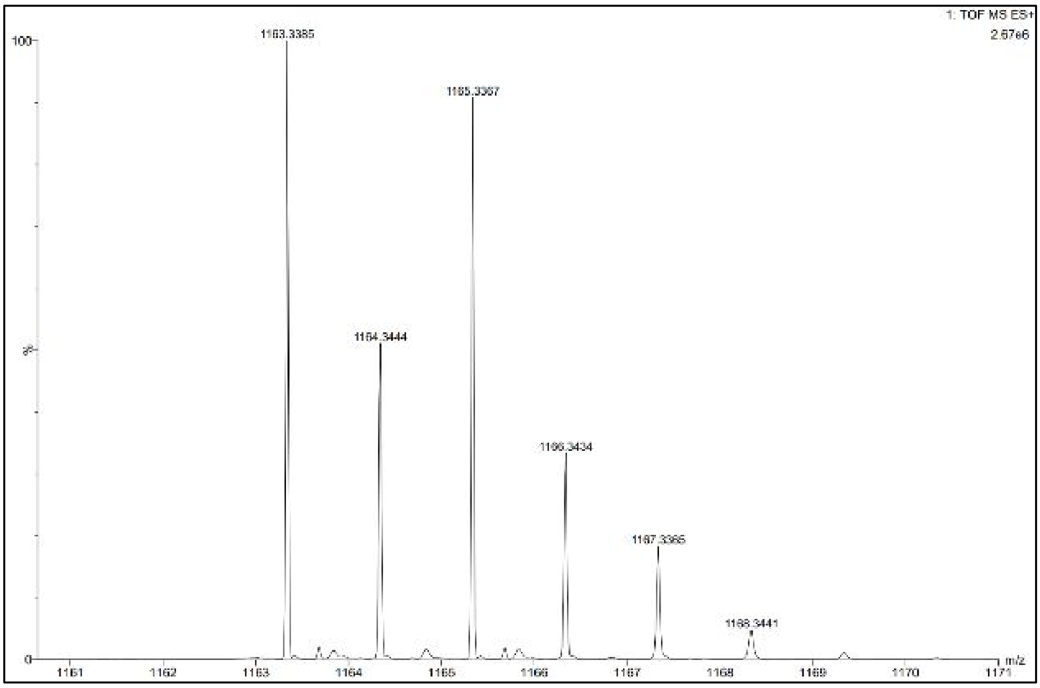

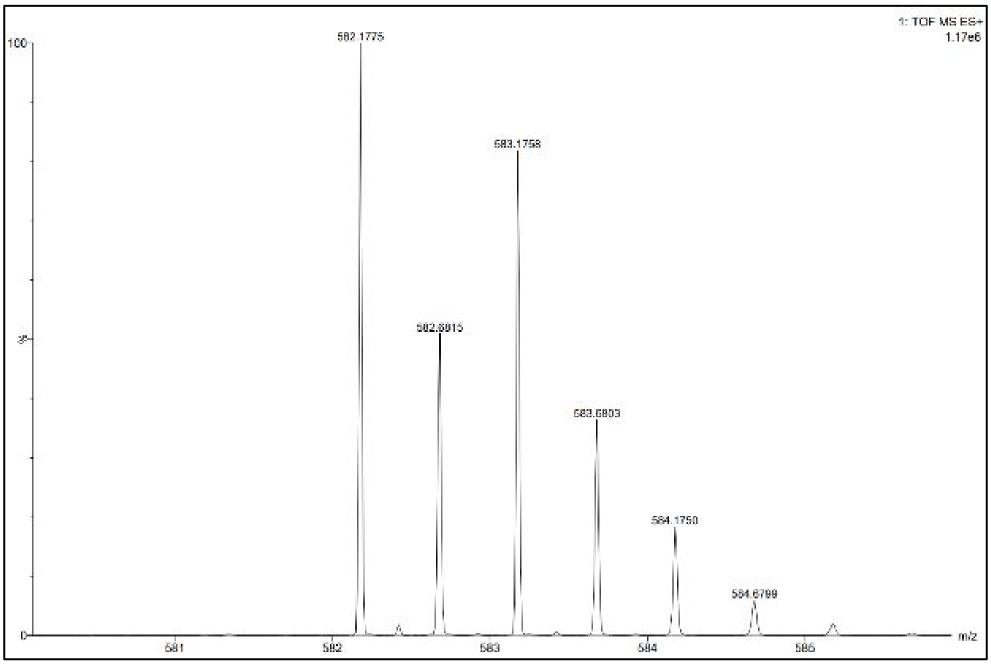

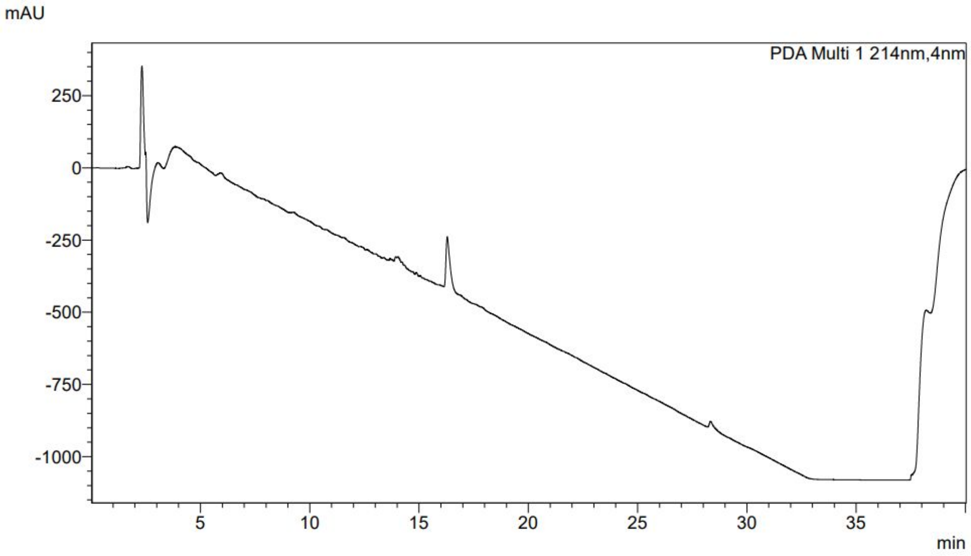

Analytical HPLC: purity = 100%

Aldehyde library compounds

Number	Name	Structure	CAS	M.W.
001	Benzaldehyde	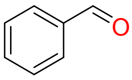	100-52-7	106.124
002	4-Formylcinnamic acid, predominantly *trans*	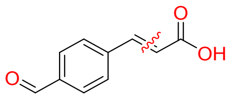	23359-08-2	176.171
003	2-(4-Formylphenyl)-isonicotinic acid	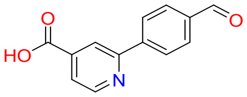	566198-44-5	227.218
004	2-Formylcinnamic acid	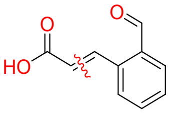	28873-89-4	176.171
005	3'-Formyl-biphenyl-3-carboxylic acid	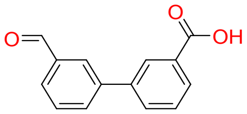	222180-19-0	226.063
006	2′-Formyl[1,1′-biphenyl]-2-carboxylic acid	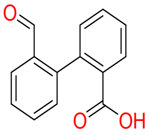	6720-26-9	226.230
007	4′-Formyl-biphenyl-4-carboxylic acid	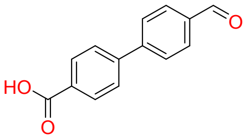	70917-02-1	226.230
008	2-Fluoro-5-formylbenzoic acid	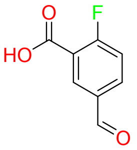	550363-85-4	168.123
009	5-Formyl-2-methoxybenzoic acid	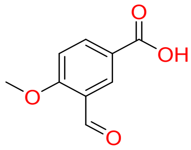	84923-70-6	180.159

Number	Name	Structure	CAS	M.W.
010	4-Formylquinoline-8-carboxylic acid	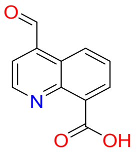	91059-56-2	201.181
011	5-Formylsalicylic acid	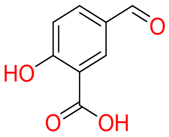	616-76-2	166.132
012	5-Chloro-2-formyl-benzoic acid	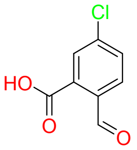	4506-45-0	184.578
013	3-Formylbenzoic acid	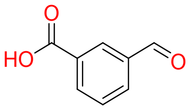	619-21-6	150.133
014	4-Fluoro-3-formyl benzoic acid	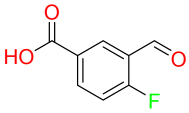	845885-90-7	168.123
015	3-(3-Formyl-1H-indol-1-yl)propanoic acid	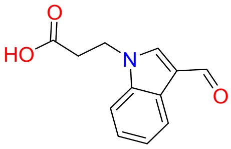	59213-02-4	217.223
016	3-(3-Formylphenoxy-methyl)benzoic acid	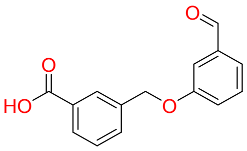	479578-96-6	256.256
017	4-(3-Formyl-2,5-dimethyl-1H-pyrrol-1-yl) benzenecarboxylic acid	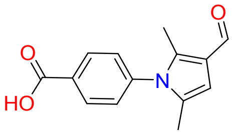	52034-38-5	243.261
018	6-Formyl-2,4-dimethyl-4H-thieno[3,2-b] pyrrole-5-carboxylic acid	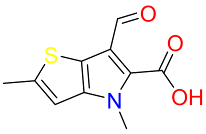	919036-50-3	223.251

Number	Name	Structure	CAS	M.W.
019	2-(4-Formyl-1H-imidazol-1-yl)acetic acid	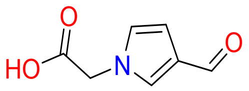	1303968-26-4	154.125
020	4-Formyl-1,2,5-trimethyl-1H-pyrrole-3-carboxylic acid	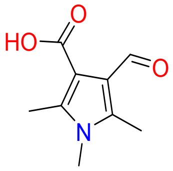	861582-85-6	181.190
021	5-Formyl-2-thiophene-carboxylic acid	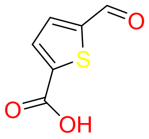	4565-31-5	156.161
022	5-Formyl-2-furoic-acid	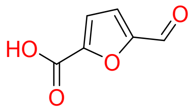	4805-98-5	140.094
023	4-(5-Formyl-2-furyl)-2-hydroxybenzoic acid	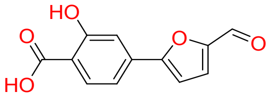	436088-45-8	232.191
024	5-Formyl-1H-pyrrole-2-carboxylic acid	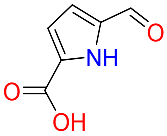	7126-51-4	139.110
025	4-Formyl-1H-pyrazole-3-carboxylic acid	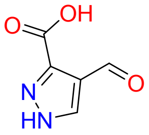	123-123-123	140.198
026	4-(2-Formyl-1H-pyrrol-1-yl)benzoic acid	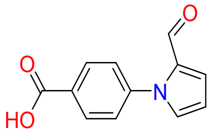	35344-94-6	215.207
027	5-((3-Formyl-1H-indol-1-yl)methyl)furan-2-carboxylic acid	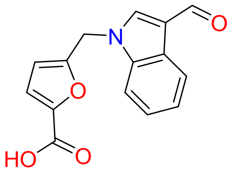	838584-50-2	269.255

Number	Name	Structure	CAS	M.W.
028	3-Formyl-1-isobutyl-1H-indole-4-carboxylic acid	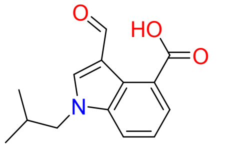	1071914-76-5	245.277
029	4-chloro-3-(5-formyl-furan-2-yl)-benzoic acid	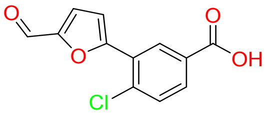		250.637
030	2-(3-Formylphenoxy)-2-phenylacetic acid	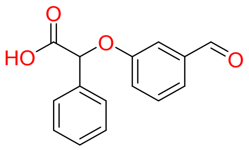	1094672-99-7	256.256
031	5-(4-Formylphenoxy-methyl)-3-methylfuran-2-carboxylic acid	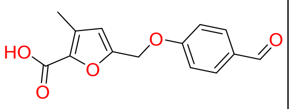	1153306-99-0	260.244
032	3-Formyl-1H-indole-7-carboxylic acid	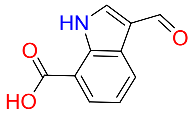	317854-65-2	189.170
033	3-(3-Formyl-2,5-dimethyl-pyrrol-1-yl)-2-methyl-benzoic acid	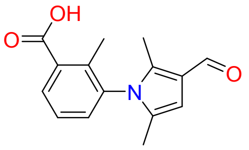	409353-61-3	257.288
034	(2-Chloro-6-ethoxy-4-formylphenoxy)acetic acid	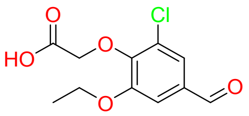	428836-03-7	258.656
035	2-(3-Formyl-1H-indol-1-yl)propanoic acid	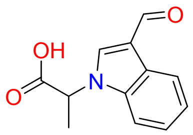	166747-91-7	217.223
036	4-(3-Methoxy-4-formyl) phenoxybutyric acid	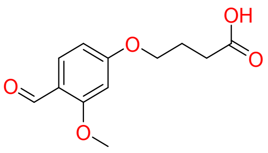	309964-23-6	238.238

Number	Name	Structure	CAS	M.W.
037	5-Formyl-2,4-dimethyl-1H-pyrrole-3-carboxylic acid	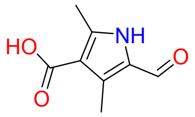	253870-02-9	167.163
038	2-Chloro-6-nitro-benzaldehyde	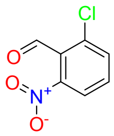	6361-22-4	185.566
039	6-Nitropiperonal	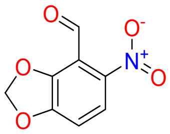	712-97-0	195.130
040	Isovaleraldehyde	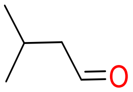	590-86-3	86.1334
041	Ethyl 2-formyl-1-cyclopropanecarboxylate, predominantly *trans*	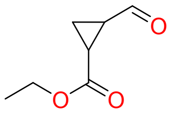	20417-61-2	142.153
042	Mesitaldehyde	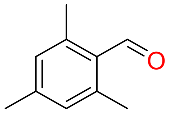	487-68-3	148.204
043	(*S*)-(-)-Perillaldehyde	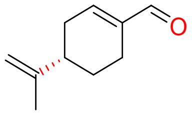	18031-40-8	150.220
044	3-Furancarboxaldehyde	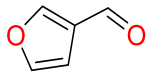	498-60-2	96.0848
045	3,3-Dimethylbutyr-aldehyde	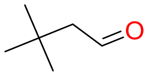	2987-16-8	100.160
046	Benzyloxyacetaldehyde	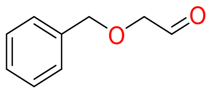	60656-87-3	150.176

Number	Name	Structure	CAS	M.W.
047	3-Formyl-6-isopropyl-chromone	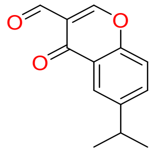	49619-58-1	216.235
048	2,3,6,7-Tetrahydro-8-hydroxy-1H,5H-benzo[ij]quinolizine-9-carboxaldehyde	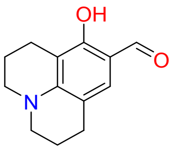	63149-33-7	217.267
049	4,5-Dimethyl-2-furaldehyde	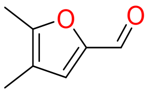	52480-43-0	124.139
050	4-Imidazole-carboxaldehyde	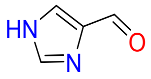	3034-50-2	96.0888
051	4-Bromo-2-fluorobenzaldehyde	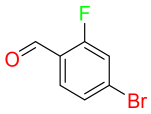	57848-46-1	203.010
052	*N*-Boc-2-aminoacet-aldehyde	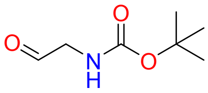	89711-08-0	159.184
053	1-Methyl-2-imidazole-carboxaldehyde	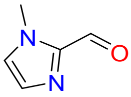	13750-81-7	110.116
054	Boc-L-prolinal	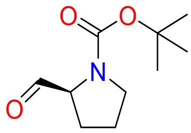	69610-41-9	199.249
055	2-Benzofuran-carboxaldehyde	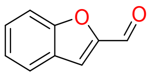	4265-16-1	146.145
056	Thianaphthene-3-carboxaldehyde	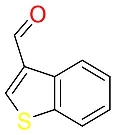	5381-20-4	162.212

Number	Name	Structure	CAS	M.W.
057	3-Formyl-2-thienylboronic acid	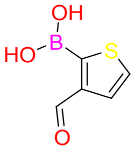	17303-83-2	155.969
058	2-Chloro-6-methylquinoline-3-carboxaldehyde	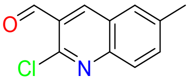	73568-27-1	205.644
059	(*R*)-1,4-Dioxaspiro [4.5]decane-2-carboxaldehyde	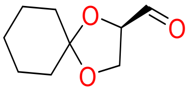	78008-36-3	170.207
060	2-Butyl-5-chloro-1H-imidazole-4-carboxaldehyde	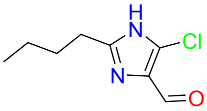	83857-96-9	186.641
061	Methyl 3-formyl-4-nitrobenzoate	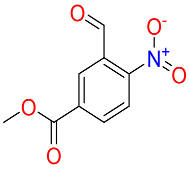	148625-35-8	209.157
062	6-Bromo-3-pyridine-carboxaldehyde	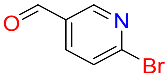	149806-06-4	186.008
063	4-Methylthiazole-5-carboxaldehyde	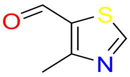	82294-70-0	127.167
064	3,5-Difluoro-4-formyl-phenylboronic acid	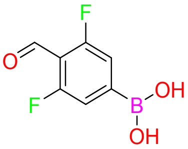	870718-11-9	185.921
065	*N*-(3-Formyl-2-pyridinyl)-2,2-dimethylpropan-amide	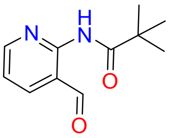	86847-64-5	206.244
066	5-Methylisoxazole-3-carboxaldehyde	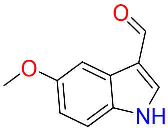	62254-74-4	175.186

Number	Name	Structure	CAS	M.W.
067	3-Formyl-5-methylphenylboronic acid	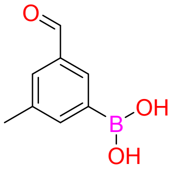	870777-33-6	163.967
068	1-Boc-4-(2-formylphenyl)piperazine	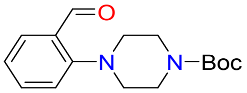	174855-57-3	290.362
069	2,4-Dihydroxy-6-methylbenzaldehyde	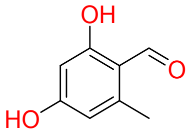	487-69-4	152.149
070	2-Aminothiazole-5-carboxaldehyde	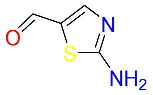	1003-61-8	128.155
071	6-Methoxy-2-pyridinecarboxaldehyde	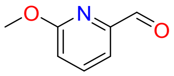	54221-96-4	137.138
072	*N*-Benzylpiperidine-4-carboxaldehyde	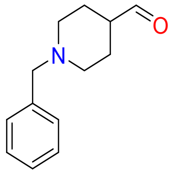	22065-85-6	203.284
073	4-(1-Piperidinyl)-benzaldehyde	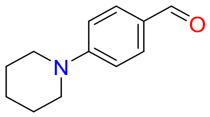	10338-57-5	189.257
074	3-Methyl-1-phenyl-1H-pyrazole-4-carboxaldehyde	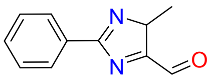	21487-48-9	186.213
075	7-Azaindole-3-carboxaldehyde	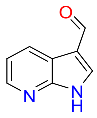	4649-9-6	146.148
076	4-Bromothiazole-2-carboxaldehyde	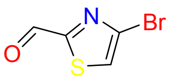	167366-05-4	192.036

Number	Name	Structure	CAS	M.W.
077	1-(2-Tetrahydropyranyl)-1H-pyrazole-5-carboxaldehyde	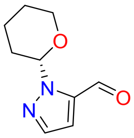	957483-88-4	180.206
078	3-Bromo-5-nitrobenzaldehyde	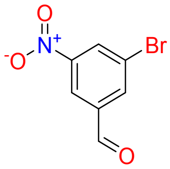	355134-13-3	230.017
079	Benzothiazole-2-carboxaldehyde	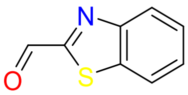	6639-57-2	163.200
080	2-Bromo-5-hydroxy-benzaldehyde	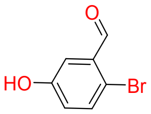	2973-80-0	201.019
081	2,4-Dichlorothiazole-5-carboxaldehyde	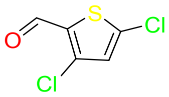	92972-48-0	182.030
082	Ethyl 4-formylpyrrole-2-carboxylate	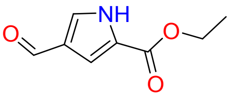	7126-57-0	167.163
083	1H-Indazole-3-carboxaldehyde	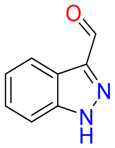	5235-10-9	146.149
084	2-Bromothiazole-5-carboxaldehyde	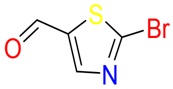	464192-28-7	192.036
085	2-Bromothiazole-4-carboxaldehyde	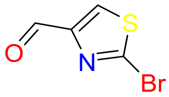	5198-80-1	192.036
086	4,6-Dichloro-2-(methylthio)pyrimidine-5-carboxaldehyde	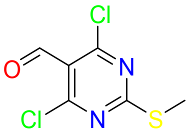	33097-11-9	223.083

Number	Name	Structure	CAS	M.W.
087	2-Chloro-3-fluoropyridine-4-carboxaldehyde	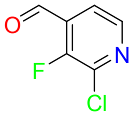	329794-28-7	159.547
088	5-Chloro-2-hydroxy-3-iodobenzaldehyde	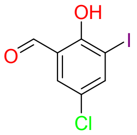	215124-03-1	282.460
089	4,5-Dimethylthiazole-2-carboxaldehyde	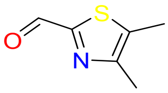	74531-15-0	141.194
090	5-Bromothiophene-3-carboxaldehyde	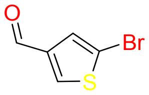	18791-79-2	191.048
091	1-Methyl-1H-indole-6-carboxaldehyde	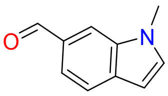	21005-45-8	159.187
092	5'-Bromo-2,2'-bithiophene-5-carboxaldehyde	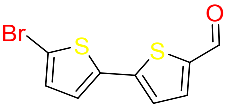	110046-60-1	273.174
093	Citral	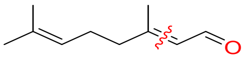	5392-40-5	152.236
094	Pyrrole-2-carboxaldehyde	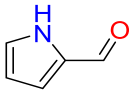	1003-29-8	95.1007
095	3-(Methylthio)butanal	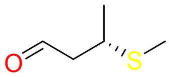	16630-52-7	118.199
096	2-Chloroisonicotin-aldehyde	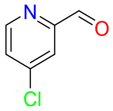	101066-61-9	141.557
097	4-Cyanobenzaldehyde	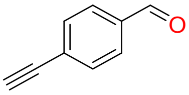	105-07-7	131.134
098	2-Formylpyridine	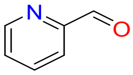	1121-60-4	107.112

Number	Name	Structure	CAS	M.W.
099	5-Bromonicotinaldehyde	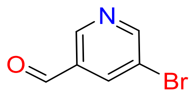	113118-81-3	186.008
100	2-Bromoisonicotin-aldehyde	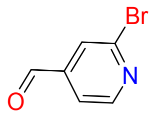	118289-17-1	186.008
101	2-Formylbenzoic acid	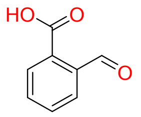	119-67-5	150.133
102	4-Hydroxybenzaldehyde	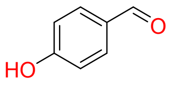	123-08-0	122.123
103	Butyraldehyde	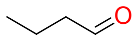	123-72-8	72.1065
104	4-(2-Pyridinyl)-benzaldehyde	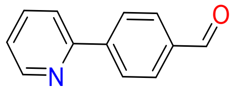	127406-56-8	183.209
105	4-Diethylaminosalicyl-aldehyde	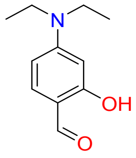	17754-90-4	193.245
106	1-Methylpyrrolo[2,3-b]pyridine-3-carbaldehyde	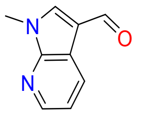	171919-36-1	160.176
107	1-Allyl-2-methyl-1H-indole-3-carbaldehyde	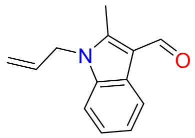	230283-19-9	199.252
108	1-(4-Methylphenyl)-pyrrole-2-carbaldehyde	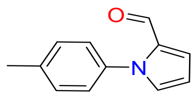	30186-38-0	185.225
109	4-Biphenylcarbaldehyde	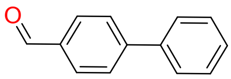	3218-36-8	182.221

Number	Name	Structure	CAS	M.W.
110	(2*Z*)-2-Phenyl-2-butenal	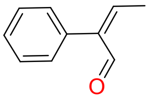	4411-89-6	146.188
111	Tetrahydro-2H-pyran-4-carbaldehyde	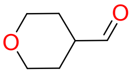	50675-18-8	114.143
112	*o*-Tolualdehyde	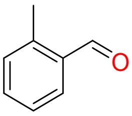	529-20-4	120.151
113	Quinaldaldehyde	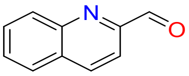	5470-96-2	157.172
114	4-Chloropicolinaldehyde	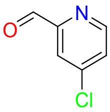	63071-13-6	141.557
115	4-Phenoxybenzalde-hyde	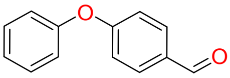	67-36-7	198.220
116	5,6-Dihydro-2H-pyran-3-carbaldehyde	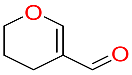	13417-49-7	112.128
117	*p*-Formylpyridine	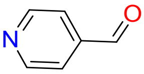	872-85-5	107.112
118	Salicylaldehyde	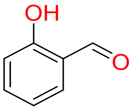	90-02-8	122.123
119	2,5-Dimethoxy-benzaldehyde	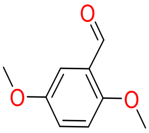	93-02-7	166.175
120	D-(+)-Glyceraldehyde	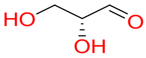	453-17-8	90.0776
121	(*R*)-(+)-Citronellal	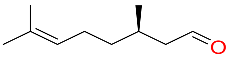	2385-77-5	154.252

Number	Name	Structure	CAS	M.W.
122	1-(Phenylsulfonyl)-2-pyrrolecarboxaldehyde	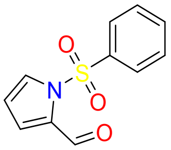	86688-93-9	235.262
123	5-[2-Chloro-5-(trifluoromethyl)-phenyl]furfural	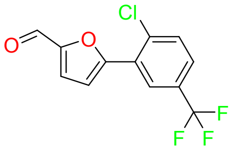	259196-40-2	274.625
124	3,5-Di-tert-butyl-2-methoxybenzaldehyde	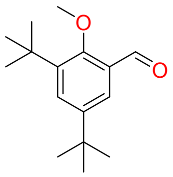	135546-15-5	248.365
125	2-Methyl-N-ethyl-N-(2-cyanoethyl)-4-aminobenzaldehyde	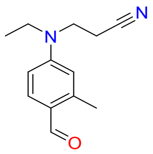	119-97-1	216.283
126	4-Phenylthiophene-2-carboxaldehyde	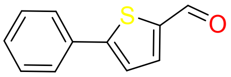	26170-87-6	188.250
127	2,3-Dimethoxy-1-naphthaldehyde	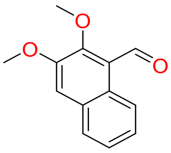	56252-09-6	216.235
128	6-(3-Thienyl)pyridine-2-carboxaldehyde	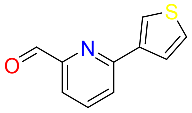	834884-76-3	189.238
129	3-Bromoben-zothiophene-2-carboxaldehyde	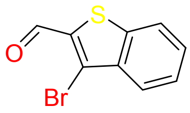	10135-00-9	241.108
130	3-Hydroxy-2-iodo-4-methoxybenzaldehyde	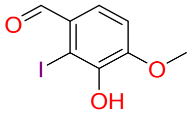	138490-94-5	278.041
131	5-(4-Bromophenyl)-2-chloropyridine-3-carboxaldehyde	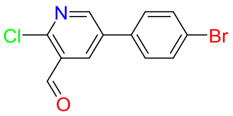	1119449-39-6	296.551

Number	Name	Structure	CAS	M.W.
132	3-Phenylaziridine-2-carboxaldehyde dimer	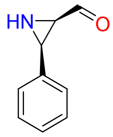	919101-04-5	147.176
133	3,4-Dibromothiophene-2-carboxaldehyde	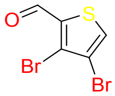	32896-02-9	269.944
134	3,5-Dibromopyridine-2-carboxaldehyde	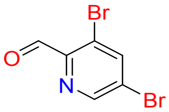	898559-25-6	264.904
135	Thieno[3,2-b]thiophene-2-carboxaldehyde	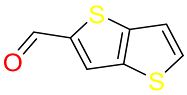	31486-86-9	168.240
136	1,2,4-Triazolo[1,5-a]pyridine-6-carboxaldehyde	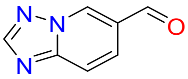	614750-81-1	147.137
137	4-Aminopyrimidine-5-carboxaldehyde	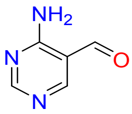	16357-83-8	123.115
138	6-Bromo-1H-indazole-4-carboxaldehyde	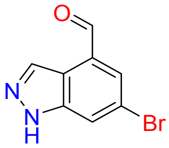	1444616-24-3	225.045
139	5-(Ethoxymethyl)furan-2-carboxaldehyde	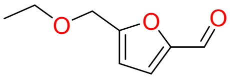	1917-65-3	154.164
140	(5-Methyl-2,4-dioxo-3,4-dihydro-1(2H)-pyrimi-dinyl)acetaldehyde	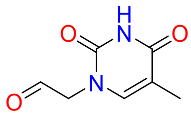	23485-31-6	168.152
141	2-Thiophene-carboxaldehyde	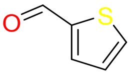	98-03-3	112.152
142	Trimethylacetaldehyde	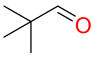	630-19-3	86.1334

Number	Name	Structure	CAS	M.W.
143	Hydroxycitronellal	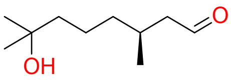	107-75-5	172.267
144	3-(5-Methyl-2-furyl)-butanal	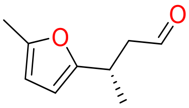	31704-80-0	152.192
145	Safranal	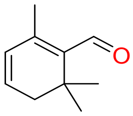	116-26-7	150.220
146	1-Naphthaldehyde	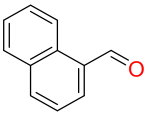	66-77-3	156.184
147	2-Naphthaldehyde	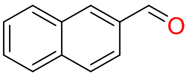	66-99-9	156.184
148	4-Methoxybenzaldehyde	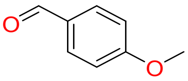	123-11-5	136.150
149	5-Bromoindole-3-carboxaldehyde	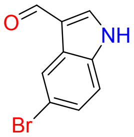	877-03-2	224.057
150	4-Chlorobenzaldehyde	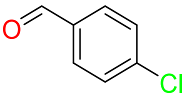	104-88-1	140.569
151	5-Chloroindole-3-carboxaldehyde	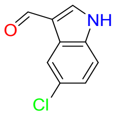	827-01-0	179.606
152	Cyclohexanecarboxaldehyde	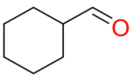	2043-61-0	112.171
153	2,4-Dimethoxy-benzaldehyde	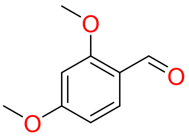	613-45-6	166.175

Number	Name	Structure	CAS	M.W.
154	5-Fluorobenzaldehyde	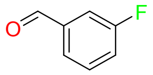	459-57-4	124.114
155	Indole-3-carboxaldehyde	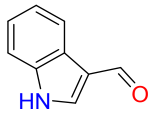	487-89-8	145.161
156	2-Methylpropion-aldehyde	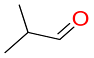	78-84-2	72.1065
157	Methional	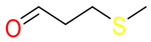	3268-49-3	104.173
158	5-Methoxyindole-3-carboxaldehyde	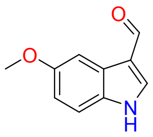	10601-19-1	175.186
159	4-(Methylthio)-benzaldehyde	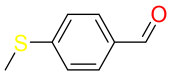	3446-89-7	152.217
160	Propionaldehyde	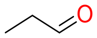	123-38-6	58.0796
161	4-(Tert-Butyloxy)-benzaldehyde	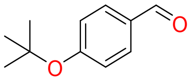	57699-45-3	178.230
162	4-(Trifluoromethyl)-benzaldehyde	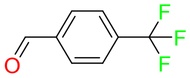	455-19-6	174.121
163	Phenyl acetaldehyde	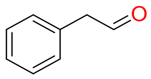	122-78-1	120.151
164	Trimethoxy-benzaldehyde	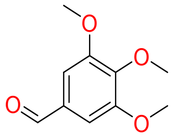	86-81-7	196.201
165	4-Carboxybenzaldehyde	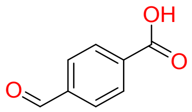	619-66-9	150.133

References
